# Organocatalytic Michael Addition of Unactivated α-Branched Nitroalkanes to Afford Optically Active Tertiary Nitrocompounds

**DOI:** 10.1021/acs.orglett.3c03340

**Published:** 2023-11-27

**Authors:** Beñat Lorea, Ane García-Urricelqui, José M. Odriozola, Jesús Razkin, Maialen Espinal-Viguri, Mikel Oiarbide, Antonia Mielgo, Jesús M. García, Claudio Palomo

**Affiliations:** † Departamento de Química Orgánica I, Universidad del País Vasco UPV/EHU, Manuel Lardizábal 3, 20018 San Sebastián, Spain; ‡ Departamento de Ciencias, Institute for Advanced Materials and Mathematics (InaMat^2^), Universidad Pública de Navarra (UPNA), 31006 Pamplona, Spain

## Abstract

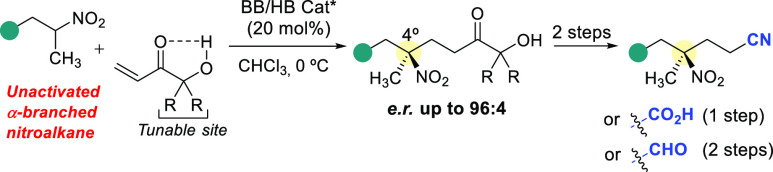

The direct, asymmetric conjugate addition of unactivated α-branched nitroalkanes is developed based on the combined use of chiral amine/ureidoaminal bifunctional catalysts and a tunable acrylate template to provide tertiary nitrocompounds in 55–80% isolated yields and high enantioselectivity (*e.r.* up to 96:4). Elaboration of the ketol moiety in thus obtained adducts allows a fast entry to not only carboxylic and aldehyde derivatives but also nitrile compounds and enantioenriched 5,5-disubstituted γ-lactams.

Stereoselective methods for the preparation of α-stereogenic nitrocompounds are highly appealing owing to the synthetic versatility of the nitro group.^1^ For example, reduction would lead to the corresponding α-stereogenic amines, which are widespread substructures within natural products and bioactive compounds.^2^ In addition, natural products that contain the nitro group are known to exhibit a wide range of biological activities.^3^ However, the number of nitro-containing molecules under development within drug discovery programs is marginal, in part because the nitro functionality is classified as a “structural alert”,^4^ a situation that may result in missed opportunities.^1b^ Another problem is that current enantioselective methodologies to prepare α-stereogenic nitrocompounds are not general.^5^ In particular, the catalytic, asymmetric α-functionalization of secondary (α-branched) nitroalkanes progresses slowly (Figure 1). Catalytic methodologies have been described for accessing optically active tertiary nitrocompounds bearing no α-stereocenter (two identical R^1^ substituents)^6,7^ or an activating α-substituent Z, with Z = COR, CO_2_R, CN, or similar.^8^ The success of these latter methods is strongly bound to the presence of an electron-withdrawing substituent, which can ease formation of the transient nitronate anion while providing an additional handle for catalyst binding. In sharp contrast, methods for direct, highly enantioselective α-functionalization of α-aryl and α-alkyl nitroalkanes remain underdeveloped. Steric constraints toward electrophiles dictated by the R^1^/R^2^ substituents in the transient nitronate **A** (Figure 1) may account for the low reactivity observed, while discrimination across the two enantiotopic faces in **A** becomes increasingly challenging as the similarity in size and electronic nature of R^1^ vs R^2^ increases.

**Figure 1:**
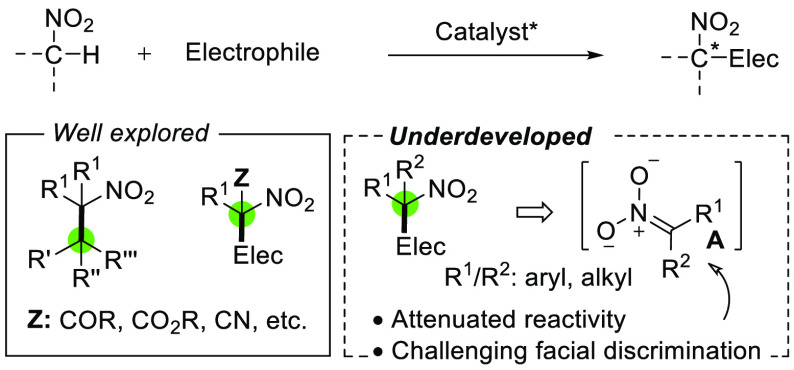
Asymmetric C_α_-functionalization of α-branched nitroalkanes leading to tertiary nitrocompounds.

Efforts toward overcoming these issues are rare in the literature (Scheme 1). Yamaguchi described an organocatalytic conjugate addition of α-branched nitroalkanes to enones using 5–10 mol % of l-proline and 4-silyloxy l-proline rubidium salts (Scheme 1a),^9^ although no data regarding the nitronate facial selectivity were reported. A couple of reports involving transition metal catalysis and chiral *P,N*-ligands are known. Kanai and Shibasaki^10^ reported the palladium-catalyzed allylic alkylations of secondary nitroalkanes with the assistance of 10 mol % of base, typically DBU (1,8-diazabicyclo[5.4.0]undec-7-ene), but attempts to get asymmetric induction at the α-position of the nitro functionality resulted in suboptimality (≤49% enantioselectivity obtained, Scheme 1b). More recently, Trost^11^ described the palladium-catalyzed decarboxylative allylic alkylation of nitroesters affording α-aryl, α-allylic nitroalkanes with high enantioselectivities (Scheme 1c). Finally, Hyster reported an innovative enzymatic photoredox α-alkylation of nitroalkanes (Scheme 1d) which, however, shows strong substrate dependence.^12^ With the existing limitations in mind and the apparent lack of any successful asymmetric organocatalytic approach toward α-stereogenic α-aryl/alkyl and α-alkyl/alkyl tertiary nitrocompounds, we set out to investigate the Brønsted base-catalyzed additions of unactivated α-branched nitroalkanes to well suited Michael acceptors. Here, our preliminary results along these lines are presented which demonstrate the feasibility of such a realization upon proper combination of chiral amine/ureidoaminal bifunctional catalysts and α′-hydroxy enones **2** as an acrylic ester/aldehyde surrogate with a tunable *gem*-disubstitution (Scheme 1e).

**Scheme 1:**
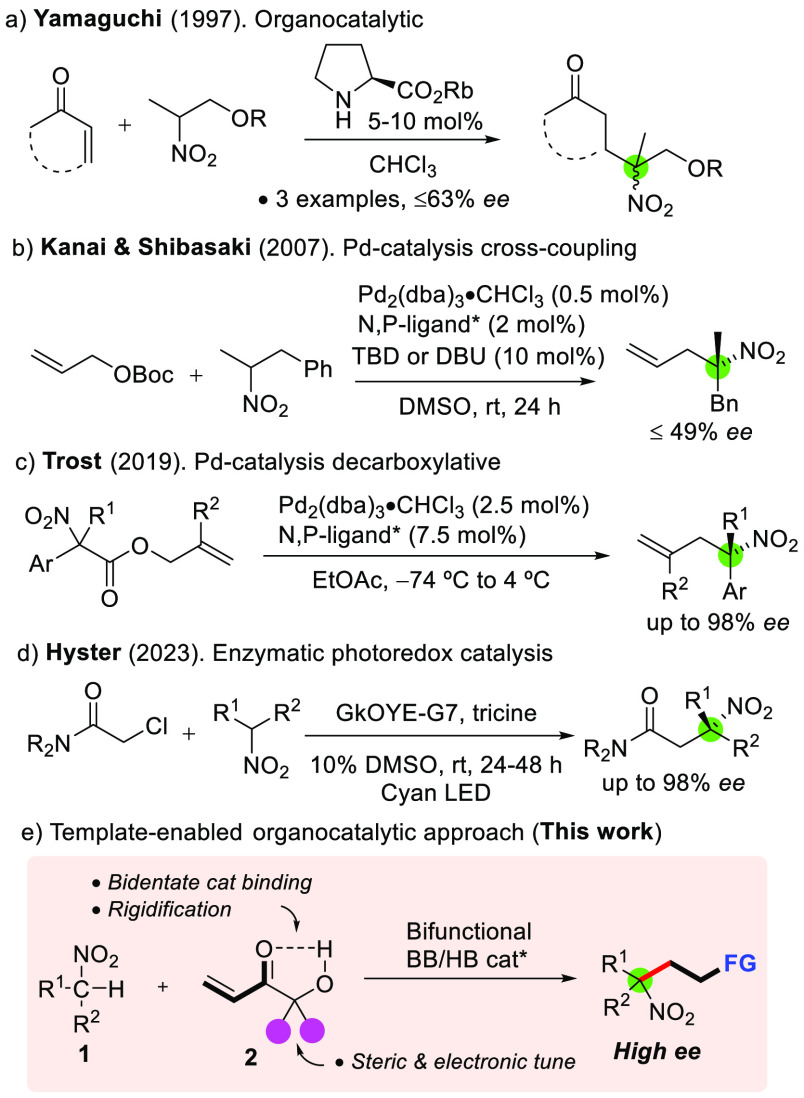
Efforts on Catalytic Asymmetric α-Functionalization of α-Branched Alkyl/Aryl Nitroalkanes

Previous research from these laboratories has shown that acrylic ester/aldehyde surrogates **2** fit well in Michael addition reactions that proceed through either H-bonding or metal-chelation mediated activation mechanisms.^13^ The ability of the ketol moiety to act as a bidentate H-bond donor/acceptor and thus tightly bind to the bifunctional organocatalyst was believed to be crucial in these developments. In addition, intramolecular H-bonding in **2** should also increase enone innate electrophilicity while favoring transition state rigidification. In this context, we envisioned that these features might counterbalance the alleged low reactivity of α-disubstituted nitronates **A** and eventually induce threshold enantioface discrimination. For the initial assessment, we commenced by studying the reaction of 1-phenyl-2-nitropropane **1A** with α′-hydroxy enone **2a** in the presence of various chiral bifunctional organobases. The reactions using 20 mol % of the popularized thiourea and squaramide-type catalysts or related ones^14,15^ proceeded smoothly at room temperature in chloroform to afford adduct **3Aa**. Although enantioselectivities were marginal in these cases, these experiments proved the organocatalytic approach was indeed feasible.^16^ Then, we turned our attention to urea-aminal type catalysts, which have the capability for multiple H-bonding interactions^17^ (Table 1) and might facilitate better stereocontrol. The reaction with catalyst **C1** reached complete conversion after 20 h at room temperature and led to a product of 78:22 *e.r.* (entry 1). With this promising result in hand, a series of related catalysts with varying substituents at the aminal carbon (R group) and the acyl termination were evaluated next. The change from R = ^t^Bu (**C1**) to R = ^i^Pr (**C2**) led to a small decrease in the enantioselectivity (entry 1 vs 2). However, the configuration of the aminal carbon in the catalyst should be preserved (*S*), as changing it to (*R*), cat **C3**, decreased both the yield and the enantioselectivity (compare entries 2 and 3). Modifying the aminal *N*-acyl side chain had a substantial impact. Thus, compared with the Fmoc carbamate **C1**, *tert*-butyl carbamate **C4** resulted in inferiority, but the naphthylmethyl carbamate **C5** led to a similar 75:25 *e.r.* (entries 1 and 4 vs 5). Gratifyingly, the larger arylmethyl carbamates **C6** and **C7**, derived from 4-pyrenylmethanol and 9-anthracenylmethanol, respectively, accomplished even better results, leading to product **3Aa** in good isolated yields (70% and 74%) and about 90:10 *e.r.* (entries 6 and 7). Finally, catalysts with an additional α-amino acid residue attached, such as **C8** and **C9**, did not improve the reaction outcome (entries 8 and 9).^18^

**Table 1:**
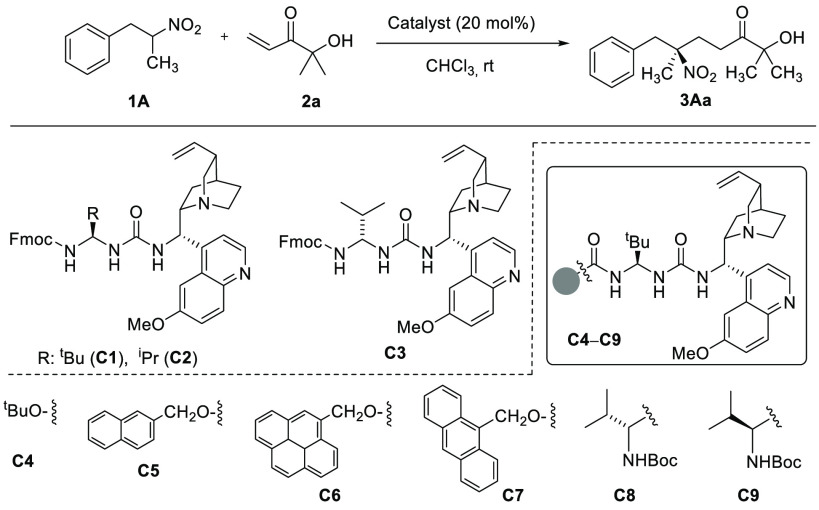
Catalyst Screening for the Reaction of **1A** with **2a**^a^

entry	cat.	*t* (h)^b^	yield (%)^c^	*e.r.*^d^
1	**C1**	20	nd	78:22
2	**C2**	23	81	75:25
3	**C3**	26	68	62:38
4	**C4**	48	nd	65:35
5	**C5**	36	nd	75:25
6	**C6**	20	70	89:11
7	**C7**	14	74	90:10
8	**C8**	22	76	72:28
9	**C9**	43	80	65:35

a Reactions conducted on a 0.2 mmol scale in 0.6 mL of CHCl_3_ (mol ratio **1A**/**2a**/catalyst 5:1:0.2). nd: Not determined.

b Time for full conversion.

c Yield of the isolated product.

d Determined by chiral HPLC.

With 20 mol % **C7** in CHCl_3_ at rt set as the best standard conditions, the influence of the nature of the two geminal R groups on template **2** in the reaction outcome was next investigated. Initial experiments showed that increasing the size of the R alkyl groups (*n*Pr, *i*Bu) caused a decrease in selectivity (see Supporting Information Table on page S27). The screening of acceptors **2** was then expanded to other alkyl- and aryl-substituted congeners.^16^ Interestingly, the aryl-substituted hydroxy enones were also competent acceptors (Table 2, entries 3, 4, and 6). The fluorinated derivatives **2c** and **2d** were found to be more reactive than **2b**, allowing one to carry out the reaction at 0 °C (entries 5 and 7), and among them, **2c** provided the same selectivity as **2a** at room temperature (entries 1 and 4); however, at 0 °C, it afforded the highest enantioselectivity (entry 5, 96:4 *e.r.*). On the other hand, enone **2e** featuring a cyclohexyl moiety was also efficient in terms of selectivity at rt, but it was much less reactive (entry 8, 39 h reaction). From these experiments, it seems that for this reaction the 3,5-bis(trifluoromethyl)phenyl substituents exhibit the best compromise between electronic and steric effects.

**Table 2:**
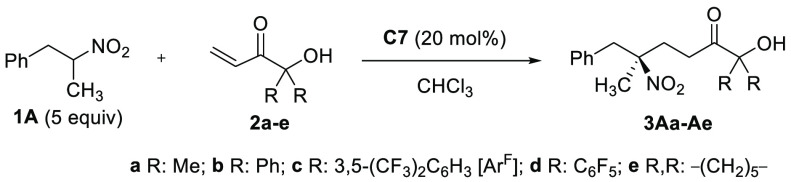
Influence of the R Groups of Enone Template **2**^a^

entry	R, R	*T* (°C)	*t* (h)	prod.	yield (%)^b^	*e.r.*^d^
1	Me	rt	14	**3Aa**	74	90:10
2	Me	0	96	**3Aa**	55^c^	91:9
3	Ph	rt	24	**3Ab**	70	84:16
4	Ar^F^	rt	16	**3Ac**	72	90:10
5	Ar^F^	0	93	**3Ac**	70	96:4
6	C_6_F_5_	rt	15	**3Ad**	64	84:16
7	C_6_F_5_	0	93	**3Ad**	67	91:9
8	–(CH_2_)_5_–	rt	39	**3Ae**	66	89:11

a Reactions run at 0.2 mmol scale in 0.6 mL of CHCl_3_ (mol ratio **1A**/**2**/**C7** 5:1:0.2).

b Yield for full conversion.

c Conversion.

d Determined by HPLC.

Under the above optimized conditions (catalyst **C7** in CHCl_3_ at 0 °C), the reaction scope was investigated (Table 3). Gratifyingly, various nitroethanes **1** with a *m*- or *p*-substituted phenylmethyl branch reacted with **2c** satisfactorily to afford the corresponding addition products **3Ac**–**3Ec** and **3Gc**–**3Ic** in good yields and with enantiomeric ratios ranging from the lowest 90:10 to the highest 96:4. Nitroethane **1F**, bearing an *o*-tolylmethyl substituent, and **1J**, bearing a *m*-disubstituted phenylmethyl group, led to products **3Fc** and **3Jc** with slightly eroded selectivities (89:11 and 87:13 *e.r.*). Bicyclic arylmethyl systems such as 1- and 2-naphthylmethyl nitroethanes **1K** and **1L** and guaiacol-derived nitroethane **1M** were also competent providing products **3Kc**, **3Lc**, and **3Mc** in 93:7, 95:5, and 94:6 *e.r.*, respectively. Even nitroalkane **1O**, with two simple alkyl groups at Cα, provided the product **3Oc** with an acceptable 81:19 *e.r.*, constituting a rare example of nonenzymatic enantioselective access to this kind of tertiary nitrocompound. On the other hand, the present method appears less suitable for α-aryl branched nitroalkanes, as shown in the moderate enantioselectivity (62:38 *e.r.*) with which product **3Nc** is obtained from nitroethane **1N**. Finally, obtention of products **3Ea**, **3Fa**, **3Ja**, **3Na**, and **3Oa** in decent to good enantioselectivities proved the method can be applied using the parent α′-hydroxy enone **2a**, a template easier to prepare in large quantity than **2c**.

**Table 3:**
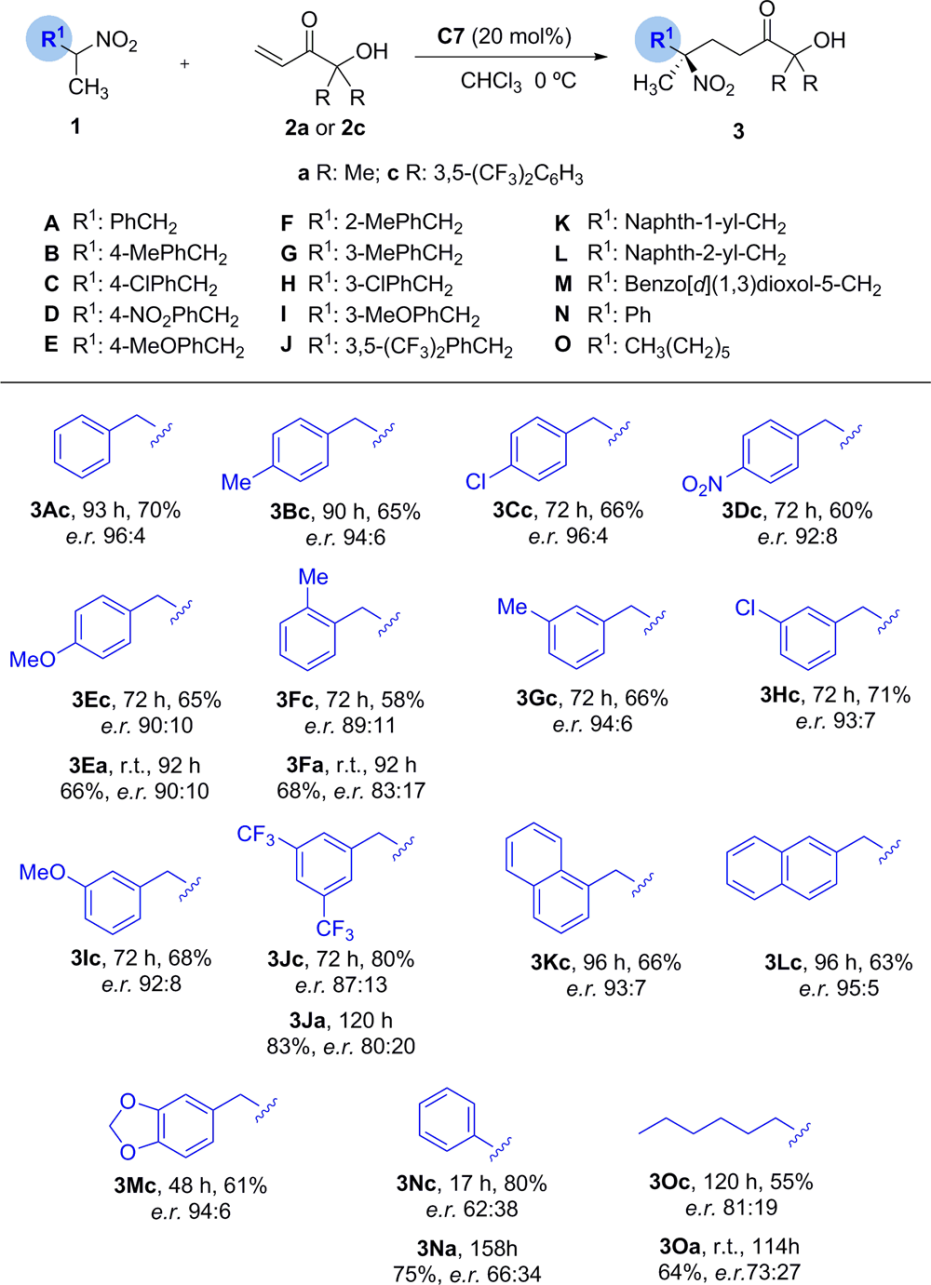
Substrate Scope for the Reaction of **1** with **2a**/**c**^a^

a Reactions run at 0.2 mmol scale in 0.6 mL of CHCl_3_ (mol ratio **1**/**2a** or **2c**/**C7** 5:1:0.2). Yields of isolated products. *e.r.* determined by chiral HPLC.

Both the nitro and the ketol groups in the thus obtained adducts could be transformed conveniently. For instance, as shown in Scheme 2, oxidation of **3Ac**, **3Cc**, **3Gc**, and **3Lc** with periodic acid afforded carboxylic acids **4** in 75–80% isolated yields. Alternatively, reduction of **3Lc** with borane and subsequent 1,2-diol oxidation afforded aldehyde **5** in a good yield. In these transformations, 3,3′,5,5′-tetrakis(trifluoromethyl)benzophenone was obtained in 80–84% yields which could be recycled for the preparation of **2c**.^16^ The γ-nitro carboxylic acids **4** could then be converted into 5,5-disubstituted γ-lactams.^19^ Thus, esterification of acids **4C** and **4G**, ulterior reduction of the nitro group in nitro esters **6C** and **6G** with NaBH_4_/NiCl_2_, and subsequent treatment with an aqueous solution of K_2_CO_3_ gave the γ-lactams **7C** and **7G** in high overall yield.^20^ The synthetic utility of this methodology was further illustrated with a successful two-step conversion of the ketol moiety into the corresponding nitrile. Thus, condensation of **3Lc** with hydroxylamine in hot ethanol produced oxime **8** in 71% yield. Subsequent acetylation of **8** in the presence of triethylamine was accompanied by spontaneous fragmentation giving rise to nitrile **9** in 85% isolated yield.^21^ In this process, the diaryl ketone byproduct was, again, recovered in 80% yield. The absolute configuration of compound **9** was determined by single crystal X-ray analysis which served to establish that of the remaining adducts.

**Scheme 2:**
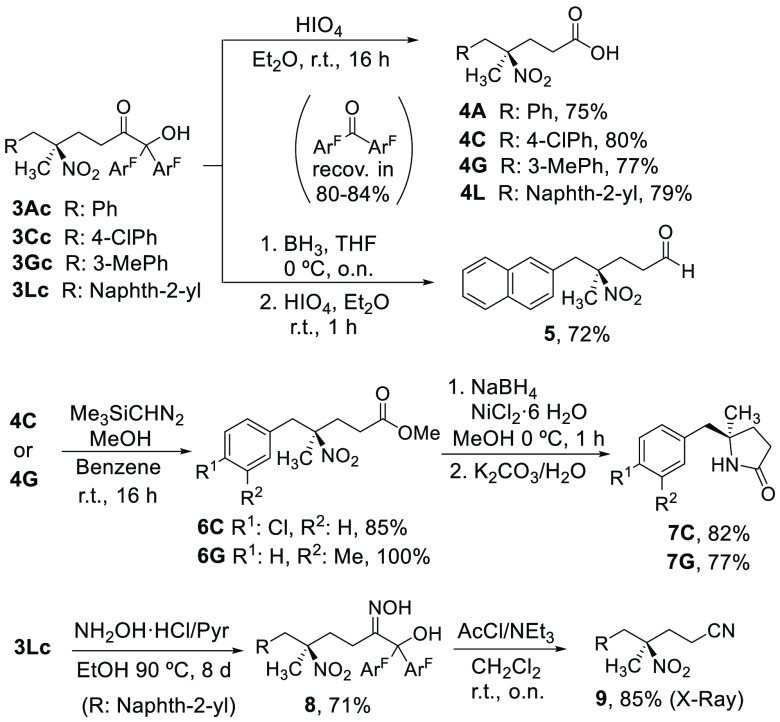
Chemical Elaboration of Adducts

The superior performance of ureidoaminal-based catalysts, i.e., **C7**, as compared with (thio)urea- and squaramide-based catalysts in these reactions might be ascribed to the capacity of the former to lead to highly ordered transition states (TSs) involving at least one additional hydrogen-bonding interaction. Figure 2 shows two plausible TS structures for the key C–C bond forming step in which the nitronate *Re*-face approaches the enone *Si*-face in concordance with the experimentally observed main product isomer. While the simultaneous coordination of the Nuc/Elec pair of reactants to the protonated catalyst obeys the Takemoto-type geometry in **TS**_**A**_, in **TS**_**B**_ the opposite, Pápai-type geometry would operate.^23,24^ As depicted in both models, the ketol hydroxy and carbonyl groups would probably be hydrogen-bonded internally, contributing to substrate activation and TS conformational rigidification. However, the nonchelated situation with both hydroxy and carbonyl groups hydrogen-bonded to the catalyst exclusively cannot be discarded.

**Figure 2:**
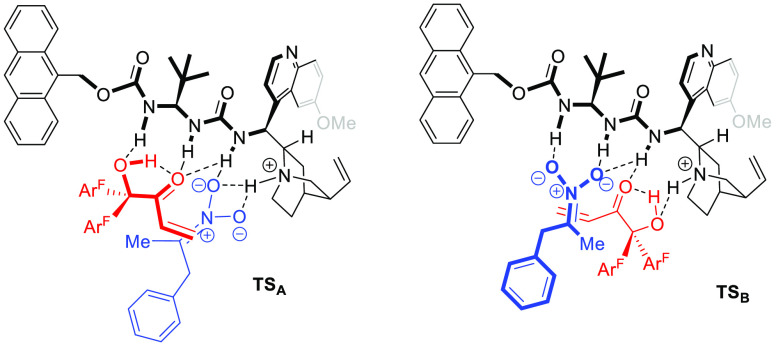
Plausible TS’s for the key C–C bond formation.

In summary, a new approach to enantioenriched α-tertiary nitrocompounds via unprecedented organocatalytic Michael addition of unactivated α-branched nitroalkanes is reported. The method is based on: (i) key advantages as acceptors of newly developed α′-hydroxy enones which serve as surrogates of acrylic acid/ester,^25^ aldehyde, and nitrile functionalities equally and (ii) the combined use of cinchona alkaloid-derived ureidoaminal catalysts. This methodology provides alternative routes to access relevant compound families in optically active form, inter alia 5,5-disubstituted γ-lactams bearing a quaternary stereocenter, whose enantioselective synthesis is still a challenge.

## Data Availability

The data underlying this study are available in the published article and its Supporting Information.

## Supporting Information Available

Experimental procedures, spectroscopic data of products, ^1^H NMR and ^13^C NMR spectra, HPLC charts, and crystallographic data (PDF)
