# Supplementary material for: Organocatalytic Michael Addition of Unactivated α-Branched Nitroalkanes to Afford Optically Active Tertiary Nitrocompounds
Source: Org Lett. 2023 Nov 27;25(48):8590–5. doi: 10.1021/acs.orglett.3c03340 (PMC10714440; doi:10.1021/acs.orglett.3c03340)
Supplement: Supplementary file 1 — ol3c03340_si_001.pdf [file ol3c03340_si_001.pdf]

SUPPORTING INFORMATION

Organocatalytic Michael Addition of Unactivated α-Branched Nitroalkanes to Afford Optically Active Tertiary Nitrocompounds

Beñat Lorea,[b] Ane García-Urricelqui,[a] José M. Odriozola,[b] Jesús Razkin,[b] Maialen Espinal-Viguri,[b] Mikel Oiarbide,[a]* Antonia Mielgo,[a]* Jesús M. García,[b]* and Claudio Palomo[a]*

[a] *Departamento de Química Orgánica I. Universidad del País Vasco UPV/EHU. Manuel Lardizábal 3, 20018-San Sebastián.*

[b] *Departamento de Ciencias. Institute for Advanced Materials and Mathematics (InaMat^2). Universidad Pública de Navarra (UPNA). 31006-Pamplona.*

Table of Contents

1. **Materials and general techniques** S2
2. **Tested Catalysts in the addition reaction. Preparation** S4
   - **2.1. Dipeptide derived catalysts C8-C10** S5
   - **2.2. Dipeptide derived catalyst C11** S10
   - **2.3. Tripeptide derived catalysts C12-C13** S11
   - **2.4. Preparation of catalyst C15** S15
3. **Preparation of starting materials** S17
   - **3.1. Synthesis of nitroalkanes 1A-1M and 1O** S17
   - **3.2. Synthesis of α-hydroxy enones 2a-2m** S20
4. **Catalytic conjugate additions** S26
   - **4.1. Catalyst screening for the model reaction between 1A and 2a** S26
   - **4.2. Acceptor screening** S27
   - **4.3. Reaction scope** S32
5. **Chemical elaboration of adducts** S40
6. **Representative NMR spectra** S45
7. **HPLC chromatograms for *e.r.* determinations** S120
8. **ORTEP diagram of compound 9** S155

1. Materials and general techniques

All non-aqueous reactions were performed under inert atmosphere using oven-dried glassware and were magnetically stirred. Yields refer to chromatographically purified and spectroscopically pure compounds, unless otherwise stated.

Heat requiring reactions were performed using a hotplate with a sand or an oil bath and a condenser. Reactions requiring low temperatures were performed using cooling bath circulators *Huber* T100E and acetone or isopropanol baths.

Organic layers washed with aqueous phases were dried over $MgSO_4$ or Na_2SO_4 and filtered through cotton. Organic solvents were evaporated under reduced pressure using rotavapors Büchi R-100, R-200 and R-210, the latter equipped with a Büchi V-700 vacuum pump and a Büchi V-850 vacuum controller, appropriate for the evaporation of solvents when products were volatile compounds. For the complete removal of solvents vacuum pump Telstar Top-3 (≈ 0.5 mmHg) was employed.

Solvents and reagents

Reagents were purchased from different commercial suppliers (Aldrich, Across, Alfa Aesar, Fluka, TCI, Merck, Fluorochem, etc.), stored as specified by the manufactured and used without previous purification unless otherwise stated.

Triethylamine was purified by distillation. When anhydrous solvents were required, they were dried following established procedures.[1] Dichloromethane (CH_2Cl_2) was dried over CaH_2 and tetrahydrofuran was distilled over sodium/benzophenone. Analytical reagent grade MeOH, $CHCl_3$, Et_2O and toluene were used without further drying. Analytical reagent grade DMF was dried over molecular sieves.

Chromatography

Reactions and flash chromatographic columns were monitored by thin layer chromatography (TLC) using Merck silica gel 60 F254 plates and visualized by fluorescence quenching under UV light, Fisher Biolock lamp VL-4LC, λ = 254 and 365 nm. In addition, TLC plates were stained with a dipping solution of potassium permanganate (1g) in 100 ml of water (limited lifetime), followed by heating.

Chromatographic purification was performed on Merck ROCC 60 silica gel 40-63 μm as stationary phase and a suitable mixture of solvents (typically hexane: ethyl acetate, pentane: diethyl ether or dichloromethane: methanol) as eluent.

Optical rotation

Optical rotations were recorded using a Jasco P-2000 polarimeter; specific rotations (SR) ($[\alpha]_D$) are reported in 10^{-1} $deg.cm^2.g^{-1}$; concentrations (*c*) are quoted in g/100 mL; $_D$ refers to the D-line of sodium (589 nm); temperatures (T) are given in degree Celsius (°C).

[1] Armarego, W. L. F.; Perrin, D. D., *Purification of Laboratory Chemicals,* 3rd Edition Butterworth-Heinemann, Oxford, **1988**.

Melting points

Melting points were determined in open capillaries in a Stuart SHP3 melting point apparatus and microscope and were uncorrected.

NMR spectra

^{1}H NMR and ^{13}C NMR spectra were recorded at 300 MHz or 400 MHz and 75 MHz or 100 MHz respectively. ^{19}F NMR spectra were recorded at 471MHz. The chemical shifts are reported in ppm relative to $CDCl_3$ (δ = 7.26), CD_3OD (δ = 3.31) and DMSO-d_6 (δ = 2.50) for ^{1}H NMR and relative to the central resonances of $CDCl_3$ (δ = 77.0), CD_3OD (δ = 49.15) and DMSO-d_6 (δ = 39.5) for ^{13}C NMR. The multiplicity of each signal is designated using the following abbreviations: s, singlet; d, doublet; dd, doublet of doublets; ddd, doublet of doublets of doublets; t, triplet; dt, doublet of triplets; q, quartet; dq doublet of quartets; m, multiplet; dm, doublet of multiplets; tm, triplet of multiplets; s_b, broad singlet. Coupling constants (*J*) are reported in Hertz (Hz). MestrReNova Mnova 11.0.4 program was used to process and edit the registered spectra.

Mass spectra

Mass spectra were recorded on an ESI-ion trap Mass spectrometer (Agilent 1100 series LC/MSD, SL model) on a UPLC-DAD-QTOF, Ultra High Performance Liquid Chromatography-Mass spectrometer, Waters UPLC ACQUITY, Waters PDA detector, Waters Sunapt G2 or on an Agilent Thermoquest LCT spectrometer. Mass spectrometry analyses were performed in the General Research Service (SGIker) of the University of the Basque Country (UPV/EHU).

Determination of enantiomeric excesses

Enantiomeric excesses were determined using analytical high performance liquid chromatography (HPLC) performed on Waters 600-E (equipped with 2996 and 2998 photodiode array UV detector) and on JASCO LC-Net II/ADC (equipped with photodiode array UV and circular dichroism detector) employing Daicel Chiralpak (IA, IB, IC, OD-H) and Phenomenex Lux 3 µm (Cellulose 1, Cellulose 2) columns.

X-Ray diffraction analysis

The X-ray diffraction analysis experiments were conducted in the General Research Service (SGIker) of the University of the Basque Country (UPV/EHU) using a SuperNova, Dual, Cu at home/near, HyPix diffractometer.

2. Tested catalysts in the addition reaction. Preparation

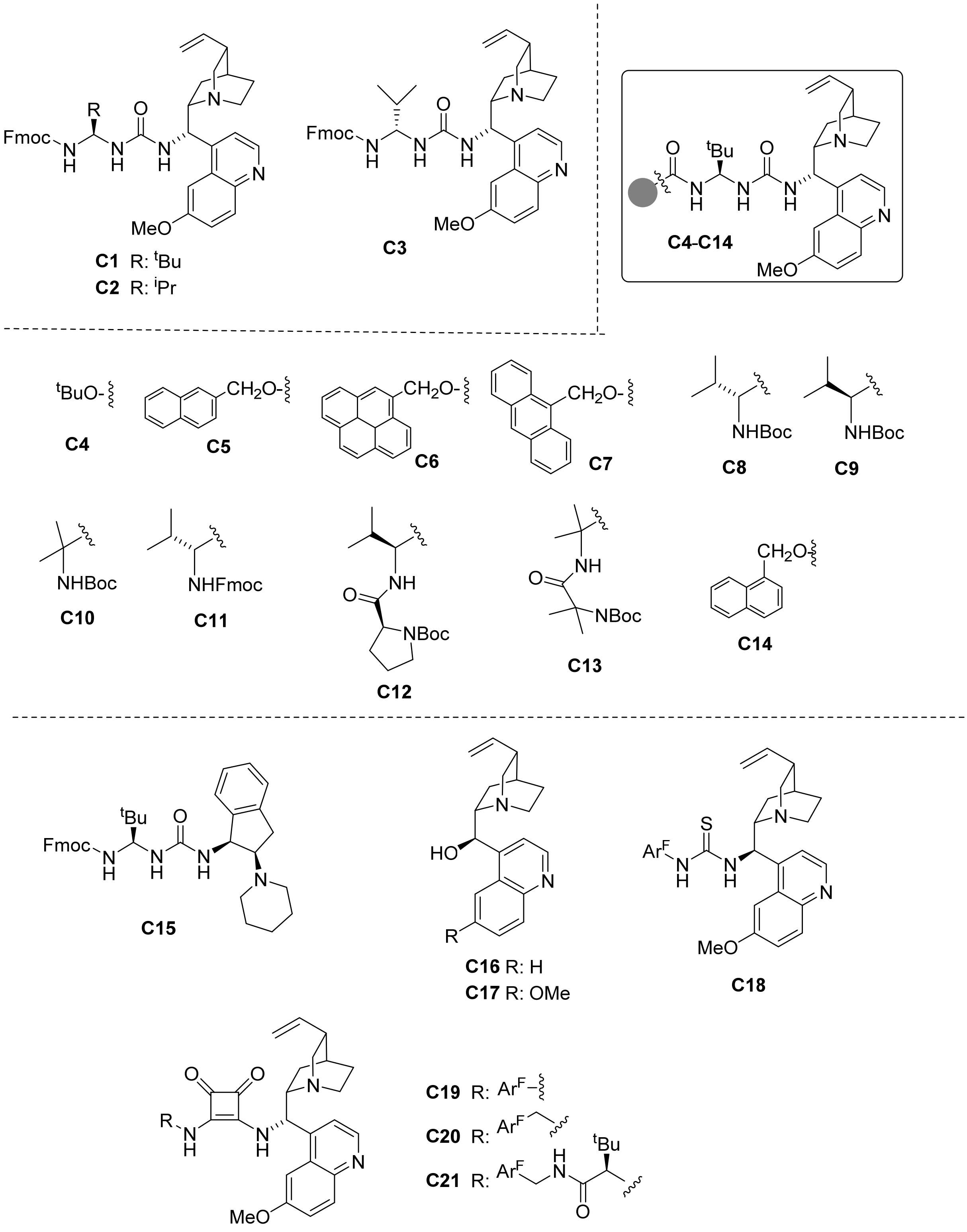

Catalysts **C16** and **C17** were commercially available. Catalysts **C1,**[2] **C2**,[3] **C3,**[3] **C4,**[3] **C5,**[2] **C6,**[4] **C7,**[2] **C14,**[5] **C18,**[6] **C19,**[7] **C20,**[8] **C21,**[4] were prepared following reported protocols.

2.1. Dipeptide derived catalysts C8-C10

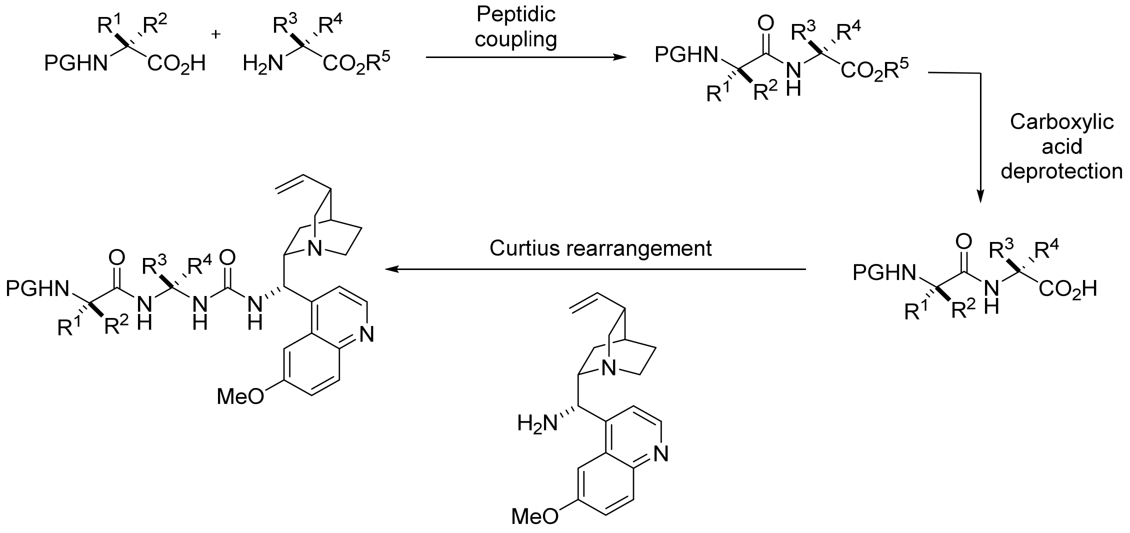

Step 1: Peptidic coupling

PROCEDURE 1: **Boc-(*L*)-Val-(*L*)^{t}Leu-OMe**[9]

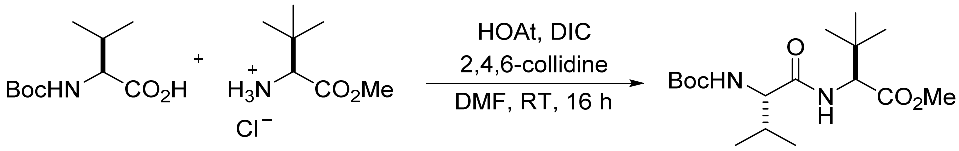

To a solution of commercially available Boc-*L*-Val-OH (1.183 g, 5.45 mmol, 1 eq) and H-(*L)*-^{t}Leu-OMe chlorohydrate[10] (1.089 g, 6 mmol, 1.1 eq) in DMF (16 mL), HOAt (858 mg, 6.3 mmol, 1.15 eq) was added. The resulting mixture was stirred under N_2 at RT for 20 min and then cooled down to 0 °C. DIC (1.27 mL, 8.18 mmol, 1.5 eq) and 2,4,6-collidine (0.8 mL, 6 mmol, 1.1 eq) were added and the solution was stirred at RT for 16 h. The reaction mixture was diluted with EtOAc and the organic phase washed

[2] Diosdado, S.; Etxabe, J.; Izquierdo, J.; Landa, A.; Mielgo, A.; Olaizola, I.; López, R.; Palomo, C. *Angew. Chem. Int. Ed.* **2013**, *52*, 11846-11851.
[3] Diosdado, S.; López, R.; Palomo, C. *Chem. Eur. J.* **2014**, *20*, 6526-6531.
[4] García-Urricelqui, A.; de Cózar, A.; Mielgo, A.; Palomo, C. *Chem. Eur. J.* **2021**, *27*, 2483-2492.
[5] Duñabeitia, E.; Landa, A.; López, R.; Palomo, C. *Org. Lett.* **2023**, *25*, 125-129.
[6] Vakulya, B.; Varga, S.; Csámpai, A.; Soós, T. *Org. Lett.* **2005**, *7*, 1967-1969.
[7] a) Dai,L.; Wang, S.-X.; Chen, F.-E. *Adv. Synth. Catal.* **2010**, *352*, 2137-2141. b) Yang, W.; Du, D. M.; *Org. Lett.* **2010**, *12*, 5450-5453.
[8] a) Malerich, J. P.; Hagihara, K.; Rawal, V. H. *J. Am. Chem. Soc.* **2008**, *130*, 14416-14417. b) Jiang, H.; Paixão, M. W.; Monge, D.; Jørgensen, K. A. *J. Am. Chem. Soc.* **2010**, *132*, 2775-2783.
[9] Adapted from: Babine, Robert Edwards, et al. *PTC Int. Appl.* 2002018369, **2002**.
[10] Synthesis procedure from: Rodrigues, L. M.; Fonseca, J. I.; Maia, H. L. S. *Tetrahedron* **2004**, *60*, 8929-8936.

with 1M HCl, saturated $NaHCO_3$ and brine (x5) and dried over $MgSO_4$. The solvents were evaporated under reduced pressure and the crude was purified by silica flash column chromatography (Hexane: EtOAc 80:20). White solid, 1.376 g, 4 mmol, 73% yield. ^{1}H NMR (300 MHz, $CDCl_3$) δ 6.47 (d, J = 8.7 Hz, 1H), 5.05 (d, J = 8.6 Hz, 1H), 4.42 (d, J = 9.3 Hz, 1H), 3.87 (dd, J = 8.6, 6.7 Hz, 1H), 3.70 (s, 3H), 2.11 (m, 1H), 1.42 (s, 9H), 1.06 – 0.83 (m, 15H).

PROCEDURE 2: **Boc-(*D*)-Val-(*L*)^{t}Leu-OBn**

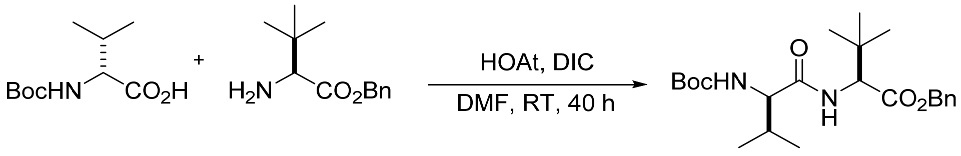

To a solution of Boc-(*D*)-Val-OH[11] (391.1 mg, 1.8 mmol, 1 eq) and H-(*L*)-^{t}Leu-OBn (438 mg, 1.98 mmol, 1.1 eq) in DMF (5.4 mL), HOAt (318.5 mg, 2.34 mmol, 1.3 eq) was added. The resulting mixture was stirred under N_2 at RT for 20 min and then cooled down to 0 °C. DIC (0.42 mL, 2.7 mmol, 1.5 eq) was added and the solution was stirred at RT for 16 h. The reaction mixture was diluted with EtOAc and the organic phase washed with 1N HCl (x3), saturated $NaHCO_3$ (x3) and brine (x5) and dried over $MgSO_4$. The solvents were evaporated under reduced pressure and the crude was purified by silica flash column chromatography (Hexane: EtOAc 90:10). White solid, 721 mg, 1.71 mmol, 71% yield. ^{1}H NMR (300 MHz, $CDCl_3$) δ 7.35 (s, 3H), 5.15 (d, J = 8.5 Hz, 1H), 4.95 (s, 1H), 4.49 (d, J = 9.4 Hz, 1H), 3.98 (s, 1H), 2.37 – 2.13 (m, 1H), 1.45 (s, 9H), 1.04 – 0.91 (m, 12H), 0.88 (d, J = 6.9 Hz, 4H).

PROCEDURE 3:[9] **Boc-AIB-(*L*)^{t}Leu-OMe**

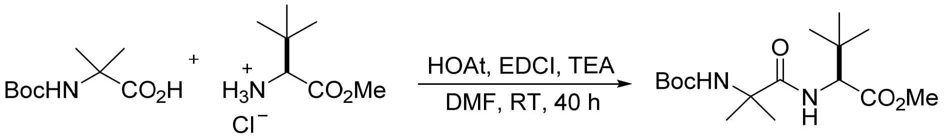

Boc-AIB-OH[10] (2.032 g, 10 mmol, 1 eq), H-(*L*)-^{t}Leu-OMe chlorohydrate[12] (1.815 g, 10 mmol, 1 eq), EDCI (2.876 g, 15 mmol, 1.5 eq) and HOAt (1.565 g, 11.5 mmol, 1.15 eq) were dissolved in DMF (20 mL) and the mixture was stirred at RT for 20 min. Then, TEA (1.2 eq) was added dropwise and the resulting yellow suspension was stirred at RT for 16 h. The reaction was quenched by the addition of 1M HCl (30 mL) and extracted with EtOAc (3 x 20 mL). The organic layers were combined, washed with brine (5 x 30 mL) and dried over $MgSO_4$. The solvent was evaporated under reduced pressure and the crude was purified by flash column chromatography on silica gel (Hexane:EtOAc 50:50) to afford the product as a white solid (1.831 g, 5.54 mmol, 55% yield). ^{1}H NMR (300 MHz, $CDCl_3$) δ 7.10 (s, 1H), 4.87 (s, 1H), 4.40 (d, J = 9.2 Hz, 1H), 3.70 (s, 3H), 1.52 (s, 3H), 1.45 (m, 12H), 0.98 (s, 9H).

[11] Synthesis procedure from: Yu, S.; Pan, X.; Ma, D. *Chem. Eur. J.* **2006**, *12*, 6572-6584.
[12] Synthesis procedure from: Anantharaj, S.; Jayakannan, M. *Biomacromolecules* **2012**, *13*, 2446-2455.

Step 2: Carboxylic acid deprotection

GENERAL PROCEDURE 1:[13]

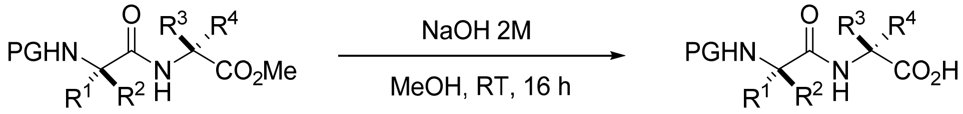

To a stirred suspension of the corresponding methyl ester protected dipeptide (1 eq) in MeOH (5 mL/mmol), 2M NaOH (3.2 eq) was added and the resulting suspension was stirred at RT overnight. Then, MeOH was evaporated under reduced pressure and the mixture was cooled down to 0 °C, acidified with 3M HCl to pH=2 and extracted with EtOAc (x3). The organic layers were combined, dried over $MgSO_4$ and evaporated under reduced pressure. The crude was used in the next step without further purification.

Boc-(*L*)-Val-(*L*)^{t}Leu-OH

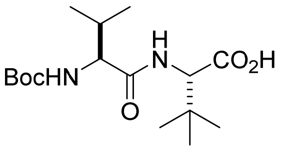

The title compound was prepared from methyl Boc-(*L*)-Val-(*L*)^{t}Leu-OMe (601.8 mg, 1.8 mmol, 1 eq) following the General Procedure 1. White foam, 595 mg, 1.8 mmol, quantitative yield. ^{1}H NMR (300 MHz, CD_3OD) δ 4.33 (m, 1H), 3.93 (d, *J* = 7.4 Hz, 1H), 2.05 (m, 1H), 1.47 (s, 9H), 1.05 (s, 9H), 0.97 (dd, *J* = 6.5, 4.0 Hz, 6H).

Boc-AIB-(*L*)^{t}Leu-OH

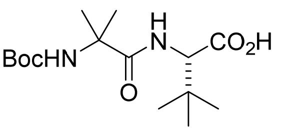

The title compound was prepared from methyl Boc-AIB-(*L*)^{t}Leu-OMe (1.652 g, 5 mmol, 1 eq) following the General Procedure 1. Yellow oil, 1.609 g, 5 mmol, quantitative yield. ^{1}H NMR (300 MHz, $CDCl_3$) δ 4.92 (s, 1H), 4.39 (d, *J* = 8.8 Hz, 1H), 1.52 (s, 3H), 1.48 (s, 3H), 1.44 (s, 9H), 1.04 (s, 9H).

PROCEDURE 2:[14] **Boc-(*D*)-Val-*(L)*^{t}Leu-OH**

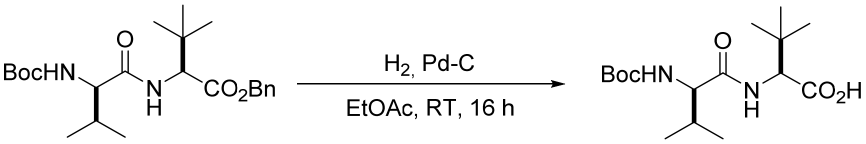

To a stirred solution of Boc-(*D*)-Val-*(L)*^{t}Leu-OBn (1.367 g, 3.25 mmol, 1 eq) in EtOAc (16 mL) under inert atmosphere, Pd-C (10% w/w) was added. The atmosphere was changed to H_2 and the resulting suspension was stirred at RT overnight. Then, the resulting suspension was filtered through Celite and the solvents were evaporated under reduced pressure. The crude was used in the next step without further purification. White foam, 1.047 g, 3.16 mmol, 97% yield. ^{1}H NMR (300 MHz, $CDCl_3$) δ 6.88 (s, 1H), 5.35 (s, 1H), 4.54 – 4.38 (m, 2H), 2.10 (s, 1H), 1.43 (s, 9H), 1.06 – 0.78 (m, 15H).

[13] Adapted from: Hata, R.; Nonaka H.; Takakusagi, Y.; Ichikawa, K.; Sando, S. *Angew. Chem. Int. Ed*. **2016**, *55*, 1765-1768.
[14] Adapted from : Kaplan, J. M.; Shang, J.; Gobbo, P.; Antonello, S.; Armelao, L.; Chatare, V.; Ratner, D. M.; Andrade, R. B.; Maran, F. *Langmuir* **2013**, *29*, 8187-8192.

Step 3: Curtius rearrangement

PROCEDURE 1:[15] **Catalyst C8**

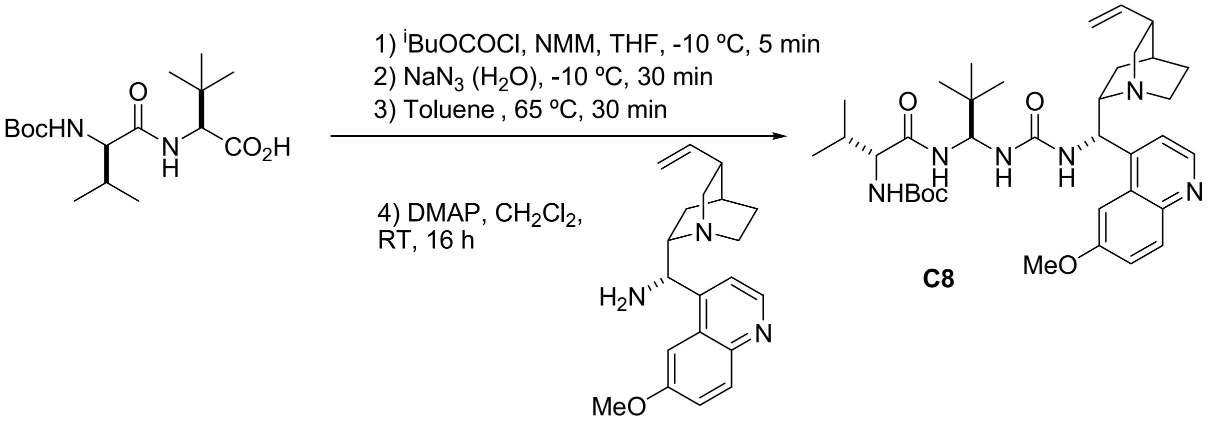

To a stirred solution of Boc-(*D*)-Val-(*L*)^{t}Leu-OH (1.047 g, 3.16 mmol, 1 eq) in dry THF (12.6 mL) under inert atmosphere and at -10 °C, isobutyl chloroformate (0.41 mL, 3.16 mmol, 1 eq) and *N*-methylmorpholine (0.35 mL, 3.16 mmol, 1 eq) were added and the resulting suspension was stirred for 5 min at the same temperature. Then, a previously prepared solution of NaN_3 (316 mg, 4.74 mmol, 1.5 eq) in H_2O (3 mL) was added and the mixture was stirred for 30 min at -10 °C. The organic layer was separated, concentrated under reduced pressure and the resulting residue redissolved in CH_2Cl_2.The solution was washed with H_2O (x3), dried over $MgSO_4$ and evaporated. The resulting slurry was redissolved in toluene (15.8 mL) and stirred at 65 °C until the disappearance of the azide band in the IR spectrum (±2140 cm^{-1}). After reaction completion (30 min) the mixture was cooled to RT and toluene was evaporated under reduced pressure. The resulting mixture was redissolved in CH_2Cl_2 (16.2 mL) and 9-amino-(9-deoxy) epiquinine[16] (0.9 eq) was added. The reaction mixture was cooled down to 0 °C, DMAP (30 mol%) was added and the mixture was stirred at RT overnight. The solvent was evaporated under reduced pressure and the resulting crude was purified by flash column chromatography on silica gel (CH_2Cl_2:MeOH 98:2) to give the product as a white solid (1.296 g, 1.99 mmol, 63% yield). m.p.: 139-142 °C. $[\alpha]_D^{25}$= –18.83 (c=1.00, CH_2Cl_2) ^{1}H NMR (300 MHz, $CDCl_3$) δ 8.72 (d, *J* = 4.6 Hz, 1H), 7.99 (d, *J* = 9.2 Hz, 1H), 7.77 (s, 1H), 7.36 (dd, *J* = 9.2, 2.6 Hz, 1H), 7.31 (d, *J* = 4.4 Hz, 1H), 6.57 (s, 1H), 5.79 (ddd, *J* = 17.5, 10.3, 7.3 Hz, 1H), 5.45 (s, 1H), 5.19 (t, *J* = 9.2 Hz, 1H), 5.05 (d, *J* = 14.7 Hz, 2H), 4.94 (s, 1H), 3.97 (s, 3H), 3.94 – 3.82 (m, 1H), 3.45 – 3.20 (m, 2H), 2.98 – 2.66 (m, 2H), 2.48 – 2.32 (m, 1H), 2.11 – 1.99 (m, 1H), 1.76 – 1.49 (m, 4H), 1.39 (s, 9H), 1.29 – 1.21 (m, 1H), 0.95 – 0.83 (m, 12H), 0.80 (s, 1H), 0.75 – 0.59 (m, 3H). ^{13}C NMR (75 MHz, $CDCl_3$) δ 171.7, 157.8, 157.6, 155.7, 147.4, 145.4, 144.4, 140.4, 131.2, 128.3, 121.5, 118.9, 114.8, 102.0, 79.2, 64.2, 60.2, 59.1, 55.5, 53.4, 40.9, 38.8, 35.0, 31.5, 29.5, 28.2, 27.3, 27.2, 25.7, 25.2, 19.3, 17.0. HRMS (ESI-TOF) m/z: $[M+H]^+$ calcd for $C_{36}H_{55}N_6O_5$ 651.4234; found 651.4234.

---

[15] Adapted from: ref. 6.

[16] Synthesis procedure from: Sudermeier, U.; Döbler, C.; Mehltretter, G. M.; Baumann, W.; Beller, M. *Chirality* **2003**, *15*, 127-134.

GENERAL PROCEDURE 2:[6] Catalysts **C9** and **C10**

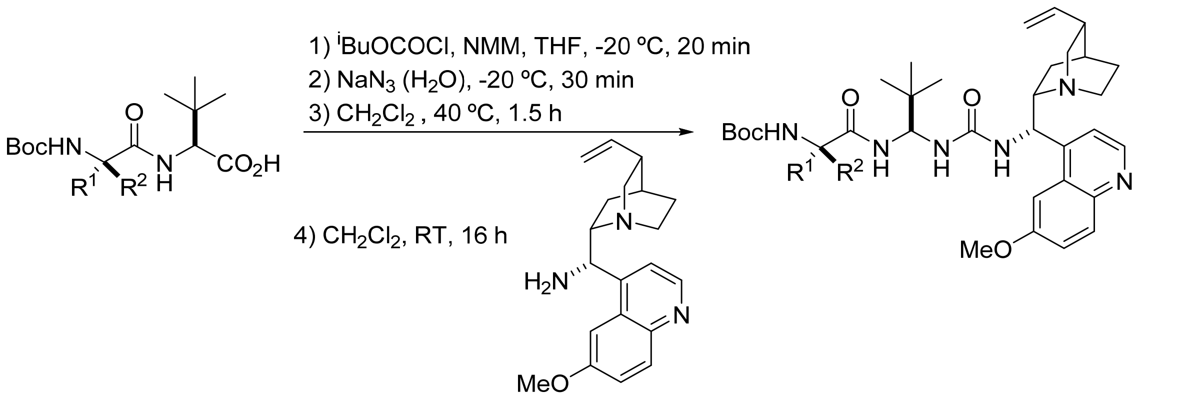

To a stirred solution of the corresponding Boc protected dipeptide (1 eq) in dry THF under inert atmosphere and at -20 °C, isobutyl chloroformate (1 eq) and *N*-methylmorpholine (1 eq) were added and the resulting suspension was stirred for 20 min at the same temperature. Then, a previously prepared solution of NaN_3 (1.5 eq) in H_2O (0.7 mL/mmol) was added and the mixture was stirred for 30 min at -20 °C. The organic layer was separated, concentrated under reduced pressure and the resulting residue redissolved in CH_2Cl_2 (5mL/mmol). The solution was washed with H_2O (x3), dried over $MgSO_4$ and evaporated. The resulting slurry was redissolved again in dry CH_2Cl_2 (3mL/mmol) and stirred at 40 °C until the disappearance of the azide band in the IR spectrum (±2140 cm^{-1}). After reaction completion the mixture was cooled to RT, 9-amino-(9-deoxy) epiquinine[16] (0.8 eq) was added and the mixture was stirred at RT overnight. The solvent was evaporated under reduced pressure and the resulting crude was purified by flash column chromatography on non-acidic silica gel.

Catalyst C9

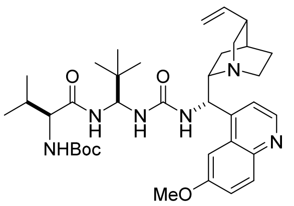

The desired product was obtained following the General Procedure 2 starting from the previously prepared Boc-(*L*)-Val-(*L*)-^{t}Leu-OH (660.4 mg, 2 mmol, 1 eq). The crude was purified by flash column chromatography on silica gel (CH_2Cl_2:MeOH 98:2) to give the product as a white solid (763.8 mg, 1.17 mmol, 66% yield). m.p.: 142-145 °C. $[\alpha]_D^{25}$ = –177.5 (c=1.00, CH_2Cl_2). ^1H NMR (300 MHz, CD_3OD) δ 8.68 (d, *J* = 4.7 Hz, 1H), 7.96 (d, *J* = 9.2 Hz, 1H), 7.81 (d, *J* = 2.6 Hz, 1H), 7.52 (d, *J* = 4.8 Hz, 1H), 7.45 (dd, *J* = 9.2, 2.6 Hz, 1H), 5.93 (ddd, *J* = 17.5, 10.3, 7.5 Hz, 1H), 5.62 (d, *J* = 10.5 Hz, 1H), 5.10 (t, *J* = 14.2 Hz, 2H), 4.02 (s, 3H), 3.79 (d, *J* = 7.8 Hz, 1H), 3.57 (s, 2H), 3.44 (dd, *J* = 13.4, 10.3 Hz, 1H), 2.96 (dd, *J* = 14.3, 4.7 Hz, 2H), 2.51 (d, *J* = 4.4 Hz, 1H), 2.09 – 1.90 (m, 1H), 1.87 – 1.57 (m, 4H), 1.45 (s, 9H), 0.98 – 0.83 (m, 15H). ^{13}C NMR (75 MHz, CD_3OD) δ 174.0, 159.9, 159.1, 148.2, 145.2, 141.7, 131.3, 130.0, 123.7, 120.7, 115.6, 103.3, 80.4, 64.9, 61.9, 61.0, 56.6, 56.4, 54.8, 42.4, 40.2, 36.5, 31.5, 28.7, 27.7, 27.0, 25.8, 20.0, 18.7. HRMS (ESI-TOF) m/z: $[M+H]^+$ calcd for $C_{36}H_{55}N_6O_5$ 651.4234; found 651.4234.

Catalyst C10

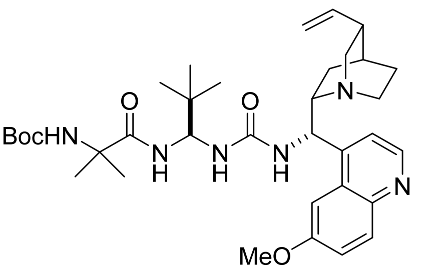

The desired product was obtained following the General Procedure 2 starting from the previously prepared Boc-AIB-(*L*)-^{t}Leu-OH (632.6 mg, 2 mmol, 1 eq). The crude was purified by flash column chromatography on silica gel (CH_2Cl_2:MeOH 98:2) to give the product as a white solid (412.3 mg, 0.65 mmol, 32% yield). m.p.: 131-134 °C. $[\alpha]_D^{25}$ = −15.7 (c=1.00, CH_2Cl_2). ^{1}H NMR (300 MHz, $CDCl_3$) δ 8.64 (d, *J* = 4.6 Hz, 1H), 7.93 (d, *J* = 9.2 Hz, 1H), 7.76 (d, *J* = 2.4 Hz, 1H), 7.31 (dd, *J* = 6.4, 3.4 Hz, 2H), 7.21 (s, 1H), 6.52 (s, 1H), 5.76 (ddd, *J* = 17.4, 10.3, 7.3 Hz, 1H), 5.48 (s, 1H), 5.26 (s, 1H), 5.12 (m, 1H), 4.97 (dd, *J* = 13.7, 7.2 Hz, 3H), 3.92 (s, 3H), 3.44 – 3.13 (m, 6H), 2.85 – 2.59 (m, 2H), 2.27 (s, 1H), 1.55 (m, 4H), 1.33 (d, *J* = 4.4 Hz, 12H), 1.05 (s, 3H), 0.80 (d, *J* = 21.9 Hz, 11H). ^{13}C NMR (75 MHz, $CDCl_3$) δ 175.7, 158.6, 158.1, 155.5, 148.0, 146.2, 145.2, 142.0, 132.0, 129.3, 122.5, 119.8, 115.1, 102.8, 80.9, 65.0, 60.2, 57.4, 56.4, 41.6, 39.9, 35.9, 28.8, 28.3, 28.1, 27.3, 26.2, 26.0, 25.5. HRMS (ESI-TOF) m/z: $[M+H]^+$ calcd for $C_{35}H_{53}N_6O_5$ 637.4077; found 637.4097.

2.2. Dipeptide derived catalyst C11

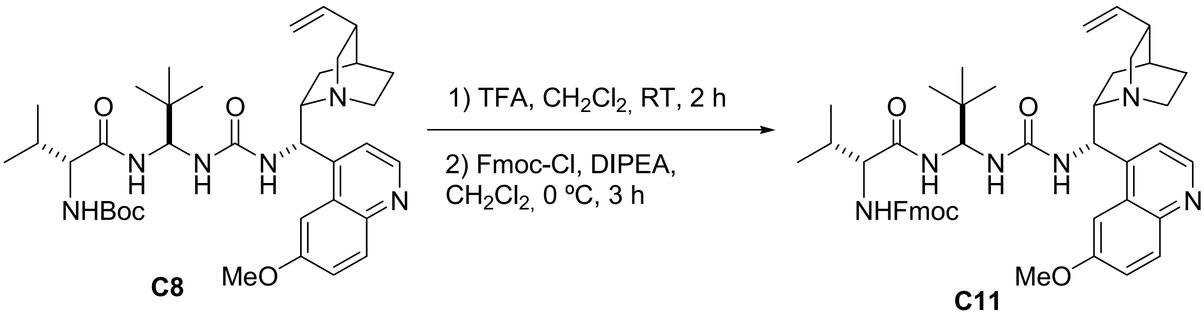

<u>*Step 1: Amine deprotection*</u>[17]

To a stirred solution of the starting *N*-Boc compound (150 mg, 0.23 mmol, 1 eq) in CH_2Cl_2 (0.23 mL, 1 mL/mmol) at 0 °C, TFA (0.46 mL, 2 mL/mmol) was added dropwise. The mixture was allowed to reach RT and then stirred for 2 h. Then, solvents were removed under reduced pressure and the remaining oil was redissolved in H_2O, cooled down to 0 °C and basified with a sat. solution of Na_2CO_3. The formed solid was extracted with EtOAc (x3) and the organic layer was washed with a sat. solution of $NaHCO_3$. and dried over $MgSO_4$. The solvents were removed under reduced pressure to afford the crude product as a yellow oil (117 mg, 0.21 mmol, 91% yield), which was used in the next step without further purification. ^{1}H NMR (300 MHz, $CDCl_3$) δ 8.60 (t, *J* = 4.5 Hz, 1H), 7.90 (d, *J* = 9.2 Hz, 1H), 7.77 (d, *J* = 8.7 Hz, 1H), 7.72 (d, *J* = 2.7 Hz, 1H), 7.40 – 7.19 (m, 2H), 7.04 – 6.90 (m, 1H), 5.69 (ddd, *J* = 17.5, 10.2, 7.3 Hz, 1H), 5.46 (s, 1H), 5.11 (t, *J* = 9.0 Hz, 1H), 5.02 – 4.89 (m, 2H), 3.88 (s, 3H), 3.36 (s, 2H), 3.20 (dd, *J* = 13.8, 10.1 Hz, 1H), 3.09 (d, *J* = 4.2 Hz, 1H), 2.91 – 2.59 (m, 4H), 2.34 – 2.21 (m, 1H), 1.65 – 1.39 (m, 4H), 0.89 – 0.69 (m, 16H), 0.46 (s, 2H).

[17] Adapted from: Müller, J.; Feifel, S. C.; Schmiederer, T.; Zocher, R.; Süssmuth, R. D. *ChemBioChem* **2009**, *10* (2), 323-328.

Step 2: Amine protection as Fmoc carbamate[18]

To a stirred solution of the free amine (117 mg, 0.21 mmol, 1 eq) in CH_2Cl_2 (1.8 mL, 8.4 mL/mmol), DIPEA (0.08 mL, 0.46 mmol, 2.2 eq) was added and the mixture was cooled down to 0 °C. A solution of Fmoc-Cl (64.5 mg, 0.25 mmol, 1.2 eq) in CH_2Cl_2 (0.48mL, 2.3 mL/mmol) was added then and the resulting mixture was stirred at the same temperature for 3 h. The reaction was quenched with a saturated solution of $NaHCO_3$ (0.85 mL) and the organic phase was separated, washed with $NaHCO_3$ sat (x3) and brine and dried over $MgSO_4$. The solvents were evaporated under reduced pressure and the crude was purified by flash column chromatography on non-acidic silica gel (CH_2Cl_2:MeOH 99:1) to give the product as a white solid (92.8 mg, 0.12 mmol, 58% yield). m.p.: 103-107 °C. $[\alpha]_D^{24}$= −7.74 (c=1.00, CH_2Cl_2). ^1H NMR (300 MHz, $CDCl_3$) δ 8.66 (d, *J* = 4.3 Hz, 1H), 7.98 (d, *J* = 9.2 Hz, 1H), 7.77 – 7.68 (m, 3H), 7.54 (t, *J* = 7.0 Hz, 2H), 7.43 – 7.21 (m, 6H), 6.86 (s, 1H), 6.54 (s, 1H), 5.72 (ddd, *J* = 17.4, 10.1, 7.5 Hz, 1H), 5.63 – 5.53 (m, 1H), 5.43 – 5.32 (m, 1H), 5.21 (t, *J* = 8.6 Hz, 1H), 5.06 – 4.88 (m, 2H), 4.40 (t, *J* = 8.8 Hz, 1H), 4.27 – 4.13 (m, 2H), 4.06 – 3.99 (m, 1H), 3.90 (s, 3H), 3.29 – 3.15 (m, 2H), 2.85 – 2.62 (m, 2H), 2.34 – 2.22 (m, 1H), 2.04 – 1.92 (m, 1H), 1.67 – 1.60 (m, 1H), 1.58 – 1.38 (m, 3H), 1.30 – 1.23 (m, 3H), 0.90 – 0.81 (m, 6H), 0.77 (s, 9H). ^{13}C NMR (75 MHz, $CDCl_3$) δ 171.6, 158.0, 157.6, 156.6, 147.7, 144.9, 144.0, 143.8, 141.4, 141.1, 131.7, 128.6, 127.9, 127.2, 125.2, 121.8, 120.1, 120.1, 114.9, 102.3, 77.4, 67.2, 64.7, 60.5, 60.2, 55.8, 47.3, 41.1, 39.3, 35.3, 31.4, 29.8, 27.5, 26.2, 25.5, 19.4, 17.5. HRMS (ESI-TOF) m/z: $[M+H]^+$ calcd for $C_{46}H_{57}N_6O_5$ 773.4390; found 773.4412.

2.3. Tripeptide derived catalysts C12-C13

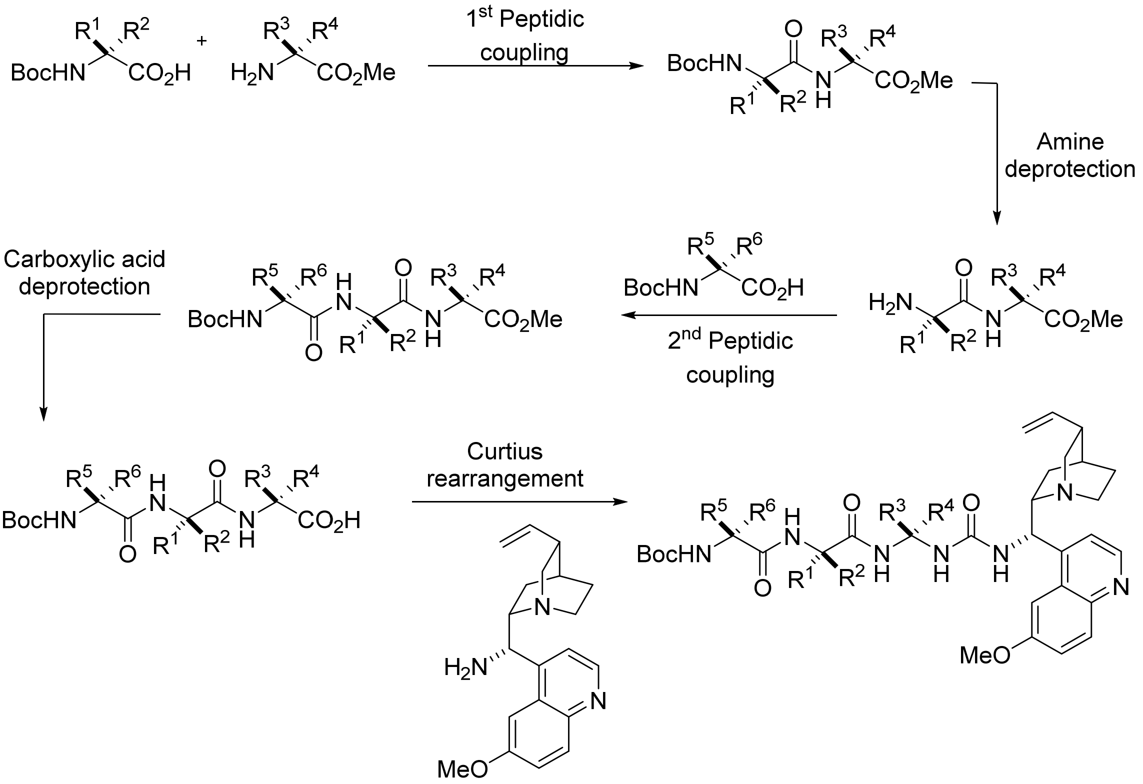

[18] Granger, B. A.; Brown, D. G. *Bioorg. Med. Chem. Lett.* **2016**, *26*, 5304-5307.

Step 1: 1st Peptidic coupling

The Procedures 2 and 3 described on page S6 were followed.

Step 2: Amine deprotection

GENERAL PROCEDURE:[17]

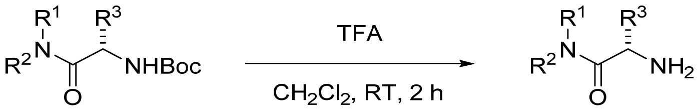

To a stirred solution of the starting *N*-Boc compound (1 eq) in CH_2Cl_2 (1 mL/mmol) at 0 °C TFA (2 mL/mmol) was added dropwise. The mixture was allowed to reach RT and then stirred for 2 h. Then, solvents were removed under reduced pressure and the remaining oil was redissolved in H_2O, cooled down to 0 °C and basified with a sat. solution of Na_2CO_3. The formed solid was extracted with EtOAc (x3) and the organic layer was washed with a sat. solution of $NaHCO_3$ and dried over $MgSO_4$. The solvents were removed under reduced pressure to afford the crude product, which was used in the next step without further purification.

H-(*L*)-Val-(*L*)^{t}Leu-OMe

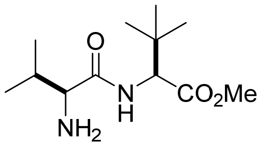

The title product was prepared starting from previously prepared Boc-(*L*)-Val-(*L*)^{t}Leu-OMe (688.5 mg, 2 mmol, 1 eq) following the General Procedure. Yellow oil, (437.5 mg, 1.9 mmol, 95% yield). ^{1}H-NMR (300 MHz, $CDCl_3$) δ 7.93 (d, *J* = 9.4 Hz, 1H), 4.41 (d, *J* = 9.5 Hz, 1H), 3.64 (s, 3H), 2.39 – 2.25 (m, 1H), 0.99 (s, 9H), 0.86 (d, *J* = 6.9 Hz, 6H). All spectroscopic data were consistent with those previously reported.[19]

H-AIB-(*L*)^{t}Leu-OMe

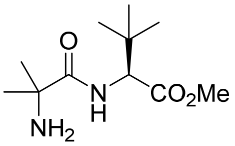

The title product was prepared starting from previously prepared Boc-AIB-(*L*)^{t}Leu-OMe (1.953 mg, 5.9 mmol, 1 eq) following the General Procedure. Yellow oil, (1.299 g, 5.6 mmol, 96% yield). ^{1}H NMR (300 MHz, $CDCl_3$) δ 8.21 (s, 1H), 4.35 (d, *J* = 9.6 Hz, 1H), 3.71 (s, 3H), 1.64 (s, 2H), 1.37 (s, 6H), 0.98 (s, 9H).

[19] Victor, F.; Lamar, J.; Snyder, N.; Yip, Y.; Guo, D.; Yumibe, N.; Johnson, R. B.; Wang, Q. M.; Glass, J. I.; Chen, S. *Bioorg. Med. Chem. Lett.* **2004,** *14*, 257-261.

Step 3: 2nd Peptidic coupling

PROCEDURE 1:[20] **Boc-(*L*)-Pro-(*L*)-Val-(*L*)^{t}Leu-OMe**

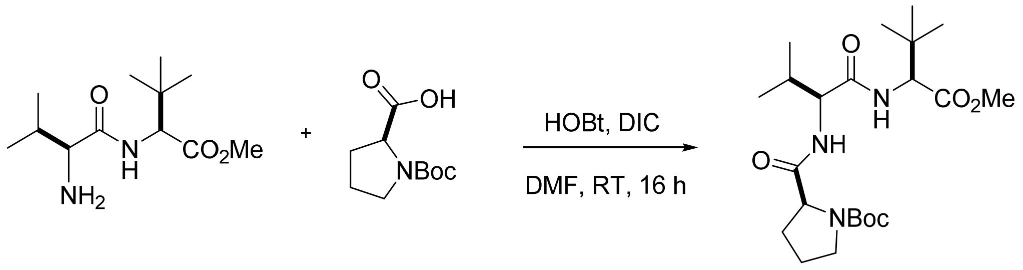

To a solution of H-(*L*)-Val-(*L*)^{t}Leu-OMe (1.046 g, 1.9 mmol, 1 eq) in DMF (6 mL), *N*-Boc-*L*-proline (450 mg, 2.09 mmol, 1.1 eq) and HOBt (0.3 g, 2.5 mmol, 1.28 eq) were added and the mixture was stirred for 20 min at RT. The reaction mixture was then cooled down to 0 °C and DIC (0.4 mL, 2.66 mmol, 1.4 eq) was added. The resulting mixture was stirred at RT for 16 h and then diluted with EtOAc and washed with H_2O (3x5 mL), brine (5x5 mL) and $NaHCO_3$ (3x5 mL). The organic layers were combined and dried over $MgSO_4$. The solvent was evaporated under reduced pressure and the crude was purified by silica flash column chromatography (CH_2Cl_2: MeOH 85:15). The desired product was obtained as a white solid (750.7 mg, 1.7 mmol, 89% yield). ^{1}H NMR (400 MHz, $CDCl_3$) δ 8.71 (d, *J* = 8.4 Hz, 1H), 8.42 (d, *J* = 8.7 Hz, 1H), 5.30 (s, 1H), 4.42 (d, *J* = 9.2 Hz, 1H), 4.22 (m, 2H), 3.81 (m, 1H), 3.71 (s, 3H), 2.57 – 2.47 (m, 1H), 2.29 (m, 4H), 1.55 (s, 9H), 1.46 (s, 6H), 0.97 (s, 9H).

PROCEDURE 2: **Boc-AIB-AIB-(*L*)^{t}Leu-OMe**

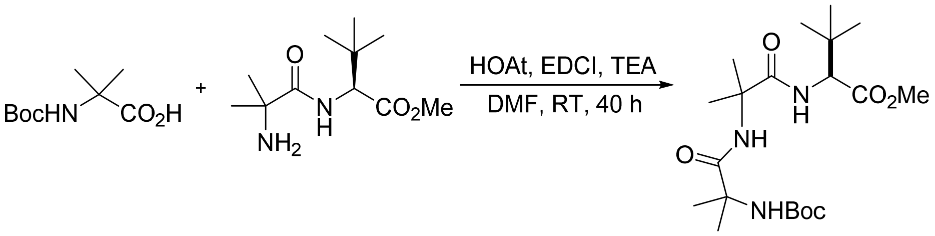

Boc-AIB-OH[10] (1.138 g, 5.6 mmol, 1 eq), H-AIB-(*L*)-^{t}Leu-OMe (1.299 g, 5.6 mmol, 1 eq), EDCI (1.610 g, 8.4 mmol, 1.5 eq) and HOAt (876.4 g, 6.44 mmol, 1.15 eq) were dissolved in DMF (11 mL) and the mixture was stirred at RT for 20 min. Then, TEA (0.92 mL, 6.72 mmol, 1.2 eq) was added dropwise and the resulting yellow suspension was stirred at RT for 16 h. The reaction was quenched by the addition of 1M HCl (15 mL) and extracted with EtOAc (3 x 10 mL). The organic layers were combined, washed with brine (5 x 15 mL) and dried over $MgSO_4$. The solvent was evaporated under reduced pressure and the crude was purified by flash column chromatography on silica gel (Hexane:EtOAc 50:50) to afford the product as a white solid (1.7736 g, 4.3 mmol, 76% yield). ^{1}H NMR (300 MHz, $CDCl_3$) δ 7.34 (d, *J* = 8.7 Hz, 1H), 6.76 (s, 1H), 4.83 (s, 1H), 4.42 (d, *J* = 8.9 Hz, 1H), 3.69 (s, 3H), 1.55 (s, 3H), 1.47 (m, 18H), 1.01 (s, 9H).

[20] Revelou, P.; Kokotos, C. G.; Moutevelis-Minakakis, P. *Tetrahedron* **2012**, *68*, 8732-8738.

Step 4: Carboxylic acid deprotection

GENERAL PROCEDURE:[13]

The General Procedure on page S7 was followed.

Boc-(*L*)-Pro-(*L*)-Val-(*L*)^{t}Leu-OH

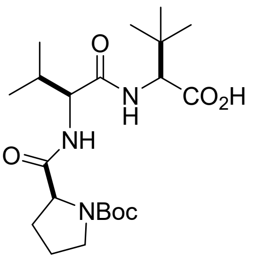

The title compound was prepared from Boc-(*L*)-Pro-(*L*)-Val-(*L*)^{t}Leu-OMe (750.7 mg, 1.7 mmol, 1 eq) following the General Procedure. White solid, (513 mg, 1.2 mmol, 70% yield). ^{1}H NMR (400 MHz, $CDCl_3$) δ 7.47 (s, 1H), 5.30 (s, 1H), 4.45 (d, *J* = 9.1 Hz, 1H), 4.31 (m, 2H), 3.82 – 3.68 (m, 1H), 2.21 (m, 1H), 1.89 (m, 4H), 1.44 (s, 9H), 1.01 (s, 9H), 0.90 (m, 6H).

Boc-AIB-AIB-(*L*)^{t}Leu-OH

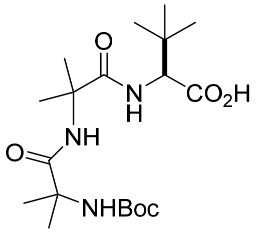

The title compound was prepared from Boc-AIB-AIB-(*L*)^{t}Leu-OMe following the General Procedure. White solid (1.7263 g, 4.3 mmol, quantitative yield). ^{1}H NMR (300 MHz, $CDCl_3$) δ 7.45 (d, *J* = 8.0 Hz, 2H), 6.73 (s, 2H), 4.94 (s, 2H), 4.32 (d, *J* = 8.0 Hz, 2H), 1.50 (d, *J* = 2.8 Hz, 14H), 1.48 – 1.42 (m, 36H), 1.10 (s, 21H).

Step 5: Curtius rearrangement

The General Procedure 1 reported on page S8 was followed.

Catalyst C12

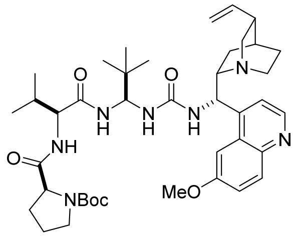

The desired product was obtained following the General Procedure 1 starting from the previously prepared Boc-(*L*)-Pro-(*L*)-Val-(*L*)-^{t}Leu-OH (427.5 mg, 1 mmol, 1 eq). The crude was purified by flash column chromatography on silica gel (CH_2Cl_2:MeOH 95:5) to give the product as a white solid (299 mg, 0.4 mmol, 40% yield). m.p.: 130-133 °C. $[\alpha]_D^{25}$ = –181.3 (c=1.00, CH_2Cl_2). ^{1}H NMR (300 MHz, $CDCl_3$) δ 8.72 (d, *J* = 4.5 Hz, 1H), 7.98 (d, *J* = 9.1 Hz, 1H), 7.77 (s, 1H), 7.34 (d, *J* = 8.2 Hz, 2H), 6.71 (d, *J* = 31.4 Hz, 1H), 6.51 (d, *J* = 45.8 Hz, 1H), 5.77 (dt, *J* = 17.4, 9.0 Hz, 1H), 5.29 (s, 1H), 5.16 (d, *J* = 17.8 Hz, 1H), 5.03 – 4.88 (m, 2H), 4.34 – 4.19 (m, 1H), 4.14 – 4.00 (m, 1H), 3.96 (s, 3H), 3.38 (s, 2H), 3.22 (d, *J* = 10.0 Hz, 3H), 2.73 (dt, *J* = 13.2, 8.2 Hz, 2H), 2.33 – 2.19 (m, 5H), 1.86 (s, 3H), 1.60 (d, *J* = 20.8 Hz, 5H), 1.44 (s, 9H), 1.25 (t, *J* = 7.0 Hz, 1H), 0.87 (s, 9H), 0.77 (s, 6H). ^{13}C NMR (75 MHz, $CDCl_3$) δ 173.7, 171.7, 158.0, 157.6, 146.9, 144.2, 140.4, 136.4, 131.0, 128.4, 127.6, 121.9, 118.4, 117.1, 100.9, 80.0, 64.9, 59.8, 59.1, 55.4, 53.2, 46.8, 41.8, 36.3, 33.7, 28.5, 27.9, 27.7, 27.6, 26.3, 24.9, 24.7, 23.9, 23.8, 18.9, 16.9. HRMS (ESI-TOF) m/z: $[M+H]^+$ calcd for $C_{41}H_{62}N_7O_6$ 748.4762; found 748.4772.

Catalyst C13

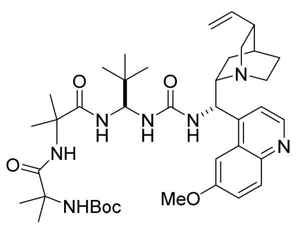

The desired product was obtained following the General Procedure 1 starting from the previously prepared Boc-AIB-AIB-(*L*)-^{t}Leu-OH (803 mg, 2 mmol, 1 eq). The crude was purified by flash column chromatography on silica gel (Hexanes:EtOAc 50:50) to give the product as a white solid (157.2 mg, 0.22 mmol, 11% yield). m.p.: 112-116 °C. $[\alpha]_D^{25}$ = –7.5 (c=0.20, CH_2Cl_2). ^{1}H NMR (300 MHz, $CDCl_3$) δ 8.69 (d, *J* = 4.6 Hz, 1H), 7.95 (d, *J* = 9.2 Hz, 1H), 7.84 (d, *J* = 2.4 Hz, 1H), 7.54 (d, *J* = 9.1 Hz, 1H), 7.42 – 7.29 (m, 2H), 6.28 (d, *J* = 16.1 Hz, 2H), 5.81 (ddd, *J* = 17.4, 10.3, 7.4 Hz, 1H), 5.47 (s, 1H), 5.20 (t, *J* = 9.1 Hz, 1H), 4.99 (dd, *J* = 13.7, 9.3 Hz, 2H), 4.81 (d, *J* = 13.9 Hz, 2H), 3.97 (s, 3H), 3.25 (dd, *J* = 13.7, 10.1 Hz, 3H), 2.95 – 2.61 (m, 2H), 2.28 (d, *J* = 12.9 Hz, 3H), 1.60 (d, *J* = 24.7 Hz, 4H), 1.38 (dt, *J* = 15.2, 6.2 Hz, 21H), 0.95 (s, 10H). ^{13}C NMR (75 MHz, $CDCl_3$) δ 175.2, 173.7, 158.5, 158.1, 148.2, 146.6, 145.4, 142.5, 132.0, 129.5, 122.4, 120.0, 115.0, 103.0, 81.8, 65.4, 60.6, 57.8, 57.6, 56.8, 56.4, 41.5, 40.4, 36.0, 28.8, 28.7, 28.3, 27.5, 27.0, 26.8, 26.1, 25.1. HRMS (ESI-TOF) m/z: $[M+H]^+$ calcd for $C_{39}H_{60}N_7O_6$ 722.4605; found 722.4601.

2.4. Preparation of catalyst C15

Step 1: Amine protection

Fmoc-(*L*)-^{t}Leu-OH[21]

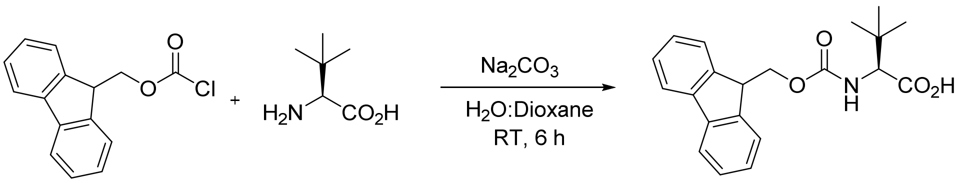

To a mixture of *L-tert*-Leucine (262.3 mg, 2 mmol, 1 eq) in H_2O (1.5 mL/mmol) and dioxane (1.5 mL/mmol), Na_2CO_3 10% aqueous solution (3 eq) was added dropwise at 0 °C and the mixture was stirred at this temperature for 10 min. Then, (9*H*-fluoren-9-yl)methyl chloroformate was added, and the resulting mixture was stirred at RT for 6 h, then poured over H_2O (10 mL/mmol) and washed with Et_2O (x2). The aqueous layer was cooled to 0 °C, acidified with 3M HCl and extracted with EtOAc (x3). The organic layers were combined, dried over $MgSO_4$ and evaporated under reduced pressure. The resulting crude was used in the next step without further purification. White solid (653.8 mg, 1.85 mmol, 92% yield). ^{1}H NMR (400 MHz, CD_3OD) δ 7.78 (d, J = 7.5 Hz, 2H), 7.68 (t, J = 6.7 Hz, 2H), 7.39 (t, J = 7.5 Hz, 2H), 7.30 (dt, J = 7.5, 1.0 Hz, 2H), 4.39-4.33 (m, 2H), 4.23 (t, J = 6.9 Hz, 1H), 4.05 (s_b, 1H), 3.66 (s, 1H), 1.03 (s, 9H); ^{13}C NMR (100 MHz, CD_3OD) δ 175.0, 159.1, 145.7, 145.6, 143.0, 129.2, 128.6, 128.5, 126.7, 121.3, 68.5, 68.4, 64.5, 35.3, 27.6. All spectroscopic data were consistent with those previously described.

---

[21] Pan, S.C.; Zhou, J.; List, B. *Angew. Chem. Int. Ed.* **2007**, *46*, 612-614.

Step 2: Curtius rearrangement[6]

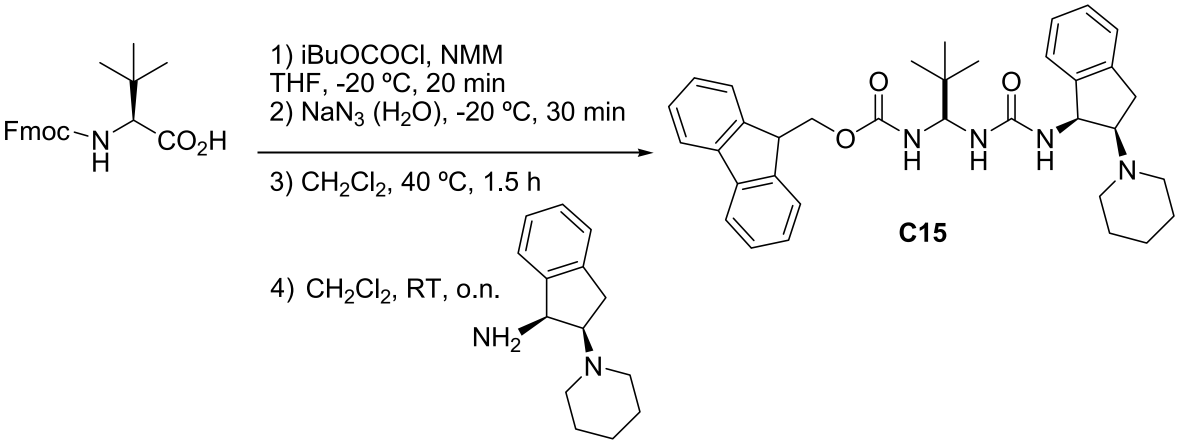

To a stirred solution of the *N*-Fmoc amino acid (0.636 mg, 1.8 mmol) in dry THF (8 mL) at -20 °C isobutyl chloroformate (0.23 mL, 1.8 mmol, 1 eq) and *N*-methylmorpholine (0.20 mL, 1.8 mmol, 1 eq) were added and the resulting suspension was stirred for 20 min at the same temperature. Then, a previously prepared solution of NaN_3 (2.7 mmol, 1.5 eq) in H_2O (2 mL) was added and the mixture was stirred for 30 min at -20 °C. The mixture was allowed to reach RT, the organic layer was separated, concentrated under reduced pressure and the resulting residue redissolved in CH_2Cl_2 (5mL/mmol). The solution was washed with H_2O (x3), dried over $MgSO_4$ and evaporated. The resulting slurry was redissolved again in dry CH_2Cl_2 (3mL/mmol) and stirred at 40 °C until the disappearance of the azide band in the IR spectrum (±2140 cm^{-1}). After reaction completion the mixture was cooled to RT, (1*S*,2*R*)-2,3-dihydro-2-(piperidin-1-yl)-1H-inden-1-amine[22] (0.8 eq) was added and the mixture was stirred at RT overnight. The solvent was evaporated under reduced pressure and the resulting crude was purified by flash column chromatography on non-acidic silica gel. White solid; yield: 714 mg, 1.26 mmol, 70%. m.p.: 166-167 °C. $[\alpha]_D^{25}$= +12.9 (c = 0.8, CH_2Cl_2). ^1H NMR (400 MHz, DMSO-d_6, 70 °C), δ: 7.88 (d, *J* = 7.5 Hz, 2H), 7.73 (d, *J* = 7.4 Hz, 2H), 7.42 (t, *J* = 7.3 Hz, 2H), 7.33 (m, 3H), 7.15 (m, 4H), 6.25 (m, 1H), 6.19 (d, *J* = 9.1 Hz, 1H), 5.19 (t, *J* = 9.0 Hz, 1H), 5.04 (t, *J* = 6.7 Hz, 1H), 4.31 (m, 1H), 4.23 (m, 2H), 2.93 (m, 2H), 2.84 (m, 1H), 2.45 (m, 2H), 2.36 (m, 2H), 1.51 (m, 4H), 1.37 (m, 2H), 0.86 (s, 9H). ^{13}C NMR (100 MHz, DMSO-d_6, 70 °C), δ: 157.4, 155.9, 145.2, 144.4, 144.3, 141.2, 140.8, 128.1, 127.9, 127.5, 127.0, 125.8, 125.3, 124.8, 120.6, 68.3, 65.8, 65.5, 54.4, 52.6, 47.2, 36.3, 34.6, 25.9, 25.8, 24.5. HRMS (ESI-TOF) m/z: $[M+H]^+$ calcd for $C_{35}H_{43}N_4O_3$ 567.3335; found 567.3337.

[22] Ren, Q.; Siau, W.-Y.; Du, Z.; Zhang, K.; Wang, J. *Chem. Eur. J.* **2011**, *17*, 7781-7785.

3. Preparation of starting materials

3.1. SYNTHESIS OF NITROALKANES 1A-1M and 1O

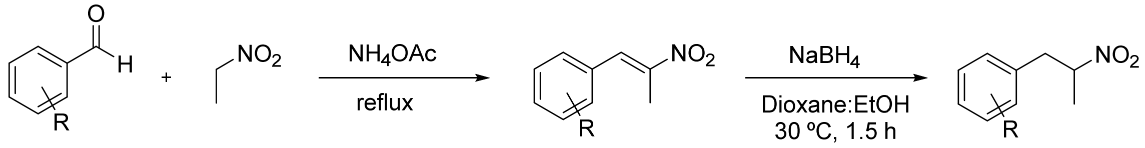

GENERAL PROCEDURE:[23]

To a stirred mixture of the corresponding aldehyde (20 mmol, 1 eq) and nitroethane (60 mL, 3mL/mmol), ammonium acetate (1.54 g, 20 mmol, 1 eq) was added and the reaction mixture was refluxed for 2 h. Nitroethane excess was then evaporated and the resulting mixture was redissolved in EtOAc, washed with H_2O (x2) and brine (x3) and dried over $MgSO_4$. Solvents were removed under reduced pressure to afford the crude nitroalkene, which was then dissolved in dioxane (15 mL) and slowly added (during 45 min) to a mixture of $NaBH_4$ (1.63 g, 43 mmol, 2.15 eq) in EtOH/dioxane (20 mL, 1:3 proportion) at 30 °C. The reaction mixture was stirred at 30 °C for another 45 minutes and then quenched with an ice/water mixture (40 mL) followed by acetic acid 50% (10 mL). After the gas evolution stopped, the aqueous phase was extracted with CH_2Cl_2 (x3). The organic layers were combined, washed with H_2O (x3) and brine, and dried over $MgSO_4$. Solvents were removed under reduced pressure and the crude was purified by flash column chromatography on silica gel (Hexane:EtOAc 98:2 to 95:5).

(2-Nitropropyl)benzene (1A)

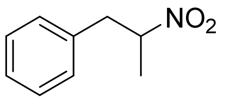

The title compound was prepared following the General Procedure starting from benzaldehyde (2.12 g, 20 mmol). The product was obtained as a colorless oil (1.65 g, 10 mmol, 50% yield). ^{1}H NMR (400 MHz, $CDCl_3$) δ 7.35 – 7.24 (m, 3H), 7.19 – 7.14 (m, 2H), 4.78 (m, 1H), 3.33 (dd, *J* = 14.0, 7.4 Hz, 1H), 3.01 (dd, *J* = 14.0, 6.9 Hz, 1H), 1.55 (d, *J* = 6.7 Hz, 3H). ^{13}C NMR (100 MHz, $CDCl_3$) δ 135.6, 129.0, 128.8, 127.4, 84.5, 41.2, 18.8. All spectroscopic data were consistent with those previously reported.[24]

1-Methyl-4-(2-nitropropyl)benzene (1B)

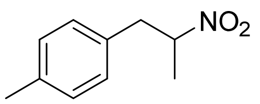

The title compound was prepared following the General Procedure starting from *p*-tolualdehyde (2.40 g, 20 mmol). The product was obtained as a yellowish oil (2.93 g, 16.4 mmol, 82% yield). ^{1}H NMR (400 MHz, $CDCl_3$) δ 7.12 (d, *J* = 7.9 Hz, 2H), 7.05 (d, *J* = 7.9 Hz, 2H), 4.75 (m, 1H), 3.28 (dd, *J* = 14.0, 7.4 Hz, 1H), 2.97 (dd, *J* = 14.0, 6.9 Hz, 1H), 2.32 (s, 3H), 1.54 (d, *J* = 6.6 Hz, 3H). ^{13}C NMR (100 MHz, $CDCl_3$) δ 137.1, 132.4, 129.5, 128.9, 84.6, 40.8, 21.1, 18.8. All spectroscopic data were consistent with those previously reported.[24]

---

[23] For the Henry reaction, see: a) Liu, G.; Liu, X.; Cai, Z.; Jiao, G.; Xu, G.; Tang, W.; Liu, G.; Liu, X.; Cai, Z.; Jiao, G.; Xu, G.; Tang, W. *Angew. Chem. Int. Ed.* **2013**, *52*, 4235-4238. For the reduction, see: b) Bhattacharjya, A.; Mukhopadhyay, R.; Pakrashi, S. C. *Synthesis* **1985**, 886-887.

[24] Li, S.; Huang, K.; Zhang, X. *Chem. Commun.* **2014**, *50*, 8878-8881.

1-Chloro-4-(2-nitropropyl)benzene (1C)

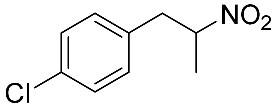

The title compound was prepared following the General Procedure starting from *p*-chlorobenzaldehyde (2.81 g, 20 mmol). The product was obtained as a colorless oil (3.51 g, 17.6 mmol, 88% yield). ^{1}H NMR (400 MHz, $CDCl_3$) δ 7.30 (d, *J* = 8.4 Hz, 2H), 7.12 (d, *J* = 8.4 Hz, 2H), 4.78 (m, 1H), 3.28 (dd, *J* = 14.2, 7.9 Hz, 1H), 3.01 (dd, *J* = 14.2, 6.3 Hz, 1H), 1.56 (d, *J* = 6.7 Hz, 3H). ^{13}C NMR (100 MHz, $CDCl_3$) δ 134.3, 133.2, 130.4, 128.9, 84.3, 40.3, 18.8. All spectroscopic data were consistent with those previously reported.[24]

1-Nitro-4-(2-nitropropyl)benzene (1D)

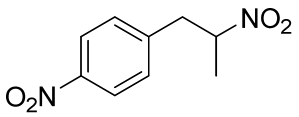

The title compound was prepared following the General Procedure starting from *p*-nitrobenzaldehyde (3.1 g, 20 mmol). The product was obtained as an orange solid (2.69 g, 12.8 mmol, 64% yield). m.p.: 49-51 °C. ^{1}H NMR (400 MHz, $CDCl_3$) δ 8.15 (d, *J* = 8.7 Hz, 2H), 7.35 (d, *J* = 8.7 Hz, 2H), 4.83 (m, 1H), 3.40 (dd, *J* = 14.3, 8.4 Hz, 1H), 3.14 (dd, *J* = 14.3, 5.8 Hz, 1H), 1.60 (d, *J* = 6.7 Hz, 3H). ^{13}C NMR (100 MHz, $CDCl_3$) δ 147.4, 143.0, 130.0, 124.0, 83.8, 40.6, 19.1. All spectroscopic data were consistent with those previously reported.[24]

1-Methoxy-4-(2-nitropropyl)benzene (1E)

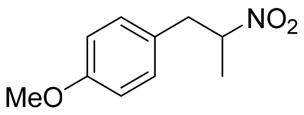

The title compound was prepared following the General Procedure starting from *p*-methoxybenzaldehyde (2.72 g, 20 mmol). The product was obtained as a yellow oil (3.24 g, 16.6 mmol, 83% yield). ^{1}H NMR (400 MHz, $CDCl_3$) δ 7.07 (d, *J* = 8.7 Hz, 2H), 6.84 (d, *J* = 8.7 Hz, 2H), 4.73 (m, 1H), 3.75 (s, 3H), 3.21 (dd, *J* = 14.0, 7.7 Hz, 1H), 2.95 (dd, *J* = 14.1, 6.5 Hz, 1H), 1.57 (d, *J* = 6.7 Hz, 3H). ^{13}C NMR (100 MHz, $CDCl_3$) δ 158.9, 130.1, 127.6, 114.2, 84.8, 55.1, 40.3, 18.6. All spectroscopic data were consistent with those previously reported.[24]

1-Methyl-2-(2-nitropropyl)benzene (1F)

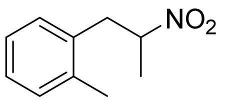

The title compound was prepared following the General Procedure starting from *o*-tolualdehyde (2.40 g, 20 mmol). The product was obtained as a colorless oil (2.35 g, 13.1 mmol, 66% yield). ^{1}H NMR (400 MHz, $CDCl_3$) δ 7.21 – 7.17 (m, 2H), 7.15 (dd, *J* = 8.2, 3.4 Hz, 1H), 7.10 (d, *J* = 6.6 Hz, 1H), 4.78 (m, 1H), 3.39 (dd, *J* = 14.1, 7.2 Hz, 1H), 3.02 (dd, *J* = 14.1, 7.3 Hz, 1H), 2.36 (s, 3H), 1.57 (d, *J* = 6.6 Hz, 3H). ^{13}C NMR (100 MHz, $CDCl_3$) δ 136.3, 133.9, 130.8, 129.8, 127.6, 126.4, 83.4, 38.6, 19.4, 18.9. All spectroscopic data were consistent with those previously reported.[25]

1-Methyl-3-(2-nitropropyl)benzene (1G)

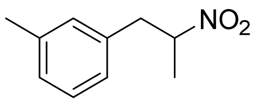

The title compound was prepared following the General Procedure starting from *m*-methylbenzaldehyde (2.40 g, 20 mmol). The product was obtained as a colorless oil (2.71 g, 15.1 mmol, 75% yield). ^{1}H NMR (400 MHz, $CDCl_3$) δ 7.22 (t, *J* = 7.5 Hz, 1H), 7.10 (d, *J* = 7.6 Hz, 1H), 7.02 – 6.94 (m, 2H), 4.79 (m, 1H), 3.30 (dd, *J* = 13.9, 7.4 Hz, 1H), 2.98 (dd, *J* = 13.9, 6.9 Hz, 1H), 2.35 (s, 3H), 1.55 (d, *J* = 6.7 Hz, 3H). ^{13}C NMR (100 MHz, $CDCl_3$) δ

[25] Kawai, Y.; Inaba, Y.; Tokitoh, N. *Tetrahedron Asymmetry* **2001**, *12*, 309-318.

138.5, 135.5, 129.8, 128.7, 128.2, 126.0, 84.5, 41.1, 21.4, 18.8. All spectroscopic data were consistent with those previously reported.[25]

1-Chloro-3-(2-nitropropyl)benzene (1H)

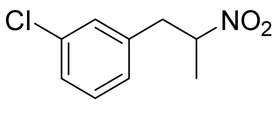

The title compound was prepared following the General Procedure starting from *m*-chlorobenzaldehyde (2.81 g, 20 mmol). The product was obtained as a colorless oil (3.65 g, 18.3 mmol, 91% yield). ^{1}H NMR (400 MHz, $CDCl_3$) δ 7.28 – 7.19 (m, 2H), 7.16 (s, 1H), 7.07 – 7.02 (m, 1H), 4.76 (m, 1H), 3.28 (dd, *J* = 14.1, 7.8 Hz, 1H), 2.98 (dd, *J* = 14.1, 6.4 Hz, 1H), 1.54 (d, *J* = 6.7 Hz, 3H). ^{13}C NMR (100 MHz, $CDCl_3$) δ 137.6, 134.6, 130.1, 129.1, 127.7, 127.2, 84.1, 40.6, 18.9. All spectroscopic data were consistent with those previously reported.[24]

1-Methoxy-3-(2-nitropropyl)benzene (1I)

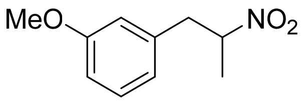

The title compound was prepared following the General Procedure starting from *m*-methoxybenzaldehyde (2.72 g, 20 mmol). The product was obtained as a yellow oil (3.16 g, 16.2 mmol, 81% yield). ^{1}H NMR (400 MHz, $CDCl_3$) δ 7.25 – 7.19 (m, 1H), 6.81(d, *J* = 7.5 Hz, 1H), 6.75 (d, *J* = 7.5 Hz, 1H), 6.70 (s, 1H), 4.78 (m, 1H), 3.79 (s, 3H), 3.31 (dd, *J* = 13.9, 7.4 Hz, 1H), 2.98 (dd, *J* = 13.9, 6.9 Hz, 1H), 1.54 (d, *J* = 6.7 Hz, 3H). ^{13}C NMR (100 MHz, $CDCl_3$) δ 159.9, 137.0, 129.8, 121.3, 114.8, 112.7, 84.3, 55.2, 41.2, 18.8. All spectroscopic data were consistent with those previously reported.[24]

1-(2-Nitropropyl)-3,5-bis(trifluoromethyl)benzene (1J)

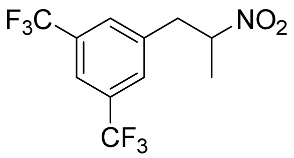

The title compound was prepared following the General Procedure starting from 3,5-bis(trifluoromethyl)benzaldehyde (4.84 g, 20 mmol). The product was obtained as a colorless oil (4.58 g, 15.2 mmol, 76% yield). ^{1}H NMR (400 MHz, $CDCl_3$) δ 7.81 (s, 1H), 7.65 (s, 2H), 4.84 (m, 1H), 3.45 (dd, *J* = 14.5, 8.4 Hz, 1H), 3.17 (dd, *J* = 14.6, 5.6 Hz, 1H), 1.62 (d, *J* = 6.7 Hz, 3H). ^{13}C NMR (100 MHz, $CDCl_3$) δ 138.1, 132.2 (q, *J* = 33.3 Hz), 129.2, 123.1 (q, *J* = 273.7 Hz), 121.3 (m), 83.6, 40.3, 19.0. All spectroscopic data were consistent with those previously reported.[24]

1-(2-Nitropropyl)naphthalene (1K)

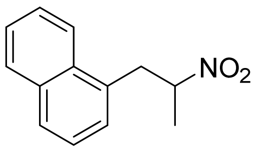

The title compound was prepared following the General Procedure starting from 1-naphthalenecarboxaldehyde (3.12 g, 20 mmol). The product was obtained as a yellow oil (2.88 g, 13.4 mmol, 67% yield). ^{1}H NMR (400 MHz, $CDCl_3$) δ 8.00 (d, *J* = 8.4 Hz, 1H), 7.89 (d, *J* = 8.0 Hz, 1H), 7.80 (d, *J* = 8.2 Hz, 1H), 7.58 (t, *J* = 7.0 Hz, 1H), 7.54 (t, *J* = 7.0 Hz, 1H), 7.41 (t, *J* = 7.6 Hz, 1H), 7.33 (d, *J* = 6.9 Hz, 1H), 4.98 (m, 1H), 3.88 (dd, *J* = 14.1, 6.7 Hz, 1H), 3.43 (dd, *J* = 14.1, 7.7 Hz, 1H), 1.58 (d, *J* = 6.6 Hz, 3H). ^{13}C NMR (100 MHz, $CDCl_3$) δ 133.5, 133.0, 132.6, 128.6, 128.0, 127.7, 127.7, 126.8, 126.4, 126.0, 84.4, 41.3, 18.9. HRMS (ESI-TOF) m/z: $[M+H]^+$ calcd for $C_{13}H_{14}NO_2$ 216.1025; found 216.1022.

2-(2-Nitropropyl)naphthalene (1L)

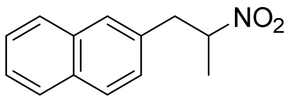

The title compound was prepared following the General Procedure starting from 2-naphthalenecarboxaldehyde (3.12 g, 20 mmol). The crude was purified by precipitation from hexanes to afford the product as an orange solid (2.93 g, 13.6 mmol, 68% yield). m.p.: 45-48 °C. ^{1}H NMR (400 MHz, $CDCl_3$) δ 7.83 – 7.79 (m, 3H), 7.63 (s, 1H), 7.48 (m, 2H), 7.29 (d, *J* = 8.3 Hz, 1H), 4.89 (m, 1H), 3.51 (dd, *J* = 14.0, 7.4 Hz, 1H), 3.18 (dd,

J = 14.0, 6.9 Hz, 1H), 1.59 (d, J = 6.6 Hz, 3H). ^{13}C NMR (100 MHz, $CDCl_3$) δ 133.5, 133.0, 132.6, 128.6, 128.0, 127.7, 127.7, 126.8, 126.4, 126.0, 84.4, 41.3, 18.9. All spectroscopic data were consistent with those previously reported.[24]

5-(2-Nitropropyl)benzo[*d*][1,3]dioxole (1M)

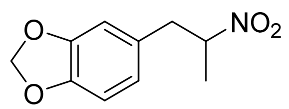

The title compound was prepared following the General Procedure starting from piperonal (3.00 g, 20 mmol). The product was obtained as a yellow oil (2.41 g, 11.5 mmol, 58% yield). ^{1}H NMR (400 MHz, $CDCl_3$) δ 6.73 (d, J = 7.9 Hz, 1H), 6.63 (s, 1H), 6.60 (d, J = 7.8 Hz, 1H), 5.93 (s, 2H), 4.71 (m, 1H), 3.21 (dd, J = 14.1, 7.6 Hz, 1H), 2.92 (dd, J = 14.1, 6.6 Hz, 1H), 1.53 (d, J = 6.6 Hz, 3H). ^{13}C NMR (100 MHz, $CDCl_3$) δ 147.9, 146.9, 129.1, 122.2, 109.2, 108.5, 101.1, 84.7, 40.9, 18.8. All spectroscopic data were consistent with those previously reported.[26]

Synthesis of 2-Nitrooctane (1O)[27]

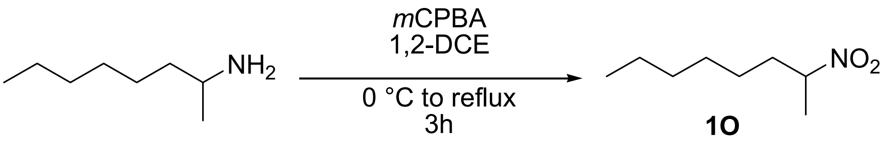

A solution of 2-aminooctane (0.52 g, 4 mmol) in 5 mL of 1,2-dichloroethane was added dropwise to a refluxing solution of *m*-chloroperbenzoic acid (2.1 g, 12 mmol, 3 eq) in 40 mL of 1,2-dichloroethane. Once the addition was finished, the mixture was stirred under reflux for 3 hours; then, the reaction mixture was cooled, filtered and washed with 3 X 10 mL of 1 N NaOH and 3 X 40 mL of brine, and the organic layer was dried over $MgSO_4$. Removal of the solvent under reduced pressure after filtration gave the crude which was purified through flash column chromatography on silica gel (Hexane:EtOAc 98:2 to 95:5) to obtain a colorless oil (0.45 g, 2.8 mmol, 70%). ^{1}H NMR (400 MHz, $CDCl_3$) δ 4.53 (m, 1H), 2.02 – 1.91 (m, 1H), 1.75 – 1.63 (m, 1H), 1.50 (d, J = 6.6 Hz, 3H), 1.27 (m, 8H), 0.86 (t, J = 6.2 Hz, 3H). ^{13}C NMR (100 MHz, $CDCl_3$) δ 83.6, 35.2, 31.4, 28.6, 25.6, 22.4, 19.2, 13.9. All spectroscopic data were consistent with those previously reported.[28]

3.2. SYNTHESIS OF α-HYDROXY ENONES 2a-2m

α-Hydroxy enones **2a**,[29] **2h**[29] and **2e**[30] were prepared following the procedure described in the literature.

[26] Pradhan, P. K.; Dey, S.; Jaisankar, P.; Giri, V. S. *Synth. Commun.* **2005**, *35*, 913-922.
[27] Gilbert, K.E.; Borden, W.T. *J. Org. Chem.* **1979**, *44*, 659-661.
[28] Durchschein, K.; Ferreira-da Silva, B.; Wallner, S.; Macheroux, P.; Kroutil, W.; Glueck, S. M.; Faber, K. *Green Chem.* **2010**, *12*, 616-619.
[29] Badiola, E.; Fiser, B.; Gómez-Bengoa, E.; Mielgo, A.; Olaizola, I.; Urruzuno, I.; García, J. M.; Odriozola, J. M.; Razkin, J.; Oiarbide, M.; Palomo, C. *J. Am. Chem. Soc.* **2014,** *136*, 17869-17881.
[30] Hoff, S.; Brandsma, L.; Arens, J. F. *Recl. Trav. Chim. Pays-Bas* **1968**, *87*, 1179-1184.

3.2.1 General procedure for the synthesis of α-hydroxy enones 2b, 2f, 2g[31]

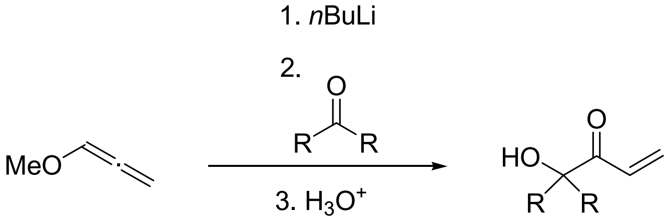

To a solution of methoxypropadiene (3.50 g, 50 mmol) in dry Et_2O (100 mL) at -40 °C, nBuLi (2.5 M in hexanes, 22 mL, 55 mmol) was added under nitrogen and the reaction mixture was stirred at -40 °C for 10 min. Then, a solution of the corresponding ketone (55 mmol) in dry Et_2O (55 mL) was added within 5 min. The mixture was stirred at the same temperature for 0.5 h and the reaction was quenched with H_2O (100 mL). The resulting mixture was allowed to warm to room temperature and extracted with Et_2O (3 x 100 mL). The combined organic extracts were dried over Na_2SO_4 and concentrated under reduced pressure to afford the corresponding addition adduct that was employed in the next step without further purification. The material from previous step was added dropwise to 5% aq H_2SO_4 (110 mL) at 0 °C and the mixture was stirred for 1.5 h. After this time the reaction mixture was allowed to warm to room temperature and was saturated with solid NaCl. Then it was extracted with Et_2O (5 x 60 mL) and the combined extracts were washed with brine and dried over Na_2SO_4. The solvent was removed under reduced pressure to afford the enone, which was purified through flash column chromatography (hexanes/EtOAc 90:10).

1-Hydroxy-1,1-diphenylbut-3-en-2-one (2b)

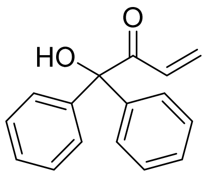

The product was obtained from commercially available benzophenone (10.02 g, 55 mmol) as a yellowish oil in 66% yield (7.86 g, 33.0 mmol). ^{1}H NMR (400 MHz, $CDCl_3$) δ 7.43 – 7.32 (m, 10H), 6.74 (dd, *J* = 17.0, 10.3 Hz, 1H), 6.53 (dd, *J* = 17.0, 1.8 Hz, 1H), 5.73 (dd, *J* = 10.3, 1.8 Hz, 1H), 5.06 (s, 1H). ^{13}C NMR (100 MHz, $CDCl_3$) δ 198.3, 141.1, 131.0, 130.6, 128.5, 128.3, 128.3, 84.6. HRMS (ESI-TOF) m/z: $[M+H]^+$ calcd for $C_{16}H_{15}O_2$ 239.1072; found 239.1076.

4-Hydroxy-4-propylhept-1-en-3-one (2f)

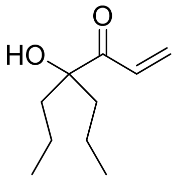

The product was obtained from commercially available heptan-4-one (6.28 g, 55 mmol) as a colorless liquid in 74% yield (6.30 g, 37.0 mmol). ^{1}H NMR (400 MHz, $CDCl_3$) δ 6.71 (dd, *J* = 17.0, 10.3 Hz, 1H), 6.51 (dd, *J* = 17.0, 1.8 Hz, 1H), 5.80 (dd, *J* = 10.3, 1.8 Hz, 1H), 3.92 (s, 1H), 1.77 – 1.59 (m, 4H), 1.42 (m, 2H), 0.98 (m, 2H), 0.86 (t, *J* = 7.2 Hz, 6H). ^{13}C-NMR (100 MHz, $CDCl_3$), δ (ppm) = 202.7, 130.7, 129.1, 80.9, 40.7, 16.5, 14.4. HRMS (ESI-TOF) m/z: $[M+H]^+$ calcd for $C_{10}H_{19}O_2$ 171.1385; found 171.1379.

[31] Odriozola, J. M.; Razkin, J.; Lorea, B.; Mielgo, A.; García, J. M.; Oiarbide, M.; Palomo, C. *Org. Biomol. Chem.* **2023**, *21*, 4833-4845.

4-Hydroxy-4-isobutyl-6-methylhept-1-en-3-one (2g)

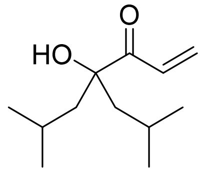

The product was obtained from commercially available 2,6-dimethylheptan-4-one (7.82 g, 55 mmol) as a colorless liquid in 78% yield (7.73 g, 39.0 mmol). ^{1}H NMR (400 MHz, $CDCl_3$) δ 6.73 (dd, *J* = 17.0, 10.3 Hz, 1H), 6.51 (dd, *J* = 17.0, 1.8 Hz, 1H), 5.79 (dd, *J* = 10.3, 1.8 Hz, 1H), 3.96 (s, 1H), 1.69 – 1.55 (m, 6H), 0.90 (d, *J* = 6.4 Hz, 6H), 0.75 (d, *J* = 6.3 Hz, 6H). ^{13}C NMR (100 MHz, $CDCl_3$) δ 203.4, 130.2, 129.8, 81.4, 47.9, 24.3, 24.1, 23.9. HRMS (ESI-TOF) m/z: $[M+H]^+$ calcd for $C_{12}H_{23}O_2$ 199.1698; found 199.1692.

3.2.2 General procedure for the synthesis of 1,1-bis(4-fluorophenyl)-1-hydroxybut-3-en-2-one 2i[32]

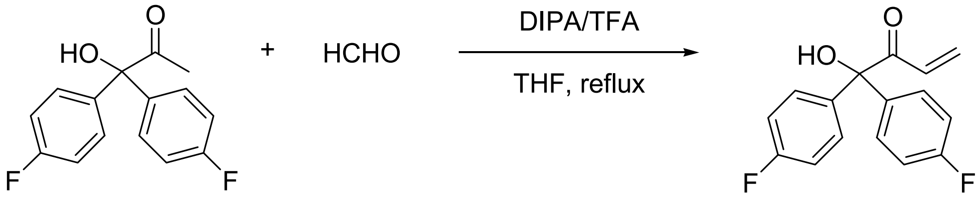

2i

1,1-Bis(4-fluorophenyl)-1-hydroxypropan-2-one[33] (6.56, 25 mmol) and paraformaldehyde (1.5 g, 50 mmol, 2 eq) were added to a previously prepared solution of DIPA (7 mL, 50 mmol, 2 eq) and TFA (4.8 mL, 62.5 mmol, 2.5 eq) in dry THF (125 mL). The mixture was stirred at reflux for 2 h and more paraformadehyde (1.5 g, 50 mmol, 2 eq) was added portion wise every 2 h aprox. during 6 h. Then, the mixture was stirred for 16 h. The reaction mixture was diluted with CH_2Cl_2 (50 mL) and washed with 1M HCl (2 x 40 mL), 1M NaOH (2 x 40 mL) and brine (2 x 40 mL). The organic layer was dried over $MgSO_4$ and the solvent was evaporated under reduced pressure. The crude was purified by flash column chromatography on silica gel using Et_2O as eluent. Colorless liquid, 54% yield (3.70 g, 13.5 mmol). ^{1}H NMR (400 MHz, $CDCl_3$) δ 7.37 – 7.29 (m, 4H), 7.11 – 7.02 (m, 4H), 6.67 (dd, *J* = 17.0, 10.1 Hz, 1H), 6.55 (dd, *J* = 17.0, 1.9 Hz, 1H), 5.78 (dd, *J* = 10.1, 1.9 Hz, 1H), 4.99 (s, 1H). ^{13}C NMR (100 MHz, $CDCl_3$) δ 197.7, 162.6 (d, *J* = 248.5 Hz), 136.8 (d, *J* = 4.0 Hz), 131.4, 130.5, 130.0 (d, *J* = 8.3 Hz), 115.5 (d, *J* = 21.8 Hz), 83.6. ^{19}F (471 MHz) δ –113.4. HRMS (ESI-TOF) m/z: $[M+H]^+$ calcd for $C_{16}H_{13}F_2O_2$ 275.0884; found 275.0881.

[32] Adapted from: Bugarin, A.; Jones, K. D.; Connell, B. T. *Chem. Commun.* **2010**, *46*, 1715-1717.
[33] Zhang, D.; Zou, J.; Chen, W.; Yiu, S.-M.; Tse, M.-K.; Luo, J.; Jen, A. K.-Y. *Chem. Mater.* **2022**, *34*, 3683-3693.

3.2.3 General procedure for the synthesis of α'-hydroxy enones 2c, 2d, 2j-2l and 2m

Step 1:[34]

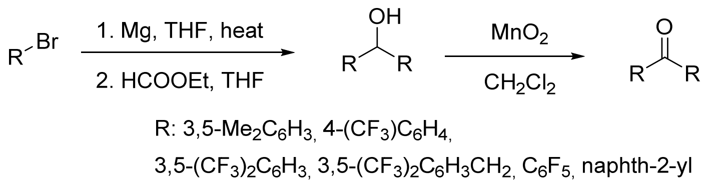

To a flame-dried three-necked round-bottomed flask filled with nitrogen and equipped with a refluxing condenser, the corresponding bromoaryl compound (43 mmol), magnesium (2.6 g, 107.5 mmol), THF (86 mL) and a few crystals of I_2 were added. The mixture was slowly heated to reflux and kept at reflux for 4 h. The resulting light brown solution was allowed to cool down to room temperature and then to 0 °C. Then ethyl formate (1.48 g, 1.62 mL, 20 mmol) was slowly added to the freshly prepared Grignard reagent and the mixture was stirred at room temperature for 16 h. The reaction was quenched at 0 °C by addition of a saturated aqueous solution of NH_4Cl. The resulting slurry was filtered through a Celite pad. The organic phase was separated. The aqueous layer was extracted with Et_2O (x3), and the combined organic layers were washed with brine, dried with $MgSO_4$, filtered and removed in vacuo to give solid products which could be used in the next step without further purification.

The obtained alcohols were dissolved in CH_2Cl_2 (300 mL) and activated MnO_2 (16.9 g, 300 mmol, 15 eq) was added to the solution. The reaction mixture was heated to reflux for two hours then it was allowed to cool down and filtered through a celite pad. The solvent was removed under vacuum to give coloured solids, which could be used in the next step without further purification.

Step 2:[35]

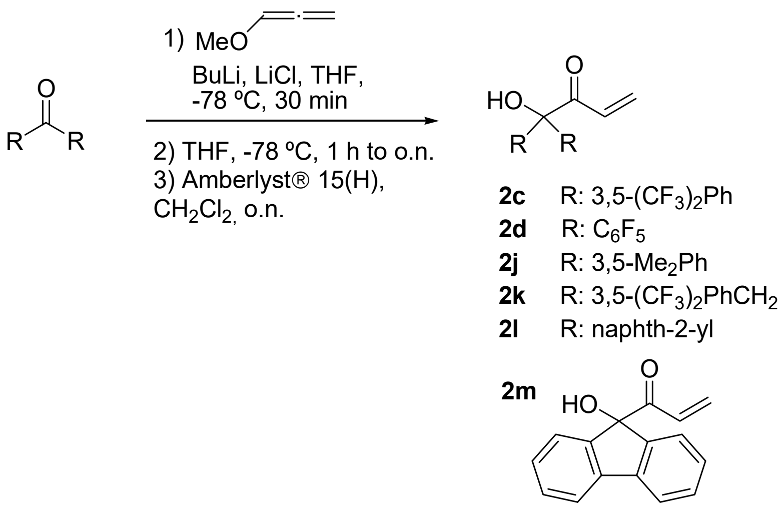

To a stirred solution of the previously prepared 1-methoxypropa-1,2-diene (783 mg, 11.2 mmol, 2.2 eq) and dried LiCl (1.94 g, 46 mmol) in dry THF (46 mL) under N_2 atmosphere and at -78 °C, n-BuLi (2.5M, 4.5 mL, 11.2 mmol, 2.2 eq) was added dropwise. The mixture was stirred for 30 min at the same

[34] Adapted from: Ren, H.; Wulff, W.D. *J. Am. Chem. Soc.* **2011**, *133*, 5656-5659.
[35] Adapted from: a) Harrington, P.E.; Tius, M.A. *Org. Lett.* **2000**, *16*, 2447-2450. b) Adapted from: ref. 29.

temperature and then a solution of the previously prepared ketone (5 mmol, 1 eq) in dry THF (46 mL) was added for 5 min. Finally, the mixture was stirred for 2 h at the same temperature, slowly quenched with H_2O (25 mL) and allowed to reach RT. The organic layer was separated and washed with water. The aqueous layer was extracted with Et_2O (x3). The organic layers were combined and dried over $MgSO_4$. The solvents were removed under reduced pressure to afford the allene as a brown oil. This allene was dissolved in dichloromethane (50 mL) and Amberlyst® 15 (2.5 g) was added at room temperature under stirring. The mixture was stirred at the same temperature overnight and then the resin was filtered. The solvent was removed under reduced pressure to afford the crude product, which was purified by flash column chromatography on silica gel (hexane:EtOAc 98:2 to 95:5).

1,1-Bis(3,5-bis(trifluoromethyl)phenyl)-1-hydroxybut-3-en-2-one (2c)

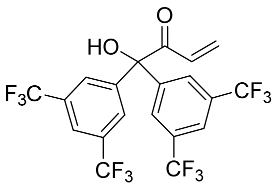

The title compound was prepared following the second step of the General Procedure starting from bis[3,5-bis(trifluoromethyl)phenyl]methanone (2.27 g, 5 mmol). The product was obtained as a yellowish oil (1.53 g, 3 mmol, 60% yield). ^{1}H NMR (400 MHz, $CDCl_3$) δ 7.95 (s, 2H), 7.80 (s, 4H), 6.77 – 6.58 (m, 2H), 6.00 (dd, *J* = 9.8, 1.6 Hz, 1H), 5.16 (s, 1H). ^{13}C NMR (100 MHz, $CDCl_3$) δ 195.1, 142.5, 134.8, 132.5 (q, *J* = 33.3 Hz), 129.0, 128.0, 123.1 (m), 122.9 (q, *J* = 273.0 Hz), 83.3. ^{19}F (471 MHz) δ –63.0. HRMS (ESI-TOF) m/z: $[M+H]^+$ calcd for $C_{20}H_{11}F_{12}O_2$ 511.0567; found 511.0564.

1-Hydroxy-1,1-bis(perfluorophenyl)but-3-en-2-one (2d)

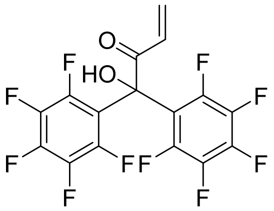

The title compound was prepared following the second step of the General Procedure starting from commercially available decaflurobenzophenone (1.81 g, 5 mmol). The product was obtained as a yellow oil (1.21 g, 2.9 mmol, 58% yield). ^{1}H NMR (400 MHz, $CDCl_3$) δ 6.71 (dd, *J* = 16.9, 1.7 Hz, 1H), 6.61 (dd, *J* = 16.9, 10.0 Hz, 1H), 5.94 (dd, *J* = 10.0, 1.6 Hz, 1H), 5.37 (s, 1H). ^{13}C NMR (100 MHz, $CDCl_3$) δ 190.3, 145.0 (dm), 141.6 (dm), 137.9 (dm), 133.7, 128.3, 113.6 (m), 77.5. ^{19}F (471 MHz) δ –160.6 (tm), –151.7 (tm), –137.7 (dm). HRMS (ESI-TOF) m/z: $[M+H]^+$ calcd for $C_{16}H_5F_{10}O_2$ 419.0130; found 419.0132.

1,1-Bis(3,5-dimethylphenyl)-1-hydroxybut-3-en-2-one (2j)

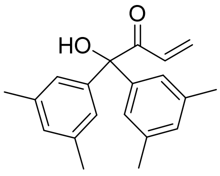

The title compound was prepared following the second step of the General Procedure starting from bis(3,5-dimethylphenyl)methanone (1.19 g, 5 mmol). The product was obtained as a colorless oil (0.85 g, 2.9 mmol, 58% yield). ^{1}H NMR (400 MHz, $CDCl_3$) δ 6.97 (s, 6H), 6.74 (dd, *J* = 17.1, 10.3 Hz, 1H), 6.51 (dd, *J* = 17.1, 1.8 Hz, 1H), 5.71 (dd, *J* = 10.3, 1.8 Hz, 1H), 4.95 (s, 1H), 2.29 (s, 12H). ^{13}C NMR (100 MHz, $CDCl_3$) δ 198.5, 141.0, 138.0, 131.2, 130.1, 129.9, 126.0, 84.6, 21.4. HRMS (ESI-TOF) m/z: $[M+H]^+$ calcd for $C_{20}H_{23}O_2$ 295.1698; found 295.1695.

4-(3,5-Bis(trifluoromethyl)benzyl)-5-(3,5-bis(trifluoromethyl)phenyl)-4-hydroxypent-1-en-3-one (2k)

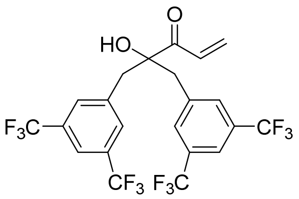

The title compound was prepared following the second step of the General Procedure starting from 1,3-bis[3,5-bis(trifluoromethyl)-phenyl]-2-propanone (2.41 g, 5 mmol) (THF needs to be substituted by Et_2O in the first step to avoid polimerization of the Grignard reagent). The product was obtained as a yellowish solid (1.61 g, 3 mmol, 60% yield). m.p.: 79-80 °C. ^1H NMR (400 MHz, $CDCl_3$) δ 7.75 (s, 2H), 7.65 (s, 4H), 7.03 (dd, *J* = 16.9, 10.4 Hz, 1H), 6.48 (dd, *J* = 16.9, 1.3 Hz, 1H), 6.03 (dd, *J* = 10.4, 1.3 Hz, 1H), 3.96 (s, 1H), 3.29 (d, *J* = 14.0 Hz, 2H), 3.22 (d, *J* = 14.0 Hz, 2H). ^{13}C NMR (100 MHz, $CDCl_3$) δ 198.5, 137.0, 133.7, 131.4 (q, *J* = 33.2 Hz), 130.4, 128.3, 123.2 (q, *J* = 272.8 Hz), 121.3 (m), 80.6, 43.4. ^{19}F (471 MHz) δ –63.0. HRMS (ESI-TOF) m/z: $[M+H]^+$ calcd for $C_{22}H_{15}F_{12}O_2$ 539.0880; found 539.0884.

1-Hydroxy-1,1-di(naphthalen-2-yl)but-3-en-2-one (2l)

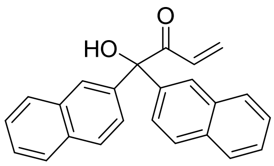

The title compound was prepared following the second step of the General Procedure starting from di-2-naphthalenylmethanone (1.41 g, 5 mmol). The product was obtained as a colorless oil (0.81 g, 2.4 mmol, 48% yield). ^1H NMR (400 MHz, $CDCl_3$) δ 7.92 – 7.83 (m, 6H), 7.83 – 7.77 (m, 2H), 7.60 – 7.44 (m, 6H), 6.85 (dd, *J* = 17.0, 10.3 Hz, 1H), 6.59 (dd, *J* = 17.0, 1.7 Hz, 1H), 5.75 (dd, *J* = 10.3, 1.7 Hz, 1H), 5.18 (s, 1H). ^{13}C NMR (100 MHz, $CDCl_3$) δ 198.2, 138.4, 133.0, 133.0, 131.1, 130.9, 128.5, 128.4, 127.6, 127.5, 126.7, 126.4, 126.0, 84.9. HRMS (ESI-TOF) m/z: $[M+H]^+$ calcd for $C_{24}H_{19}O_2$ 339.1385; found 339.1382.

1-(9-Hydroxy-9*H*-fluoren-9-yl)prop-2-en-1-one (2m)

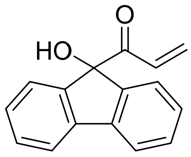

The title compound was prepared following the second step of the General Procedure starting from commercially available 9*H*-fluoren-9-one (0.90 g, 5 mmol). The product was obtained as a yellow oil (0.73 g, 3.1 mmol, 62% yield). ^1H NMR (400 MHz, $CDCl_3$) δ 7.75 (d, *J* = 7.6 Hz, 2H), 7.52 – 7.41 (m, 2H), 7.32 (m, 4H), 6.43 (dd, *J* = 17.0, 1.4 Hz, 1H), 5.66 (dd, *J* = 17.0, 10.4 Hz, 1H), 5.46 (dd, *J* = 10.4, 1.4 Hz, 1H), 5.20 (s, 1H). ^{13}C NMR (100 MHz, $CDCl_3$) δ 197.1, 143.7, 141.7, 130.8, 130.0, 128.9, 128.6, 124.5, 120.5, 87.3. HRMS (ESI-TOF) m/z: $[M+H]^+$ calcd for $C_{16}H_{13}O_2$ 237.0916; found 237.0913.

4. Catalytic conjugate additions

4.1. Catalyst screening for the model reaction between (2-nitropropyl)benzene (1A) and 4-hydroxy-4-methyl-1-penten-3-one (2a)

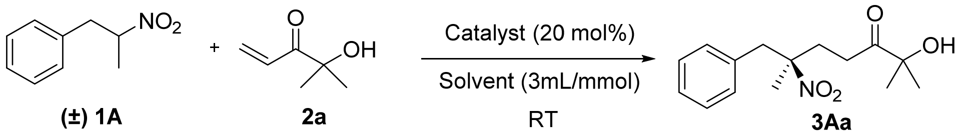

To a stirred solution of α-hydroxy enone **2a** (22.8 mg, 0.2 mmol, 1 eq) and nitroalkane **1A** (165 mg, 1 mmol, 5 eq) in $CHCl_3$, CH_2Cl_2 or toluene (0.6 mL) the corresponding catalyst (0.04 mmol, 20 mol%) was added and the mixture was stirred at room temperature. The reaction was followed by TLC (4:1 Hexane: EtOAc). After the indicated reaction time (see table below), the mixture was concentrated under reduced pressure. Reaction conversions were determined by ^{1}H NMR. Enantiomeric ratios were determined by HPLC using a chiral column.

The racemic addition adducts were obtained following the above procedure, using TEA (30 mol%) as catalyst.

Entry	Cat.	t(h)	Conv.[a] (%)	Yield[b] (%)	*e.r.*[c]	Entry	Cat.	t(h)	Conv.[a] (%)	Yield[b] (%)	*e.r.*[c]
1	**C1**	20	>99	nd	78:22	15	**C9**	43	>99	80	65:35
2	**C2**	23	>99	81	75:25	16	**C10**	16	>99	59	66:34
3	**C3**	26	>99	68	62:38	17	**C11**	25	>99	56	66:34
4	**C4**	48	>99	nd	65:35	18	**C12**	20	>99	73	63:37
5	**C5**	36	>99	nd	75:25	19	**C13**	26	>99	89	57:43
6	**C6**	20	>99	70	89:11	20	**C14**	30	>99	81	85:15
7[d]	**C6**	96	36	70	92:8	21	**C15**	336	>99	nd	68:32
8[e]	**C6**	14	34	nd	nd	22	**C16**	18	>99	nd	47:53
9	**C7**	14	>99	74	90:10	23	**C17**	23	>99	nd	46:54
10[d]	**C7**	96	55	nd	91:9	24	**C18**	72	>99	nd	60:40
11[e]	**C7**	14	71	nd	90:10	25	**C19**	216	95	nd	64:36
12[f]	**C7**	14	>99	72	90:10	26	**C20**	192	74	49	60:40
13[g]	**C7**	14	85	nd	90:10	27	**C21**	192	>99	77	59:41
14	**C8**	22	>99	76	72:28						

[a]Determined by ^{1}H NMR analysis on the crude product. [b]Yield of the isolated product by column chromatography. [c]Determined by chiral HPLC. [d]Reaction carried out at 0 °C. [e]Catalyst loading 10 mol%. [f] Reaction carried out in CH_2Cl_2. [g]Reaction carried out in toluene. nd: Not determined.

4.2. Acceptor screening

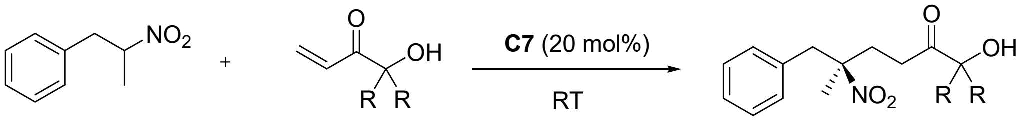

1A 2 3

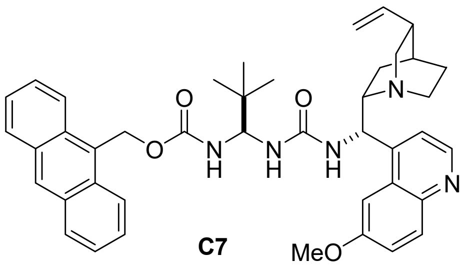

C7

3Aa R: Me
3Ab R: Ph
3Ac R: 3,5-$(CF_3)_2C_6H_3$
3Ad R: C_6F_5
3Ae R,R: -$(CH_2)_5$-
3Af R: ^{n}Pr
3Ag R: ^{i}Bu
3Ah R: Bn
3Ai R: 4-FC_6H_4
3Aj R: 3,5-$Me_2C_6H_3$
3Ak R: 3,5-$(CF_3)_2C_6H_3CH_2$
3Al R: Naphth-2-yl
3Am

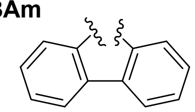

Entry	R,R	T (ºC)	t (h)	Yield (%)[a, b]	*e.r.*[c]
1	**a** Me	rt	14	74	90:10
2	Me	0	96	nd(55)	91:9
3	Me	0	96	nd(36)[d]	92:8
4	**b** Ph	rt	24	70	84:16
5	**c** Ar^F	rt	16	72	90:10
6	Ar^F	0	93	70	96:4
7	Ar^F	0	96	nd(65)[d]	95:5
8	**d** C_6F_5	rt	15	64	84:16
9	C_6F_5	0	93	67	91:9
10	**e** –$(CH_2)_5$-	rt	39	66	89:11
11	**f** *n*Pr	rt	46	82	86:14
12	**g** *i*Bu	rt	46	63	86:14
13	**h** Bn	rt	41	75	91:9
14	Bn	0	6d	nd (47)	92:8
15	**i** 4-FC_6H_4	rt	40	80	82:18
16	**j** 3,5-$Me_2C_6H_3$	rt	44	75	85:15
17	**k** Ar^FCH_2	rt	45	75	87:13
18	**l** Naphth-2-yl	rt	48	64	87:13
19	**m** 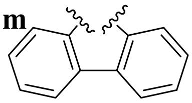	rt	64	80	87:13

[a]Yield for full conversion. [b]Conversion (%). [c]Determined by chiral HPLC. [d]Catalyst **C6** was used. nd: Not determined.

<u>GENERAL PROCEDURE:</u>

To a stirred solution of the corresponding α-hydroxy enone **2** (0.2 mmol, 1 eq) and (2-nitropropyl)benzene **(1A)** (165 mg, 1 mmol, 5 eq) in $CHCl_3$ (0.6 mL), catalyst **C7** (27.4 mg, 0.04 mmol, 20 mol%) was added and the mixture was stirred at room temperature. The reaction was followed by TLC (1:1 Hexane: EtOAc). After the indicated reaction time (see table), the mixture was concentrated

under reduced pressure and purified by flash column chromatography on silica gel (hexane:EtOAc 98:2 to 90:10). Enantiomeric ratios were determined by HPLC using a chiral column.

The racemic addition adducts were obtained following the above procedure, using TEA (30 mol%) as catalyst.

(*R*)-2-Hydroxy-2,6-dimethyl-6-nitro-7-phenylheptan-3-one (3Aa)

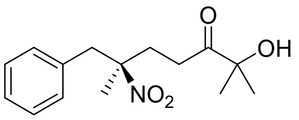

Prepared following the General Procedure using α-hydroxy enone **2a** (22.8 mg, 0.2 mmol). The product was isolated as a white solid (41.3 mg, 0.148 mmol, 74% yield). m.p.: 79-84 °C. The enantiomeric ratio (90:10) was determined by chiral HPLC analysis (IA, hexane/isopropanol = 95/5, flow rate = 0.5 mL/min, λ = 202 nm) t_R = 44.7 min (major), t_R = 49.4 min (minor). ^{1}H NMR (400 MHz, $CDCl_3$) δ 7.35 – 7.27 (m, 3H), 7.12 – 7.05 (m, 2H), 3.41 (s, 1H), 3.37 (d, *J* = 13.9 Hz, 1H), 3.08 (d, *J* = 13.9 Hz, 1H), 2.62 – 2.53 (m, 2H), 2.45 (ddd, *J* = 15.1, 9.1, 6.1 Hz, 1H), 2.21 – 2.06 (m, 1H), 1.48 (s, 3H), 1.36 (s, 6H). ^{13}C NMR (100 MHz, $CDCl_3$) δ 212.7, 134.2, 130.1, 128.6, 127.7, 91.1, 76.5, 46.5, 33.0, 30.3, 26.7, 26.6, 21.4. HRMS (ESI-TOF) m/z: $[M-H]^-$ calcd for $C_{15}H_{20}NO_4$ 278.1392; found 278.1389.

(*R*)-1-Hydroxy-5-methyl-5-nitro-1,1,6-triphenylhexan-2-one (3Ab)

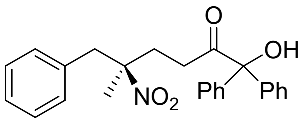

Prepared following the General Procedure using α-hydroxy enone **2b** (47.6 mg, 0.2 mmol). The product was isolated as a colorless oil (56.5 mg, 0.140 mmol, 70% yield). The enantiomeric ratio (84:16) was determined by chiral HPLC analysis (IA, hexane/isopropanol = 90/10, flow rate = 0.5 mL/min, λ = 202 nm) t_R = 31.3 min (major), t_R = 34.1 min (minor). ^{1}H NMR (400 MHz, $CDCl_3$) δ 7.42 – 7.29 (m, 7H), 7.28 – 7.23 (m, 6H), 7.02 – 6.95 (m, 2H), 4.39 (s, 1H), 3.20 (d, *J* = 13.9 Hz, 1H), 2.95 (d, *J* = 13.9 Hz, 1H), 2.61 (m, 1H), 2.37 – 2.25 (m, 2H), 1.93 (ddd, *J* = 14.8, 9.2, 6.8 Hz, 1H), 1.32 (s, 3H). ^{13}C NMR (100 MHz, $CDCl_3$) δ 209.6, 141.0, 134.1, 130.0, 128.7, 128.6, 128.4, 127.9, 127.6, 90.9, 85.9, 46.2, 33.7, 33.1, 21.3. HRMS (ESI-TOF) m/z: $[M-H]^-$ calcd for $C_{25}H_{24}NO_4$ 402.1705; found 402.1702.

(*R*)-1-Hydroxy-5-methyl-5-nitro-1,1-bis(perfluorophenyl)-6-phenylhexan-2-one (3Ad)

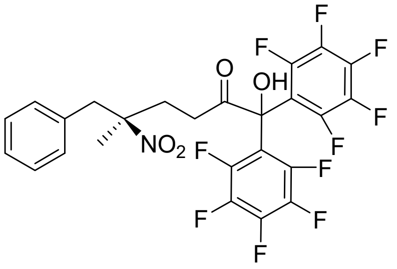

Prepared following the General Procedure using α-hydroxy enone **2d** (83.6 mg, 0.2 mmol). The product was isolated as a colorless oil (74.7 mg, 0.128 mmol, 64% yield). The enantiomeric ratio (84:16) was determined by chiral HPLC analysis (Phenomenex Lux 3 µm Cellulose 1, hexane/isopropanol = 95/5, flow rate = 1.0 mL/min, λ = 202 nm) t_R = 16.9 min (minor), t_R = 19.7 min (major). ^{1}H NMR (400 MHz, $CDCl_3$) δ 7.34 – 7.27 (m, 3H), 7.09 – 6.99 (m, 2H), 4.97 (s, 1H), 3.27 (d, *J* = 13.9 Hz, 1H), 3.09 (d, *J* = 13.9 Hz, 1H), 2.62 (m, 2H), 2.38 (m, 1H), 2.16 – 2.01 (m, 1H), 1.48 (s, 3H). ^{13}C NMR (100 MHz, $CDCl_3$) δ 201.1, 144.9 (dm), 141.7 (dm), 138.0 (dm), 133.8, 130.0, 128.6, 127.8, 113.8 (m), 90.5, 78.5, 46.6, 33.9, 32.0, 21.7. ^{19}F (471 MHz) δ –159.6 (dt, *J* = 56.5, 20.5 Hz), –150.7 (dt, *J* = 78.0, 23.1 Hz), –138.4 (dd, *J* = 118.7, 21.4 Hz). HRMS (ESI-TOF) m/z: $[M-H]^-$ calcd for $C_{25}H_{14}F_{10}NO_4$ 582.0763; found 582.0766.

(*R*)-1-(1-Hydroxycyclohexyl)-4-methyl-4-nitro-5-phenylpentan-1-one (3Ae)

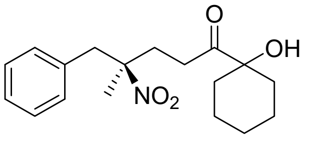

Prepared following the General Procedure using α-hydroxy enone **2e** (30.8 mg, 0.2 mmol). The product was isolated as a white solid (42.2 mg, 0.132 mmol, 66% yield). m.p.: 86-88 °C. The enantiomeric ratio (89:11) was determined by chiral HPLC analysis (Phenomenex Lux 3 μm Cellulose 1, hexane/isopropanol = 98/2, flow rate = 1.0 mL/min, λ = 202 nm) t_R = 36.2 min (minor), t_R = 38.5 min (major). ^{1}H NMR (400 MHz, $CDCl_3$) δ 7.33 – 7.27 (m, 3H), 7.11 – 7.06 (m, 2H), 3.36 (d, *J* = 13.8 Hz, 1H), 3.18 (s, 1H), 3.07 (d, *J* = 13.8 Hz, 1H), 2.66 – 2.52 (m, 2H), 2.43 – 2.41 (m, 1H), 2.17 – 2.05 (m, 1H), 1.77 – 1.55 (m, 8H), 1.55 – 1.45 (m, 2H), 1.47 (m, 3H). ^{13}C NMR (100 MHz, $CDCl_3$) δ 213.0, 134.3, 130.1, 128.6, 127.7, 91.2, 78.2, 46.5, 34.0, 33.1, 30.6, 25.1, 21.4, 21.0. HRMS (ESI-TOF) m/z: [M-H]$^-$ calcd for $C_{18}H_{24}NO_4$ 318.1705; found 318.1707.

(*R*)-6-Hydroxy-2-methyl-2-nitro-1-phenyl-6-propylnonan-5-one (3Af)

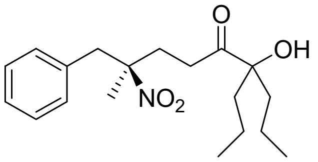

Prepared following the General Procedure using α-hydroxy enone **2f** (34.0 mg, 0.2 mmol). The product was isolated as a colorless oil (55.0 mg, 0.164 mmol, 82% yield). The enantiomeric ratio (86:14) was determined by chiral HPLC analysis (IA, hexane/isopropanol = 99/1, flow rate = 1.0 mL/min, λ = 202 nm) t_R = 21.9 min (minor), t_R = 24.9 min (major). ^{1}H NMR (400 MHz, $CDCl_3$) δ 7.34 – 7.27 (m, 3H), 7.12 – 7.07 (m, 2H), 3.60 (s, 1H), 3.35 (d, *J* = 13.9 Hz, 1H), 3.09 (d, *J* = 13.9 Hz, 1H), 2.48 – 2.40 (m, 3H), 2.10 (m, 1H), 1.70 – 1.57 (m, 4H), 1.49 (s, 3H), 1.45 – 1.35 (m, 2H), 0.98 – 0.84 (m, 8H). ^{13}C NMR (100 MHz, $CDCl_3$) δ 212.8, 134.2, 130.0, 128.6, 127.7, 91.0, 82.0, 46.5, 41.2, 33.1, 30.9, 21.5, 16.7, 14.3. HRMS (ESI-TOF) m/z: [M-H]$^-$ calcd for $C_{19}H_{28}NO_4$ 334.2018; found 334.2020.

(*R*)-6-Hydroxy-6-isobutyl-2,8-dimethyl-2-nitro-1-phenylnonan-5-one (3Ag)

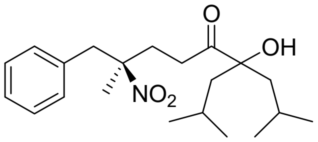

Prepared following the General Procedure using α-hydroxy enone **2g** (39.6 mg, 0.2 mmol). The product was isolated as a colorless oil (45.8 mg, 0.126 mmol, 63% yield). The enantiomeric ratio (86:14) was determined by chiral HPLC analysis (IA, hexane/isopropanol = 99/1, flow rate = 1.0 mL/min, λ = 251 nm) t_R = 10.5 min (minor), t_R = 11.8 min (major). ^{1}H NMR (400 MHz, $CDCl_3$) δ 7.35 – 7.30 (m, 3H), 7.14 – 7.09 (m, 2H), 3.67 (s, 1H), 3.40 (d, *J* = 13.9 Hz, 1H), 3.10 (d, *J* = 13.9 Hz, 1H), 2.59 – 2.41 (m, 3H), 2.14 (ddd, *J* = 14.1, 9.4, 6.4 Hz, 1H), 1.72 – 1.53 (m, 6H), 1.51 (s, 3H), 0.96 (d, *J* = 6.4 Hz, 3H), 0.95 (d, *J* = 6.4 Hz, 3H), 0.82 (d, *J* = 6.4 Hz, 3H), 0.81 (d, *J* = 6.4 Hz, 3H). ^{13}C NMR (75 MHz, $CDCl_3$) δ (ppm) = 213.6, 134.2, 130.0, 128.6, 127.7, 91.0, 82.5, 48.3, 46.5, 33.3, 31.2, 24.3, 24.0, 21.4. HRMS (ESI-TOF) m/z: [M-H]$^-$ calcd for $C_{21}H_{32}NO_4$ 362.2331; found 362.2333.

(*R*)-2-Benzyl-2-hydroxy-6-methyl-6-nitro-1,7-diphenylheptan-3-one (3Ah)

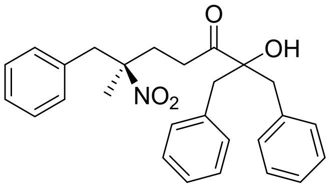

Prepared following the General Procedure using α-hydroxy enone **2h** (53.2 mg, 0.2 mmol**).** The product was isolated as a white solid (64.7 mg, 0.150 mmol, 75% yield). m.p.: 131-133 °C. The enantiomeric ratio (91:9) was determined by chiral HPLC analysis (Phenomenex Lux 3μm Cellulose 2, hexane/isopropanol = 95/5, flow rate = 1.0 mL/min, λ = 202 nm) t_R = 9.1 min (major), t_R = 11.4 min (minor). ^{1}H NMR (300 MHz, $CDCl_3$) δ 7.36 – 7.13 (m, 13H), 7.06 – 6.96 (m, 2H), 3.25 (d, *J* = 13.9 Hz, 1H), 3.17 (dd, *J* = 13.7, 7.3 Hz, 2H), 3.04 – 2.94 (m, 2H), 2.91 (d, *J* = 13.9 Hz, 1H), 2.27 – 2.18 (m, 2H), 2.19 – 2.05 (m, 1H), 1.83 – 1.68 (m, 1H), 1.59 (s, 1H), 1.26 (s, 3H). ^{13}C NMR

(75 MHz, $CDCl_3$) δ 212.8, 135.4, 134.4, 130.2, 128.7, 128.6, 127.7, 127.4, 127.3, 91.0, 83.1, 46.2, 45.4, 45.1, 33.8, 32.6, 21.3. HRMS (ESI-TOF) m/z: $[M-H]^-$ calcd for $C_{27}H_{28}NO_4$ 430.2018; found 430.2021.

(*R*)-1,1-Bis(4-fluorophenyl)-1-hydroxy-5-methyl-5-nitro-6-phenylhexan-2-one (3Ai)

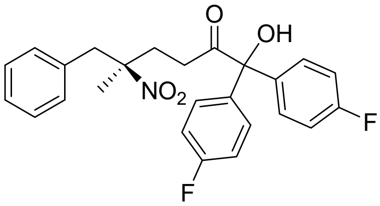

Prepared following the General Procedure using α-hydroxy enone **2i** (54.8 mg, 0.2 mmol). The product was isolated as a colorless oil (70.3 mg, 0.160 mmol, 80% yield). The enantiomeric ratio (82:18) was determined by chiral HPLC analysis (Phenomenex Lux 3μm Cellulose 2, hexane/isopropanol = 98/2, flow rate = 1.0 mL/min, λ = 202 nm) t_R = 41.9 min (minor), t_R = 47.4 min (major). ^{1}H NMR (400 MHz, $CDCl_3$) δ 7.32 – 7.26 (m, 8H), 7.10 – 6.99 (m, 3H), 7.02 – 6.99 (m, 2H), 4.24 (s, 1H), 3.24 (d, *J* = 13.9 Hz, 1H), 3.00 (d, *J* = 13.9 Hz, 1H), 2.68 – 2.48 (m, 2H), 2.39 – 2.24 (m, 1H), 2.01 – 1.87 (m, 1H), 1.35 (s, 3H). ^{13}C NMR (100 MHz, $CDCl_3$) δ 209.2, 162.7 (d, *J* = 249.5 Hz), 136.8 (2d, *J* = 4.0 Hz, 2C), 134.0, 130.0, 129.7 (2d, *J* = 7.1 Hz, 2C), 128.6, 127.7, 115.7 (2d, *J* = 22.2 Hz, 2C), 90.8, 84.9, 46.3, 33.5, 33.0, 21.4. ^{19}F (471 MHz) δ –112.9, –112.8. HRMS (ESI-TOF) m/z: $[M-H]^-$ calcd for $C_{25}H_{22}F_2NO_4$ 438.1517; found 438.1515.

(*R*)-1,1-Bis(3,5-dimethylphenyl)-1-hydroxy-5-methyl-5-nitro-6-phenylhexan-2-one (3Aj)

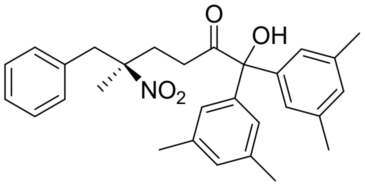

Prepared following the General Procedure using α-hydroxy enone **2j** (58.8 mg, 0.2 mmol). The product was isolated as a colorless oil (68.9 mg, 0.150 mmol, 75% yield). The enantiomeric ratio (85:15) was determined by chiral HPLC analysis (IC-3, hexane/isopropanol = 98/2, flow rate = 1.0 mL/min, λ = 202 nm) t_R = 14.2 min (minor), t_R = 16.3 min (major). ^{1}H NMR (400 MHz, $CDCl_3$) δ 7.36 – 7.20 (m, 3H), 7.08 – 6.95 (m, 4H), 6.92 (s, 2H), 6.90 (s, 2H), 4.36 (s, 1H), 3.23 (d, *J* = 13.8 Hz, 1H), 2.96 (d, *J* = 13.8 Hz, 1H), 2.70 – 2.54 (m, 2H), 2.39 – 2.22 (m, 1H), 2.31 (s, 6H), 2.29 (s, 6H), 1.89 (ddd, *J* = 14.7, 9.7, 6.4 Hz, 1H), 1.32 (s, 3H). ^{13}C NMR (100 MHz, $CDCl_3$) δ 210.0, 141.0, 138.1, 134.2, 130.0, 128.6, 127.6, 125.7, 125.7, 91.0, 86.0, 46.3, 34.0, 33.1, 21.4, 21.2. HRMS (ESI-TOF) m/z: $[M-H]^-$ calcd for $C_{29}H_{32}NO_4$ 458.2331; found 458.2334.

(*R*)-2-(3,5-Bis(trifluoromethyl)benzyl)-1-(3,5-bis(trifluoromethyl)phenyl)-2-hydroxy-6-methyl-6-nitro-7-phenylheptan-3-one (3Ak)

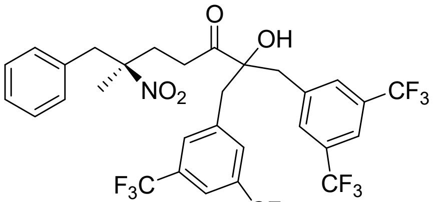

Prepared following the General Procedure using α-hydroxy enone **2k** (107.6 mg, 0.2 mmol). The product was isolated as a white solid (105.5 mg, 0.150 mmol, 75% yield). m.p.: 105-108 °C. The enantiomeric ratio (87:13) was determined by chiral HPLC analysis (Phenomenex Lux 3 μm Cellulose 1, hexane/ethanol = 99/1, flow rate = 1.0 mL/min, 0 °C, λ = 202 nm) t_R = 48.8 min (major), t_R = 54.6 min (minor). ^{1}H NMR (400 MHz, $CDCl_3$) δ 7.79 (s, 2H), 7.69 (s, 4H), 7.30 – 7.27 (m, 3H), 7.05 – 7.00 (m, 2H), 3.61 (s, 1H), 3.28 (d, *J* = 13.9 Hz, 1H), 3.23 – 3.06 (m, 4H), 3.01 (d, *J* = 13.8 Hz, 1H), 2.63 – 2.49 (m, 1H), 2.44 – 2.32 (m, 1H), 2.23 – 2.16 (m, 1H), 1.88 – 1.84 (m, 1H), 1.40 (s, 3H). ^{13}C NMR (100 MHz, $CDCl_3$) δ 209.8, 137.2 (2s, 2C), 133.8, 131.8 (2q, *J* = 33.4, 2C), 130.2, 130.0, 128.7, 127.8, 123.1 (2q, *J* = 272.8, 2C), 121.5 (m), 90.7, 81.9, 46.7, 44.0, 43.6, 32.4, 21.1. ^{19}F (471 MHz) δ –62.9, –62.8. HRMS (ESI-TOF) m/z: $[M-H]^-$ calcd for $C_{31}H_{24}F_{12}NO_4$ 702.1514; found 702.1516.

(*R*)-2-Hydroxy-6-methyl-1-(naphthalen-2-yl)-2-(naphthalen-2-ylmethyl)-6-nitro-7-phenylheptan-3-one (3Al)

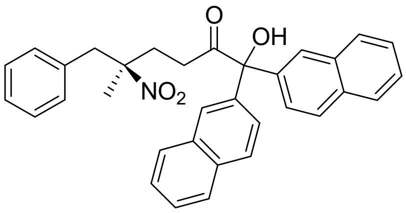

Prepared following the General Procedure using α-hydroxy enone **2l** (67.6 mg, 0.2 mmol). The product was isolated as a slightly orange foam (64.4 mg, 0.128 mmol, 64% yield). The enantiomeric ratio (87:13) was determined by chiral HPLC analysis (OD-H, hexane/isopropanol = 80/20, flow rate = 0.5 mL/min, λ = 202 nm) t_R = 49.1 min (minor), t_R = 65.3 min (major). ^1H NMR (400 MHz, $CDCl_3$) δ 7.92 – 7.76 (m, 8H), 7.59 – 7.46 (m, 4H), 7.43 (d, *J* = 8.6 Hz, 2H), 7.24 – 7.14 (m, 3H), 6.99 – 6.90 (m, 2H), 4.52 (s, 1H), 3.22 (d, *J* = 13.9 Hz, 1H), 2.96 (d, *J* = 13.9 Hz, 1H), 2.85 – 2.63 (m, 2H), 2.51 – 2.30 (m, 1H), 2.14 – 1.88 (m, 1H), 1.31 (s, 3H). ^{13}C NMR (100 MHz, $CDCl_3$) δ 209.6, 138.6, 138.4, 134.1, 133.0, 132.9, 130.0, 128.7, 128.6, 128.5, 128.4, 127.6, 127.2, 126.9, 126.8, 126.6, 125.70, 125.6, 90.9, 86.2, 46.3, 33.5, 33.4, 21.4. HRMS (ESI-TOF) m/z: $[M-H]^-$ calcd for $C_{33}H_{28}NO_4$ 502.2018; found 502.2015.

(*R*)-1-(9-Hydroxy-9H-fluoren-9-yl)-4-methyl-4-nitro-5-phenylpentan-1-one (3Am)

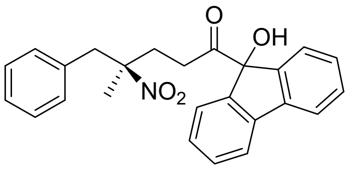

Prepared following the General Procedure using α-hydroxy enone **2m** (47.2 mg, 0.2 mmol). The product was isolated as a yellow solid (64.2 mg, 0.160 mmol, 80% yield). m.p.: 92-96 °C. The enantiomeric ratio (87:13) was determined by chiral HPLC analysis (IA, hexane/isopropanol = 98/2, flow rate = 1.0 mL/min, λ = 202 nm) t_R = 64.2 min (major), t_R = 71.8 min (minor). ^1H NMR (400 MHz, $CDCl_3$) δ 7.74 (m, 2H), 7.50 – 7.40 (m, 2H), 7.37 – 7.31 (m, 1H), 7.30 – 7.23 (m, 6H), 6.90 – 6.83 (m, 2H), 5.00 (s, 1H), 3.06 (d, *J* = 13.9 Hz, 1H), 2.80 (d, *J* = 13.9 Hz, 1H), 2.13 (m, 1H), 1.92 – 1.78 (m, 3H), 1.12 (s, 3H). ^{13}C NMR (100 MHz, $CDCl_3$) δ 207.9, 143.9 (2s, 2C), 141.5 (2s, 2C), 134.0, 130.2 (2s, 2C), 130.0, 128.6 (2s, 2C), 128.5 (2s, 2C), 127.6, 123.9 (2s, 2C), 120.9, 90.6, 88.3, 45.7, 33.4, 29.5, 21.2. HRMS (ESI-TOF) m/z: $[M-H]^-$ calcd for $C_{25}H_{22}NO_4$ 400.1549; found 400.1546.

4.3. Reaction scope

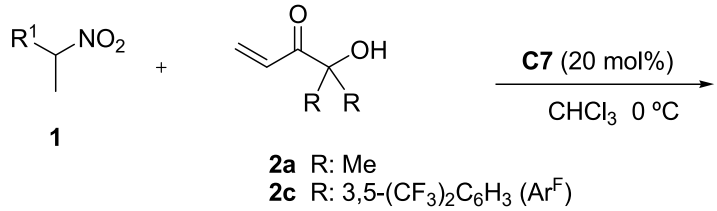

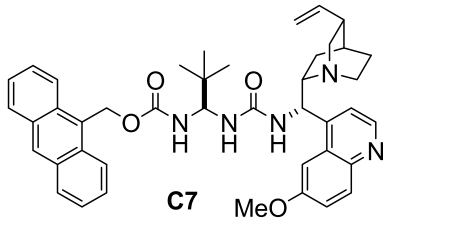

**3Ac**	R^1: $PhCH_2$	**3Ic**	R^1: 3-$MeOPhCH_2$
**3Bc**	R^1: 4-$MePhCH_2$	**3Jc**	R^1: 3,5-$(CF_3)_2PhCH_2$
**3Cc**	R^1: 4-$ClPhCH_2$	**3Ja**	R^1: 3,5-$(CF_3)_2PhCH_2$
**3Dc**	R^1: 4-NO_2PhCH_2	**3Kc**	R^1: Naphth-1-yl-CH_2
**3Ec**	R^1: 4-$MeOPhCH_2$	**3Lc**	R^1: Naphth-2-yl-CH_2
**3Ea**	R^1: 4-$MeOPhCH_2$	**3Mc**	R^1: Benzo[*d*](1,3)dioxol-5-CH_2
**3Fc**	R^1: 2-$MePhCH_2$	**3Nc**	R^1: Ph
**3Fa**	R^1: 2-$MePhCH_2$	**3Na**	R^1: Ph
**3Gc**	R^1: 3-$MePhCH_2$	**3Oc**	R^1: $CH_3(CH_2)_5$
**3Hc**	R^1: 3-$ClPhCH_2$	**3Oa**	R^1: $CH_3(CH_2)_5$

GENERAL PROCEDURE:

To a stirred solution of 4-hydroxy-4-methyl-1-penten-3-one **2a** (22.8 mg, 0.2 mmol, 1 eq) or 1,1-bis(3,5-bis(trifluoromethyl)phenyl)-1-hydroxybut-3-en-2-one **2c** (102.0 mg, 0.2 mmol, 1 eq) and the corresponding nitroalkane **1** (1 mmol, 5 eq) in $CHCl_3$ (0.6 mL) at 0 °C, catalyst **C7** (27.4 mg, 0.04 mmol, 20 mol%) was added and the mixture was stirred at this temperature. The reaction was followed by TLC (1:1 Hexane: EtOAc). After reaction completion (see table 3), the mixture was concentrated under reduced pressure and purified by flash column chromatography on silica gel (hexane:EtOAc 98:2 to 90:10). Enantiomeric ratios were determined by HPLC using a chiral column.

The racemic addition adducts were obtained following the above procedure, using TEA (30 mol%) as catalyst.

(*R*)-1,1-Bis(3,5-Bis(trifluoromethyl)phenyl)-1-hydroxy-5-methyl-5-nitro-6-phenylhexan-2-one (3Ac)

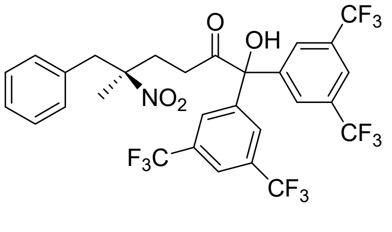

Prepared following the General Procedure starting from **2c** and nitroalkane **1A** (165.2 mg, 1 mmol, 5 eq.). The product was isolated as a white solid (94.6 mg, 0.140 mmol, 70% yield). m.p.: 136-138 °C. The enantiomeric ratio (96:4) was determined by chiral HPLC analysis (Phenomenex Lux 3µm Cellulose 1, hexane/isopropanol = 98/2, flow rate = 1.0 mL/min, 0 °C, λ = 202 nm) t_R =22.7 min (minor), t_R = 27.8 min (major). $[\alpha]_D^{25}$ = –9.5 (c=0.4, CH_2Cl_2). ^1H NMR (400 MHz, $CDCl_3$) δ 7.94 (s, 2H), 7.78 (s, 4H), 7.31 – 7.24 (m, 3H), 7.07 – 6.98 (m, 2H), 4.24 (s, 1H), 3.26 (d, *J* = 13.9 Hz, 1H), 3.06 (d, *J* = 13.9 Hz, 1H), 2.68 (m, 2H), 2.40 (m, 1H), 2.03 (m, 1H), 1.41 (s, 3H). ^{13}C NMR (100 MHz, $CDCl_3$) δ 207.1, 142.5 (2s, 2C), 133.8, 132.6 (q, *J* = 33.7 Hz), 130.0, 128.7, 127.8, 127.6, 123.3 (m), 122.8 (q, *J* = 273.0 Hz), 90.6, 84.4, 46.5, 33.1, 32.7, 21.6. ^{19}F (471 MHz) δ –63.0. HRMS (ESI-TOF) m/z: $[M-H]^-$ calcd for $C_{29}H_{20}F_{12}NO_4$ 674.1206; found 674.1194.

EXPERIMENTAL PROCEDURE FOR 1 mmol SCALE:

To a stirred solution of 1,1-bis(3,5-bis(trifluoromethyl)phenyl)-1-hydroxybut-3-en-2-one **2c** (510 mg, 1 mmol, 1 eq) and (2-Nitropropyl)benzene **1A** (5 mmol, 826 mg) in $CHCl_3$ (3 mL) at 0 °C, catalyst **C7** (137 mg, 0.2 mmol, 20 mol%) was added and the mixture was stirred at this temperature. After reaction completion (95 h), the mixture was concentrated under reduced pressure and purified by flash column chromatography on silica gel (hexane:EtOAc 98:2). The product was isolated as a white solid (486 mg, 0.72 mmol, 72% yield). The enantiomeric ratio (96:4) was determined by chiral HPLC analysis.

(*R*)-1,1-Bis(3,5-Bis(trifluoromethyl)phenyl)-1-hydroxy-5-methyl-5-nitro-6-(*p*-tolyl)hexan-2-one (3Bc)

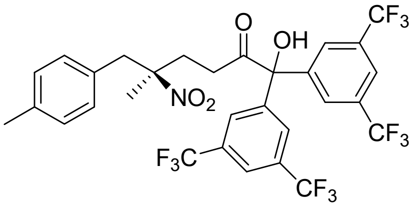

Prepared following the General Procedure starting from **2c** and nitroalkane **1B** (179.1 mg, 1 mmol, 5 eq.). The product was isolated as a white solid (89.6 mg, 0.130 mmol, 65% yield). m.p.: 147-148 °C. The enantiomeric ratio (94:6) was determined by chiral HPLC analysis (Phenomenex Lux 3µm Cellulose 1, hexane/isopropanol = 95/5, flow rate = 1.0 mL/min, λ = 202 nm) t_R = 7.4 min (minor), t_R = 8.2 min (major). $[\alpha]_D^{25}$ = –10.5 (c=0.1, CH_2Cl_2). ^{1}H NMR (400 MHz, $CDCl_3$) δ 7.94 (s, 2H), 7.78 (s, 4H), 7.07 (d, *J* = 7.9 Hz, 2H), 6.90 (d, *J* = 7.9 Hz, 2H), 4.17 (s, 1H), 3.20 (d, *J* = 13.9 Hz, 1H), 3.02 (d, *J* = 13.9 Hz, 1H), 2.74 – 2.61 (m, 2H), 2.45 – 2.33 (m, 1H), 2.31 (s, 3H), 2.06 – 1.92 (m, 1H), 1.40 (s, 3H). ^{13}C NMR (100 MHz, $CDCl_3$) δ 207.1, 142.6 (2s, 2C), 137.6, 132.6 (q, *J* = 33.8 Hz), 130.7, 129.8, 129.3, 127.6, 123.2 (m), 122.8 (q, *J* = 273.2 Hz), 90.7, 84.4, 46.1, 33.1, 32.6, 21.6, 21.0. ^{19}F (471 MHz) δ –63.3. HRMS (ESI-TOF) m/z: $[M-H]^-$ calcd for $C_{30}H_{22}F_{12}NO_4$ 688.1363; found 688.1368.

(*R*)-1,1-Bis(3,5-Bis(trifluoromethyl)phenyl)-6-(4-chlorophenyl)-1-hydroxy-5-methyl-5-nitrohexan-2-one (3Cc)

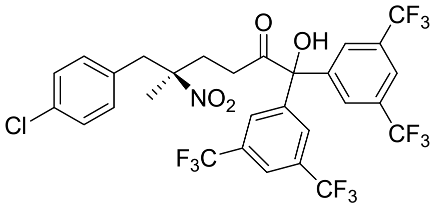

Prepared following the General Procedure starting from **2c** and nitroalkane **1C** (200 mg, 1 mmol, 5 eq.). The product was isolated as a white solid (93.7 mg, 0.132 mmol, 66% yield). m.p.: 134-136 °C. The enantiomeric ratio (96:4) was determined by chiral HPLC analysis (IB, hexane/isopropanol = 99/1, flow rate = 1.0 mL/min, λ = 202 nm) t_R = 20.9 min (minor), t_R = 22.3 min (major). $[\alpha]_D^{25}$ = –1.9 (c=0.1, CH_2Cl_2). ^{1}H NMR (400 MHz, $CDCl_3$) δ 7.95 (s, 2H), 7.77 (s, 4H), 7.25 (m, 2H), 6.96 (d, *J* = 8.3 Hz, 2H), 4.12 (s, 1H), 3.24 (d, *J* = 14.0 Hz, 1H), 3.01 (d, *J* = 14.0 Hz, 1H), 2.70 (t, *J* = 7.6 Hz, 2H), 2.39 (m, 1H), 2.01 (m, 1H), 1.39 (s, 3H). ^{13}C NMR (100 MHz, $CDCl_3$) δ 207.0, 142.5 (2s, 2C), 134.0, 132.6 (q, *J* = 34.0 Hz), 132.2, 131.2, 128.9, 127.6, 123.3 (m), 122.8 (q, *J* = 274.3 Hz), 90.4, 84.4, 45.8, 33.0, 32.8, 21.4. ^{19}F (471 MHz) δ –63.3. HRMS (ESI-TOF) m/z: $[M-H]^-$ calcd for $C_{29}H_{19}ClF_{12}NO_4$ 708.0816; found 708.0812.

(*R*)-1,1-Bis(3,5-bis(trifluoromethyl)phenyl)-1-hydroxy-5-methyl-5-nitro-6-(4-nitrophenyl)hexan-2-one (3Dc)

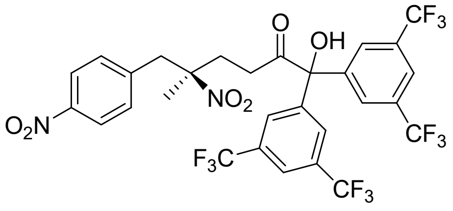

Prepared following the General Procedure starting from **2c** and nitroalkane **1D** (210 mg, 1 mmol, 5 eq.). The product was isolated as a white solid (86.4 mg, 0.120 mmol, 60% yield). m.p.: 102-104 °C. The enantiomeric ratio (92:8) was determined by chiral HPLC analysis (Phenomenex Lux 3µm Cellulose 1, hexane/isopropanol = 95/5, flow rate = 1.0 mL/min, λ = 202 nm) t_R = 22.0 min (major), t_R = 24.6 min (minor). $[\alpha]_D^{25}$ = –9.7 (c=0.6, CH_2Cl_2). ^{1}H NMR

(400 MHz, $CDCl_3$) δ 8.16 (d, *J* = 8.7 Hz, 2H), 7.95 (s, 2H), 7.78 (s, 4H), 7.22 (d, *J* = 8.7 Hz, 2H), 4.05 (s, 1H), 3.41 (d, *J* = 13.9 Hz, 1H), 3.12 (d, *J* = 13.9 Hz, 1H), 2.75 (t, *J* = 7.6 Hz, 2H), 2.44 (m, 1H), 2.07 (m, 1H), 1.42 (s, 3H). ^{13}C NMR (100 MHz, $CDCl_3$) δ 206.9, 147.7, 142.5 (2s, 2C), 141.0, 132.7 (q, *J* = 33.9 Hz), 130.9, 127.5, 123.9, 123.3 (m), 122.8 (q, *J* = 274.3 Hz), 90.1, 84.4, 46.0, 33.1, 32.9, 21.3. ^{19}F (471 MHz) δ –63.4. HRMS (ESI-TOF) m/z: $[M-H]^-$ calcd for $C_{29}H_{19}F_{12}N_2O_6$ 719.1057; found 719.1050.

(*R*)-1,1-Bis(3,5-bis(trifluoromethyl)phenyl)-1-hydroxy-6-(4-methoxyphenyl)-5-methyl-5-nitrohexan-2-one (3Ec)

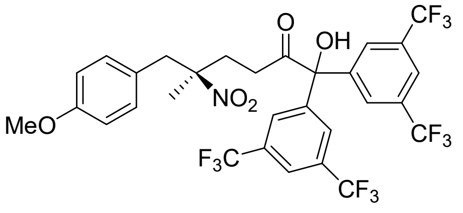

Prepared following the General Procedure starting from **2c** and nitroalkane **1E** (195 mg, 1 mmol, 5 eq.). The product was isolated as a white solid (91.7 mg, 0.130 mmol, 65% yield). m.p.: 151-153 °C. The enantiomeric ratio (90:10) was determined by chiral HPLC analysis (IB, hexane/isopropanol = 98/2, flow rate = 1.0 mL/min, λ = 202 nm) t_R = 14.4 min (minor), t_R = 15.6 min (major). $[\alpha]_D^{25}$ = –8.7 (c=0.6, CH_2Cl_2). ^1H NMR (400 MHz, $CDCl_3$) δ 7.94 (s, 2H), 7.78 (s, 4H), 6.93 (d, *J* = 8.7 Hz, 2H), 6.79 (d, *J* = 8.7 Hz, 2H), 4.27 (s, 1H), 3.77 (s, 3H), 3.19 (d, *J* = 14.0 Hz, 1H), 2.99 (d, *J* = 14.0 Hz, 1H), 2.69 (m, 2H), 2.38 (m, 1H), 2.01 (m, 1H), 1.39 (s, 3H). ^{13}C NMR (100 MHz, $CDCl_3$) δ 207.2, 159.2, 142.6 (2s, 2C), 132.6 (q, *J* = 33.8 Hz), 131.0, 127.6, 125.7, 123.2 (m), 122.8 (q, *J* = 274.2 Hz), 114.0, 90.8, 84.4, 55.2, 45.8, 33.1, 32.6, 21.5. ^{19}F (471 MHz) δ –63.2. HRMS (ESI-TOF) m/z: $[M-H]^-$ calcd for $C_{30}H_{22}F_{12}NO_5$ 704.1312; found 704.1300.

(*R*)-2-Hydroxy-7-(4-methoxyphenyl)-2,6-dimethyl-6-nitroheptan-3-one (3Ea)

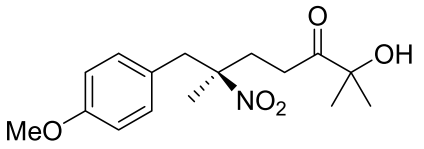

Prepared following the General Procedure starting from **2a** and nitroalkane **1E** (195 mg, 1 mmol, 5 eq.) carrying out the reaction at room temperature. The product was isolated as a colorless oil (40.8 mg, 0.132 mmol, 66% yield). The enantiomeric ratio (90:10) was determined by chiral HPLC analysis (IA, hexane/isopropanol = 95/5, flow rate = 0.5 mL/min, λ = 202 nm) t_R = 58.1 min (major), t_R = 62.7 min (minor). ^1H NMR (400 MHz, $CDCl_3$) δ 7.00 (d, *J* = 8.7 Hz, 2H), 6.82 (d, *J* = 8.7 Hz, 2H), 3.78 (s, 3H), 3.42 (s, 1H), 3.29 (d, *J* = 14.1 Hz, 1H), 3.02 (d, *J* = 14.1 Hz, 1H), 2.57 (m, 2H), 2.43 (m, 1H), 2.10 (m, 1H), 1.46 (s, 3H), 1.36 (s, 6H). ^{13}C NMR (100 MHz, $CDCl_3$) δ 212.8, 159.1, 131.1, 126.2, 114.0, 91.2, 76.5, 55.2, 45.8, 32.9, 30.3, 26.6, 26.6, 21.3. HRMS (ESI-TOF) m/z: $[M-H]^-$ calcd for $C_{16}H_{22}NO_5$ 308.1498; found 308.1490.

(*R*)-1,1-Bis(3,5-bis(trifluoromethyl)phenyl)-1-hydroxy-5-methyl-5-nitro-6-(*o*-tolyl)hexan-2-one (3Fc)

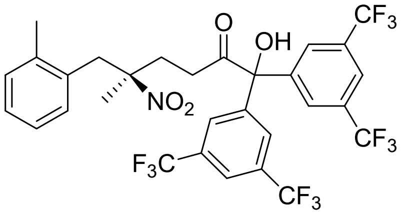

Prepared following the General Procedure starting from **2c** and nitroalkane **1F** (179 mg, 1 mmol, 5 eq.). The product was isolated as a white solid (80.0 mg, 0.116 mmol, 58% yield). m.p.: 96-98 °C. The enantiomeric ratio (89:11) was determined by chiral HPLC analysis (IB, hexane/isopropanol = 98/2, flow rate = 1.0 mL/min, λ = 202 nm) t_R = 10.5 min (minor), t_R = 11.3 min (major). ^1H NMR (400 MHz, $CDCl_3$) δ 7.94 (s, 2H), 7.78 (s, 4H), 7.22 – 7.13 (m, 2H), 7.12 – 7.05 (m, 1H), 6.90 (d, *J* = 7.5 Hz, 1H), 3.30 (d, *J* = 14.4 Hz, 1H), 3.18 (d, *J* = 14.4 Hz, 1H), 2.67 (m, 2H), 2.46 (m, 1H), 2.34 (s, 1H), 2.26 (s, 3H), 2.05 (m, 1H), 1.40 (s, 3H). ^{13}C NMR (100 MHz, $CDCl_3$) δ 207.2, 142.6 (2s, 2C), 137.0, 132.6 (q, *J* = 34.0 Hz), 132.4, 131.0, 130.4, 127.8, 127.6, 126.2, 123.2 (m), 122.8 (q, *J* = 274.3 Hz), 91.2, 84.3, 42.9, 33.2, 33.0, 21.2, 20.0. ^{19}F (471 MHz) δ –63.1. HRMS (ESI-TOF) m/z: $[M-H]^-$ calcd for $C_{30}H_{22}F_{12}NO_4$ 688.1363; found 688.1352.

(*R*)-2-Hydroxy-2,6-dimethyl-6-nitro-7-(*o*-tolyl)heptan-3-one (3Fa)

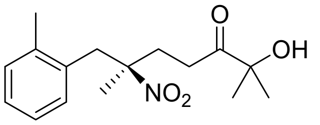

Prepared following the General Procedure starting from **2a** and nitroalkane **1F** (179 mg, 1 mmol, 5 eq.) carrying out the reaction at room temperature. The product was isolated as a colorless oil (39.9 mg, 0.136 mmol, 68% yield). The enantiomeric ratio (83:17) was determined by chiral HPLC analysis (IA, hexane/isopropanol = 95/5, flow rate = 1.0 mL/min, λ = 202 nm) t_R = 20.1 min (major), t_R = 39.9 min (minor). ^{1}H NMR (400 MHz, $CDCl_3$) δ 7.18 (m, 2H), 7.16 – 7.09 (m, 1H), 6.97 (d, *J* = 7.3 Hz, 1H), 3.41 (s, 1H), 3.39 (d, *J* = 14.7 Hz, 1H), 3.21 (d, *J* = 14.7 Hz, 1H), 2.61 – 2.47 (m, 3H), 2.32 (s, 3H), 2.19 – 2.06 (m, 1H), 1.47 (s, 3H), 1.36 (s, 6H). ^{13}C NMR (100 MHz, $CDCl_3$) δ 212.8, 137.1, 132.9, 130.9, 130.4, 127.7, 126.2, 91.7, 76.5, 42.9, 33.3, 30.4, 26.6, 26.6, 20.9, 20.1. HRMS (ESI-TOF) m/z: $[M-H]^-$ calcd for 292.1549; found 292.1552.

(*R*)-1,1-Bis(3,5-bis(trifluoromethyl)phenyl)-1-hydroxy-5-methyl-5-nitro-6-(*m*-tolyl)hexan-2-one (3Gc)

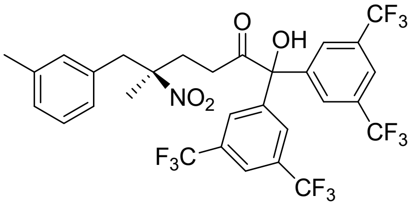

Prepared following the General Procedure starting from **2c** and nitroalkane **1G** (179 mg, 1 mmol, 5 eq.). The product was isolated as a white solid (91.0 mg, 0.132 mmol, 66% yield). m.p.: 108-110 °C. The enantiomeric ratio (94:6) was determined by chiral HPLC analysis (IB, hexane/isopropanol = 98/2, flow rate = 1.0 mL/min, λ = 202 nm) t_R = 8.4 min (minor), t_R = 9.4 min (major). $[\alpha]_D^{25}$ = –6.8 (c=0.8, CH_2Cl_2). ^{1}H NMR (400 MHz, $CDCl_3$) δ 7.94 (s, 2H), 7.78 (s, 4H), 7.15 (m, 1H), 7.08 (m, 1H), 6.82 (m, 2H), 4.23 (s, 1H), 3.22 (d, *J* = 13.8 Hz, 1H), 3.01 (d, *J* = 13.8 Hz, 1H), 2.69 (t, *J* = 7.6 Hz, 2H), 2.34 (m, 1H), 2.30 (s, 3H), 2.04 (m, 1H), 1.40 (s, 3H). ^{13}C NMR (100 MHz, $CDCl_3$) δ 207.2, 142.6 (2s, 2C), 138.4, 133.7, 132.6 (q, *J* = 34.0 Hz), 130.7, 128.5 (2C), 127.6, 127.0, 123.2 (m), 122.8 (dd, *J* = 546.2, 273.2 Hz), 90.6, 84.4, 46.4, 33.1, 32.7, 21.6, 21.3. ^{19}F (471 MHz) δ –62.9. HRMS (ESI-TOF) m/z: $[M-H]^-$ calcd for $C_{30}H_{22}F_{12}NO_4$ 688.1363; found 688.1371.

(*R*)-1,1-Bis(3,5-bis(trifluoromethyl)phenyl)-6-(3-chlorophenyl)-1-hydroxy-5-methyl-5-nitrohexan-2-one (3Hc)

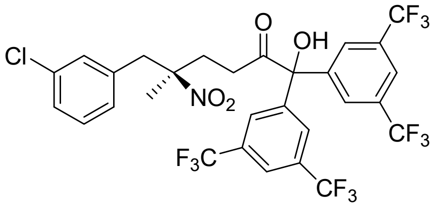

Prepared following the General Procedure starting from **2c** and nitroalkane **1H** (200 mg, 1 mmol, 5 eq.). The product was isolated as a white solid (100.8 mg, 0.142 mmol, 71% yield). m.p.: 100-102 °C. The enantiomeric ratio (93:7) was determined by chiral HPLC analysis (Phenomenex Lux 3μm Cellulose 1, hexane/isopropanol = 98/2, flow rate = 1.0 mL/min, λ = 202 nm) t_R = 19.2 min (minor), t_R = 22.9 min (major). $[\alpha]_D^{25}$ = –6.0 (c=0.9, CH_2Cl_2). ^{1}H NMR (400 MHz, $CDCl_3$) δ 7.95 (s, 2H), 7.78 (s, 4H), 7.27 (m, 1H), 7.21 (t, *J* = 7.7 Hz, 1H), 7.04 (t, *J* = 1.7 Hz, 1H), 6.91 (d, *J* = 7.5 Hz, 1H), 4.20 (s, 1H), 3.27 (d, *J* = 14.0 Hz, 1H), 3.00 (d, *J* = 14.0 Hz, 1H), 2.71 (t, *J* = 7.6 Hz, 2H), 2.41 (dt, *J* = 15.0, 7.4 Hz, 1H), 2.04 (dt, *J* = 15.2, 7.5 Hz, 1H), 1.40 (s, 3H). ^{13}C NMR (100 MHz, $CDCl_3$) δ 207.0, 142.5 (2s, 2C), 135.7, 134.5, 132.6 (q, *J* = 33.8 Hz), 130.0 (2s, 2C), 128.1, 127.6, 123.3 (m), 122.8 (q, *J* = 274.3 Hz), 90.3, 84.4, 45.9, 33.0, 32.9, 21.4. ^{19}F (471 MHz) δ –63.1. HRMS (ESI-TOF) m/z: $[M-H]^-$ calcd for $C_{29}H_{19}ClF_{12}NO_4$ 708.0816; found 708.0820.

(*R*)-1,1-Bis(3,5-bis(trifluoromethyl)phenyl)-1-hydroxy-6-(3-methoxyphenyl)-5-methyl-5-nitrohexan-2-one (3Ic)

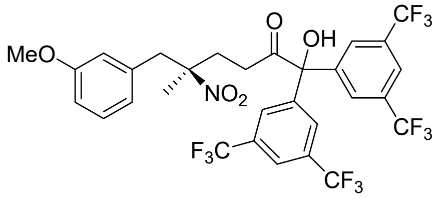

Prepared following the General Procedure starting from **2c** and nitroalkane **1I** (195 mg, 1 mmol, 5 eq.). The product was isolated as a syrup (95.9 mg, 0.136 mmol, 68% yield). The enantiomeric ratio (92:8) was determined by chiral HPLC analysis (IB, hexane/isopropanol = 98/2, flow rate = 1.0 mL/min, λ = 202 nm) t_R = 18.9 min (minor), t_R =22.3 min (major). $[\alpha]_D^{25}$ = –4.0 (c=0.7, CH_2Cl_2). ^1H NMR (400 MHz, $CDCl_3$) δ 7.94 (s, 2H), 7.78 (s, 4H), 7.18 (t, *J* = 7.9 Hz, 1H), 6.80 (d, *J* = 8.3 Hz, 1H), 6.60 (d, *J* = 7.5 Hz, 1H), 6.56 (s, 1H), 4.23 (s, 1H), 3.76 (s, 3H), 3.24 (d, *J* = 13.7 Hz, 1H), 3.02 (d, *J* = 13.7 Hz, 1H), 2.70 (t, *J* = 7.6 Hz, 2H), 2.40 (m, 1H), 2.04 (m, 1H), 1.41 (s, 3H). ^{13}C NMR (100 MHz, $CDCl_3$) δ 207.3, 159.6, 142.6 (2s, 2C), 135.3, 132.5 (q, *J* = 33.8 Hz), 129.6, 127.6, 123.2 (m), 122.3, 122.8 (q, *J* = 273.5 Hz), 116.0, 112.8, 90.6, 84.3, 55.2, 46.4, 33.1, 32.8, 21.7. ^{19}F (471 MHz) δ -63.0. HRMS (ESI-TOF) m/z: $[M-H]^-$ calcd for $C_{30}H_{22}F_{12}NO_5$ 704.1312; found 704.1303.

(*R*)-1,1,6-Tris(3,5-bis(trifluoromethyl)phenyl)-1-hydroxy-5-methyl-5-nitrohexan-2-one (3Jc)

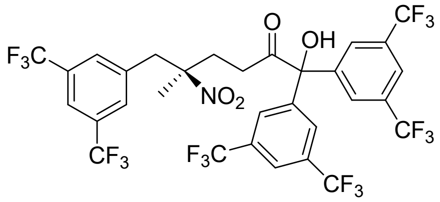

Prepared following the General Procedure starting from **2c** and nitroalkane **1J** (301 mg, 1 mmol, 5 eq.). The product was isolated as a white solid (129.8 mg, 0.160 mmol, 80% yield). m.p.: 106-108 °C. The enantiomeric ratio (87:13) was determined by chiral HPLC analysis (Phenomenex Lux 3µm Cellulose 1, hexane/isopropanol = 98/2, flow rate = 1.0 mL/min, λ = 202 nm) t_R =13.5 min (major), t_R = 15.2 min (minor). ^1H NMR (400 MHz, $CDCl_3$) δ 7.95 (s, 2H), 7.82 (s, 1H), 7.79 (s, 4H), 7.50 (s, 2H), 4.02 (s, 1H), 3.45 (d, *J* = 14.1 Hz, 1H), 3.13 (d, *J* = 14.1 Hz, 1H), 2.76 (t, *J* = 7.5 Hz, 2H), 2.43 (dt, *J* = 14.9, 7.4 Hz, 1H), 2.10 (dt, *J* = 15.0, 7.6 Hz, 1H), 1.42 (s, 3H). ^{13}C NMR (100 MHz, $CDCl_3$) δ 206.8, 142.4 (2s, 2C), 136.3, 132.7 (q, *J* = 33.9 Hz), 132.2 (q, *J* = 33.6 Hz), 130.1, 127.5, 123.3 (m), 123.0 (q, *J* = 273.8 Hz), 122.8 (q, *J* = 274.2 Hz), 122.0 (m), 89.9, 84.4, 45.5, 33.1, 32.9, 21.3. ^{19}F (471 MHz) δ –63.4, –63.3. HRMS (ESI-TOF) m/z: $[M-H]^-$ calcd for $C_{31}H_{18}F_{18}NO_4$ 810.0954; found 810.0960.

(*R*)-7-(3,5-Bis(trifluoromethyl)phenyl)-2-hydroxy-2,6-dimethyl-6-nitroheptan-3-one (3Ja)

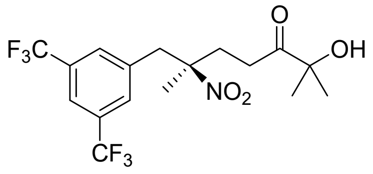

Prepared following the General Procedure starting from **2a** and nitroalkane **1J** (301 mg, 1 mmol, 5 eq.). The product was isolated as a colorless oil (68.9 mg, 0.166 mmol, 83% yield). The enantiomeric ratio (80:20) was determined by chiral HPLC analysis (Phenomenex Lux 3µm Cellulose 2, hexane/isopropanol = 95/5, flow rate = 1.0 mL/min, λ = 202 nm) t_R =16.6 min (minor), t_R = 19.9 min (major). ^1H NMR (400 MHz, $CDCl_3$) δ 7.83 (s, 1H), 7.55 (s, 2H), 3.55 (d, *J* = 14.2 Hz, 1H), 3.28 (s, 1H), 3.17 (d, *J* = 14.2 Hz, 1H), 2.63 (m, 2H), 2.45 (ddd, *J* = 15.0, 9.3, 5.7 Hz, 1H), 2.18 (ddd, *J* = 15.1, 9.0, 6.3 Hz, 1H), 1.50 (s, 3H), 1.37 (s, 6H). ^{13}C NMR (100 MHz, $CDCl_3$) δ 212.3, 136.7, 132.1 (q, *J* = 33.3 Hz), 130.2, 123.0 (q, *J* = 273.7 Hz), 121.9 (m), 90.4, 76.6, 45.4, 33.4, 30.2, 26.7, 26.7, 21.3. ^{19}F (471 MHz) δ –62.9. HRMS (ESI-TOF) m/z: $[M-H]^-$ calcd for $C_{17}H_{18}F_6NO_4$ 414.1140; found 414.1145.

(*R*)-1,1-Bis(3,5-bis(trifluoromethyl)phenyl)-1-hydroxy-5-methyl-6-(naphthalen-1-yl)-5-nitrohexan-2-one (3Kc)

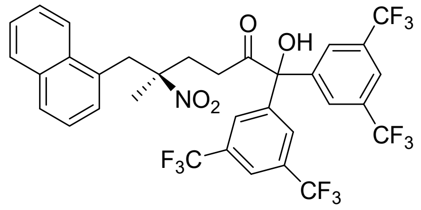

Prepared following the General Procedure starting from **2c** and nitroalkane **1K** (215 mg, 1 mmol, 5 eq.). The product was isolated as a syrup (95.8 mg, 0.132 mmol, 66% yield). The enantiomeric ratio (93:7) was determined by chiral HPLC analysis (Phenomenex Lux 3μm Cellulose 1, hexane/isopropanol = 95/5, flow rate = 1.0 mL/min, λ = 202 nm) t_R =16.2 min (minor), t_R = 21.5 min (major). $[\alpha]_D^{25}$ = –11.8 (c=0.6, CH_2Cl_2). ^1H NMR (400 MHz, $CDCl_3$) δ 7.93 (s, 2H), 7.89 – 7.83 (m, 2H), 7.77 (s, 4H), 7.55 – 7.45 (m, 3H), 7.38 (t, *J* = 7.6 Hz, 1H), 7.18 (d, *J* = 7.1 Hz, 1H), 4.19 (s, 1H), 3.73 (d, *J* = 14.6 Hz, 1H), 3.66 (d, *J* = 14.6 Hz, 1H), 2.67 (m, 2H), 2.55 (m, 1H), 2.12 (m, 1H), 1.39 (s, 3H). ^{13}C NMR (100 MHz, $CDCl_3$) δ 207.2, 142.6 (2s, 2C), 133.9, 132.6 (q, *J* = 33.8 Hz), 132.5, 130.4, 129.1, 128.7, 128.5, 127.6, 126.5, 125.8, 125.3, 123.3, 123.2 (m), 122.8 (q, *J* = 273.3 Hz), 91.6, 84.3, 41.9, 33.2, 30.9, 21.7. ^{19}F (471 MHz) δ –63.3. HRMS (ESI-TOF) m/z: $[M-H]^-$ calcd for $C_{33}H_{22}F_{12}NO_4$ 724.1363; found 724.1345.

(*R*)-1,1-Bis(3,5-bis(trifluoromethyl)phenyl)-1-hydroxy-5-methyl-6-(naphthalen-2-yl)-5-nitrohexan-2-one (3Lc)

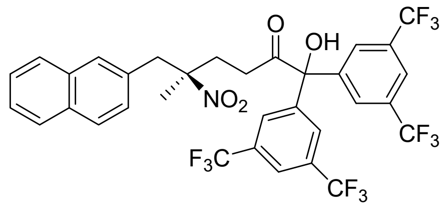

Prepared following the General Procedure starting from **2c** and nitroalkane **1L** (215 mg, 1 mmol, 5 eq.). The product was isolated as a white solid (91.4 mg, 0.126 mmol, 63% yield). m.p.: 165-167 °C. The enantiomeric ratio (95:5) was determined by chiral HPLC analysis (Phenomenex Lux 3μm Cellulose 1, hexane/isopropanol = 98/2, flow rate = 1.0 mL/min, λ = 202 nm) t_R = 27.5 min (minor), t_R = 35.2 min (major). $[\alpha]_D^{25}$ = –11.5 (c=0.2, CH_2Cl_2). ^1H NMR (400 MHz, $CDCl_3$) δ 7.93 (s, 2H), 7.80 (s, 4H), 7.78 – 7.72 (m, 2H), 7.53 – 7.45 (m, 4H), 7.13 (d, *J* = 8.4 Hz, 1H), 4.34 (s, 1H), 3.45 (d, *J* = 13.9 Hz, 1H), 3.22 (d, *J* = 13.9 Hz, 1H), 2.74 (t, *J* = 7.6 Hz, 2H), 2.47 (m, 1H), 2.06 (m, 1H), 1.45 (s, 3H). ^{13}C NMR (100 MHz, $CDCl_3$) δ 207.3, 142.7 (2s, 2C), 133.2, 132.5 (q, *J* = 33.9 Hz), 131.4, 129.1, 128.6, 128.4, 127.7, 127.7, 127.6, 127.5, 126.4, 126.2, 123.1 (m), 122.8 (q, *J* = 274.2 Hz), 90.8, 84.3, 46.6, 32.8, 30.9, 21.60. ^{19}F (471 MHz) δ –63.3. HRMS (ESI-TOF) m/z: $[M-H]^-$ calcd for $C_{33}H_{22}F_{12}NO_4$ 724.1363; found 724.1357.

(*R*)-6-(Benzo[*d*][1,3]dioxol-5-yl)-1,1-bis(3,5-bis(trifluoromethyl)phenyl)-1-hydroxy-5-methyl-5-nitrohexan-2-one (3Mc)

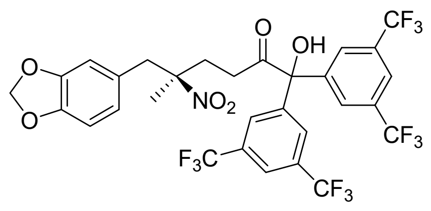

Prepared following the General Procedure starting from **2c** and nitroalkane **1M** (209 mg, 1 mmol, 5 eq.). The product was isolated as a white solid (87.8 mg, 0.122 mmol, 61% yield). m.p.: 121-123 °C. The enantiomeric ratio (94:6) was determined by chiral HPLC analysis (Phenomenex Lux 3μm Cellulose 1, hexane/isopropanol = 98/2, flow rate = 1.0 mL/min, λ = 202 nm) t_R = 29.6 min (minor), t_R = 35.7 min (major). $[\alpha]_D^{25}$ = –7.7 (c=0.5, CH_2Cl_2). ^1H NMR (400 MHz, $CDCl_3$) δ 7.95 (s, 2H), 7.78 (s, 4H), 6.70 (d, *J* = 7.8 Hz, 1H), 6.48 (s, 1H), 6.47 (d, *J* = 7.8 Hz, 1H), 5.93 (s, 2H), 4.22 (s, 1H), 3.17 (d, *J* = 14.1 Hz, 1H), 2.95 (d, *J* = 14.1 Hz, 1H), 2.70 (t, *J* = 7.7 Hz, 2H), 2.39 (dt, *J* = 15.1, 7.4 Hz, 1H), 2.00 (dt, *J* = 15.3, 7.3 Hz, 1H), 1.39 (s, 3H). ^{13}C NMR (100 MHz, $CDCl_3$) δ 207.1, 147.8, 147.3, 142.5 (2s, 2C), 132.6 (q, *J* = 33.8 Hz), 127.6, 127.2, 123.3, 123.2 (m), 122.8 (q, *J* = 273.7 Hz), 110.1, 108.4, 101.2, 90.7, 84.4, 46.3, 33.1, 32.7, 21.4. ^{19}F (471 MHz) δ –62.9. HRMS (ESI-TOF) m/z: $[M-H]^-$ calcd for $C_{30}H_{20}F_{12}NO_6$ 718.1104; found 718.1097.

(*R*)-1,1-Bis(3,5-bis(trifluoromethyl)phenyl)-1-hydroxy-5-nitro-5-phenylhexan-2-one (3Nc)

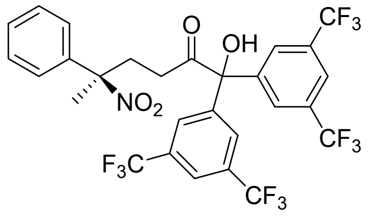

Prepared following the General Procedure starting from **2c** and commercially available nitroalkane **1N** (151 mg, 1 mmol, 5 eq.). The product was isolated as a colorless syrup (105.8 mg, 0.160 mmol, 80% yield). The enantiomeric ratio (62:38) was determined by chiral HPLC analysis (Phenomenex Lux 3μm Cellulose 1, hexane/isopropanol = 98/2, flow rate = 1.0 mL/min, λ = 202 nm) t_R =15.2 min (major), t_R = 17.1 min (minor). ^{1}H NMR (400 MHz, $CDCl_3$) δ 7.93 (s, 2H), 7.74 (s, 4H), 7.33 (m, 3H), 7.21 (m, 2H), 4.29 (s, 1H), 2.66 (t, *J* = 13.0 Hz, 2H), 2.61 (t, *J* = 13.0 Hz, 2H), 1.88 (s, 3H). ^{13}C NMR (100 MHz, $CDCl_3$) δ 207.0, 142.6 (2s, 2C), 138.4, 132.6 (q, *J* = 33.7 Hz), 129.2, 129.0, 127.6, 124.9, 123.2 (m), 122.8 (q, *J* = 274.7 Hz), 92.4, 84.3, 34.1, 33.7, 24.5. ^{19}F (471 MHz) δ –63.3. HRMS (ESI-TOF) m/z: $[M-H]^-$ calcd for $C_{28}H_{18}F_{12}NO_4$ 660.1050; found 660.1033.

(*R*)-2-hydroxy-2-methyl-6-nitro-6-phenylheptan-3-one (3Na)

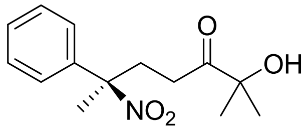

Prepared following the General Procedure starting from **2a** and commercially available nitroalkane **1N** (151 mg, 1 mmol, 5 eq.). The product was isolated as a colorless syrup (39.8 mg, 0.150 mmol, 75% yield). The enantiomeric ratio (66:34) was determined by chiral HPLC analysis (IA, hexane/isopropanol = 90/10, flow rate = 1.0 mL/min, λ = 202 nm) t_R =14.9 min (major), t_R = 20.5 min (minor). ^{1}H NMR (400 MHz, $CDCl_3$) δ 7.44 – 7.33 (m, 5H), 3.39 (s, 1H), 2.77 (dt, *J* = 14.3, 6.8 Hz, 1H), 2.64 (dt, *J* = 15.5, 7.8 Hz, 1H), 2.60 – 2.53 (m, 2H), 1.96 (s, 3H), 1.33 (s, 6H). ^{13}C NMR (100 MHz, $CDCl_3$) δ 212.7, 139.1, 129.0, 128.9, 125.2, 92.8, 76.4, 33.5, 31.1, 26.6, 24.5. HRMS (ESI-TOF) m/z: $[M-H]^-$ calcd for $C_{14}H_{18}NO_4$ 264.1236; found 264.1233.

(*S*)-1,1-Bis(3,5-bis(trifluoromethyl)phenyl)-1-hydroxy-5-methyl-5-nitroundecan-2-one (3Oc)

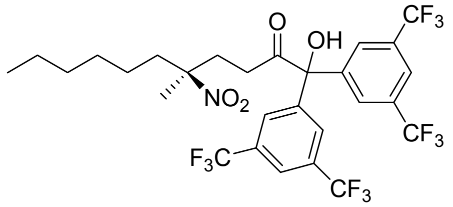

Prepared following the General Procedure starting from **2c** and nitroalkane **1O** (159 mg, 1 mmol, 5 eq.). The product was isolated as a white solid (73.6 mg, 0.110 mmol, 55% yield). m.p.: 80-82 °C. The enantiomeric ratio (81:19) was determined by chiral HPLC analysis (Phenomenex Lux 3μm Cellulose 1, hexane/isopropanol = 98/2, flow rate = 1.0 mL/min, λ = 202 nm) t_R = 5.8 min (major), t_R = 6.4 min (minor). ^{1}H NMR (400 MHz, $CDCl_3$) δ 7.95 (s, 2H), 7.79 (s, 4H), 4.28 (s, 1H), 2.67 (t, *J* = 7.5 Hz, 2H), 2.30 (m, 1H), 2.04 (m, 1H), 1.91 (m, 1H), 1.73 (m, 1H), 1.44 (s, 3H), 1.31 – 1.10 (m, 8H), 0.86 (t, *J* = 6.2 Hz, 3H). ^{13}C NMR (100 MHz, $CDCl_3$) δ 207.2, 142.6 (2s, 2C), 132.6 (q, *J* = 34.3 Hz), 127.6, 123.2 (m), 122.8 (q, *J* = 273.7 Hz), 90.2, 84.4, 40.0, 33.1, 32.9, 31.4, 29.0, 23.7, 22.4, 22.0, 13.9. ^{19}F (471 MHz) δ –63.2. HRMS (ESI-TOF) m/z: $[M-H]^-$ calcd for $C_{28}H_{26}F_{12}NO_4$ 668.1676; found 668.1679.

(*S*)-2-hydroxy-2,6-dimethyl-6-nitrododecan-3-one (3Oa)

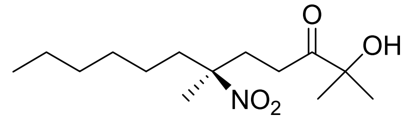

Prepared following the General Procedure starting from **2a** and nitroalkane **1O** (159 mg, 1 mmol, 5 eq.) carrying out the reaction at room temperature. The product was isolated as a colorless syrup (35.0 mg, 0.128 mmol, 64% yield). The enantiomeric ratio (97:3) was determined by chiral HPLC analysis (Phenomenex Lux 3μm Cellulose 1, hexane/isopropanol = 98/2, flow rate = 1.0 mL/min, λ = 202 nm) t_R = 13.7 min (minor), t_R = 15.7 min (major). ^{1}H NMR (400 MHz, $CDCl_3$) δ 3.46 (s, 1H), 2.70 – 2.48 (m, 2H), 2.35 (ddd, *J* = 15.2, 9.6, 5.8 Hz, 1H), 2.14 (ddd, *J* = 15.0, 12.9, 7.9 Hz, 1H), 2.03 (ddd, *J* = 13.9, 8.9, 3.0 Hz, 1H), 1.86 – 1.76 (m, 1H), 1.55 (s, 3H), 1.39 (s, 6H), 1.30

(m, 8H), 0.90 (t, *J* = 6.8 Hz, 3H). ^{13}C NMR (100 MHz, $CDCl_3$) δ 212.9, 90.7, 76.4, 40.1, 33.1, 31.5, 30.3, 29.1, 26.6, 23.8, 22.5, 21.9, 14.0. HRMS (ESI-TOF) m/z: $[M-H]^-$ calcd for $C_{14}H_{26}NO_4$ 272.1862; found 272.1865.

5. Chemical elaboration of adducts

5.1. Synthesis of acids and methyl esters

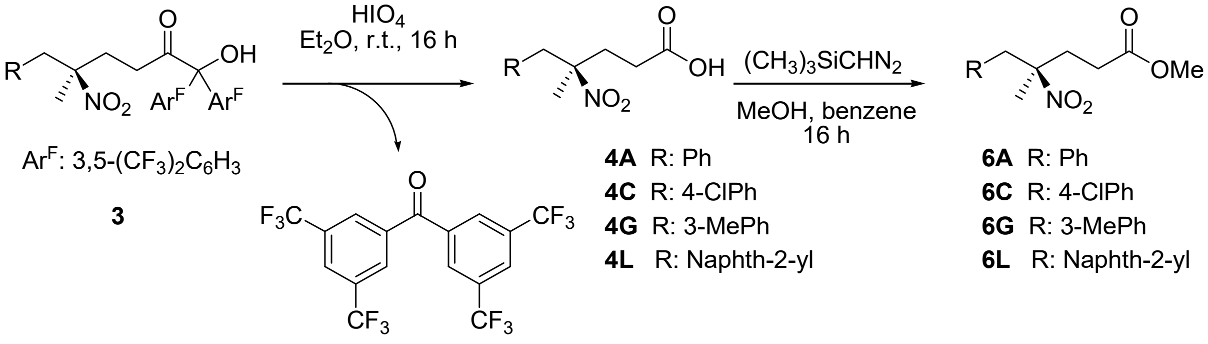

<u>*GENERAL PROCEDURE FOR THE SYNTHESIS OF ACIDS* (**4**)</u>[36]

To a solution of the corresponding addition adduct **3** (0.5 mmol) in dry Et_2O (5 mL) at room temperature, under a nitrogen atmosphere, periodic acid (0.23 g, 1 mmol) was added. The reaction mixture was stirred until TLC analysis indicated that the reaction was complete. The solvent was decanted and the remaining white solid was washed twice with CH_2Cl_2. The crude product obtained after removing the solvent was directly purified by column chromatography (hexane/EtOAc 95:5 to EtOAc 100%) to afford the corresponding carboxylic acid and the recovered 3,3',5,5'-tetrakis(trifluoromethyl)benzophenone (80-84%).

(*R*)-4-Methyl-4-nitro-5-phenylpentanoic acid (4A)

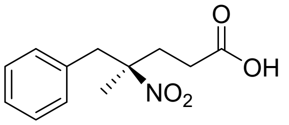

Prepared following the General Procedure starting from adduct **3Ac** (338 mg, 0.5 mmol). The product was isolated as a colorless oil (89 mg, 0.375 mmol, 75% yield). $[\alpha]_D^{25}$= –29.79 (c=0.26, CH_2Cl_2). ^1H NMR (400 MHz, $CDCl_3$) δ 7.34 – 7.27 (m, 3H), 7.12 – 7.05 (m, 2H), 3.35 (d, *J* = 13.9 Hz, 1H), 3.08 (d, *J* = 13.9 Hz, 1H), 2.51 (ddd, *J* = 13.2, 9.4, 4.7 Hz, 1H), 2.45 – 2.32 (m, 2H), 2.12 (ddd, *J* = 12.6, 9.9, 5.0 Hz, 1H), 1.50 (s, 3H). ^{13}C NMR (100 MHz, $CDCl_3$) δ 177.2, 134.1, 130.1, 128.7, 127.7, 90.9, 46.2, 33.7, 28.7, 21.3. HRMS (ESI-TOF) m/z: $[M+H]^+$ calcd for $C_{12}H_{16}NO_4$ 238.1079; found 238.1076. The enantiomer ratio (96:4) was determined by chiral HPLC of the corresponding methyl ester **6A**.

(*R*)-5-(4-Chlorophenyl)-4-methyl-4-nitropentanoic acid (4C)

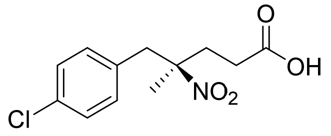

Prepared following General Procedure starting from adduct **3Cc** (355 mg, 0.5 mmol). The product was isolated as a white solid (109 mg, 0.4 mmol, 80% yield). m.p.: 106-108 ˚C. $[\alpha]_D^{25}$= –2.56 (c=1.05, CH_2Cl_2). ^1H NMR (400 MHz, $CDCl_3$) δ 7.28 (d, *J* = 8.4 Hz, 2H), 7.02 (d, *J* = 8.4 Hz, 2H), 3.33 (d, *J* = 14.0 Hz, 1H), 3.04 (d, *J* = 14.0 Hz, 1H), 2.58 – 2.28 (m, 3H), 2.16 – 2.06 (m, 1H), 1.49 (s, 3H). ^{13}C NMR (100 MHz, $CDCl_3$) δ 177.5, 133.9, 132.5, 131.4, 128.9, 90.6, 45.5, 33.7, 28.7, 21.2. HRMS (ESI-TOF) m/z: $[M+H]^+$ calcd for $C_{12}H_{15}ClNO_4$ [272.0690; found 272.0692.

[36] García, J.M.; Maestro, M.A.; Oiarbide, M.; Odriozola, J.M.; Razkin, J.; Palomo, C. *Org. Lett.* **2009**, *11* , 3826-3829.

(*R*)-4-Methyl-4-nitro-5-(*m*-tolyl)pentanoic acid (4G)

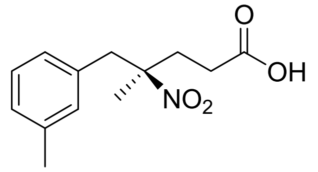

Prepared following the General Procedure starting from adduct **3Gc** (345 mg, 0.5 mmol). The product was isolated as a white solid (97 mg, 0.38 mmol, 77% yield). m.p.: 89-92 °C. $[\alpha]_D^{25}$= –23.0 (c=0.4, CH_2Cl_2). ^{1}H NMR (400 MHz, $CDCl_3$) δ 7.21 – 7.15 (m, 1H), 7.09 (d, *J* = 7.4 Hz, 1H), 6.89 (s, 1H), 6.88 (d, *J* = 6.2 Hz, 1H), 3.31 (d, *J* = 13.9 Hz, 1H), 3.04 (d, *J* = 13.9 Hz, 1H), 2.50 (ddd, *J* = 12.4, 8.9, 4.5 Hz, 1H), 2.44 – 2.37 (m, 2H), 2.32 (s, 3H), 2.12 (ddd, *J* = 12.3, 9.8, 4.8 Hz, 1H), 1.50 (s, 3H). ^{13}C NMR (100 MHz, $CDCl_3$) δ 176.3, 138.3, 134.1, 130.8, 128.5, 128.5, 127.1, 90.9, 46.2, 33.7, 28.5, 21.4, 21.3. HRMS (ESI-TOF) m/z: $[M+H]^+$ calcd for $C_{13}H_{18}NO_4$ 252.1236; found 252.1233.

(*R*)-4-Methyl-5-(naphthalen-2-yl)-4-nitropentanoic acid (4L)

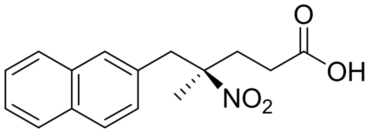

Prepared following the General Procedure starting from adduct **3Lc** (363 mg, 0.5 mmol). The product was isolated as a white solid (113 mg, 0.39 mmol, 79% yield). m.p.: 174-176 °C. $[\alpha]_D^{25}$= –25.2 (c=0.6, CH_2Cl_2). ^{1}H NMR (400 MHz, $CDCl_3$) δ 7.86 – 7.80 (m, 3H), 7.59 (s, 1H), 7.50 (m, 2H), 7.22 (dd, *J* = 8.4 Hz, 1.8 Hz, 1H), 3.56 (d, *J* = 13.9 Hz, 1H), 3.27 (d, *J* = 13.9 Hz, 1H), 2.60 (m, 1H), 2.44 (m, 2H), 1.19 (ddd, *J* = 14.0, 10.0, 5.8 Hz, 1H), 1.57 (s, 3H). ^{13}C NMR (100 MHz, $CDCl_3$) δ 177.2, 133.3, 132.7, 131.6, 129.2, 128.3, 127.9, 127.8, 127.6, 126.4, 126.2, 91.0, 46.4, 33.8, 28.7, 21.4. HRMS (ESI-TOF) m/z: $[M+H]^+$ calcd for $C_{16}H_{18}NO_4$ 288.1236; found 288.1232.

GENERAL PROCEDURE FOR THE SYNTHESIS OF ESTERS (**6**)[36]

Trimethylsilyl diazomethane (2M solution in hexanes, 0.274 mL, 0.55 mmol) was added dropwise to a solution of the corresponding acid **4** (0.5 mmol) in benzene (10 mL) and methanol (1 mL) at room temperature. After stirring overnight, solvents were removed under reduced pressure to afford the corresponding ester.

Methyl (*R*)-4-methyl-4-nitro-5-phenylpentanoate (6A)

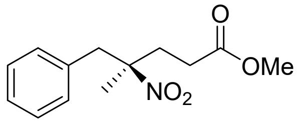

Prepared following General Procedure starting from acid **4A** (119 mg, 0.5 mmol). The product was isolated as a colorless oil (126 mg, 0.5 mmol, quantitative). The enantiomeric ratio (96:4) was determined by chiral HPLC analysis (Phenomenex Lux 3µm Cellulose 1, hexane/isopropanol = 95/5, flow rate = 1.0 mL/min, λ = 202 nm) t_R = 16.6 min (major), t_R = 17.8 min (minor). $[\alpha]_D^{25}$ = –25.2 (c=0.6, CH_2Cl_2). ^{1}H NMR (400 MHz, $CDCl_3$) δ 7.33 – 7.26 (m, 3H), 7.12 – 7.04 (m, 2H), 3.69 (s, 3H), 3.35 (d, *J* = 13.9 Hz, 1H), 3.06 (d, *J* = 13.9 Hz, 1H), 2.56 – 2.44 (m, 1H), 2.43 – 2.25 (m, 2H), 2.12 (m, 1H), 1.48 (s, 3H). ^{13}C NMR (100 MHz, $CDCl_3$) δ 172.6, 134.2, 130.1, 128.6, 127.7, 91.0, 52.0, 46.3, 34.1, 28.8, 21.2. HRMS (ESI-TOF) m/z: $[M+Na]^+$ calcd for $C_{13}H_{17}NO_4Na$ 274.1055; found 274.1063.

Methyl (*R*)-5-(4-chlorophenyl)-4-methyl-4-nitropentanoate (6C)

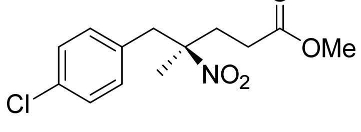

Prepared following General Procedure starting from acid **4C** (136 mg, 0.5 mmol). The product was isolated as a colorless oil (121 mg, 0.42 mmol, 85% yield). $[\alpha]_D^{25}$= –2.19 (c=0.97, CH_2Cl_2). ^{1}H NMR (400 MHz, $CDCl_3$) δ 7.29 (d, *J* = 8.4 Hz, 2H), 7.04 (d, *J* = 8.4 Hz, 2H), 3.71 (s, 3H), 3.36 (d, *J* = 14.0 Hz, 1H), 3.04 (d, *J* = 14.0 Hz, 1H), 2.60 – 2.46 (m, 1H), 2.46 – 2.27 (m, 2H), 2.21 – 2.08

(m, 1H), 1.49 (s, 3H). ^{13}C NMR (100 MHz, $CDCl_3$) δ 172.5, 133.8, 132.6, 131.4, 128.8, 90.8, 52.0, 45.5, 34.2, 28.8, 21.1. HRMS (ESI-TOF) m/z: $[M+Na]^+$ calcd for $C_{13}H_{16}ClNO_4Na$ 308.0666; found 308.0668.

Methyl (*R*)-4-methyl-4-nitro-5-(*m*-tolyl)pentanoate (6G)

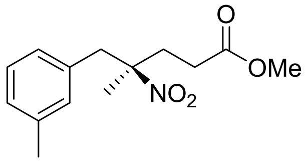

Prepared following the General Procedure starting from acid **4G** (126 mg, 0.5 mmol). The product was isolated as a colorless oil (133 mg, 0.5 mmol, quantitative). $[\alpha]_D^{25}$= –17.00 (c=0.54, CH_2Cl_2). ^{1}H NMR (400 MHz, $CDCl_3$) δ 7.18 (t, *J* = 7.9 Hz, 1H), 7.08 (d, *J* = 7.5 Hz, 1H), 6.89 (s, 1H), 6.88 (d, *J* = 6.5 Hz, 1H), 3.69 (s, 3H), 3.31 (d, *J* = 13.9 Hz, 1H), 3.02 (d, *J* = 13.9 Hz, 1H), 2.56 – 2.45 (m, 1H), 2.43 – 2.26 (m, 2H), 2.32 (s, 3H), 2.12 (m, 1H), 1.48 (s, 3H). ^{13}C NMR (100 MHz, $CDCl_3$) δ 172.6, 138.2, 134.1, 130.8, 128.5, 128.4, 127.1, 91.0, 52.0, 46.2, 34.1, 28.9, 21.4, 21.2. HRMS (ESI-TOF) m/z: $[M+Na]^+$ calcd for $C_{14}H_{19}NO_4Na$ 288.1206; found 288.1214.

SYNTHESIS OF ALDEHYDE (**5**)

(*R*)-4-Methyl-5-(naphthalen-2-yl)-4-nitropentanal[36]

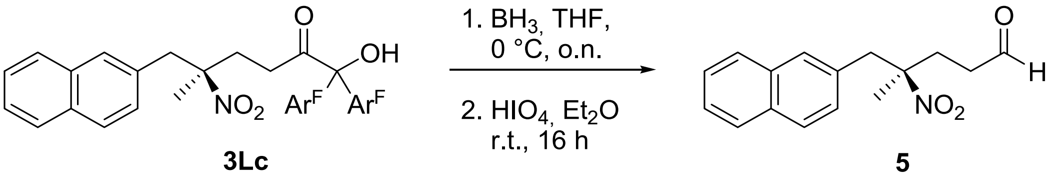

In a round bottom flask, under nitrogen atmosphere, to a solution of 1,1-bis(3,5-bis(trifluoromethyl)phenyl)-1-hydroxy-5-methyl-6-(naphthalen-2-yl)-5-nitrohexan-2-one **(3Lc)** (246.7 mg, 0.34 mmol) in THF (0.7 mL), cooled to 0 °C (ice bath), a solution of borane in THF (1M sol., 0.7 mL, 0.7 mmol) was added dropwise keeping the temperature at 0 °C. The mixture was stirred overnight at the same temperature. While keeping cool (0 °C), methanol (2 mL) was added and then the mixture was allowed to reach room temperature and stirred for additional 30 minutes. Evaporation of solvent afforded the crude diol compound which was purified by column chromatography (hexane-EtOAc 90:10). In a round bottom flask, under nitrogen atmosphere at room temperature, the crude diol was dissolved in dry diethyl ether (4 mL) and periodic acid (130.5 mg, 0.68 mmol) was added. The reaction mixture was stirred at room temperature for 16 h. The solvent was decanted and the remaining white solid was washed twice with CH_2Cl_2. The crude product obtained after removing the solvent was directly purified by column chromatography (hexane-EtOAc 20:1) to afford the aldehyde as a colorless oil (66.4 mg, 0.24mmol, 72%). $[\alpha]_D^{25}$= -0,74 (c = 0,65, CH_2Cl_2). ^{1}H NMR (400 MHz, $CDCl_3$) δ 9.81 (s, 1H), 7.91 – 7.74 (m, 3H), 7.59 (s, 1H), 7.53 – 7.47 (m, 2H), 7.22 (m, 1H), 3.56 (d, *J* = 13.9 Hz, 1H), 3.28 (d, *J* = 13.9 Hz, 1H), 2.63 – 2.47 (m, 3H), 2.26 – 2.12 (m, 1H), 1.54 (s, 3H). ^{13}C NMR (100 MHz, $CDCl_3$) δ 199.5, 133.3, 132.7, 131.7, 129.1, 128.3, 127.9, 127.8, 127.6, 126.4, 126.2, 91.1, 46.5, 38.7, 31.2, 21.5. HRMS (ESI-TOF) m/z: $[M+H]^+$ calcd for $C_{16}H_{18}NO_3$ 272.1287; found 272.1282.

GENERAL PROCEDURE FOR THE SYNTHESIS OF LACTAMS (**7**)[37]

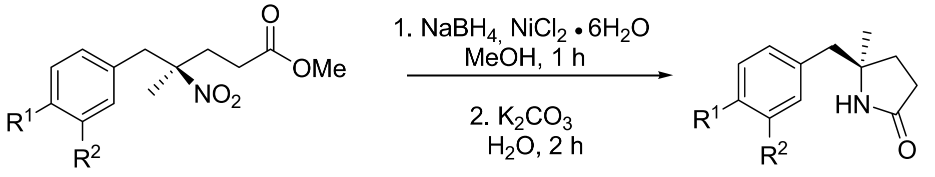

6C R^1: Cl, R^2: H
6G R^1: H, R^2: Me

7C R^1: Cl, R^2: H
7G R^1: H, R^2: Me

To a solution of the corresponding methyl ester **6** (0.2 mmol) and $NiCl_2·6H_2O$ (47.5 mg, 0.2 mmol) in MeOH (2 mL) cooled in an ice-water bath (0 °C), $NaBH_4$ (37.8 mg, 1 mmol) was slowly added. The resultant mixture was stirred at the same temperature for 1 h. The reaction was quenched by adding a solution of K_2CO_3 (112.2 mg, 0.8 mmol) in H_2O (1 mL) followed by stirring at room temperature for 2 h. The mixture was extracted with EtOAc (3x10 mL) and the organic layer was washed with brine, dried over anhydrous $MgSO_4$, filtered, and evaporated under reduced pressure. The residue was purified by flash column chromatography on silica gel to give the lactam **7**.

(*R*)-5-(4-Chlorobenzyl)-5-methylpyrrolidin-2-one (7C)

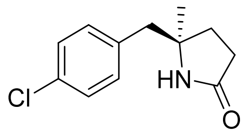

Prepared following General Procedure starting from ester **6C** (57 mg, 0.2 mmol). The product was isolated as a colorless oil (36.7 mg, 0.16 mmol, 82% yield). $[\alpha]_D^{25}$ = –8.4 (c=1.0, CH_2Cl_2). ^1H NMR (400 MHz, $CDCl_3$) δ 7.26 (d, *J* = 8.4 Hz, 2H), 7.10 (d, *J* = 8.4 Hz, 2H), 6.72 (s_b, 1H), 2.76 (d, *J* = 13.5 Hz, 1H), 2.72 (d, *J* = 13.5 Hz, 1H), 2.33 – 2.21 (m, 1H), 2.11 – 1.97 (m, 2H), 1.90 – 1.80 (m, 1H), 1.26 (s, 3H). ^{13}C NMR (100 MHz, $CDCl_3$) δ 177.4, 135.2, 132.9, 131.6, 128.5, 59.5, 47.1, 33.1, 30.5, 27.7. HRMS (ESI-TOF) m/z: $[M+H]^+$ calcd for $C_{12}H_{15}ClNO$ 224.0842; found 224.0840.

(*R*)-5-Methyl-5-(3-methylbenzyl)pyrrolidine-2-one (7G)

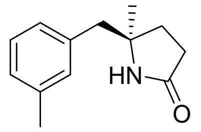

Prepared following General Procedure starting from ester **6G** (53.1 mg, 0.2 mmol). The product was isolated as a colorless oil (31.3 mg, 0.154 mmol, 77% yield). The enantiomeric ratio (94:6) was determined by chiral HPLC analysis (IA, hexane/isopropanol = 95/5, flow rate = 1.0 mL/min, λ = 202 nm) t_R = 14.4 min (major), t_R = 20.7 min (minor). $[\alpha]_D^{25}$ = –9.7 (c=1.2, CH_2Cl_2). ^1H NMR (400 MHz, $CDCl_3$) δ 7.19 (t, *J* = 7.6 Hz, 1H), 7.07 (d, *J* = 7.5 Hz, 1H), 6.97 (s, 1H), 6.96 (d, *J* = 7.9 Hz, 1H), 5.74 (s_b, 1H), 2.82 – 2.65 (m, 2H), 2.33 (s, 3H), 2.38 – 2.28 (m, 1H), 2.24 – 2.05 (m, 2H), 1.92 (m, 1H), 1.25 (s, 3H). ^{13}C NMR (100 MHz, $CDCl_3$) δ 176.9, 138.1, 136.6, 131.0, 128.3, 127.6, 127.3, 59.3, 47.9, 33.9, 30.4, 27.3, 21.4. HRMS (ESI-TOF) m/z: $[M+H]^+$ calcd for $C_{13}H_{18}NO$ 204.1383; found 204.1384.

[37] Xu, J.; Li, X.; Wu, J.; Dai, W-M. *Tetrahedron* **2014**, *70*, 3839-3846.

SYNTHESIS OF NITRILE (**9**)[38]

(*R*,*E*)-1,1-Bis(3,5-bis(trifluoromethyl)phenyl)-1-hydroxy-5-methyl-6-(naphthalen-2-yl)-5-nitrohexan-2-one oxime (8)

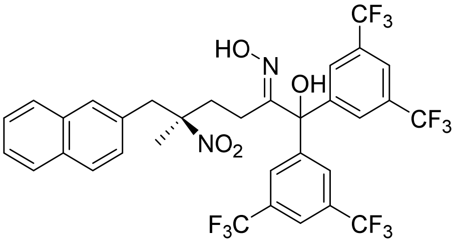

To a solution of adduct **3Lh** (417 mg, 0.58 mmol) and $NH_2OH·HCl$ (74 mg, 1.1 mmol) in EtOH (6 mL) under nitrogen atmosphere was added pyridine (115 mg, 1.45 mmol) and the mixture was heated at 90 ºC under stirring for 8 days. The crude product obtained after removing the solvent was directly purified through flash column cromatrography (Hexanes/EtOAc 95:5 to 85:15). The product was isolated as a white solid (301 mg, 0.41 mmol, 71% yield). m.p.: 172-173 ºC. $[\alpha]_D^{25}$= –23.04 (c=1, CH_2Cl_2). ^{1}H NMR (400 MHz, $CDCl_3$) δ 8.35 (s, 1H), 7.90 (d, *J* = 5.5 Hz, 2H), 7.79 (ddd, *J* = 16.2, 12.4, 7.7 Hz, 7H), 7.50 (dd, *J* = 5.7, 3.7 Hz, 3H), 7.10 (dd, *J* = 8.4, 1.5 Hz, 1H), 4.59 (s, 1H), 3.37 (d, *J* = 13.9 Hz, 1H), 3.12 (d, *J* = 13.9 Hz, 1H), 2.57 (td, *J* = 12.1, 4.3 Hz, 1H), 2.30 – 2.09 (m, 2H), 1.85 (dd, *J* = 12.2, 9.4 Hz, 1H), 1.49 (s, 3H). ^{13}C NMR (100 MHz, $CDCl_3$) δ 160.0, 144.5 (2s, 2C), 133.2, 132.6, 132.1 (2q, *J* = 33.7 Hz, 2C), 131.4, 129.0, 128.2, 127.8, 127.7, 127.6, 126.4, 126.2, 123.0 (q, *J* = 272.8 Hz), 122.7 (m), 91.4, 80.9, 46.4, 34.5, 22.0, 21.2. HRMS (ESI-TOF) m/z: $[M+H]^+$ calcd for $C_{33}H_{25}F_{12}N_2O_4$ 741.1623; found 741.1620.

(*R*)-4-Methyl-5-(naphthalen-2-yl)-4-nitropentanenitrile (9)

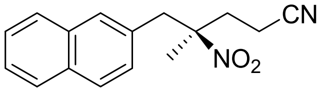

To a solution of the oxime (**8**) (301 mg, 0.41 mmol) and NEt_3 (137 mg, 1.35 mmol) in CH_2Cl_2 (4.1 mL) under inert atmosphere was added acetyl chloride (97 mg, 1.23 mmol) and the mixture was stirred at room temperature overnight. The solvent was evaporated under reduced pressure and the crude product was purified through flash column chromatography (Hex/AcOEt 95:5 to 85:15). The product was isolated as a colorless solid (93.3 mg, 0.35 mmol, 85% yield). The enantiomeric ratio (95:5) was determined by chiral HPLC analysis (OD-H, hexane/isopropanol = 90/10, flow rate = 1.0 mL/min, λ = 202 nm) t_R = 44.5 min (major), t_R = 48.9 min (minor). m.p.: 133-134 ºC. $[\alpha]_D^{25}$= –16.94 (c=1.1, CH_2Cl_2). ^{1}H NMR (400 MHz, $CDCl_3$) δ 7.86 – 7.78 (m, 3H), 7.56 (s, 1H), 7.53 – 7.46 (m, 2H), 7.18 (dd, *J* = 8.4, 1.7 Hz, 1H), 3.47 (d, *J* = 13.9 Hz, 1H), 3.31 (d, *J* = 13.9 Hz, 1H), 2.68 – 2.55 (m, 1H), 2.41 (dd, *J* = 8.5, 7.0 Hz, 2H), 2.24 – 2.10 (m, 1H), 1.62 (s, 3H). ^{13}C NMR (100 MHz, $CDCl_3$) δ 133.27, 132.8, 130.9, 129.2, 128.6, 127.8, 127.7, 127.6, 126.6, 126.4, 118.3, 90.5, 46.5, 34.3, 21.5, 12.7. HRMS (ESI-TOF) m/z: $[M+H]^+$ calcd for $C_{16}H_{17}N_2O_2$ 269.1290; found 269.1294.

[38] Adapted from: Zhang, X.; Xue, X.; Gu, Z. *Org. Lett.* **2023**, *25*, 3602 – 3606.

6. Representative NMR spectra

^{1}H NMR (300 MHz, $CDCl_3$) of compound **C8**:

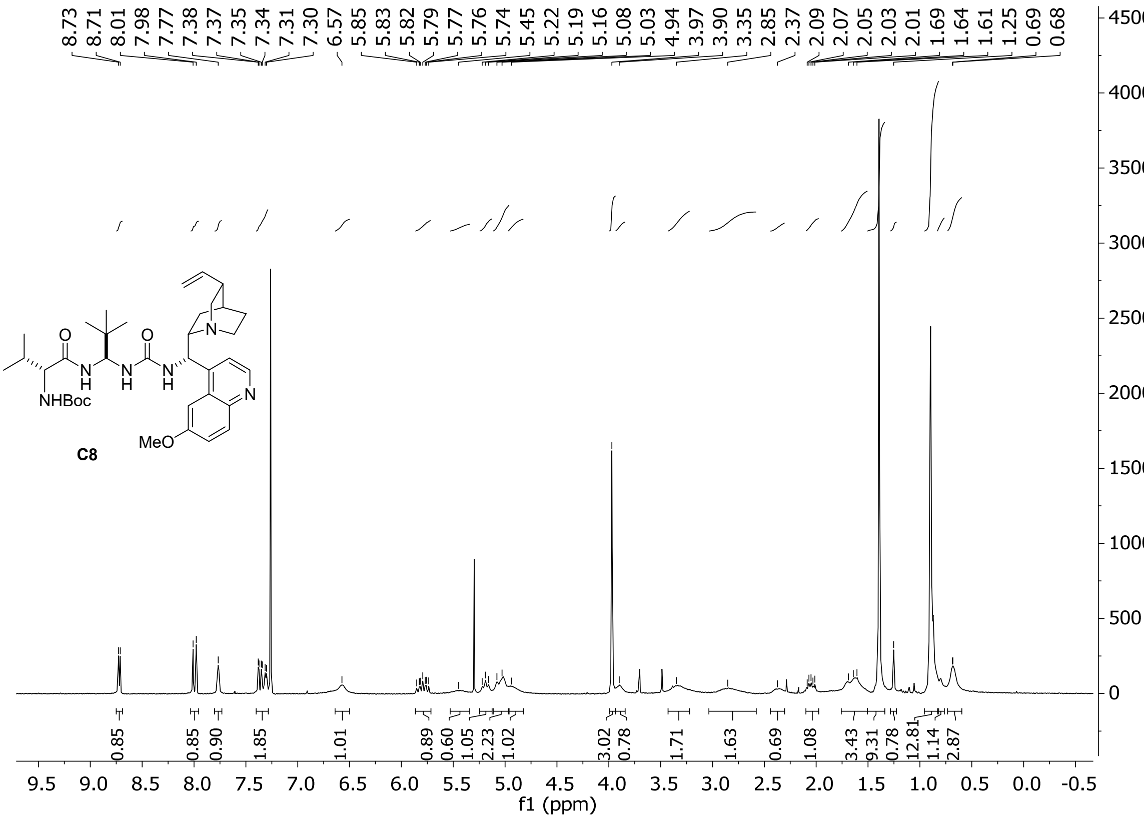

^{13}C NMR (75 MHz, $CDCl_3$) of compound **C8**:

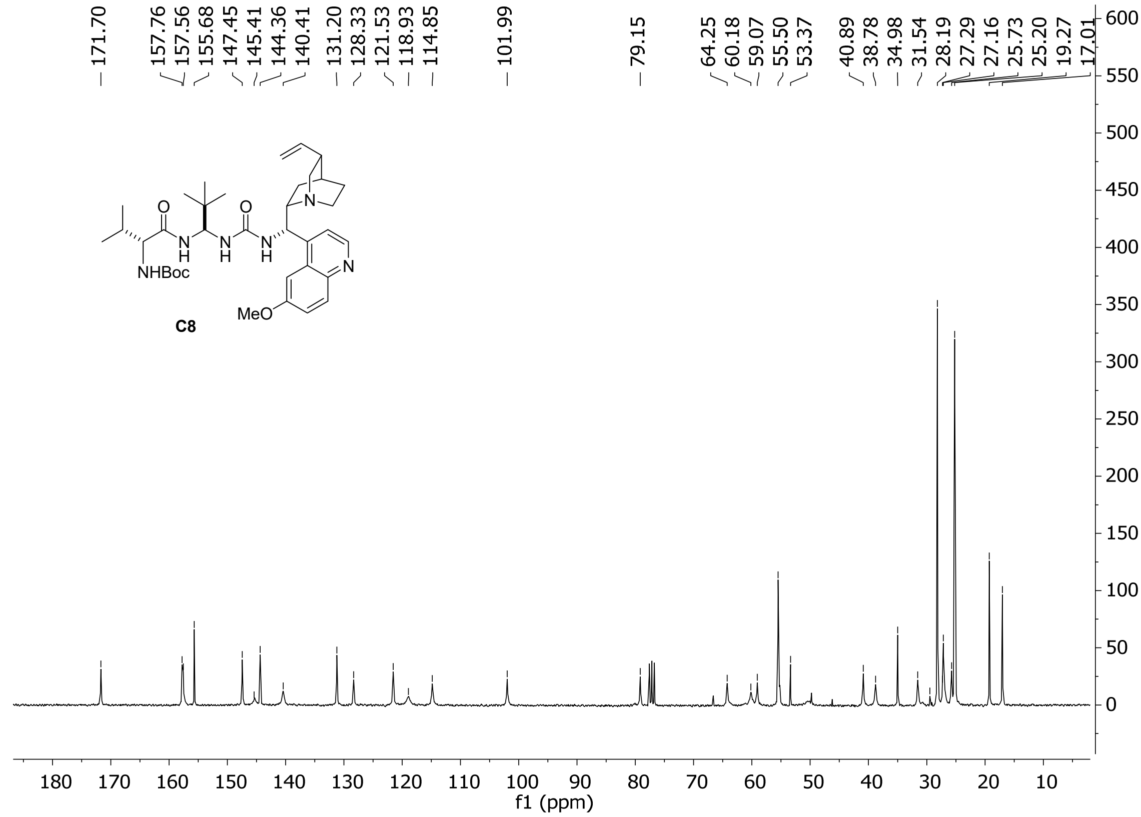

^{1}H NMR (300 MHz, CD_3OD) of compound **C9**:

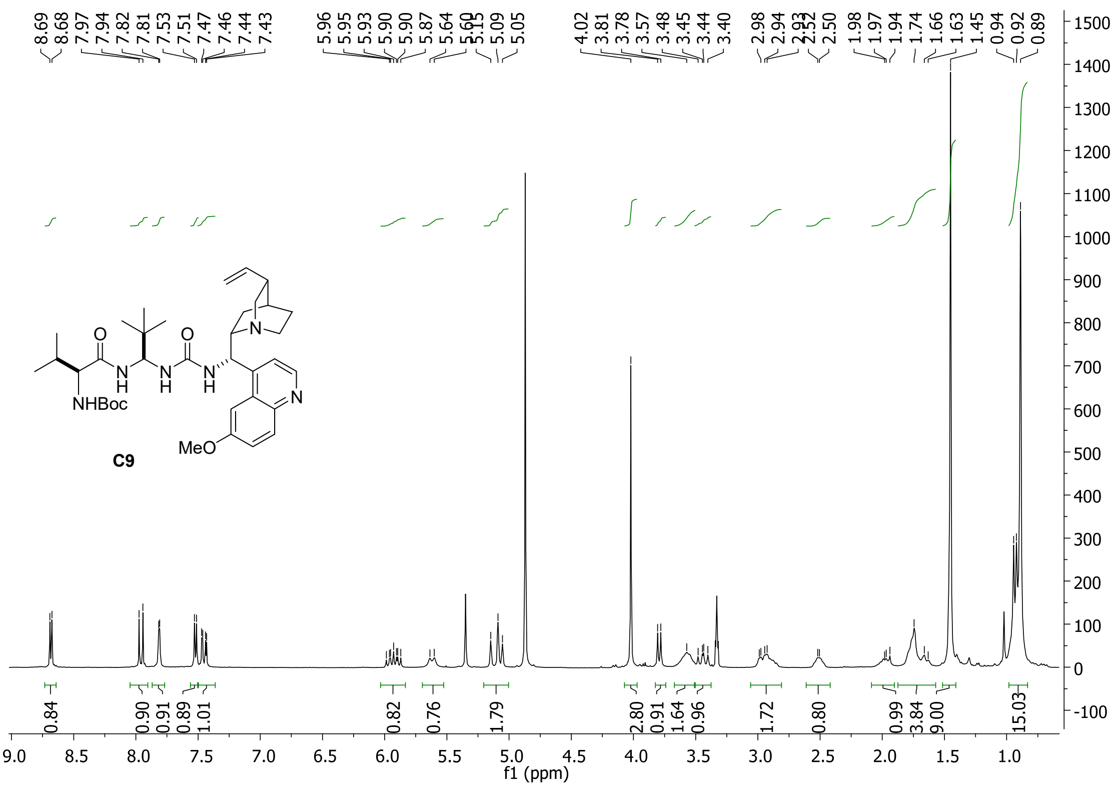

^{13}C NMR (75 MHz, CD_3OD) of compound **C9**:

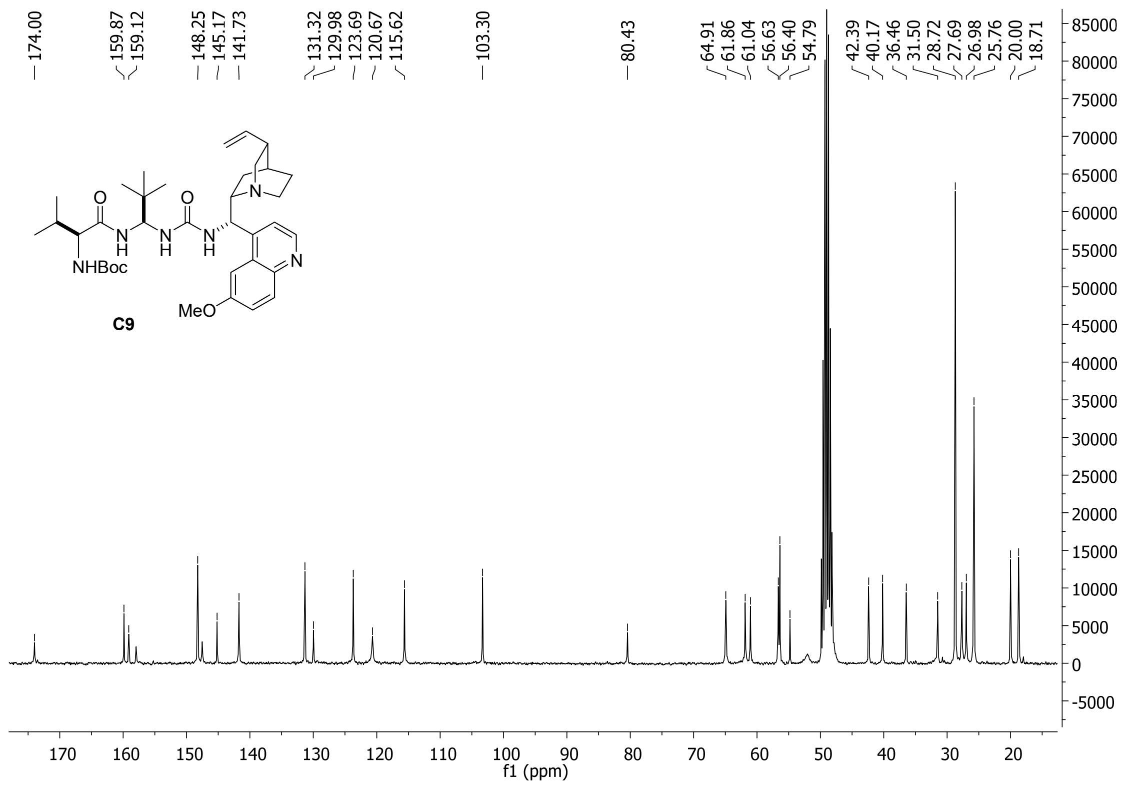

^{1}H NMR (300 MHz, $CDCl_3$) of compound **C10**:

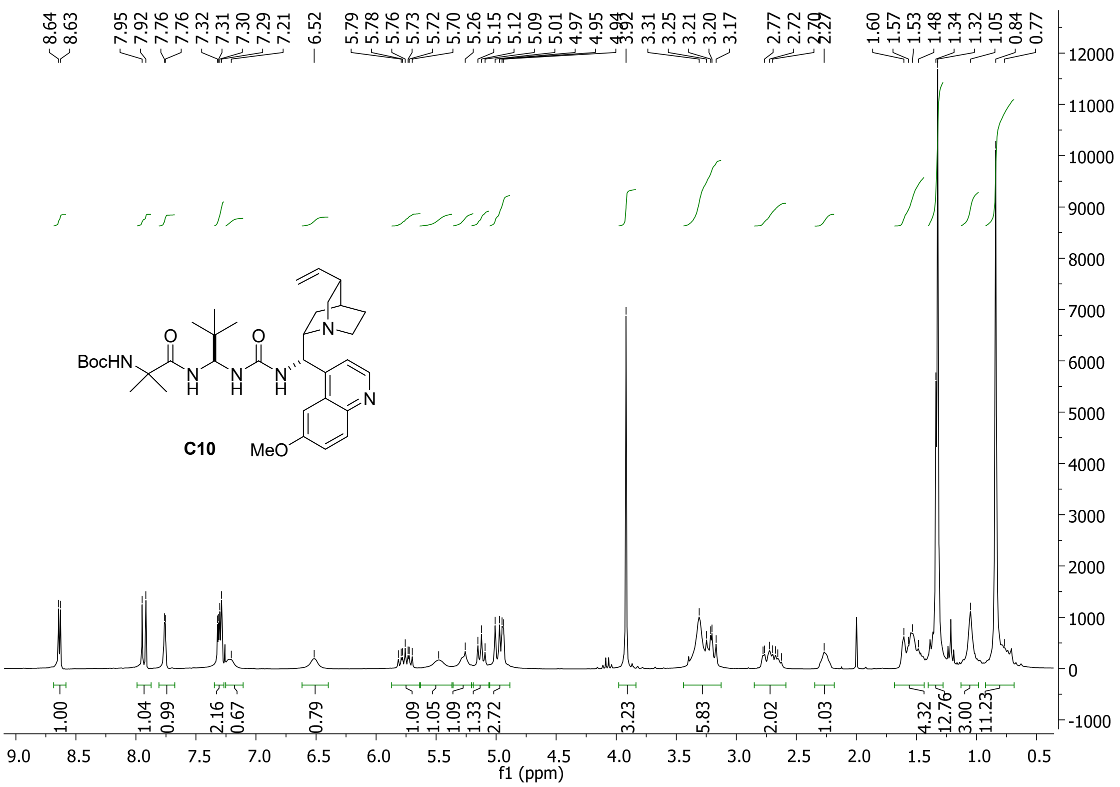

^{13}C NMR (75 MHz, $CDCl_3$) of compound **C10**:

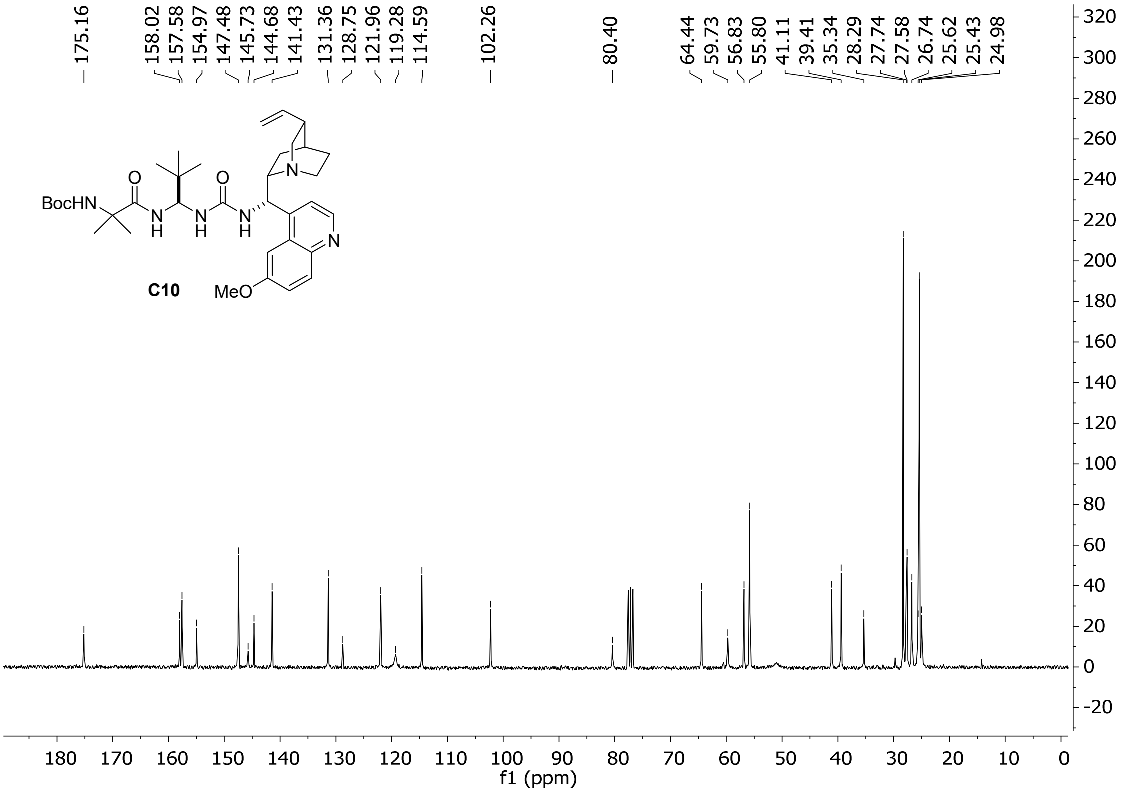

^{1}H NMR (300 MHz, $CDCl_3$) of compound **C11**:

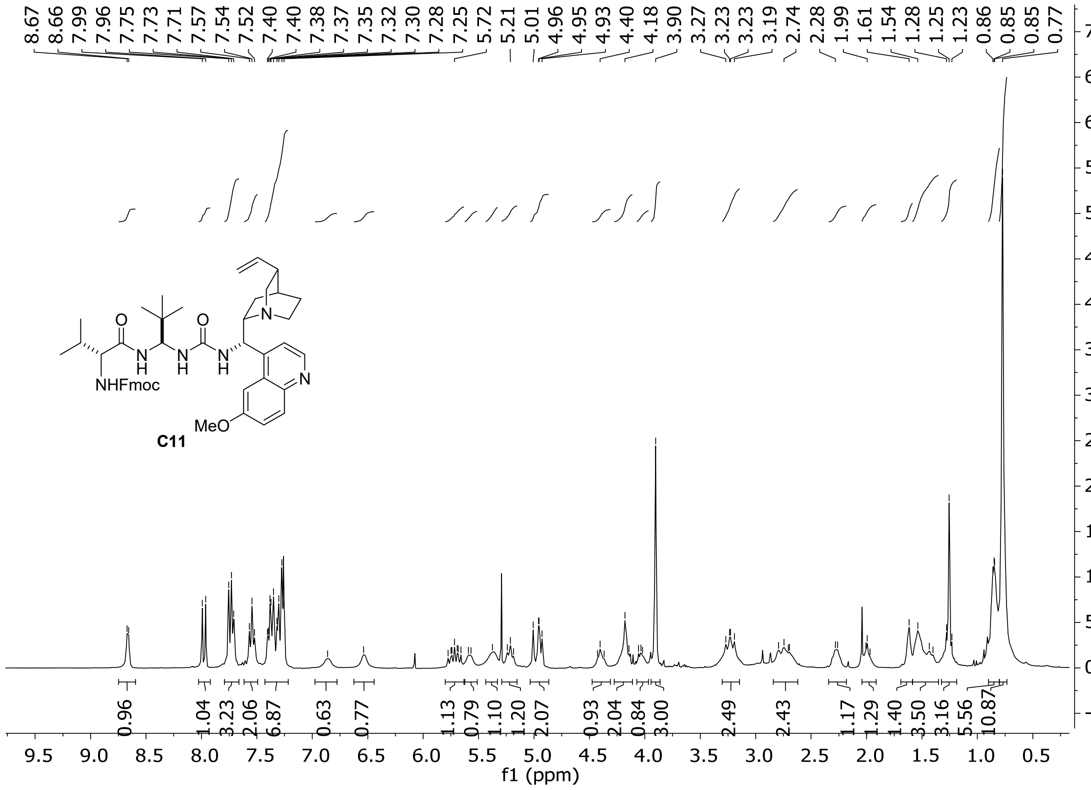

^{13}C NMR (75 MHz, $CDCl_3$) of compound **C11**:

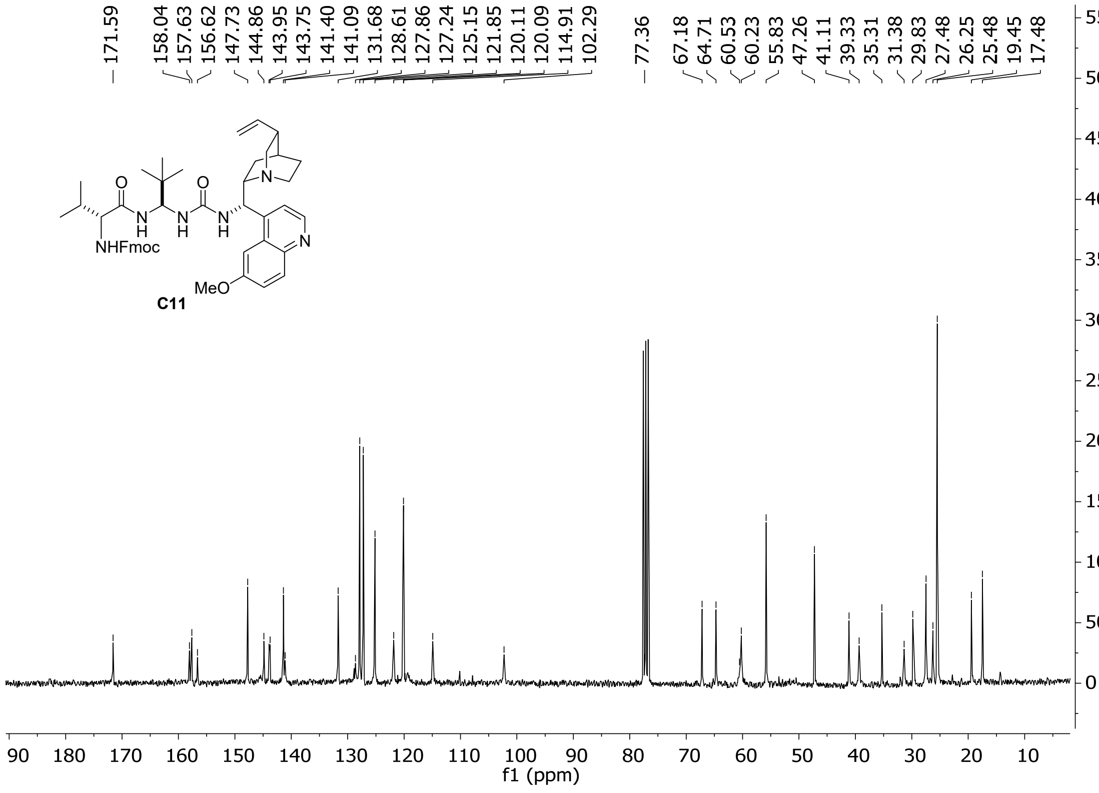

^{1}H NMR (300 MHz, $CDCl_3$) of compound **C12**:

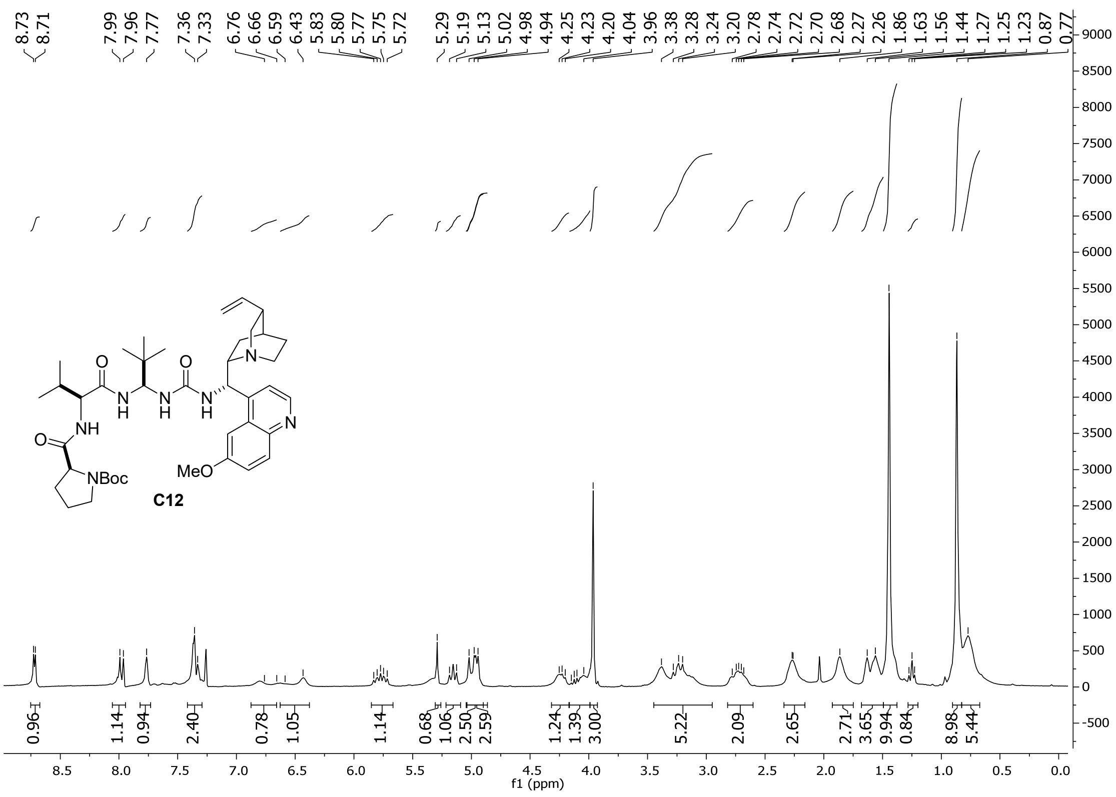

^{13}C NMR (75 MHz, $CDCl_3$) of compound **C12**:

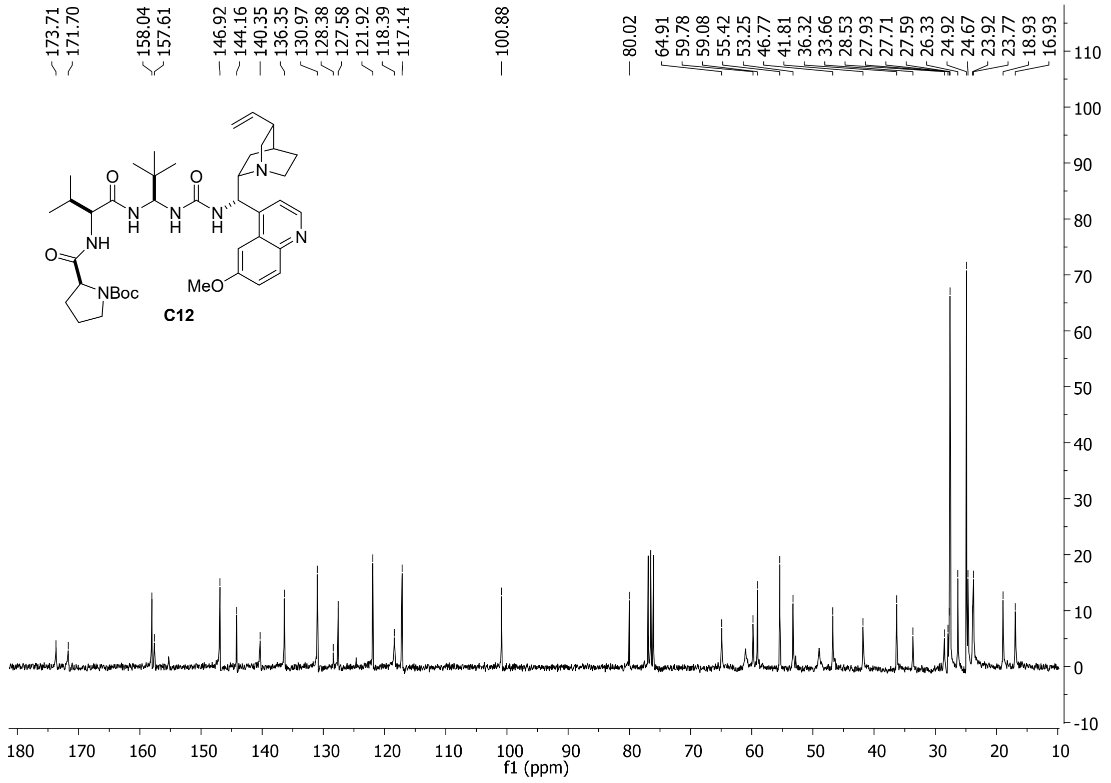

^{1}H NMR (300 MHz, $CDCl_3$) of compound **C13**:

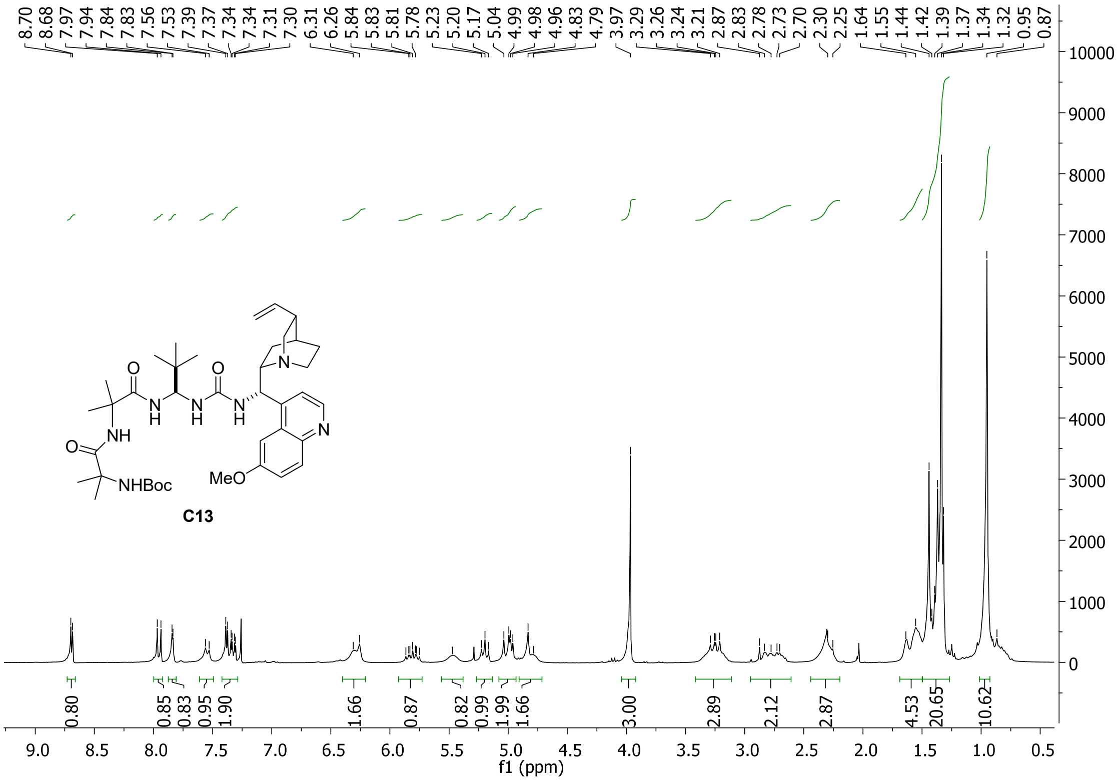

^{13}C NMR (75 MHz, $CDCl_3$) of compound **C13**:

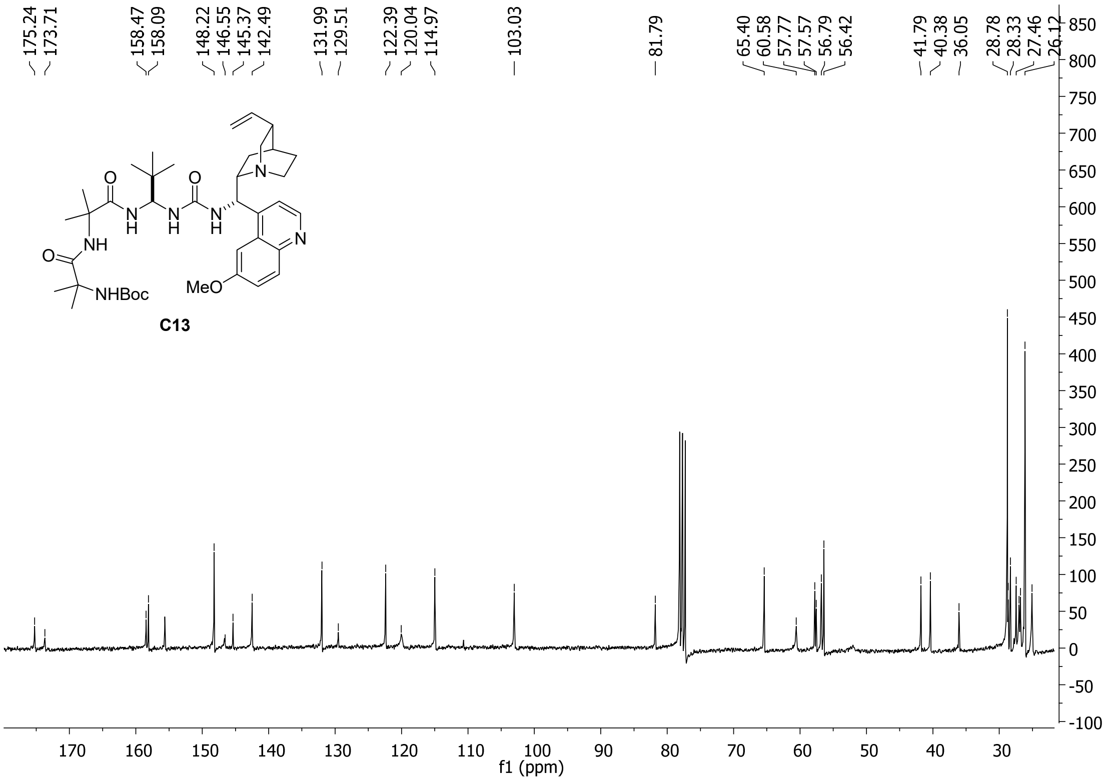

^{1}H NMR (400 MHz, DMSO-d$_6$, 70 °C) of compound **C15**:

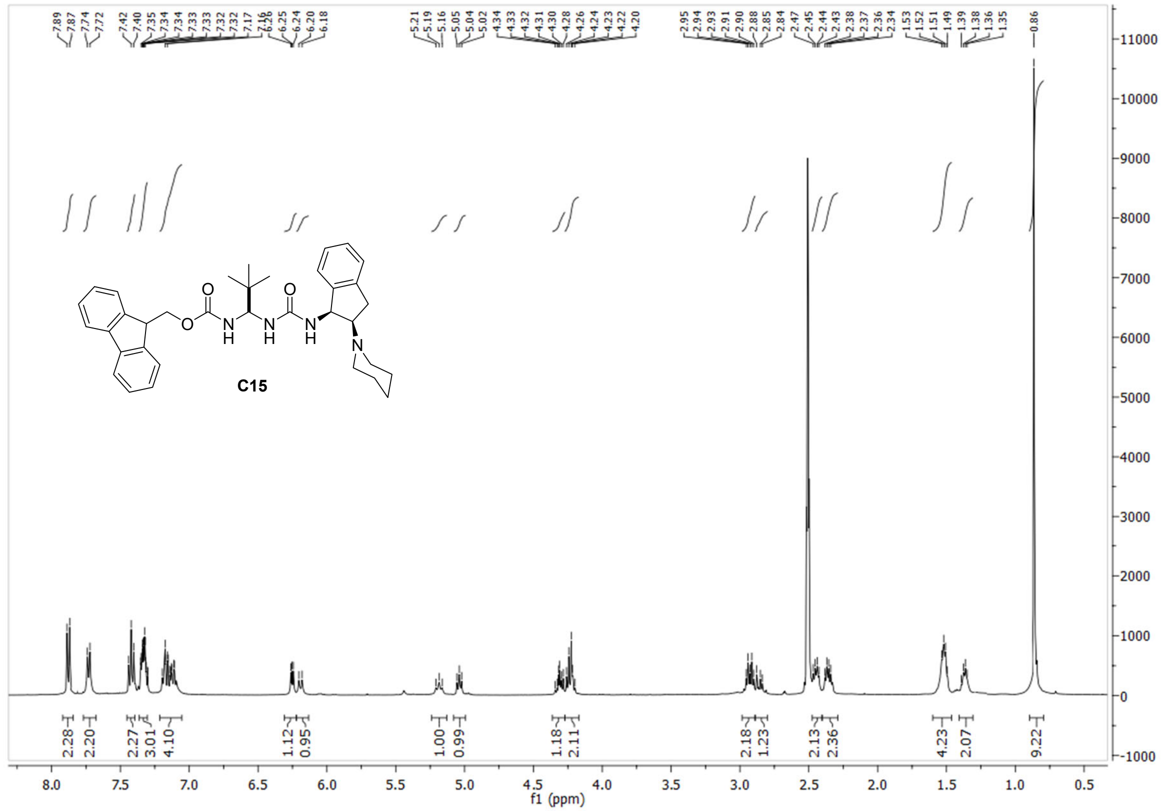

^{13}C NMR (100 MHz, DMSO-d$_6$, 70 °C) of compound **C15**:

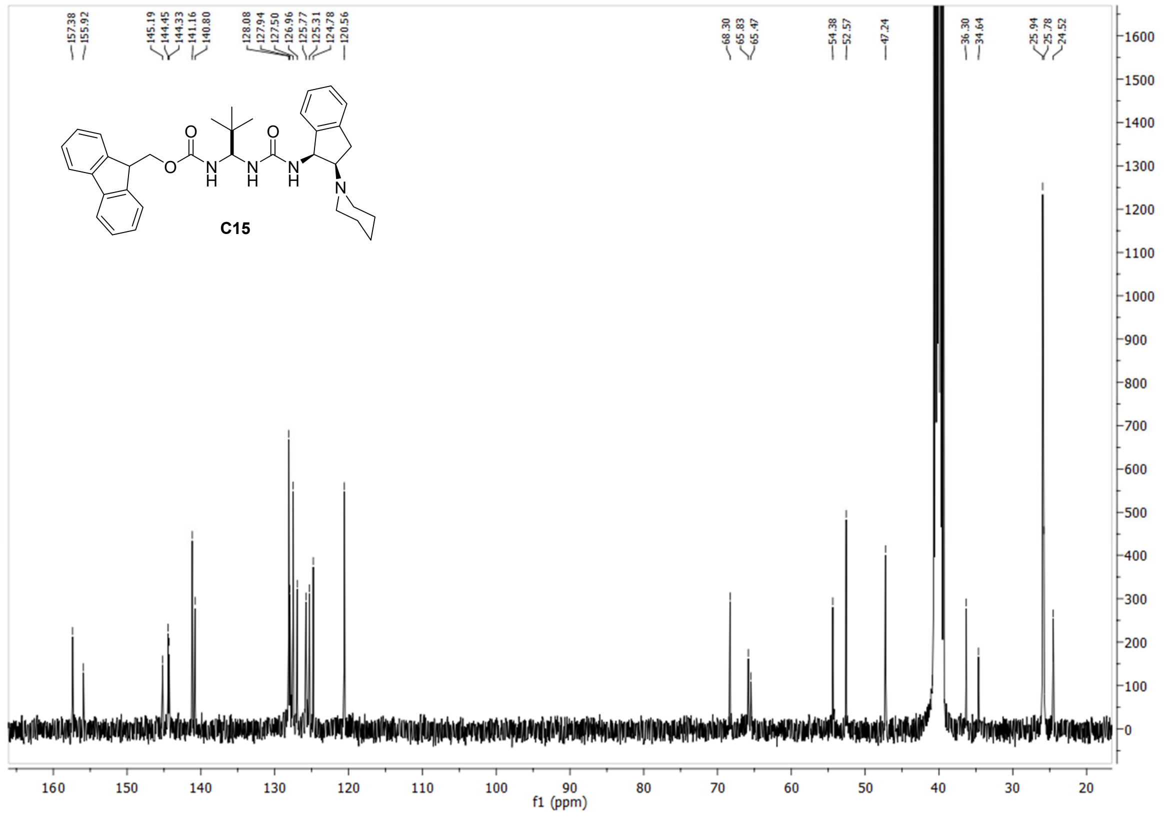

^{1}H NMR (400 MHz, $CDCl_3$) of compound **1A**:

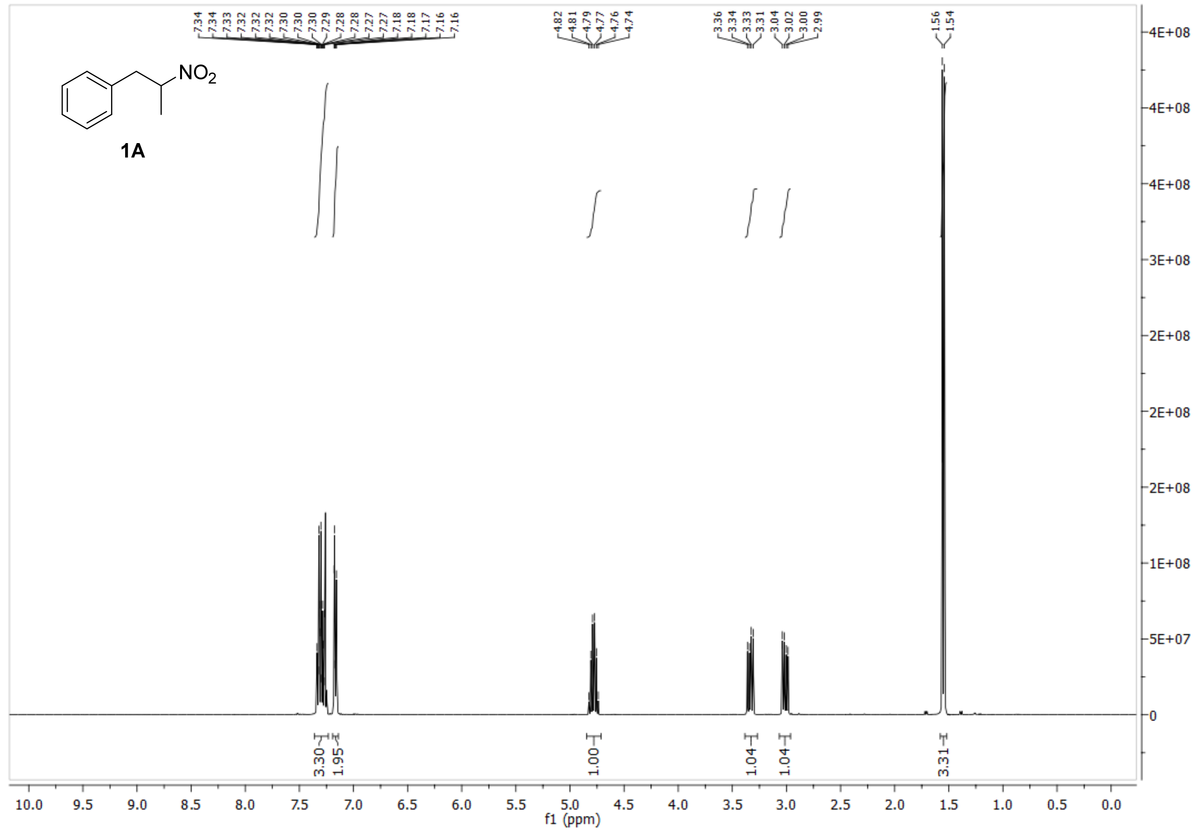

^{13}C NMR (100 MHz, $CDCl_3$) of compound **1A**:

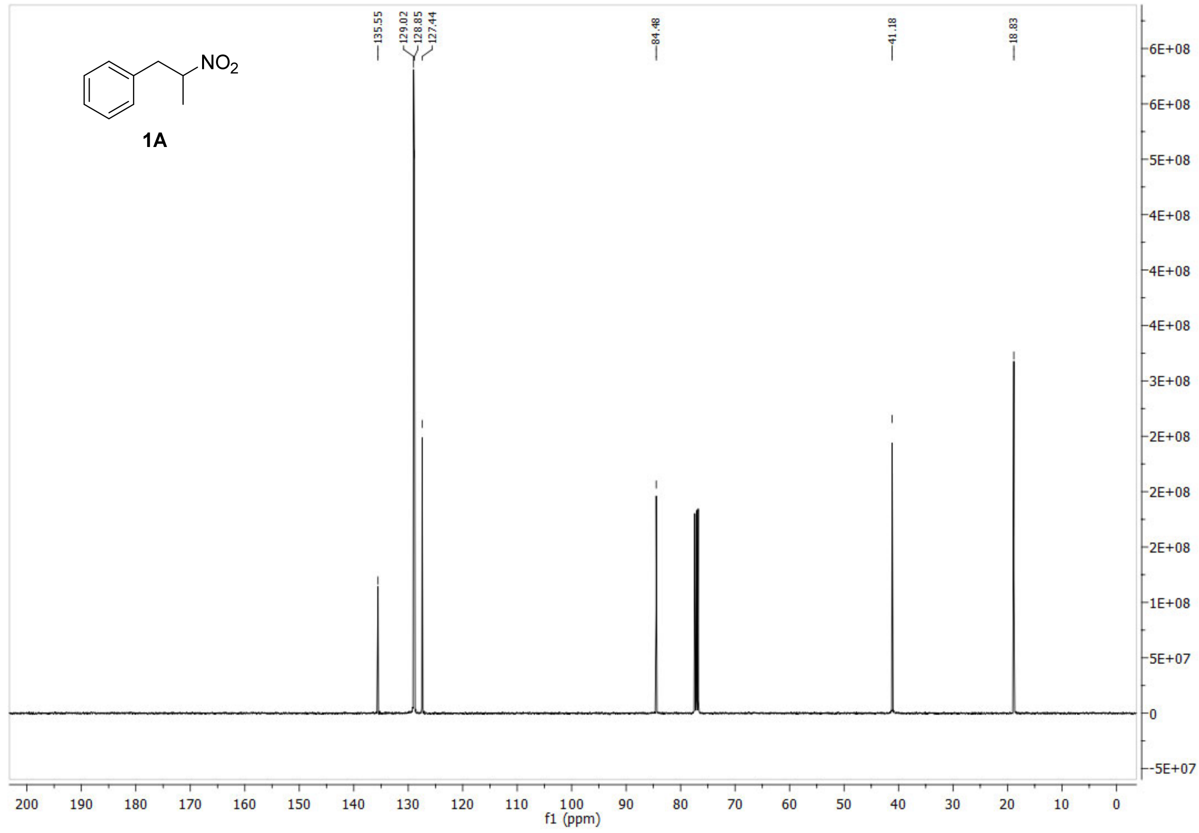

^{1}H NMR (400 MHz, $CDCl_3$) of compound **1B**:

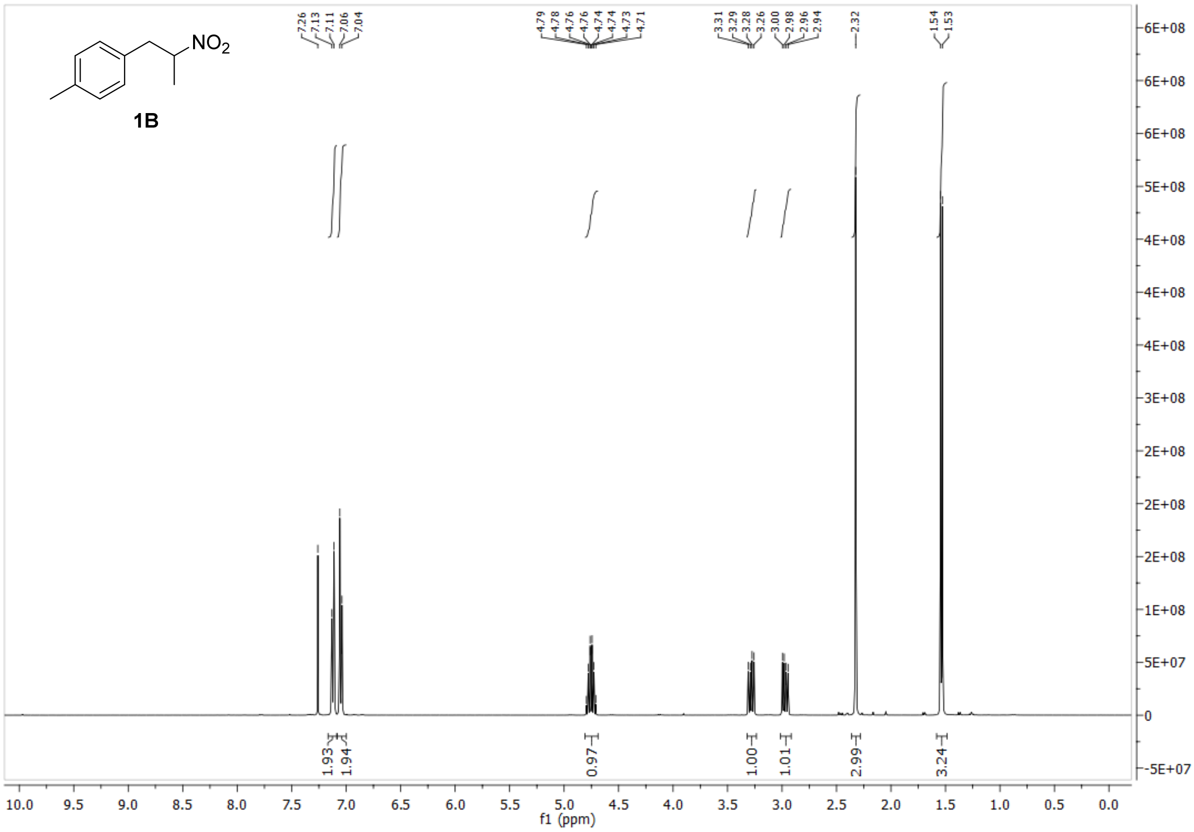

^{13}C NMR (100 MHz, $CDCl_3$) of compound **1B**:

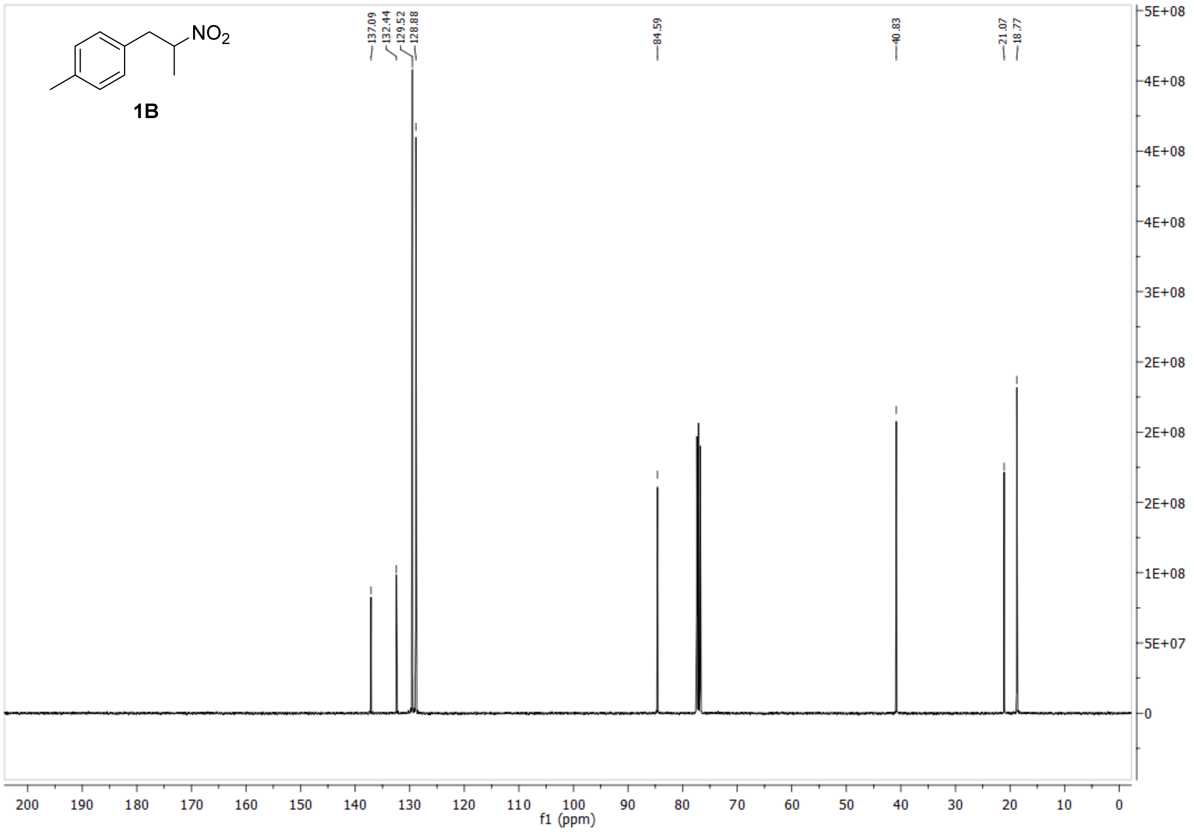

^{1}H NMR (400 MHz, $CDCl_3$) of compound **1C**:

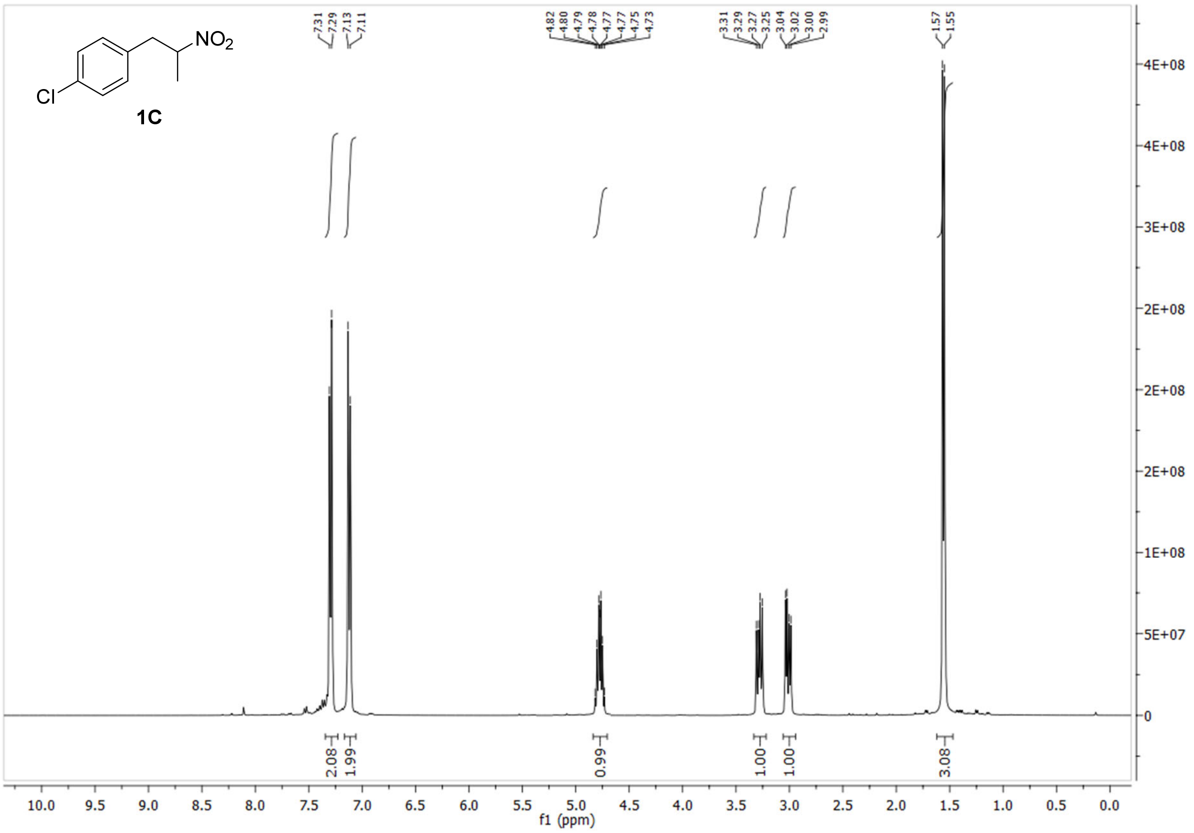

^{13}C NMR (100 MHz, $CDCl_3$) of compound **1C**:

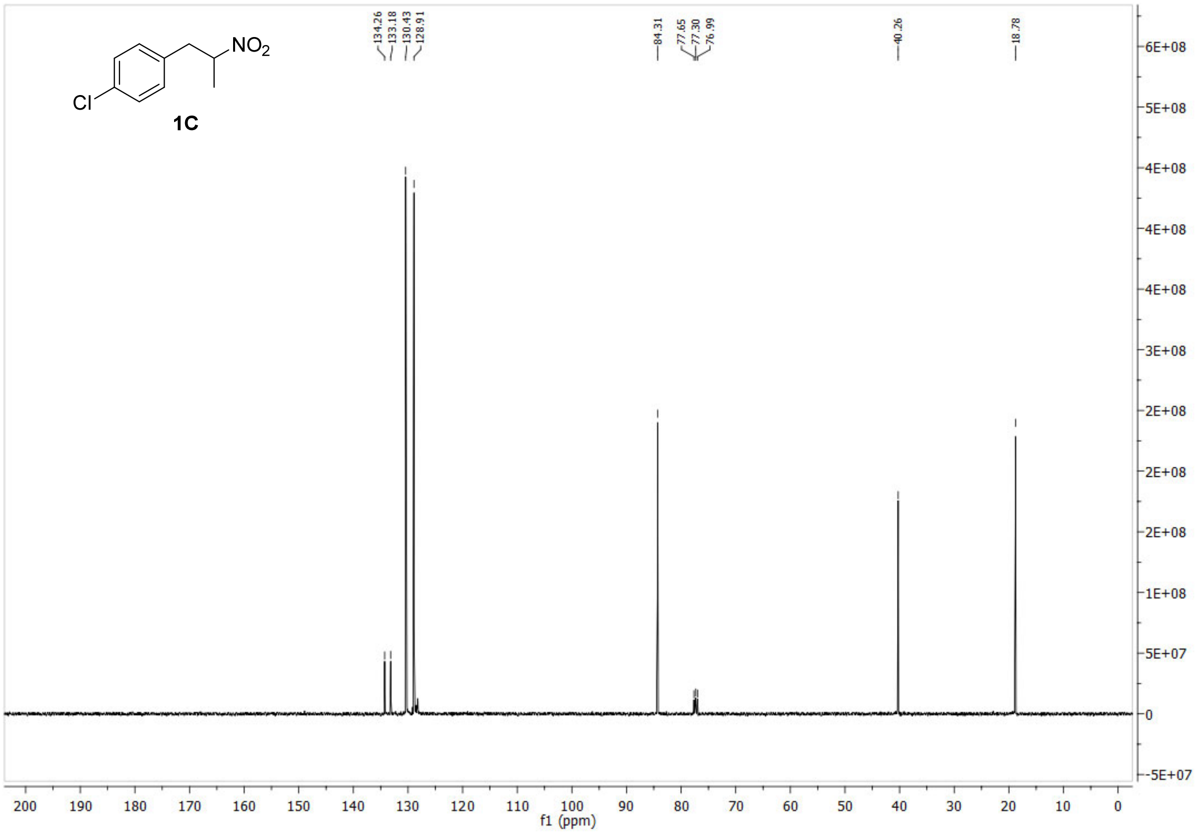

^{1}H NMR (400 MHz, $CDCl_3$) of compound **1D**:

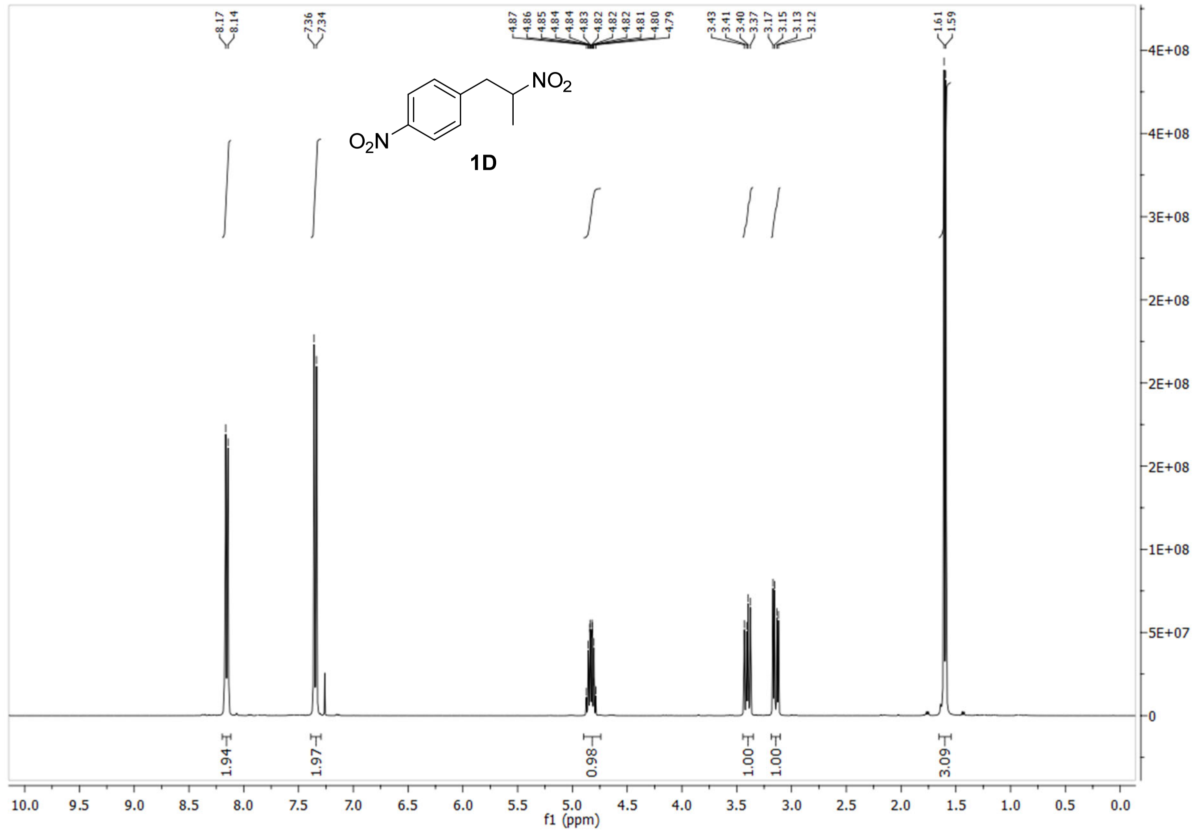

^{13}C NMR (100 MHz, $CDCl_3$) of compound **1D**:

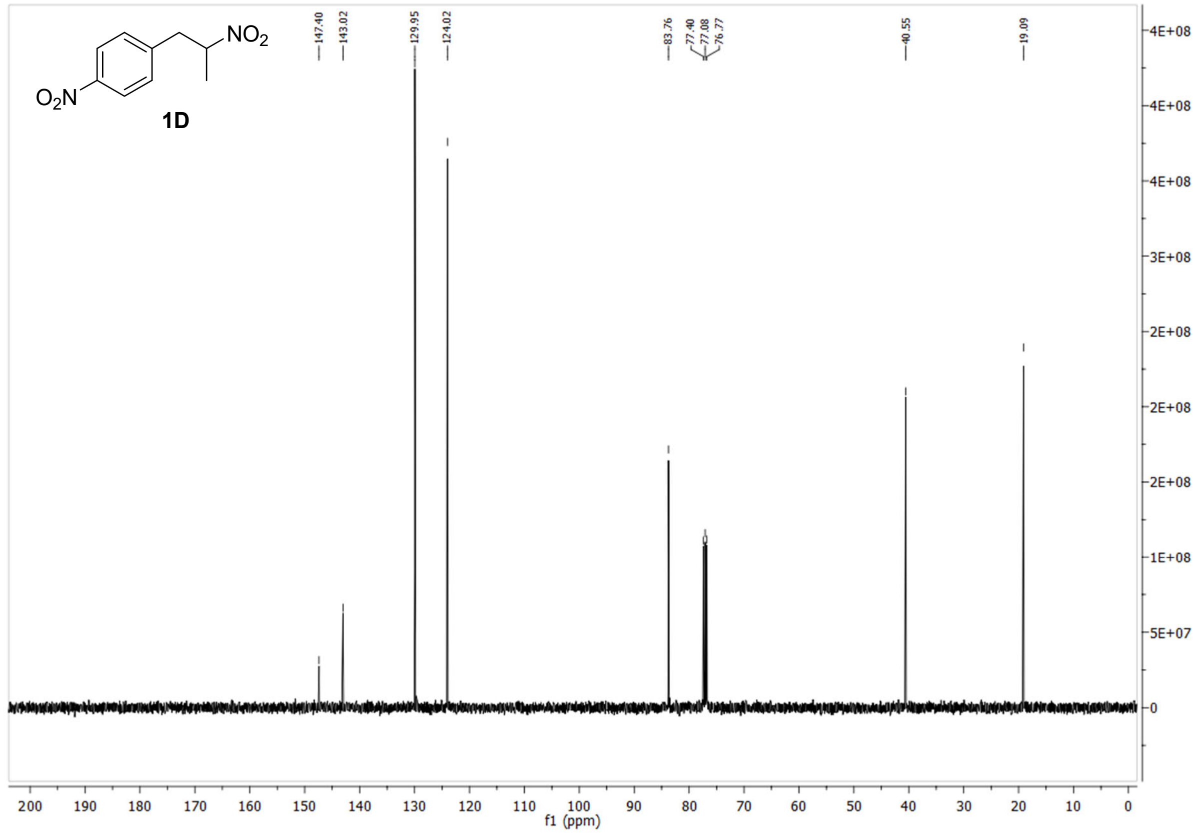

^{1}H NMR (400 MHz, $CDCl_3$) of compound **1E**:

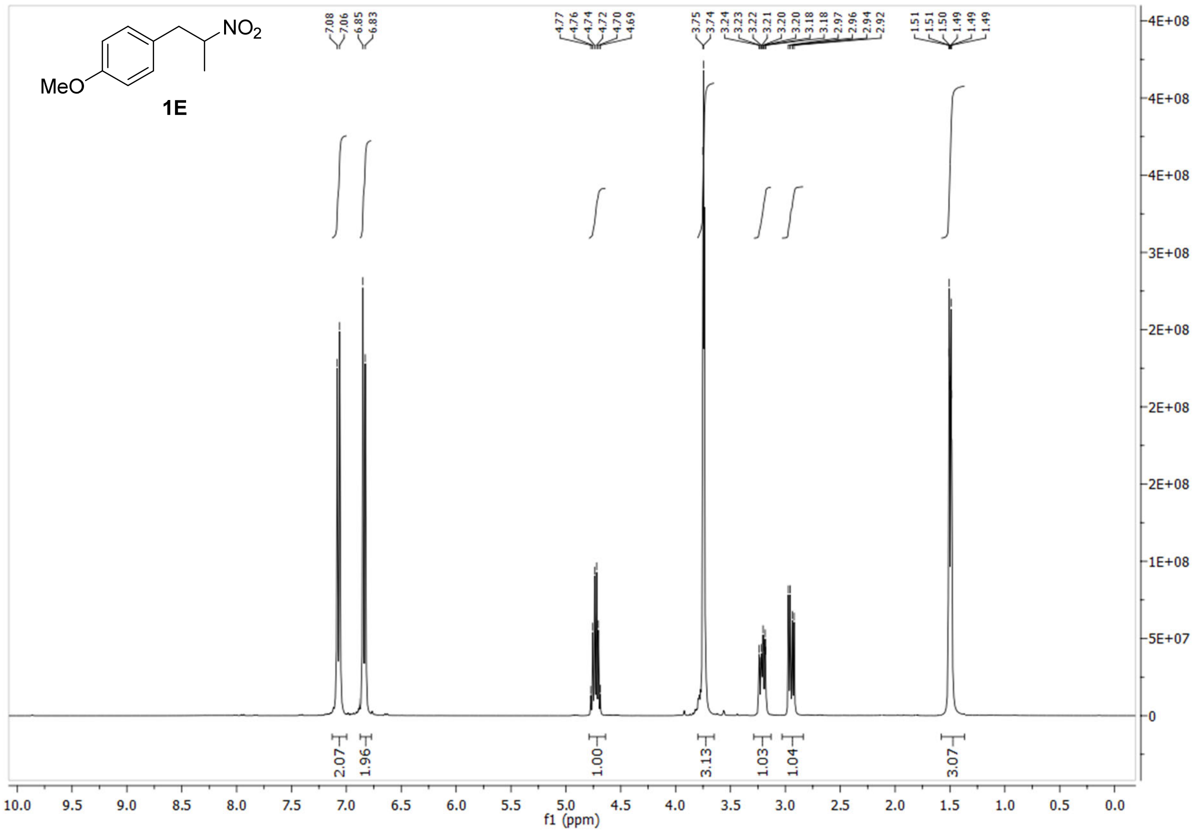

^{13}C NMR (100 MHz, $CDCl_3$) of compound **1E**:

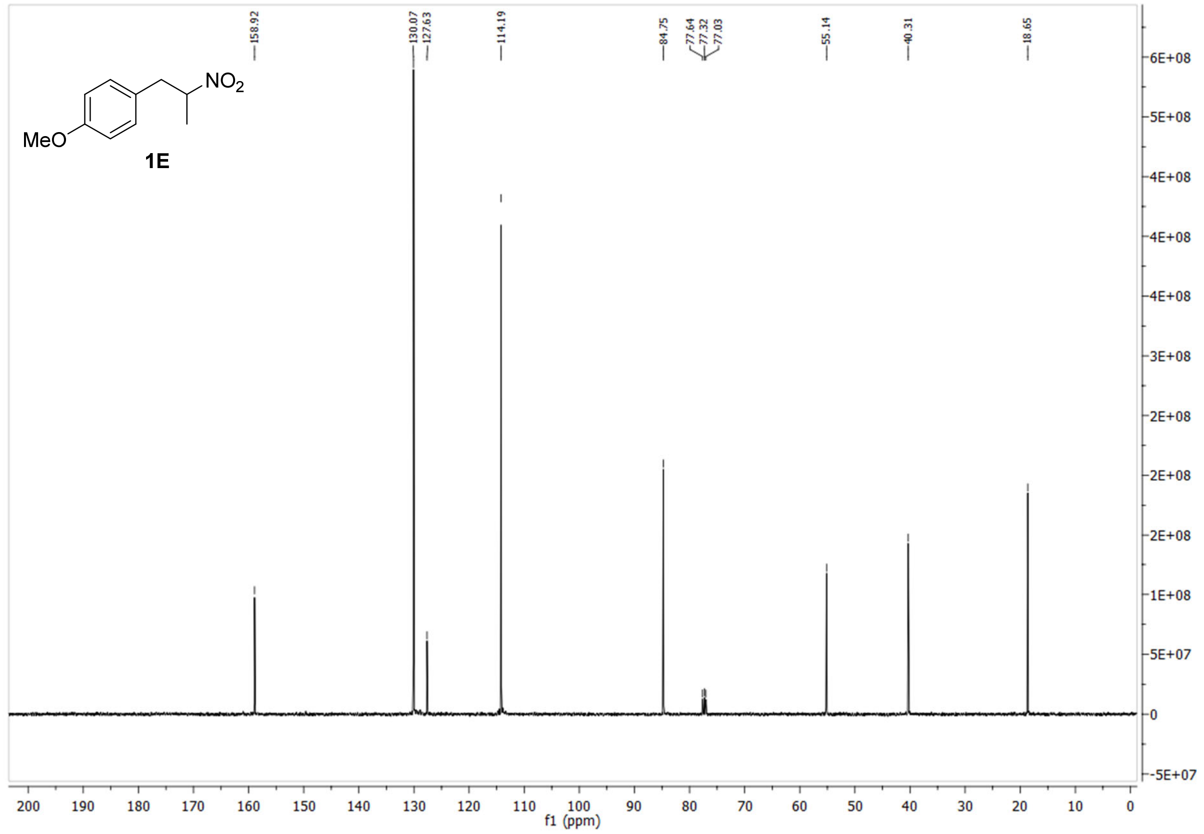

^{1}H NMR (400 MHz, $CDCl_3$) of compound **1F**:

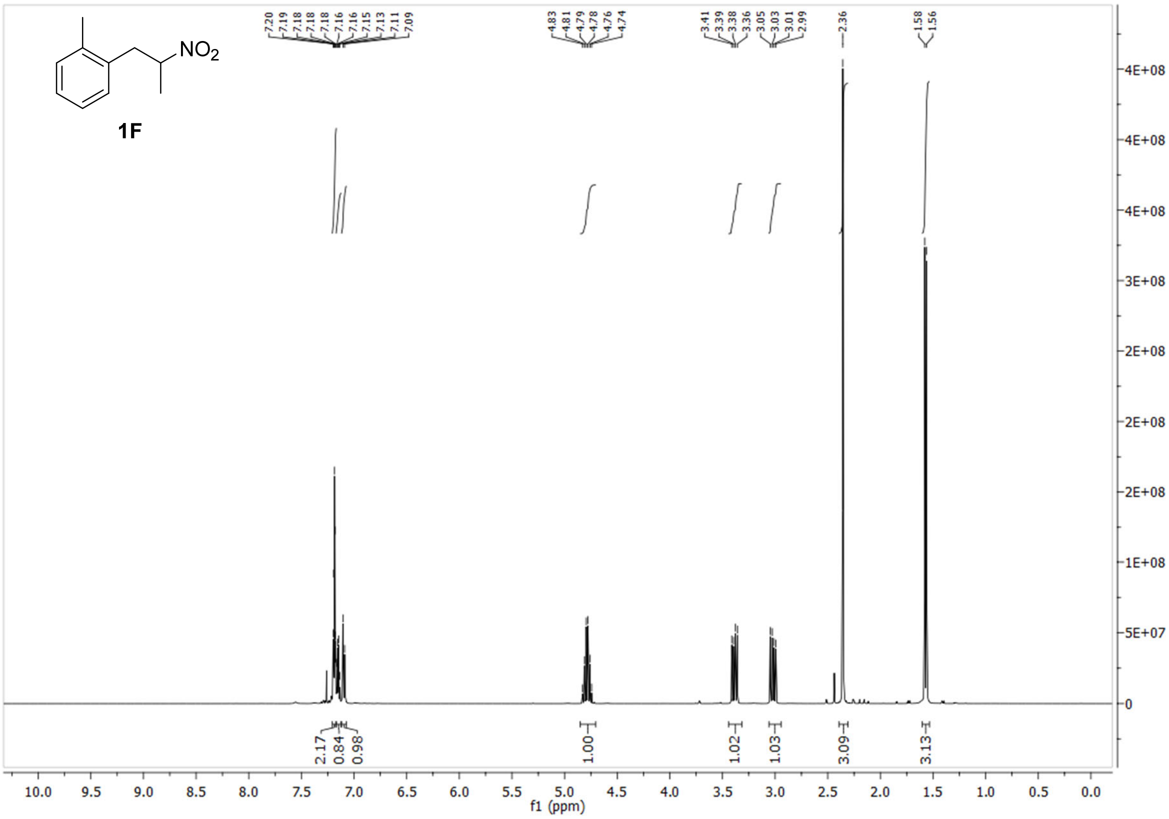

^{13}C NMR (100 MHz, $CDCl_3$) of compound **1F**:

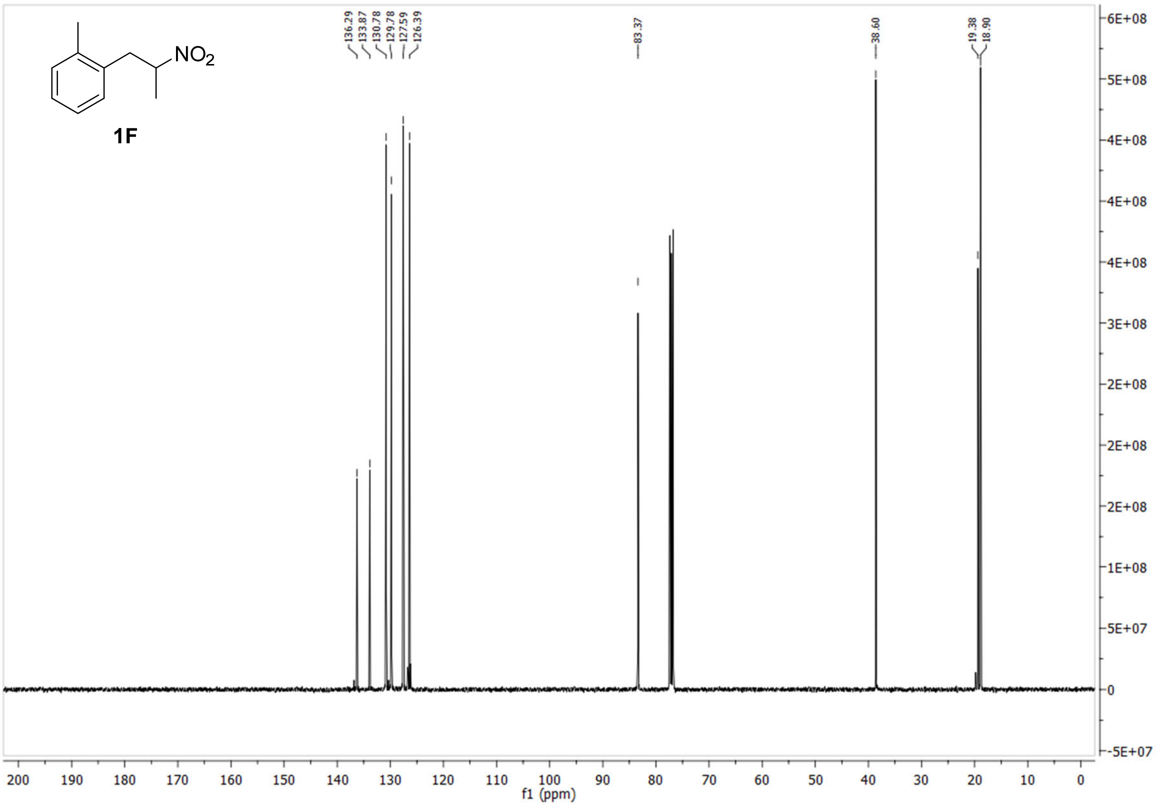

^{1}H NMR (400 MHz, $CDCl_3$) of compound **1G**:

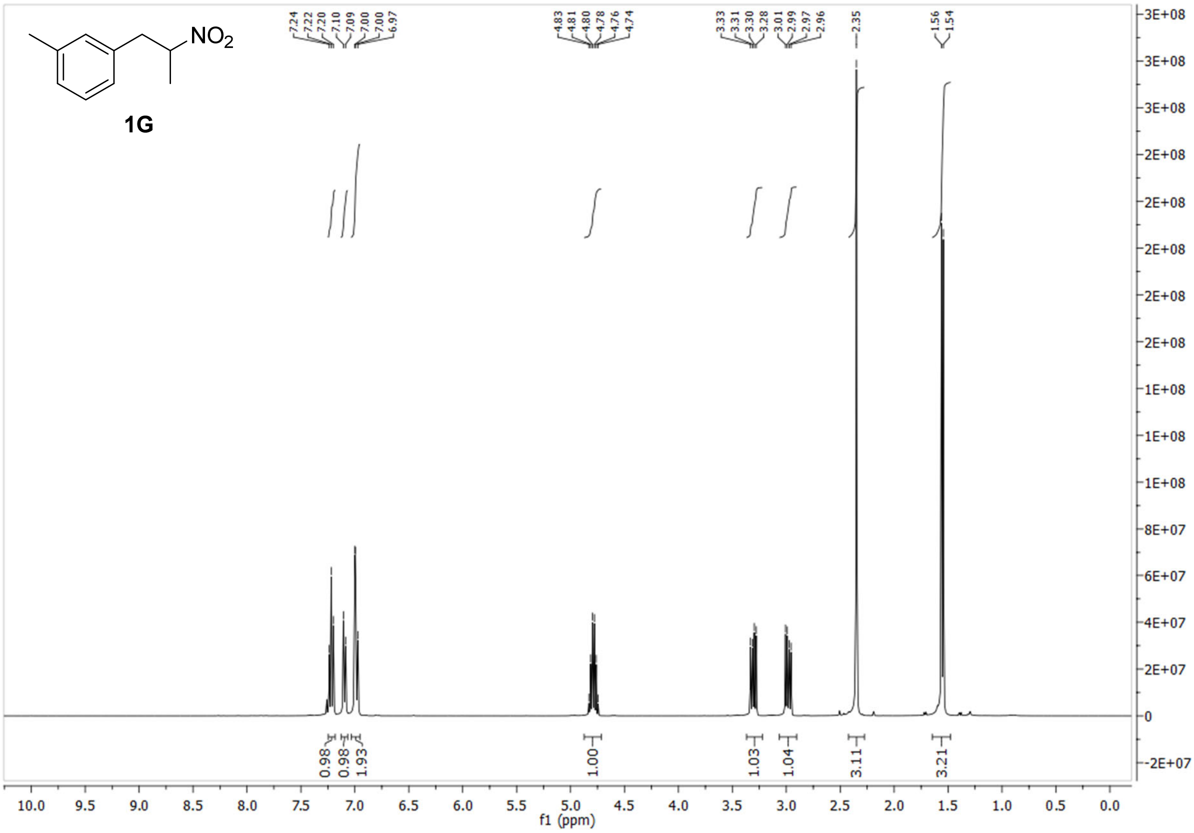

^{13}C NMR (100 MHz, $CDCl_3$) of compound **1G**:

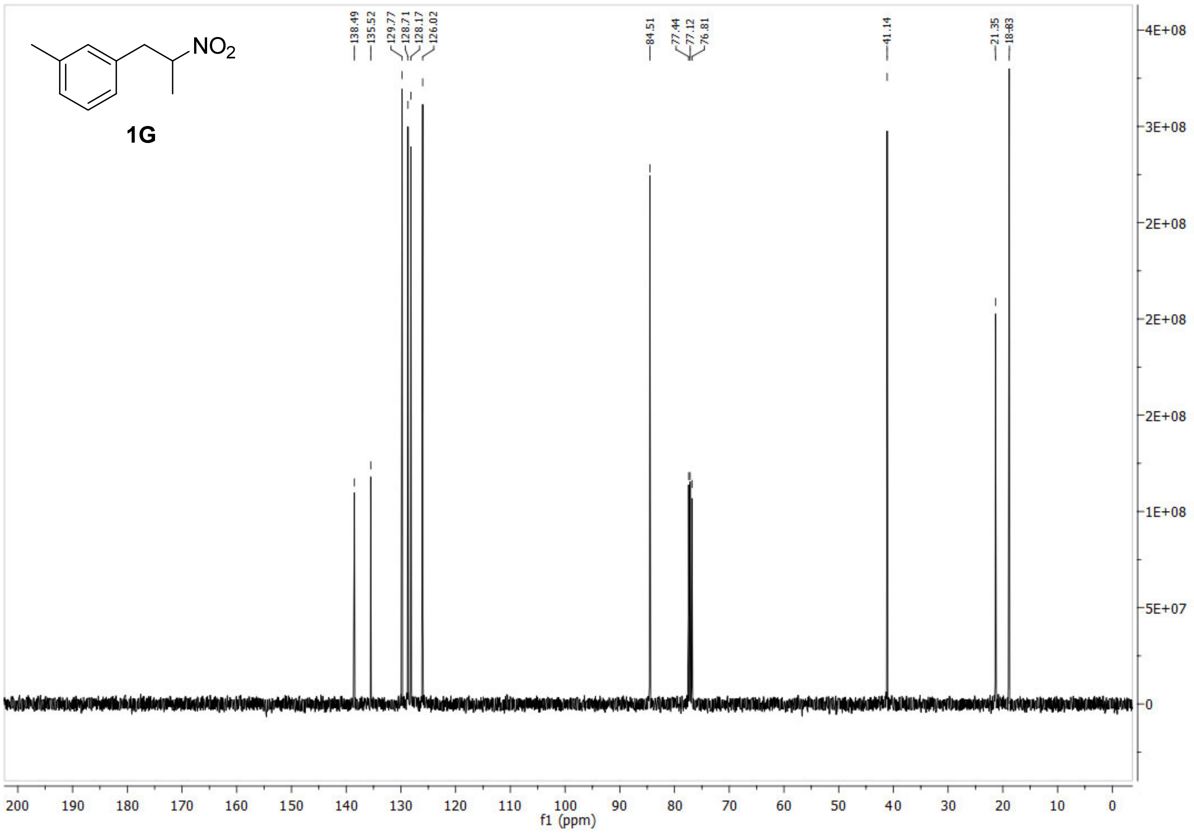

^{1}H NMR (400 MHz, $CDCl_3$) of compound **1H**:

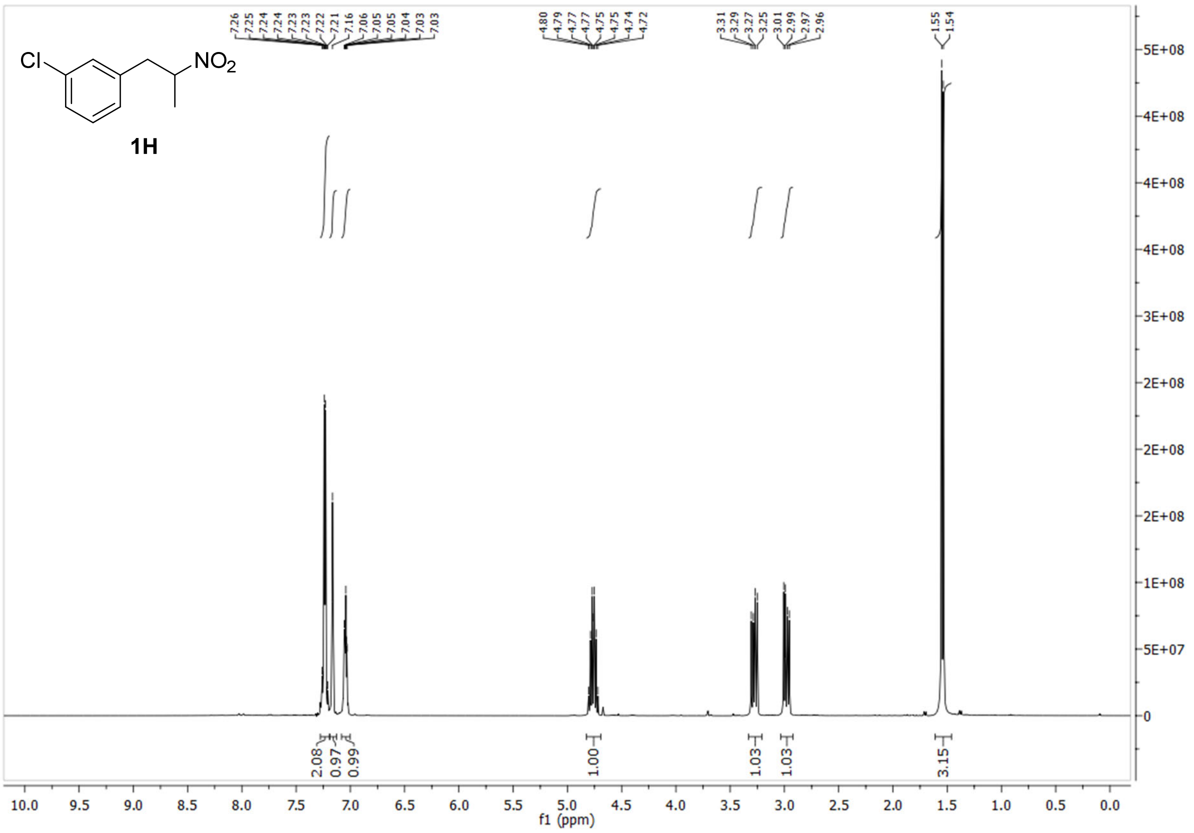

^{13}C NMR (100 MHz, $CDCl_3$) of compound **1H**:

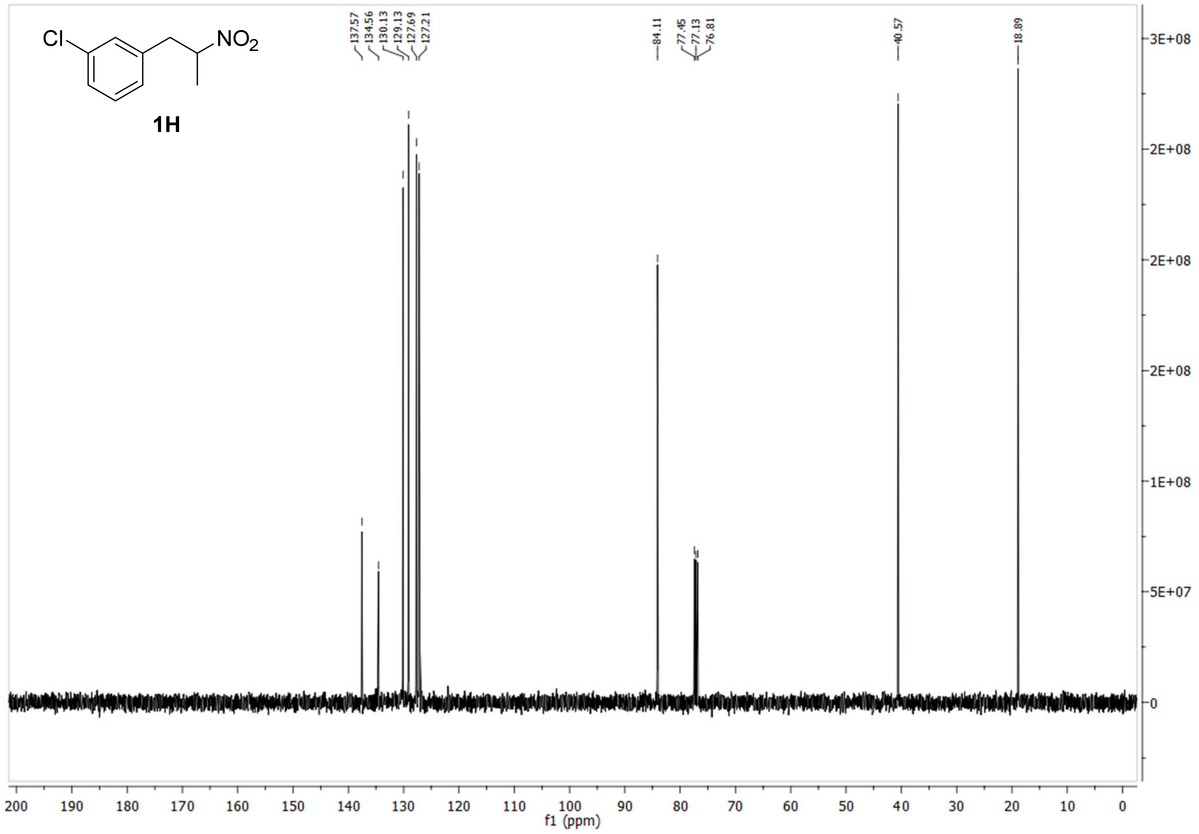

^{1}H NMR (400 MHz, $CDCl_3$) of compound **1l**:

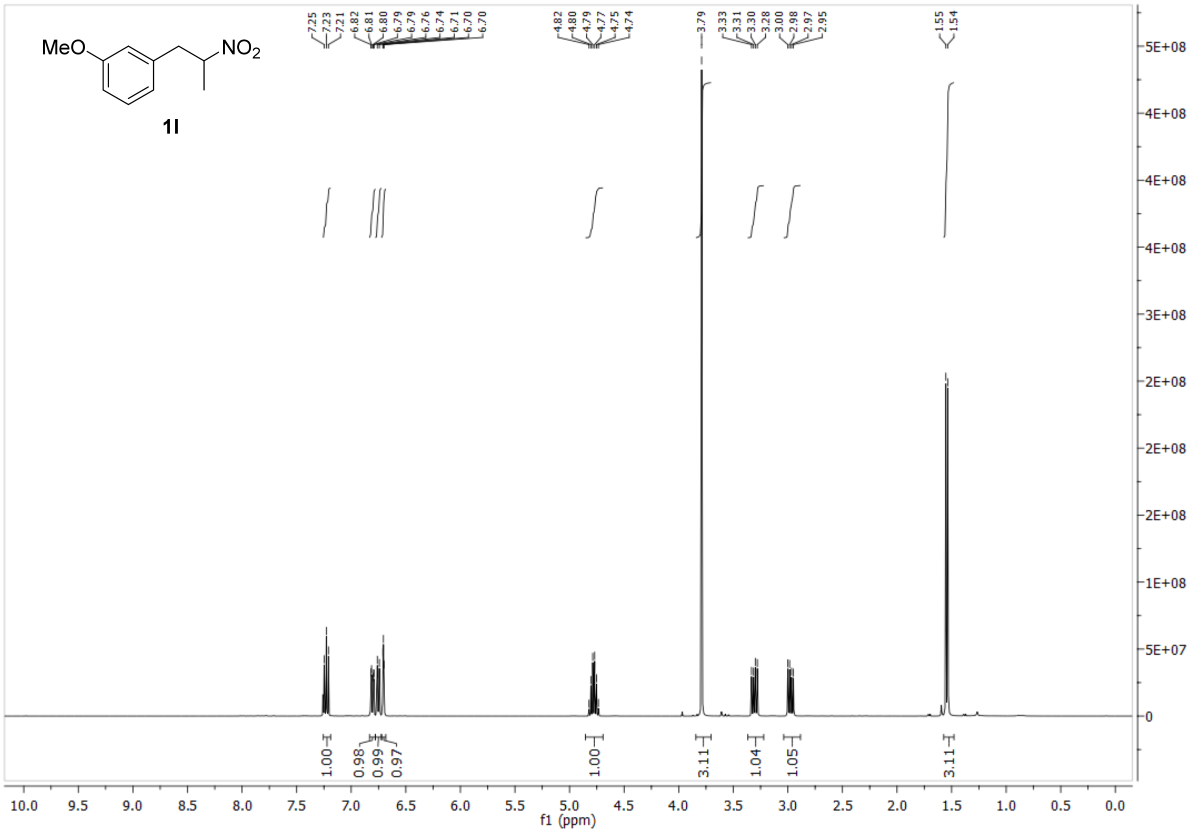

^{13}C NMR (100 MHz, $CDCl_3$) of compound **1l**:

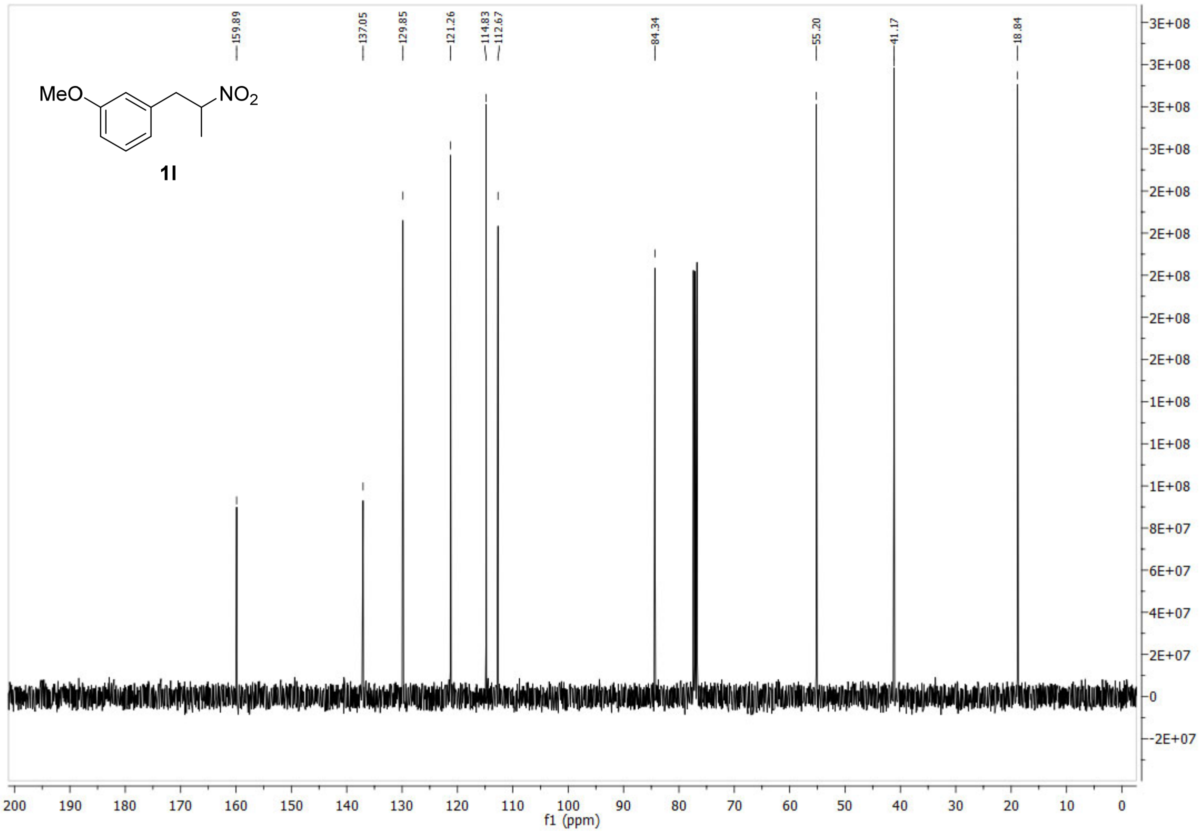

^{1}H NMR (400 MHz, $CDCl_3$) of compound **1J**:

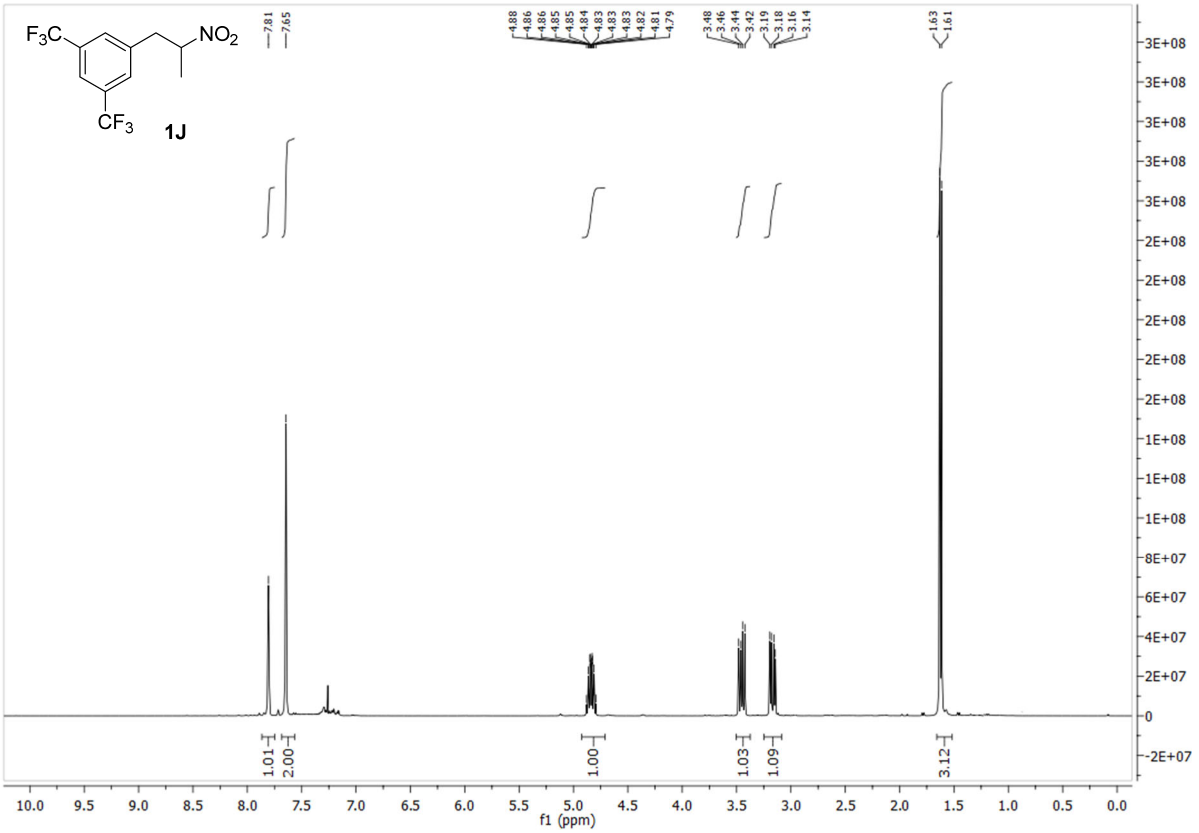

^{13}C NMR (100 MHz, $CDCl_3$) of compound **1J**:

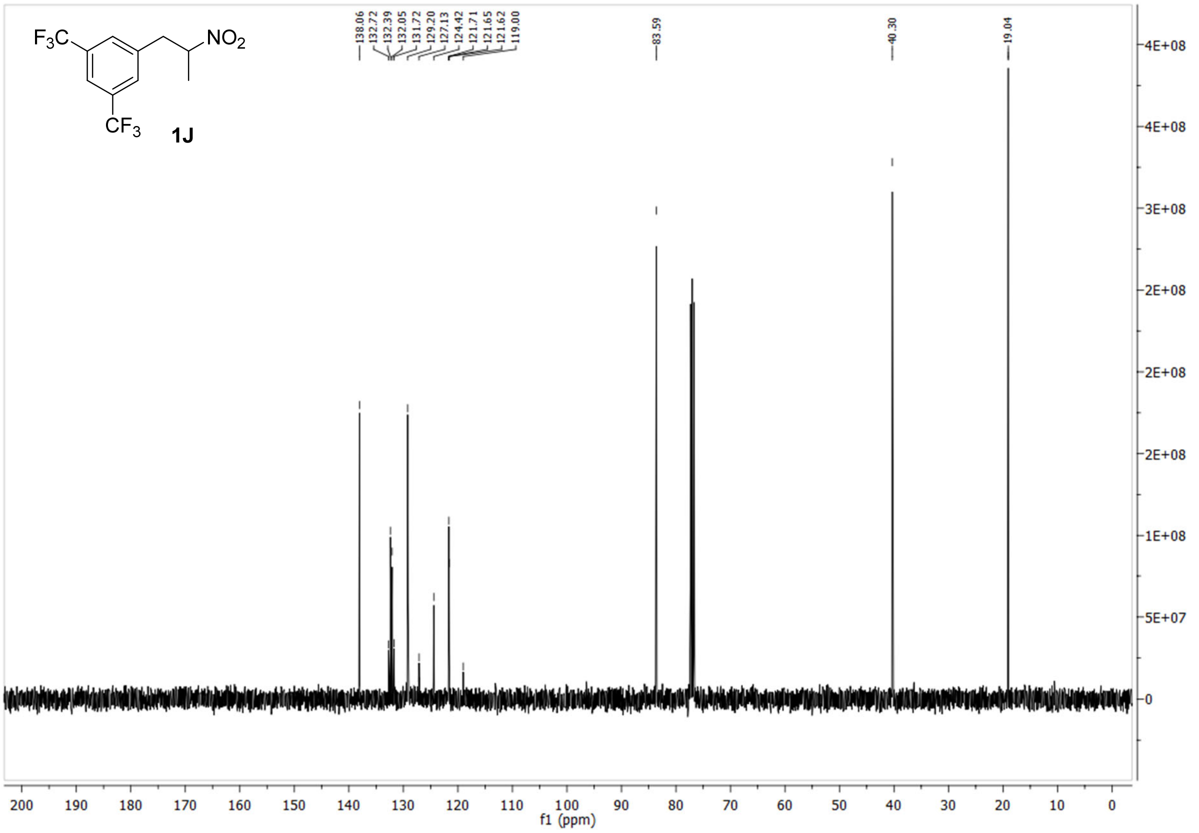

^{1}H NMR (400 MHz, $CDCl_3$) of compound **1K**:

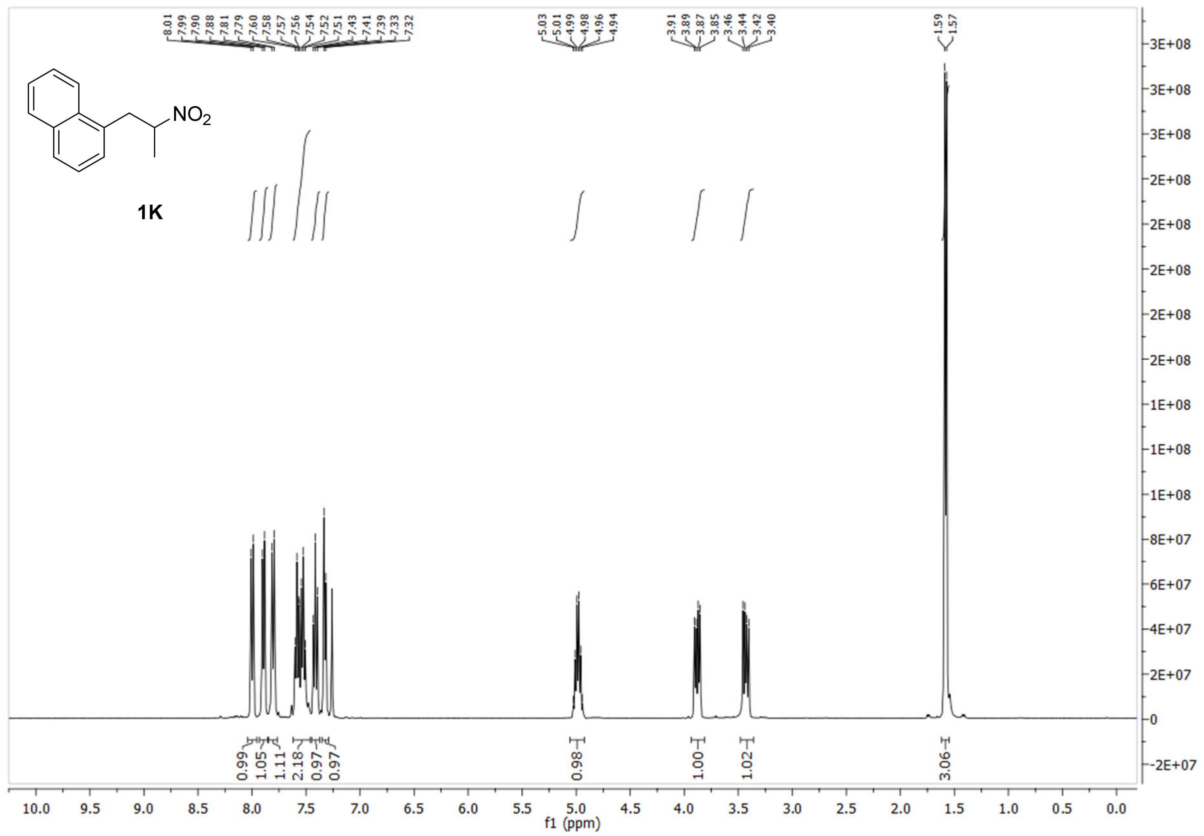

^{13}C NMR (100 MHz, $CDCl_3$) of compound **1K**:

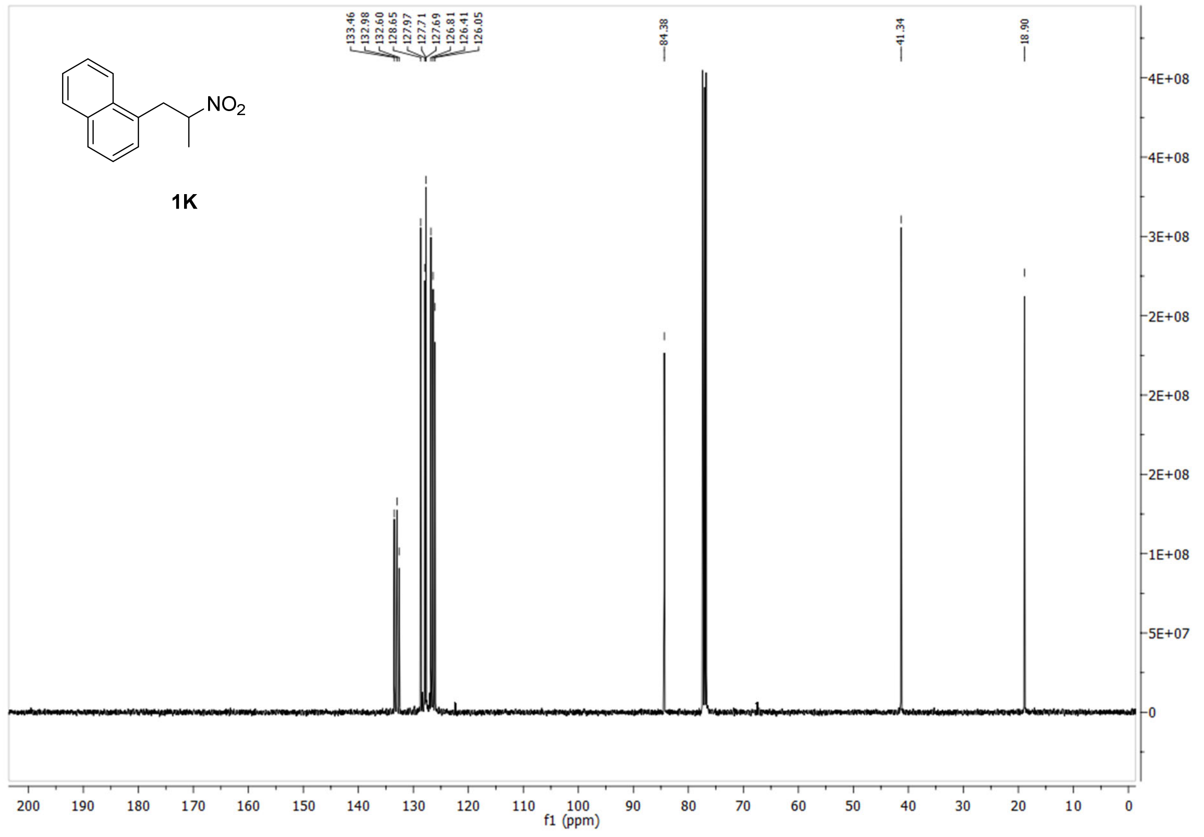

^{1}H NMR (400 MHz, $CDCl_3$) of compound **1L**:

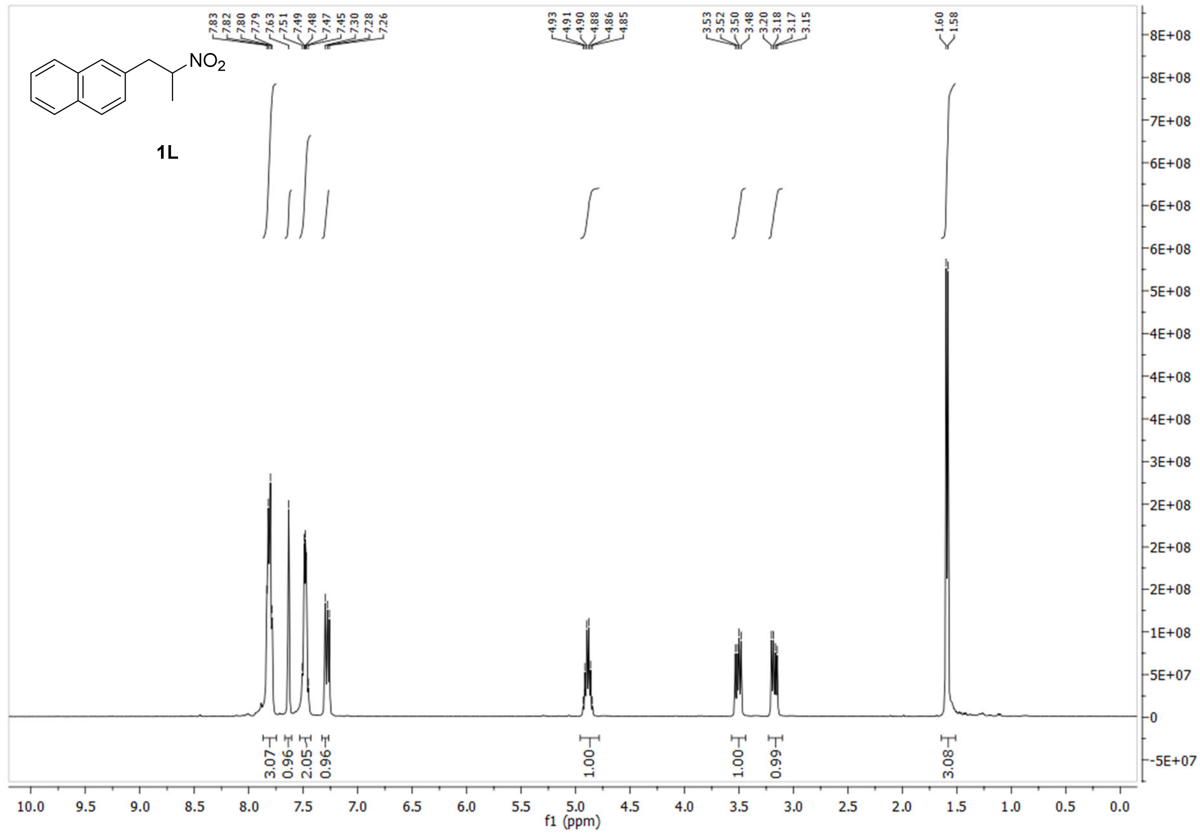

^{13}C NMR (100 MHz, $CDCl_3$) of compound **1L**:

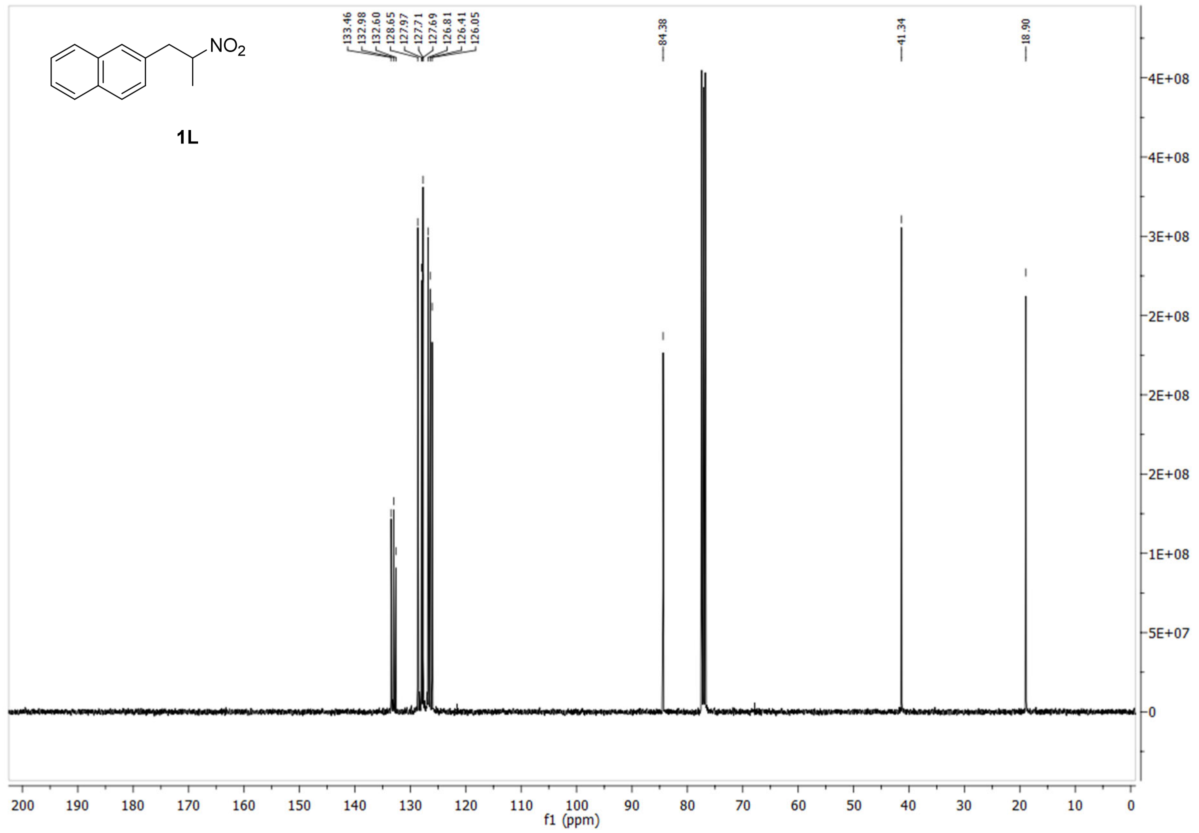

^{1}H NMR (400 MHz, $CDCl_3$) of compound **1M**:

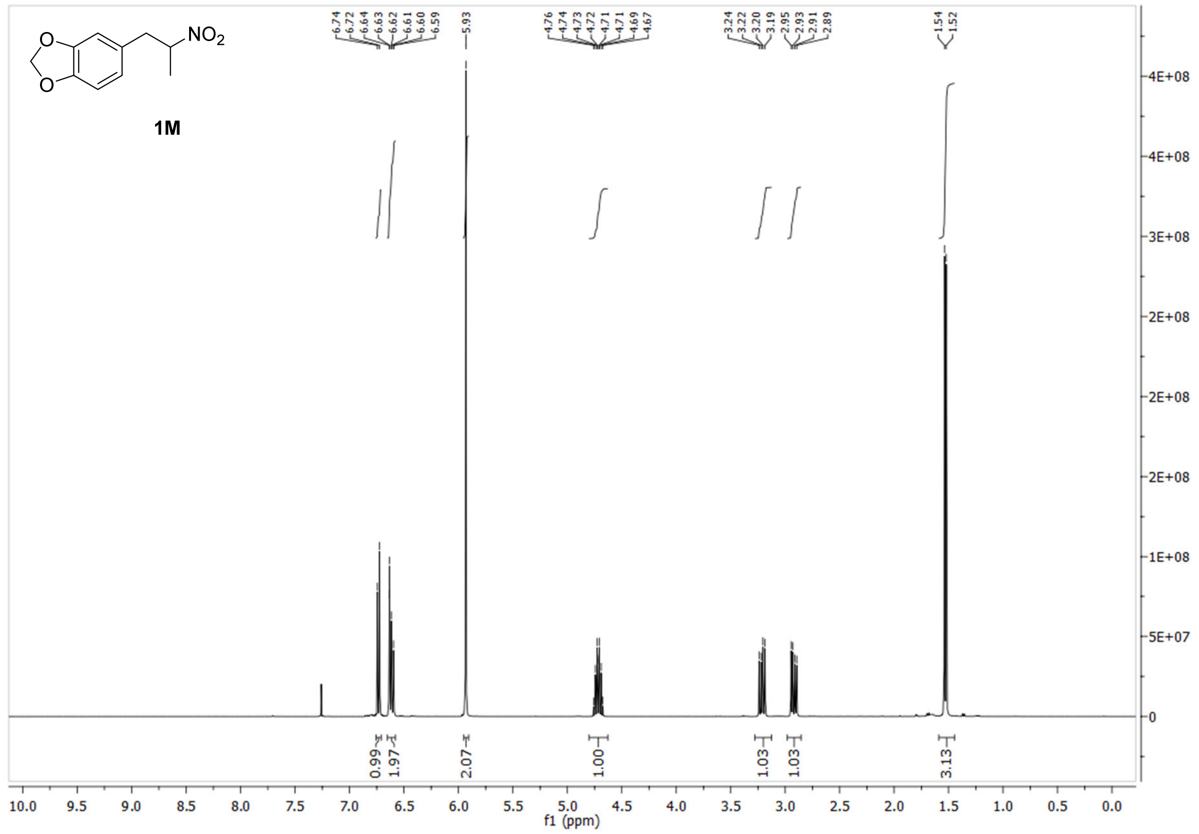

^{13}C NMR (100 MHz, $CDCl_3$) of compound **1M**:

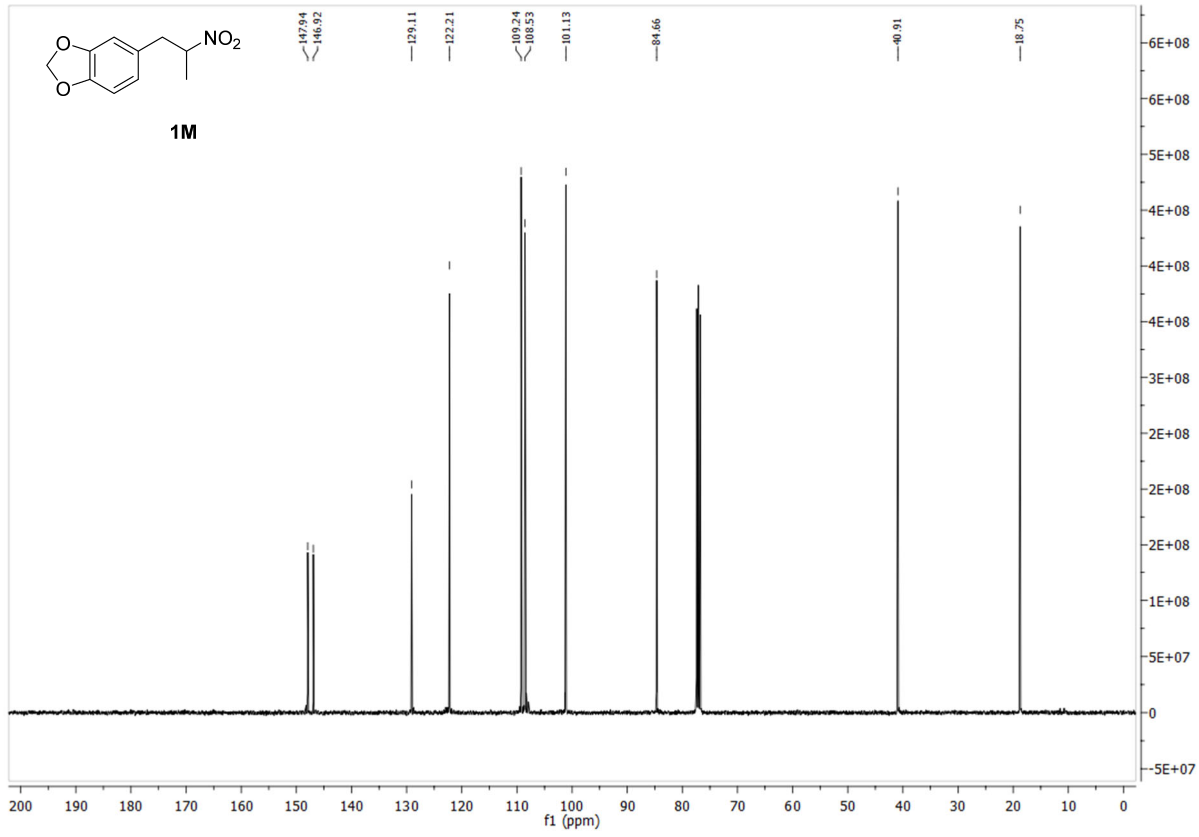

^{1}H NMR (400 MHz, $CDCl_3$) of compound **1O**:

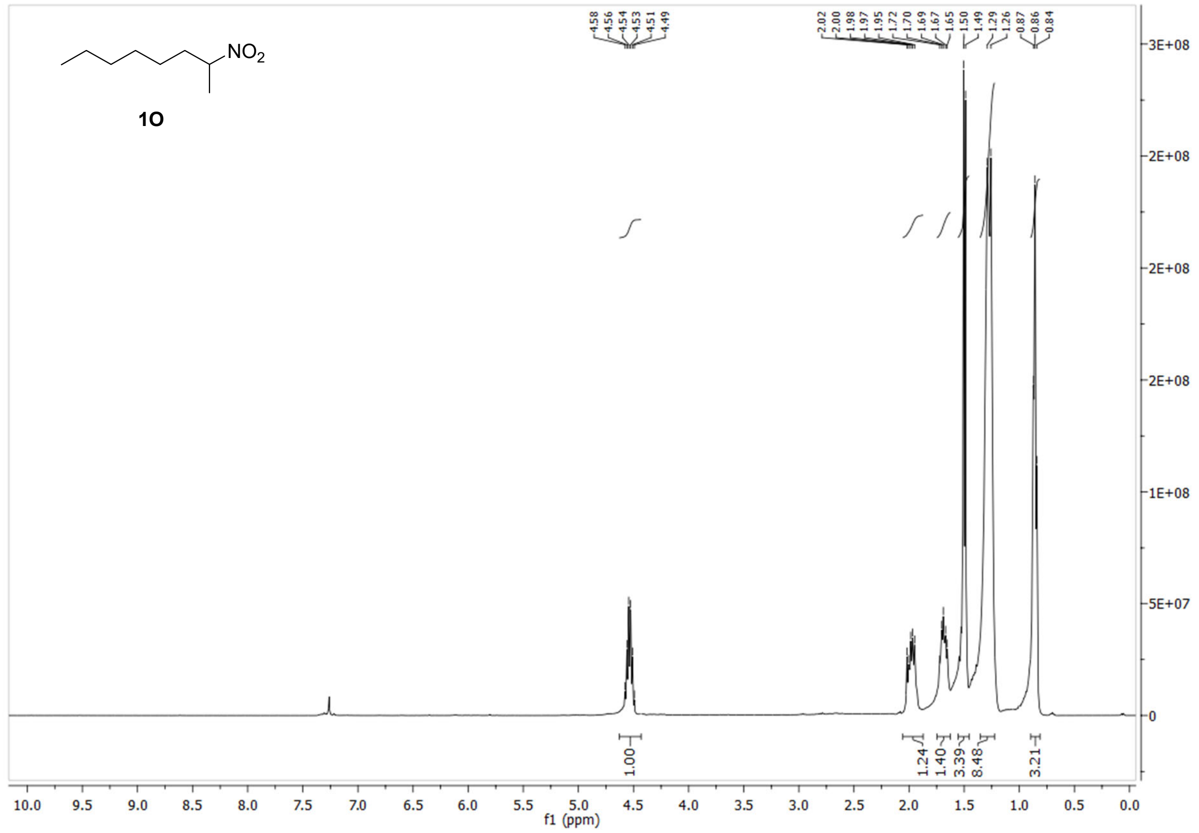

^{13}C NMR (100 MHz, $CDCl_3$) of compound **1O**:

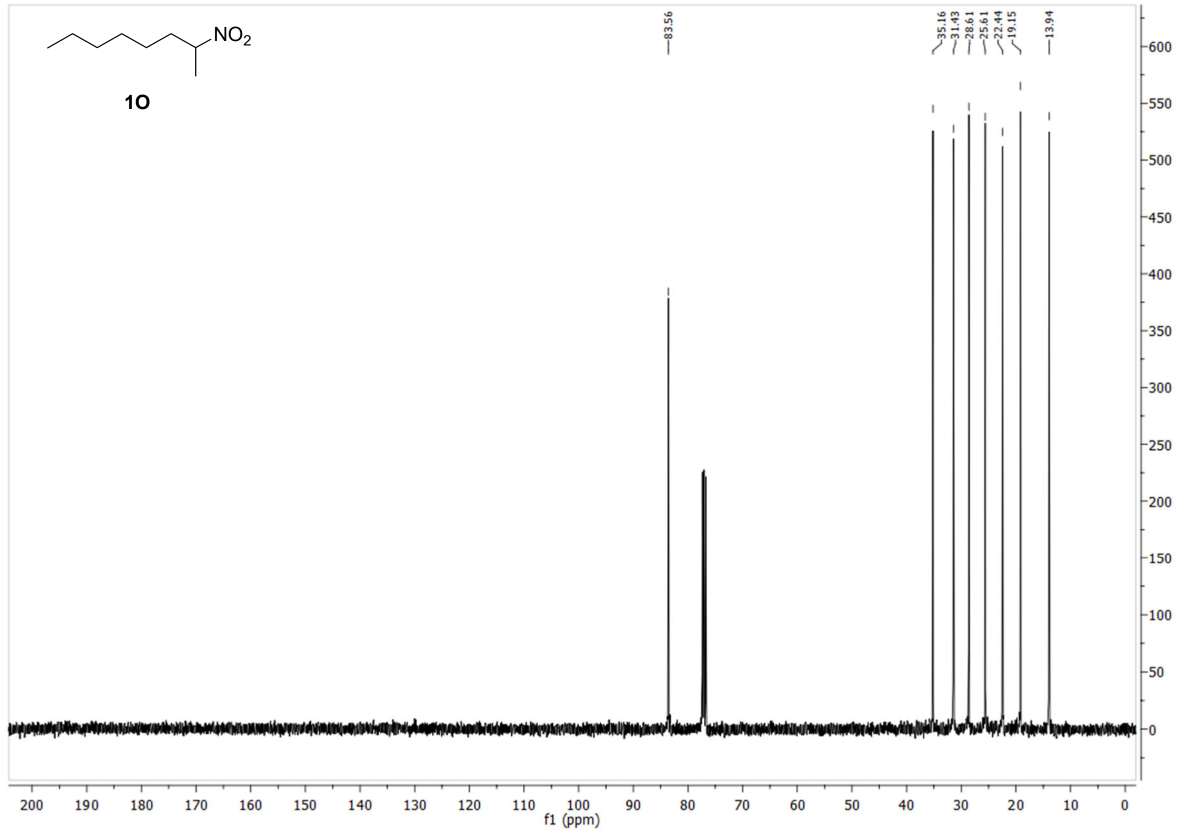

^{1}H NMR (400 MHz, $CDCl_3$) of compound **2b**:

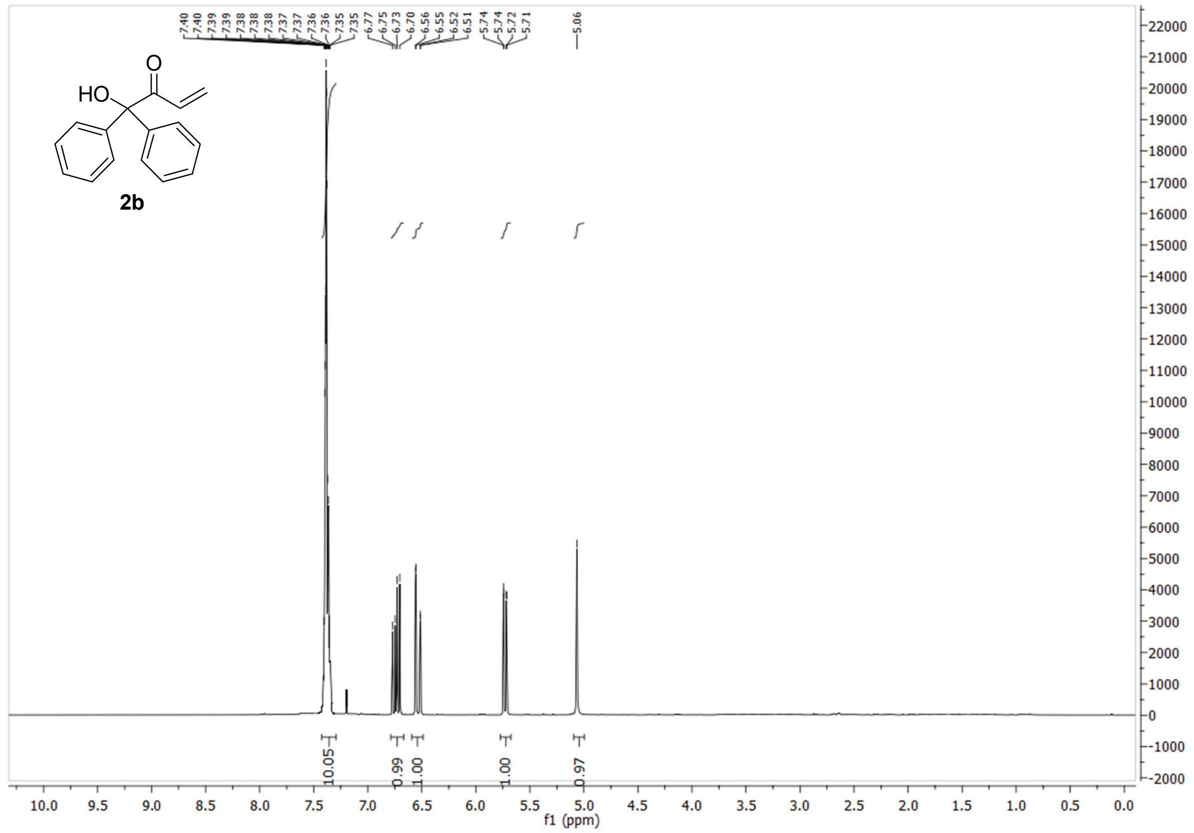

^{13}C NMR (100 MHz, $CDCl_3$) of compound **2b**:

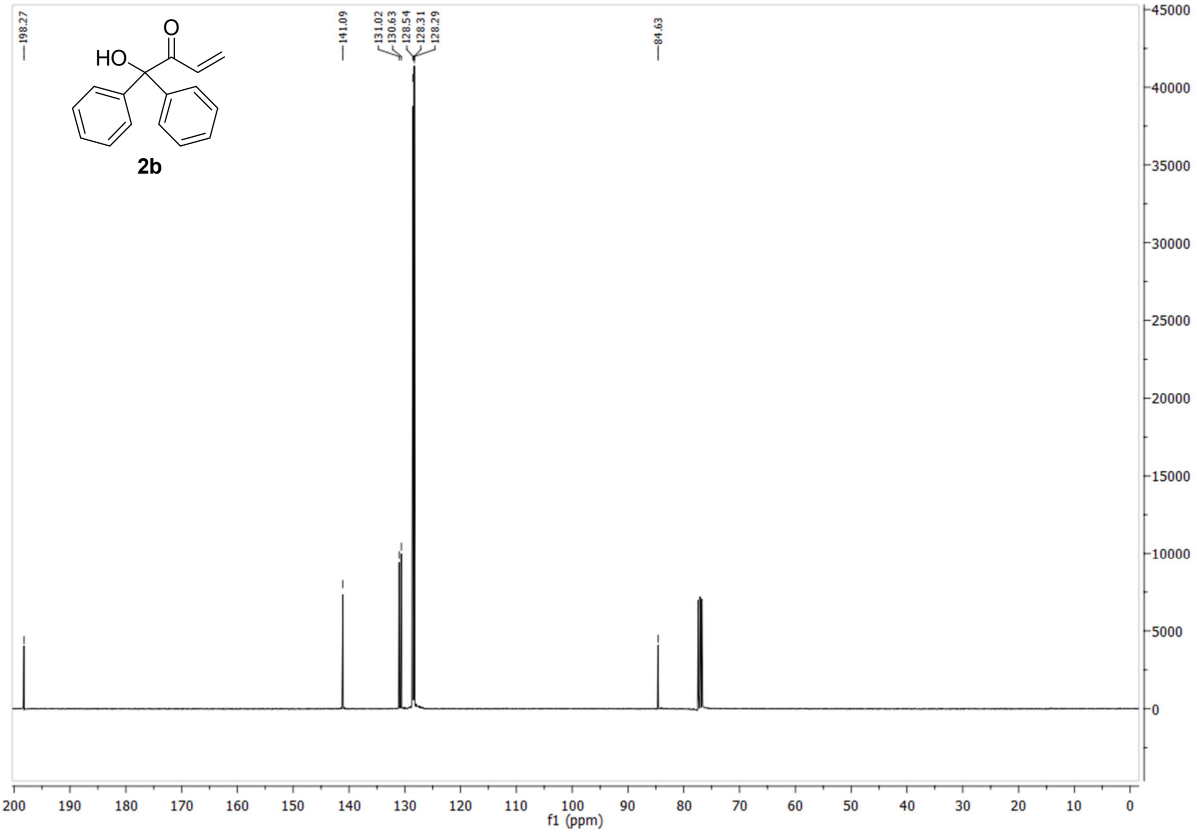

^{1}H NMR (400 MHz, $CDCl_3$) of compound **2c**:

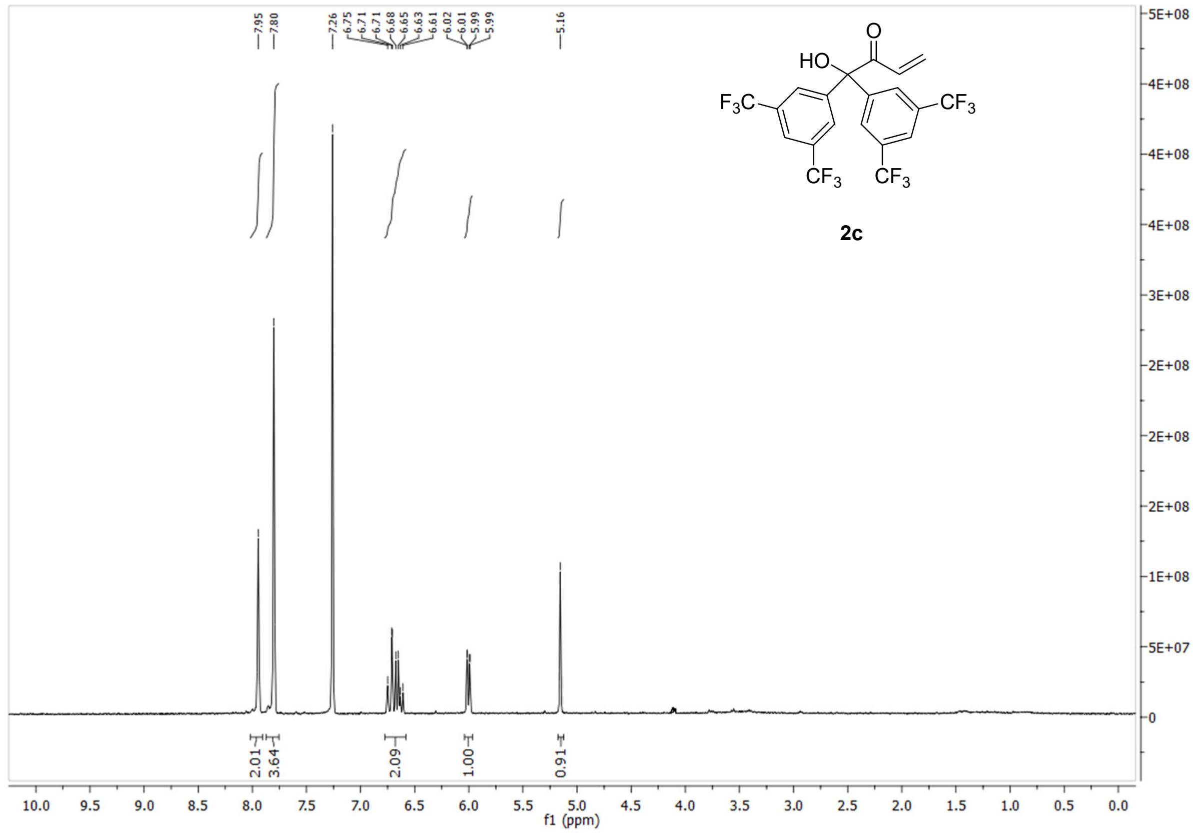

^{13}C NMR (100 MHz, $CDCl_3$) of compound **2c**:

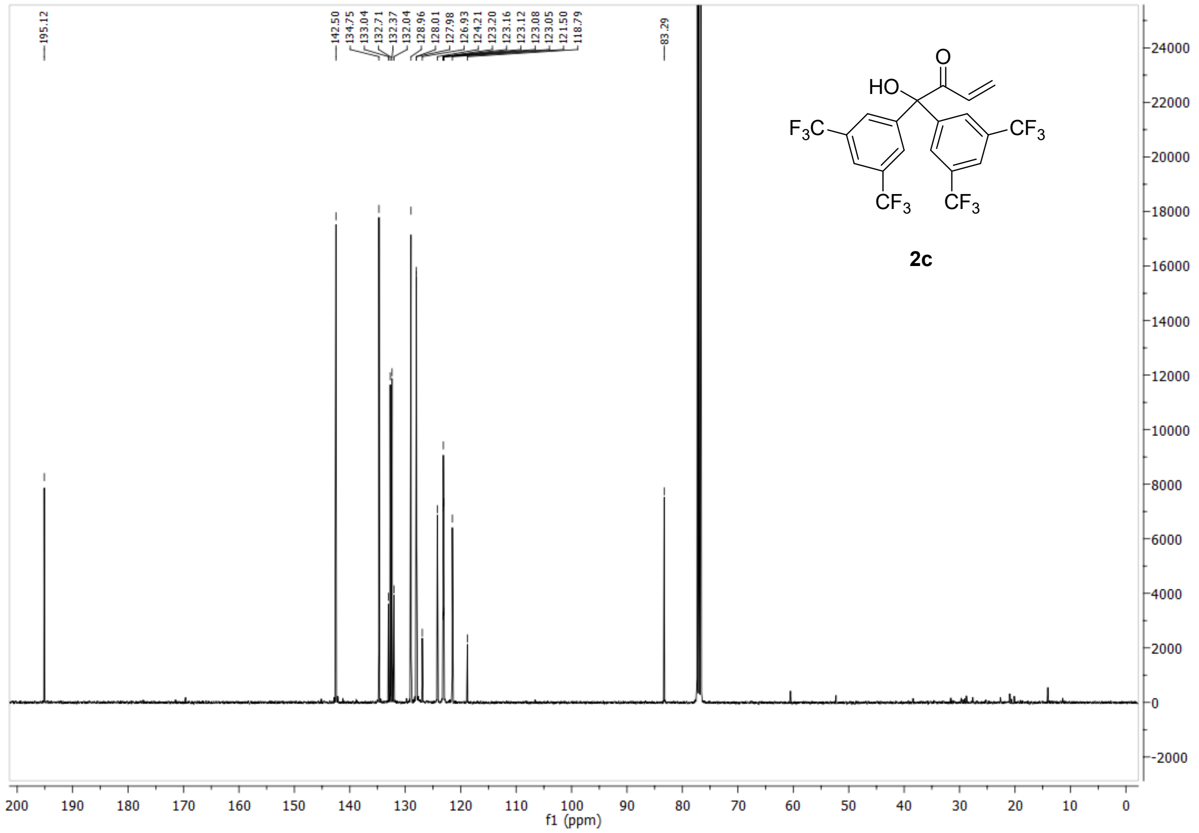

^{1}H NMR (400 MHz, $CDCl_3$) of compound **2d**:

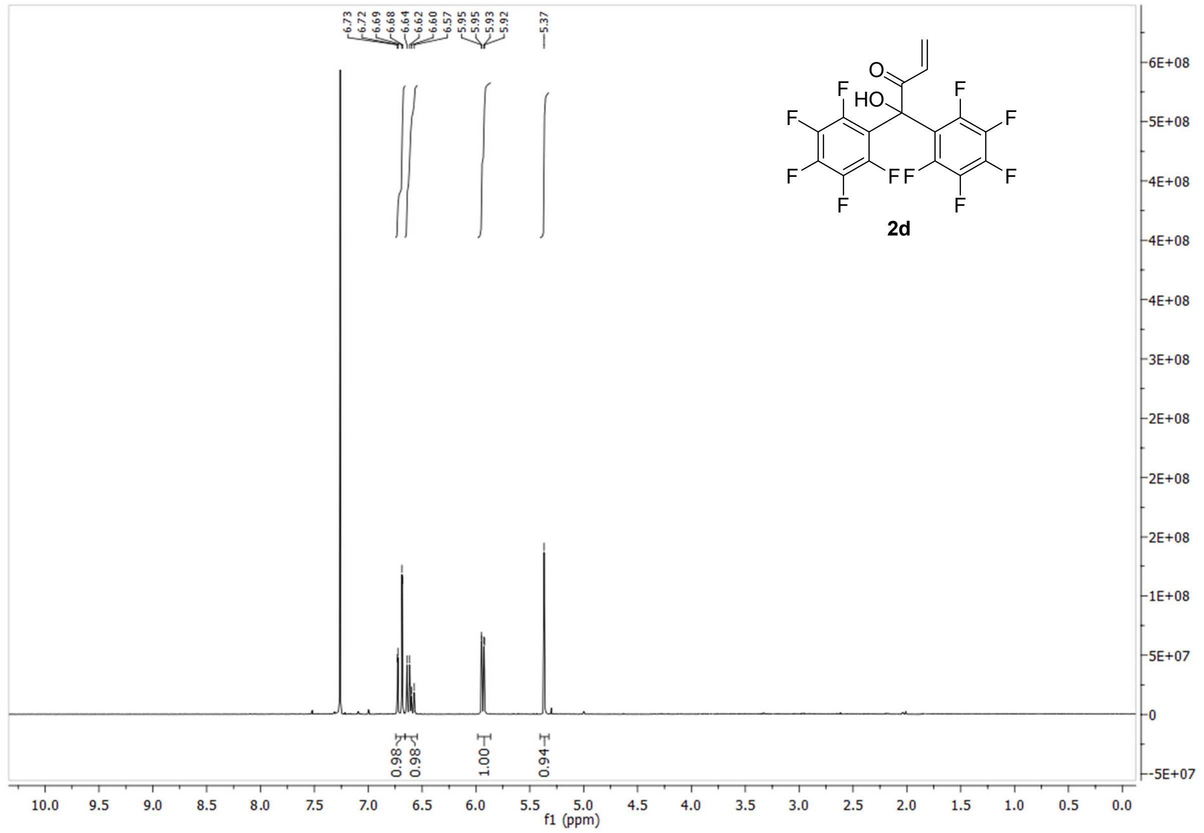

^{13}C NMR (100 MHz, $CDCl_3$) of compound **2d**:

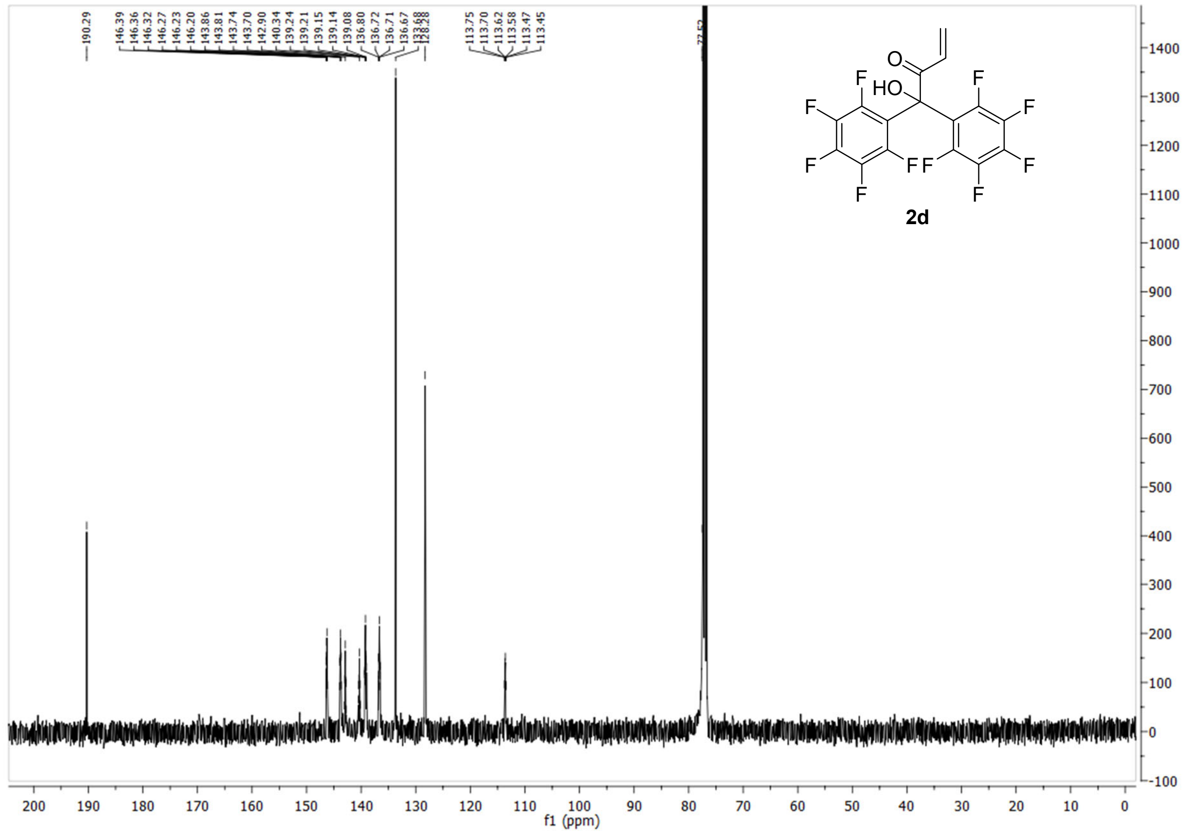

^{1}H NMR (400 MHz, $CDCl_3$) of compound **2f**:

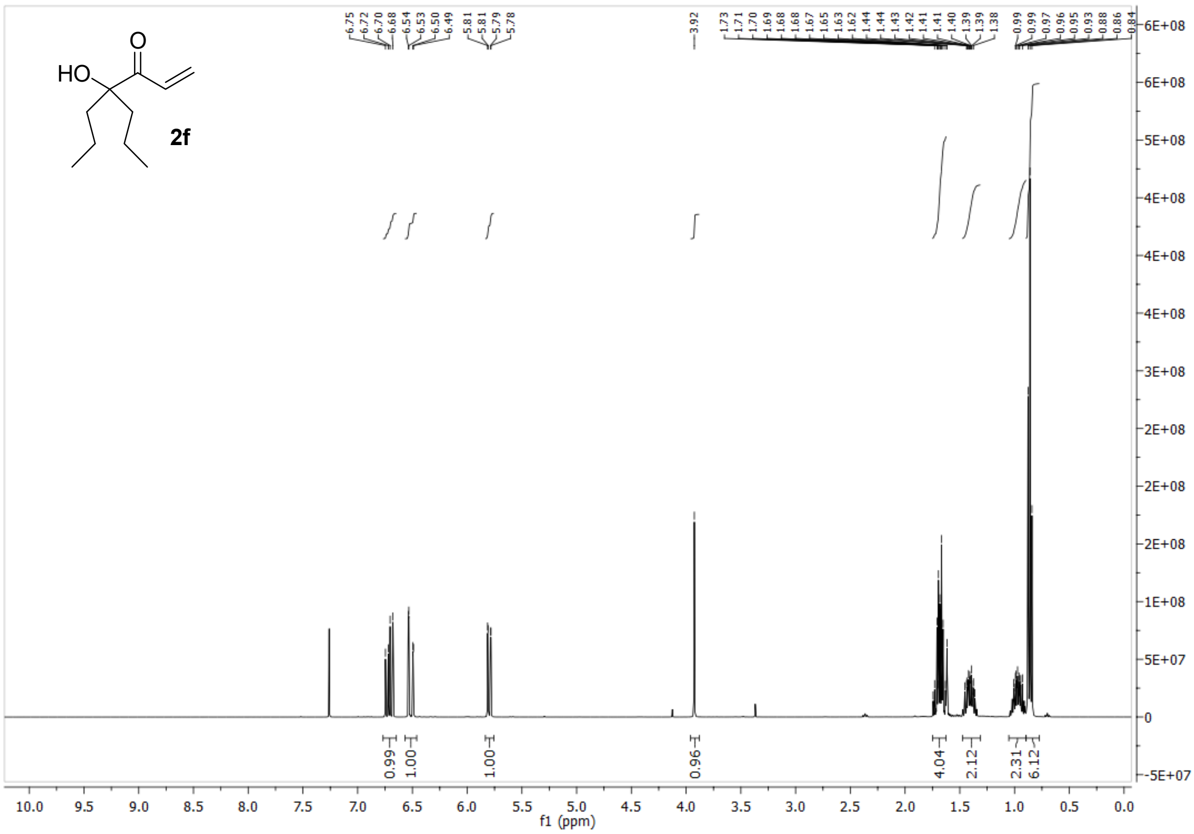

^{13}C NMR (100 MHz, $CDCl_3$) of compound **2f**:

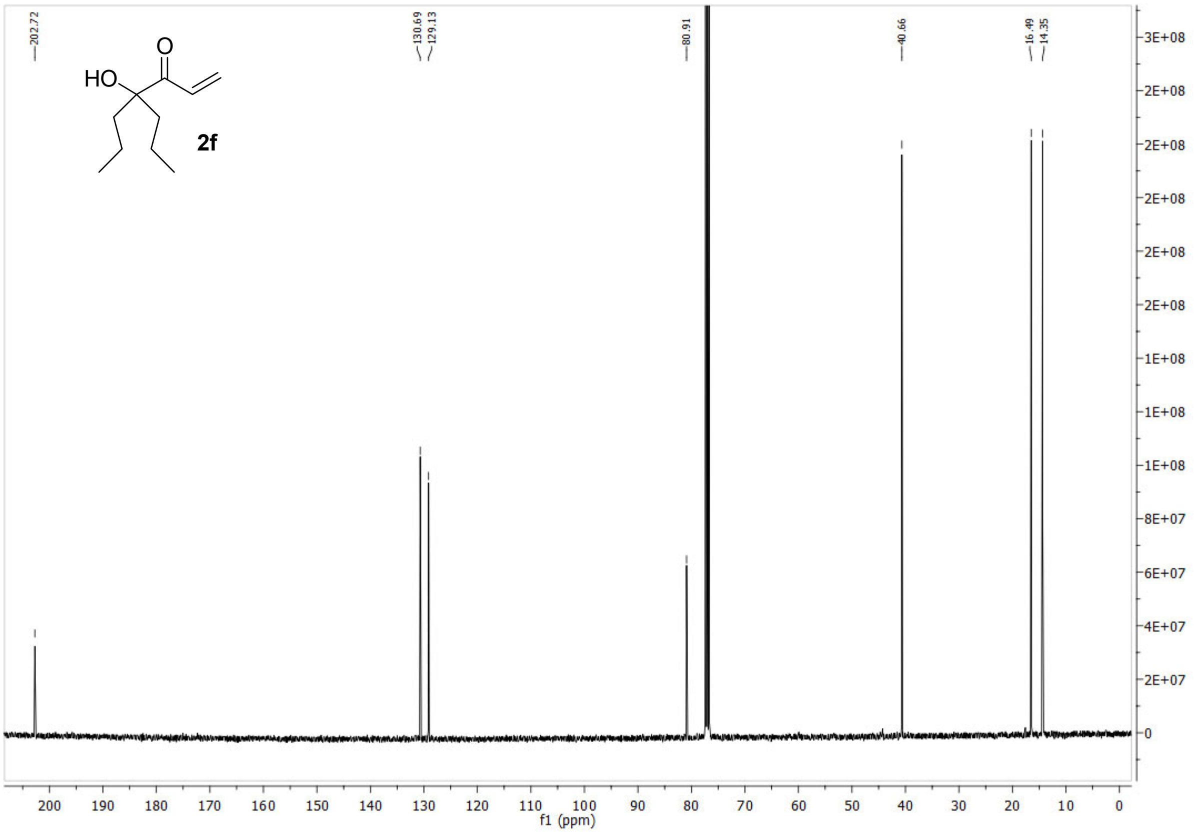

^{1}H NMR (400 MHz, $CDCl_3$) of compound **2g**:

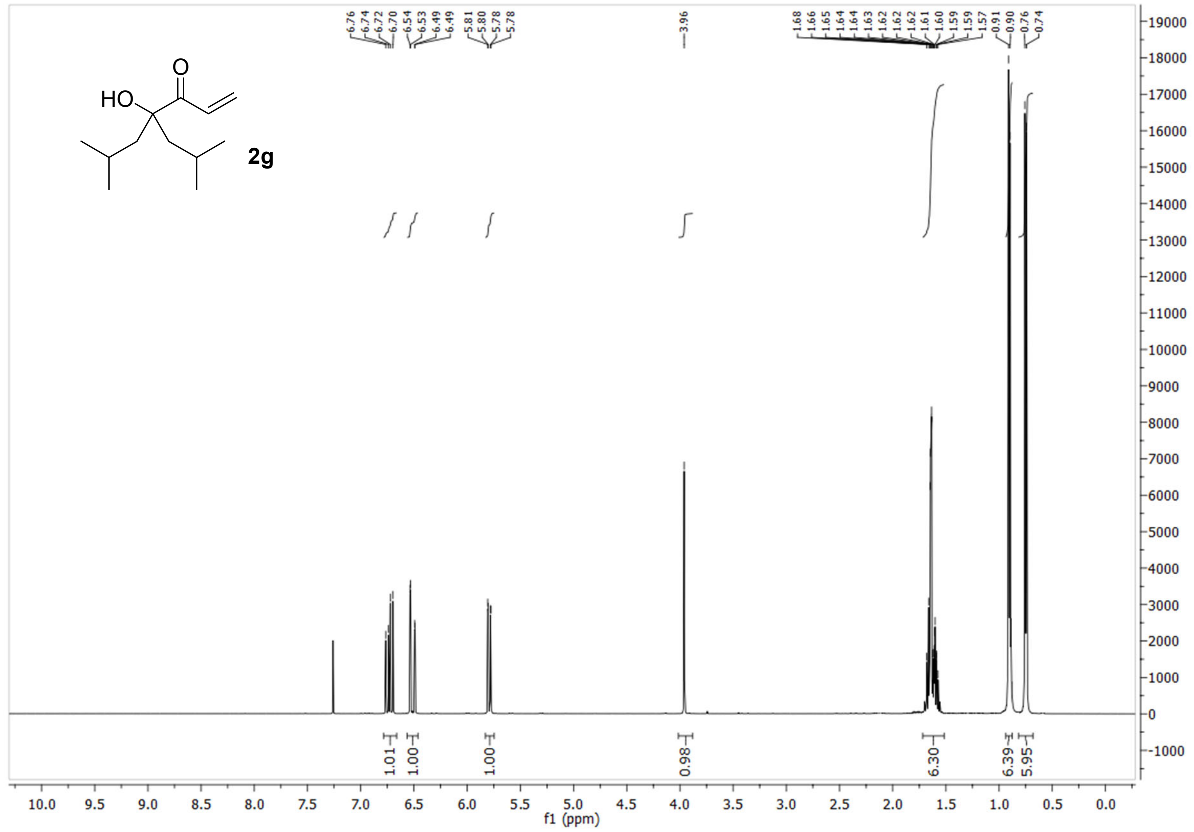

^{13}C NMR (100 MHz, $CDCl_3$) of compound **2g**:

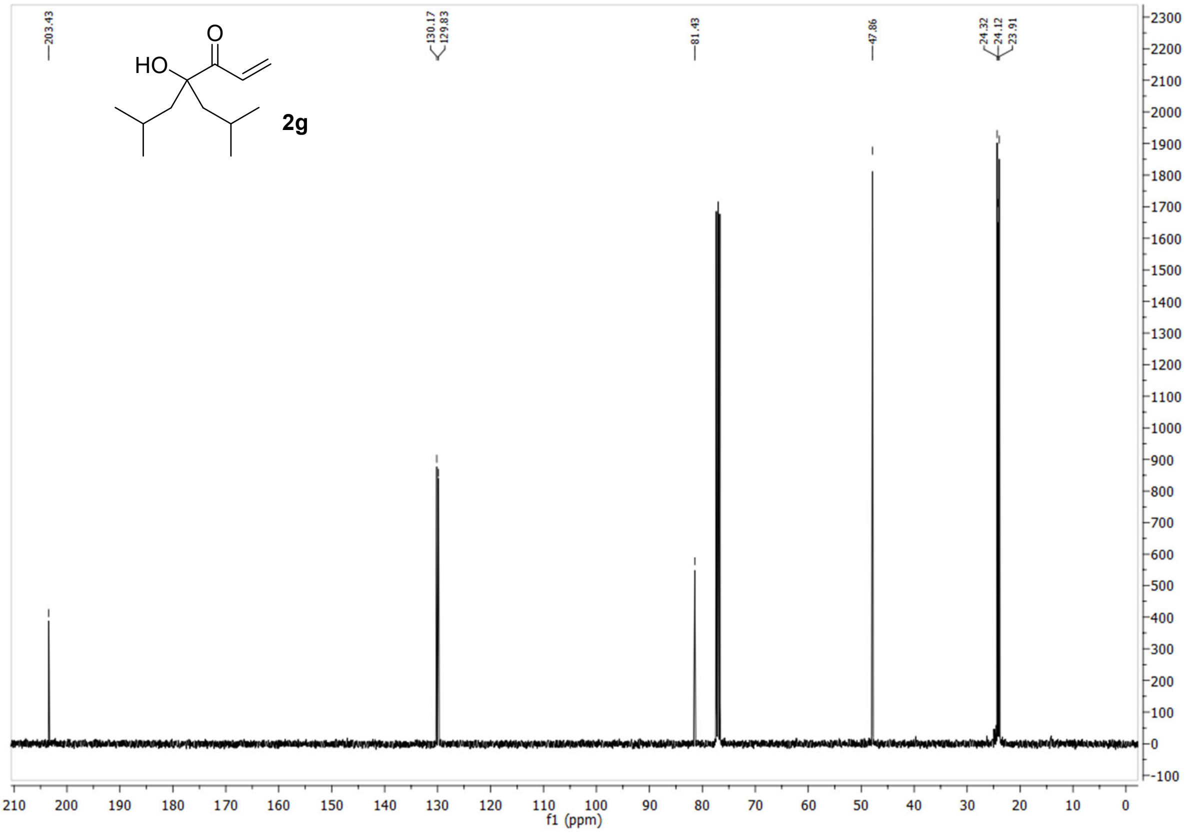

^{1}H NMR (400 MHz, $CDCl_3$) of compound **2i**:

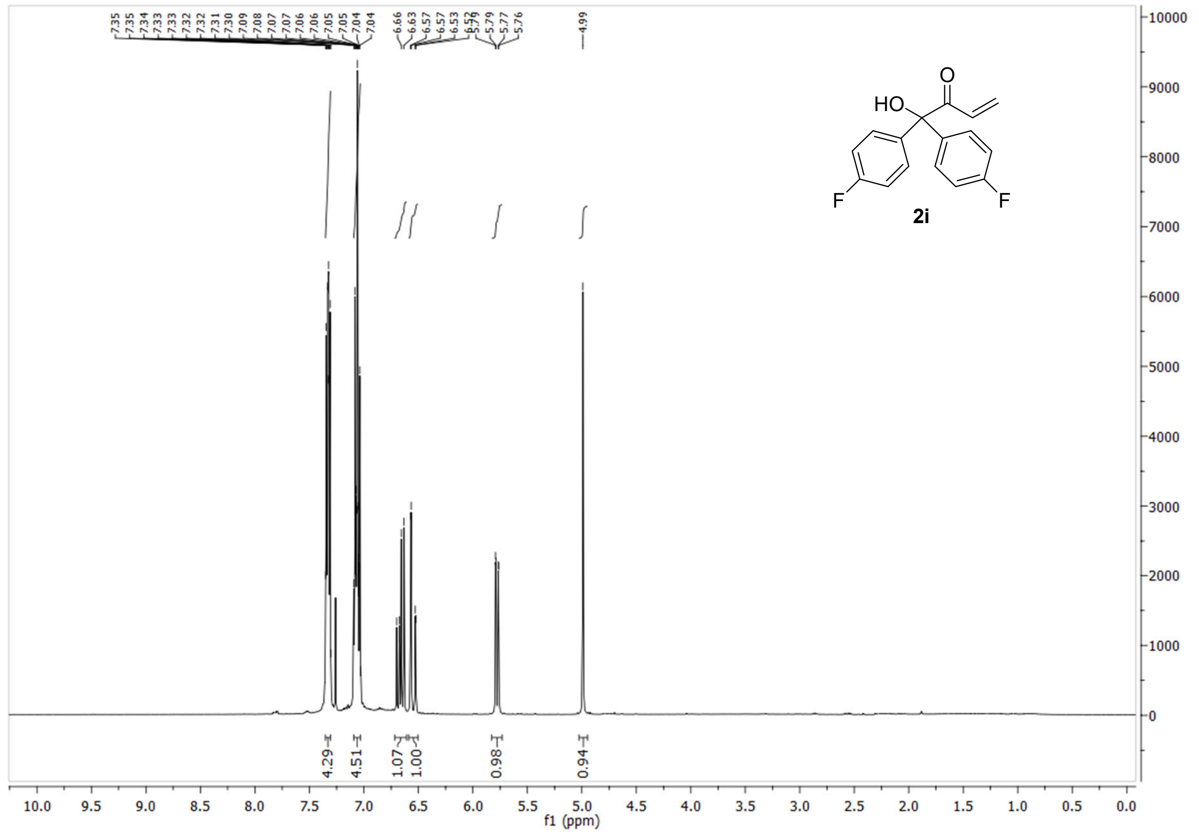

^{13}C NMR (100 MHz, $CDCl_3$) of compound **2i**:

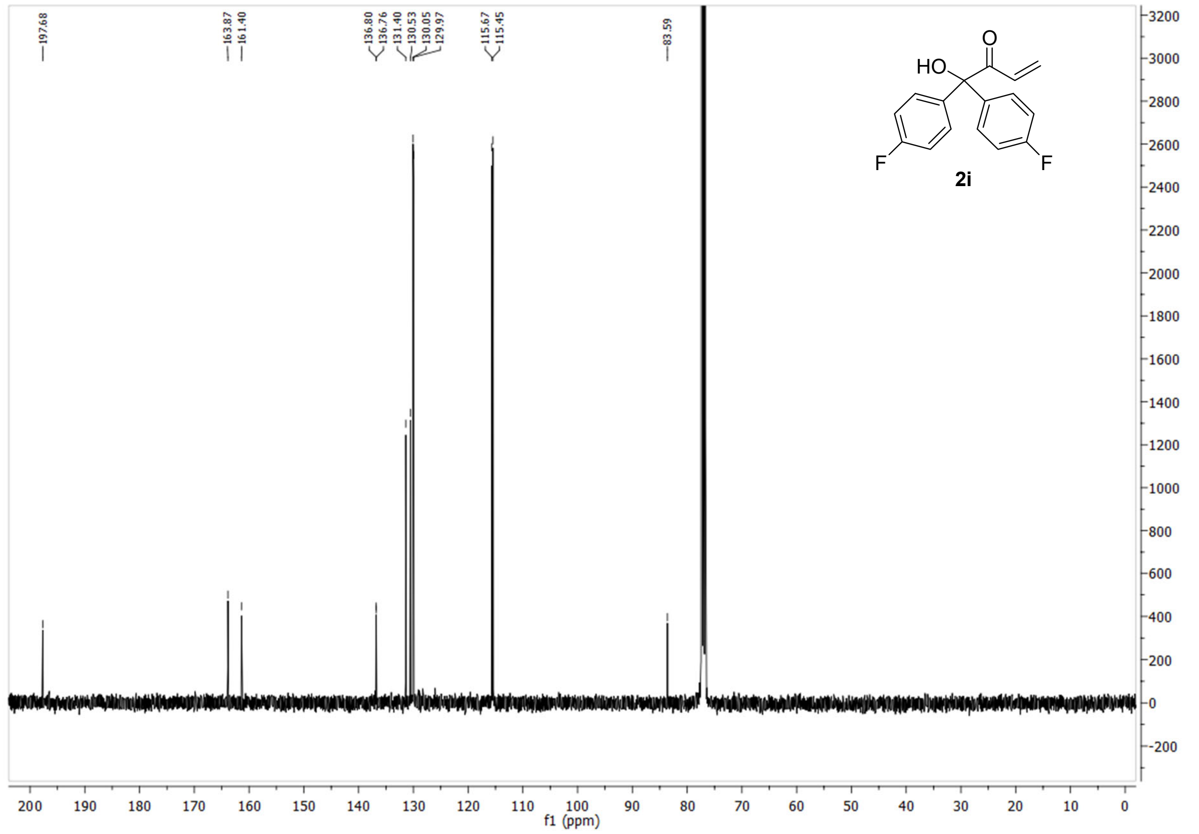

^{1}H NMR (400 MHz, $CDCl_3$) of compound **2j**:

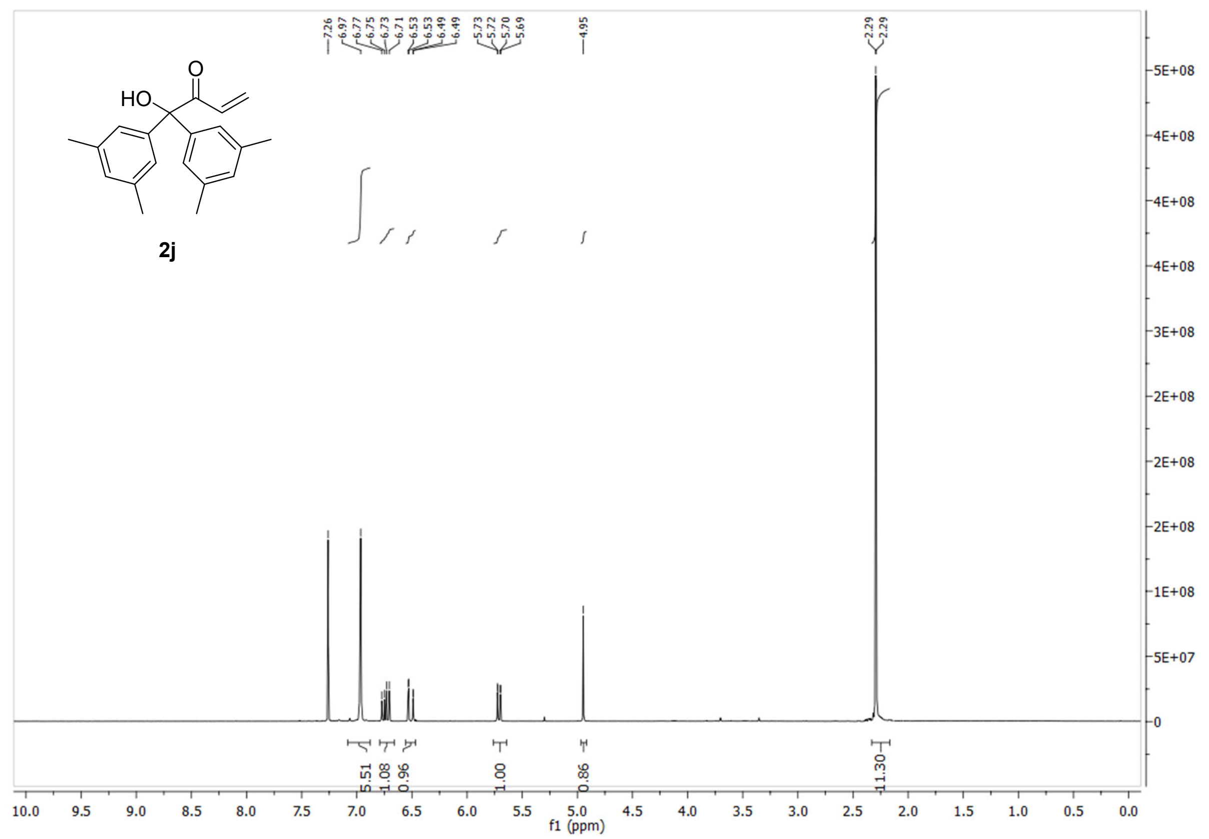

^{13}C NMR (100 MHz, $CDCl_3$) of compound **2j**:

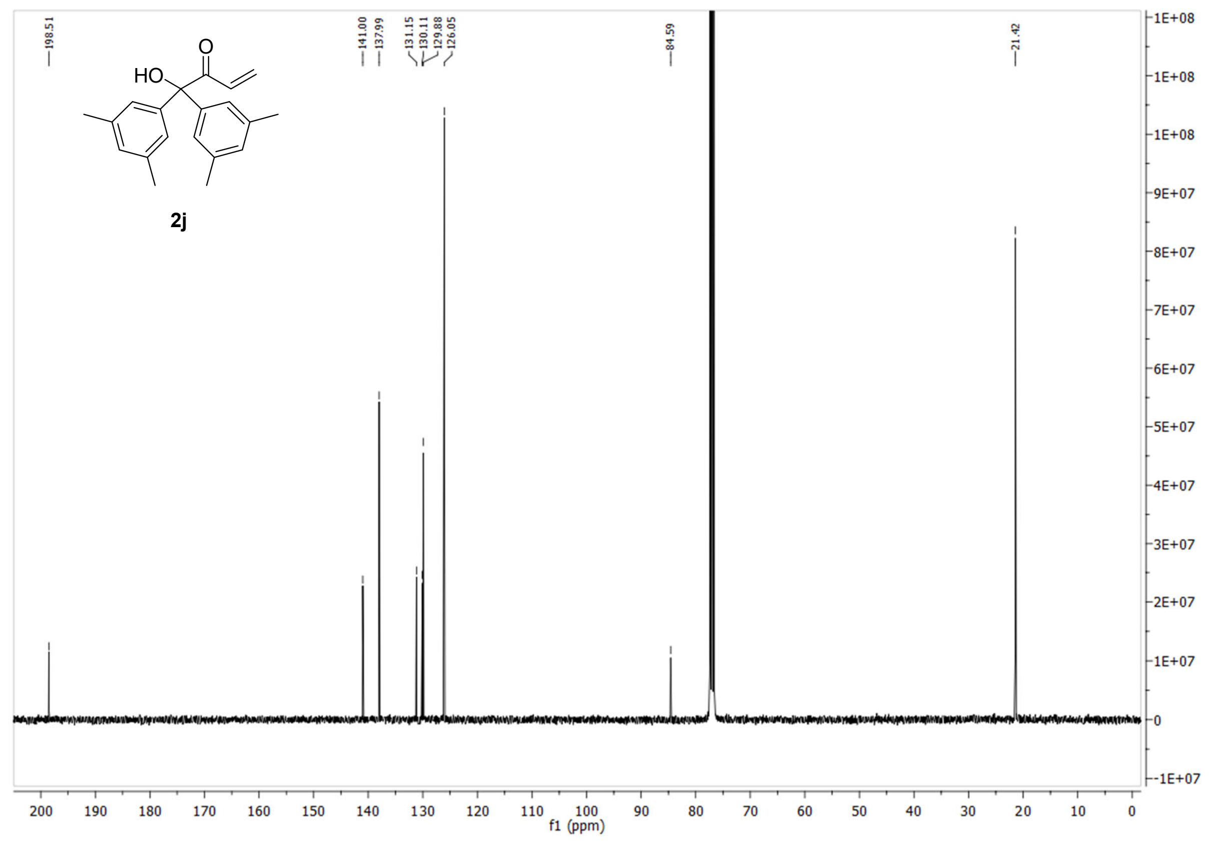

^{1}H NMR (400 MHz, $CDCl_3$) of compound **2k**:

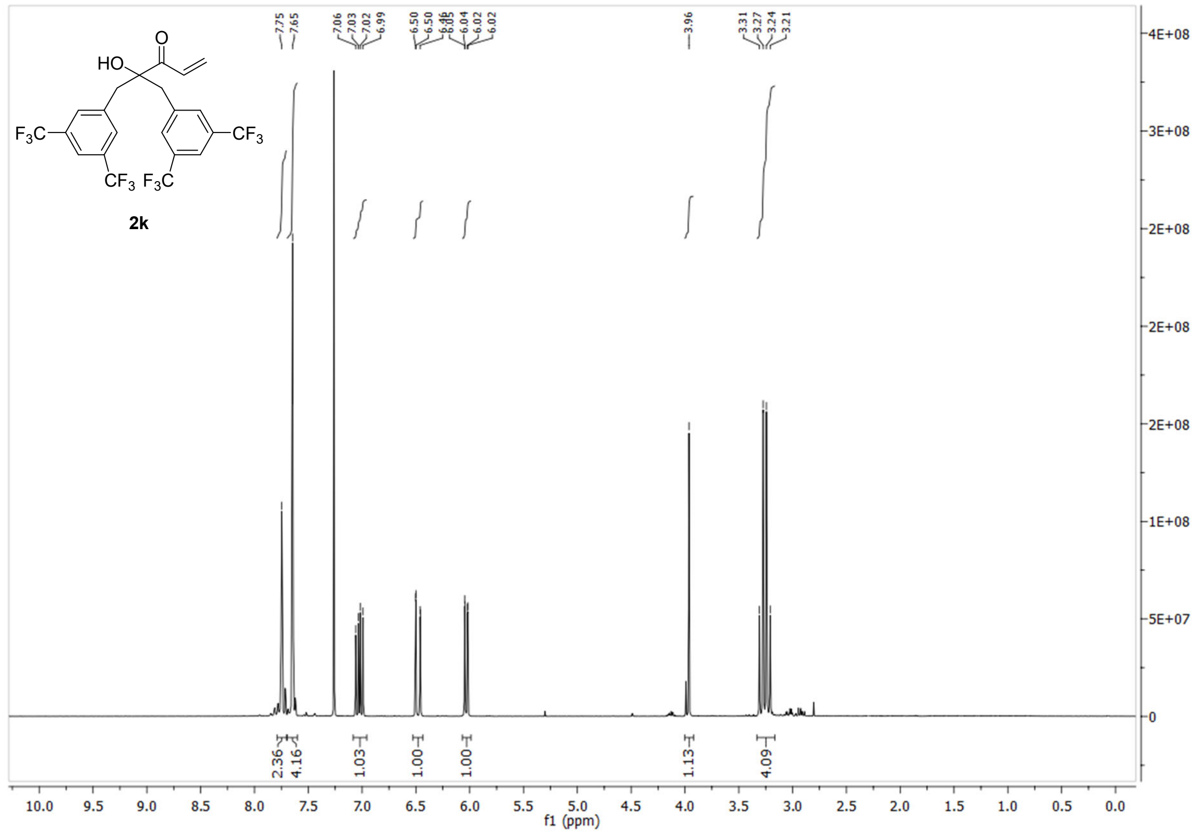

^{13}C NMR (100 MHz, $CDCl_3$) of compound **2k**:

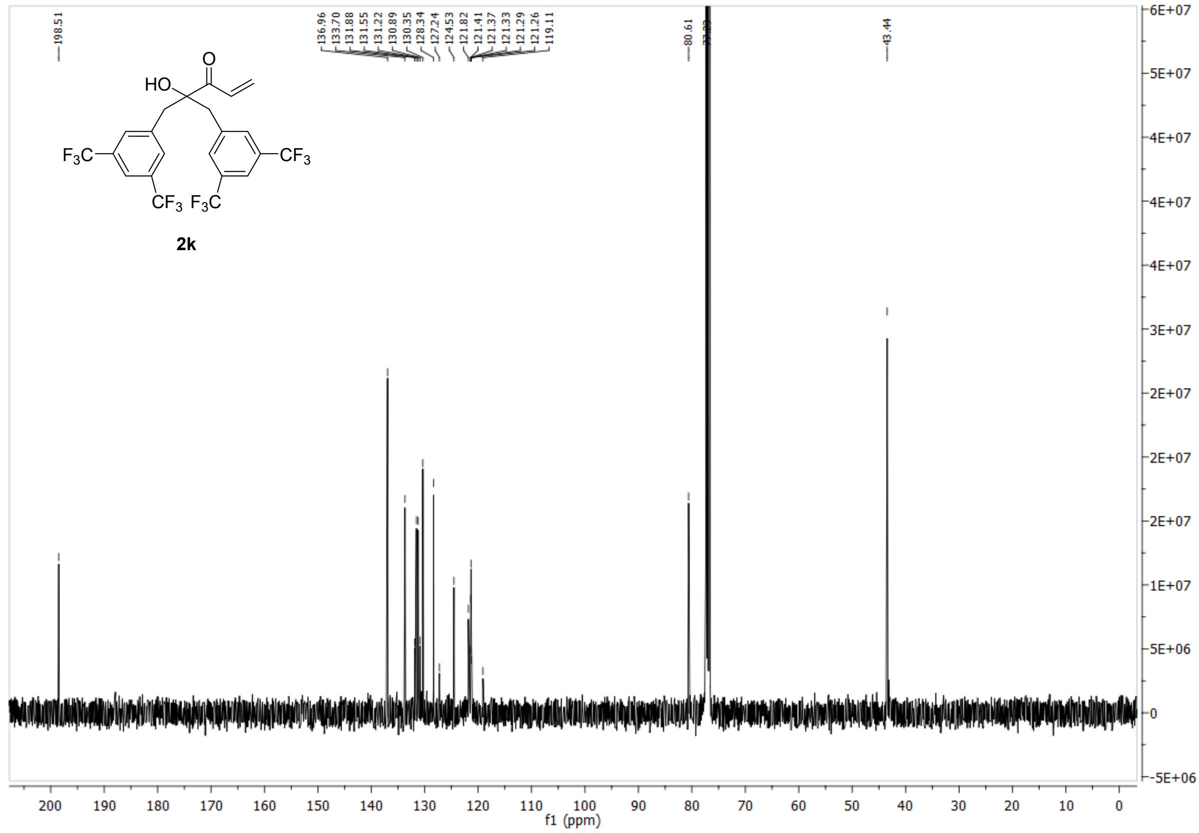

^{1}H NMR (400 MHz, $CDCl_3$) of compound **2l**:

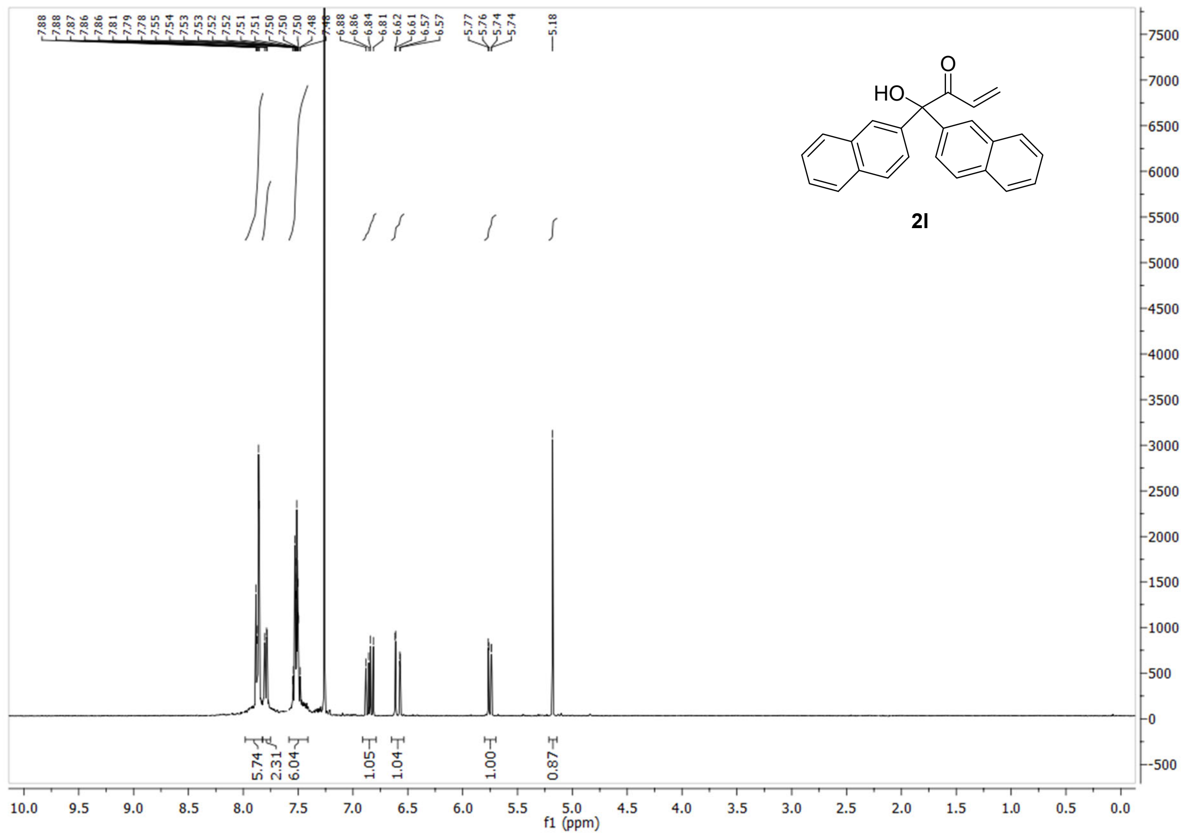

^{13}C NMR (100 MHz, $CDCl_3$) of compound **2l**:

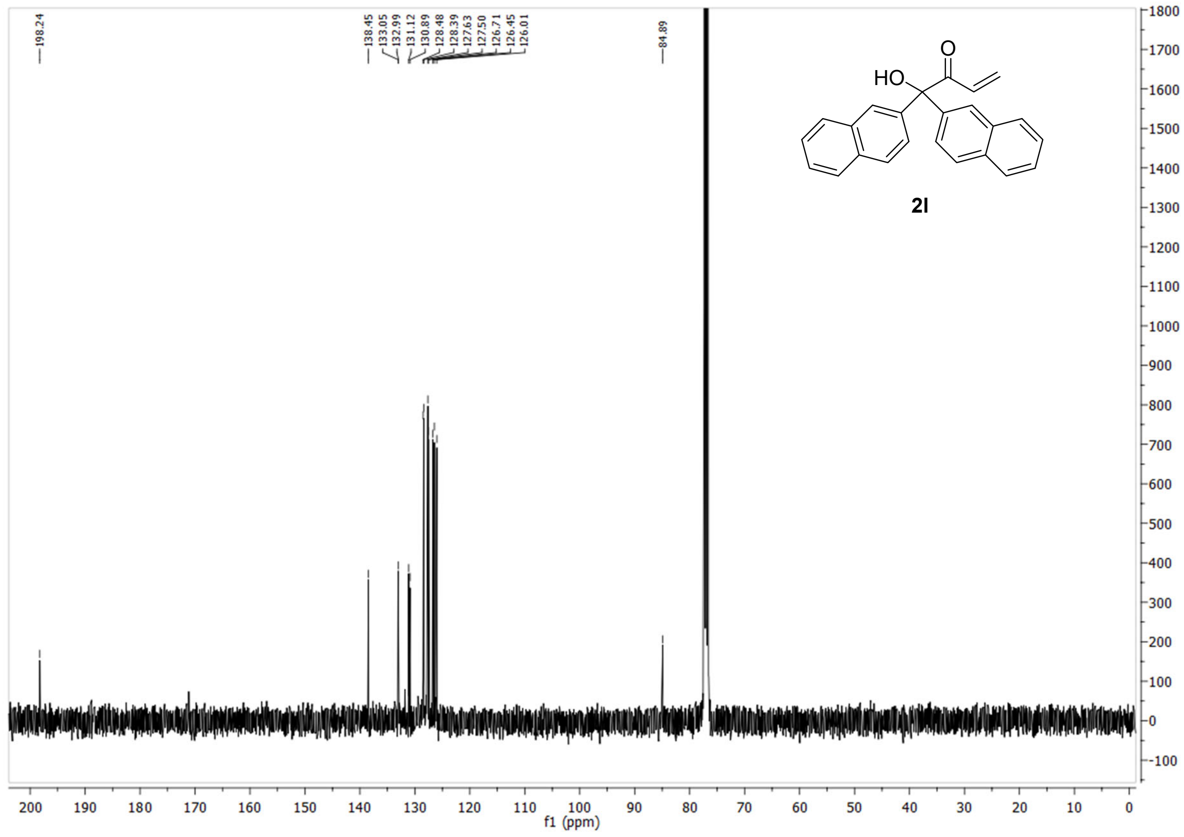

^{1}H NMR (400 MHz, $CDCl_3$) of compound **2m**:

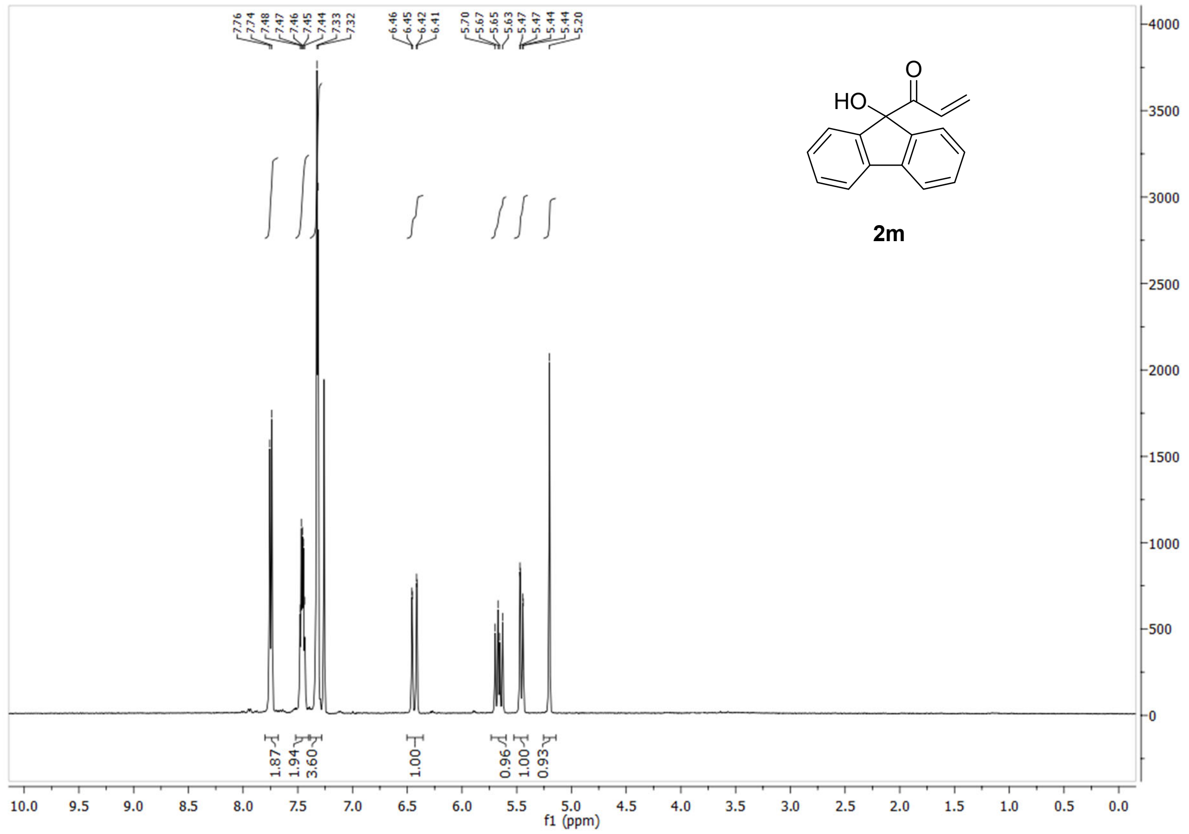

^{13}C NMR (100 MHz, $CDCl_3$) of compound **2m**:

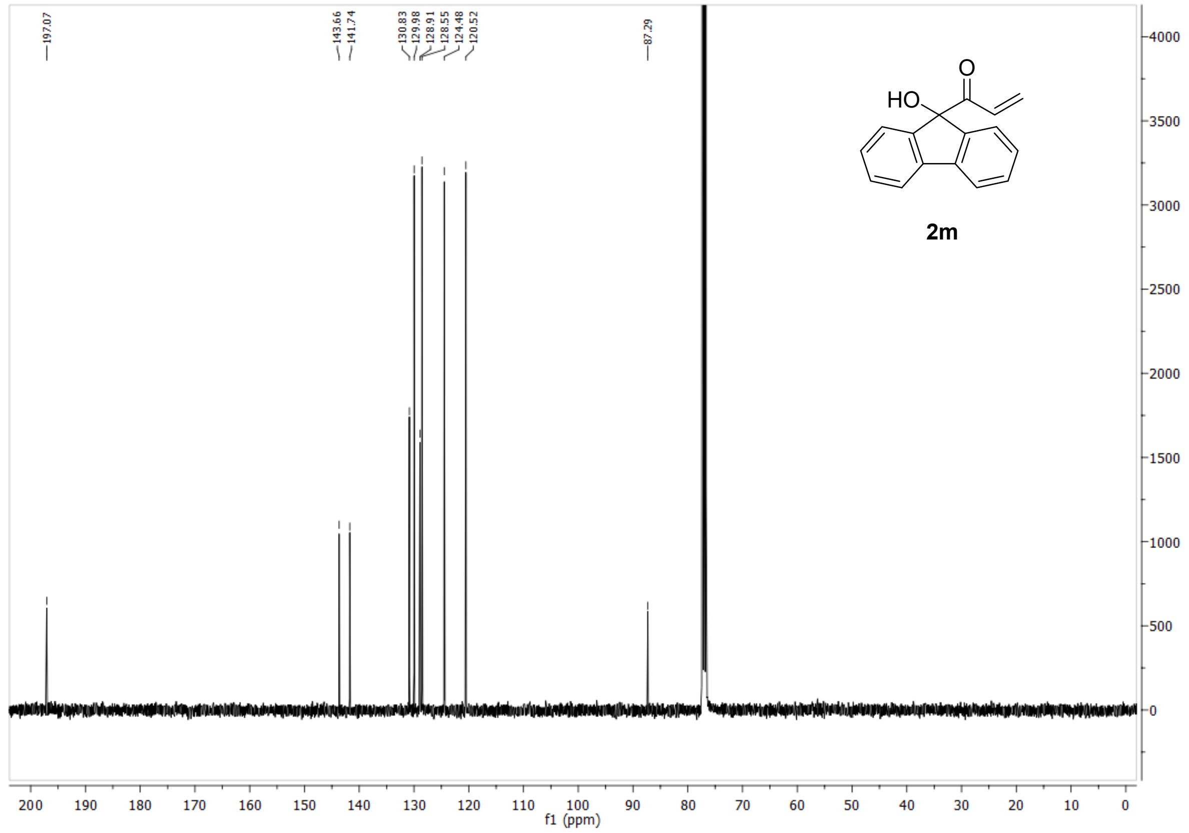

^{1}H NMR (400 MHz, $CDCl_3$) of compound **3Aa**:

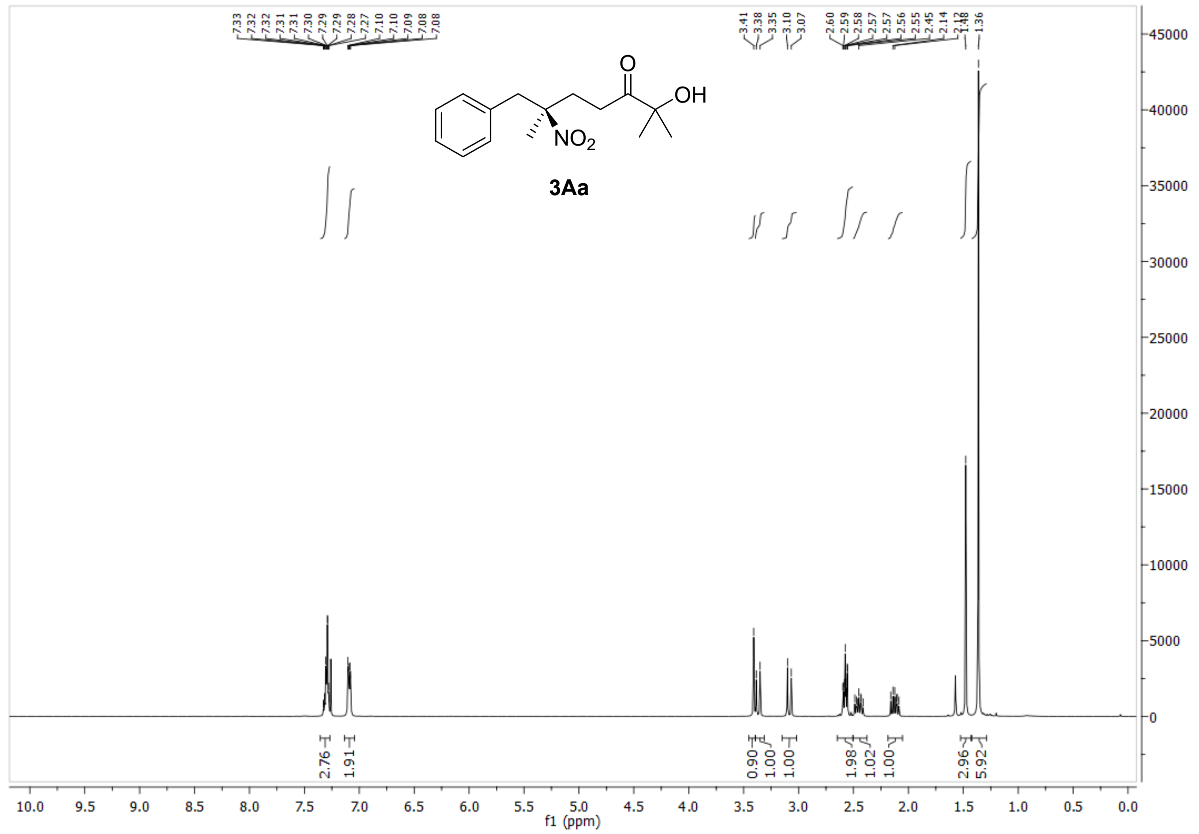

^{13}C NMR (100 MHz, $CDCl_3$) of compound **3Aa**:

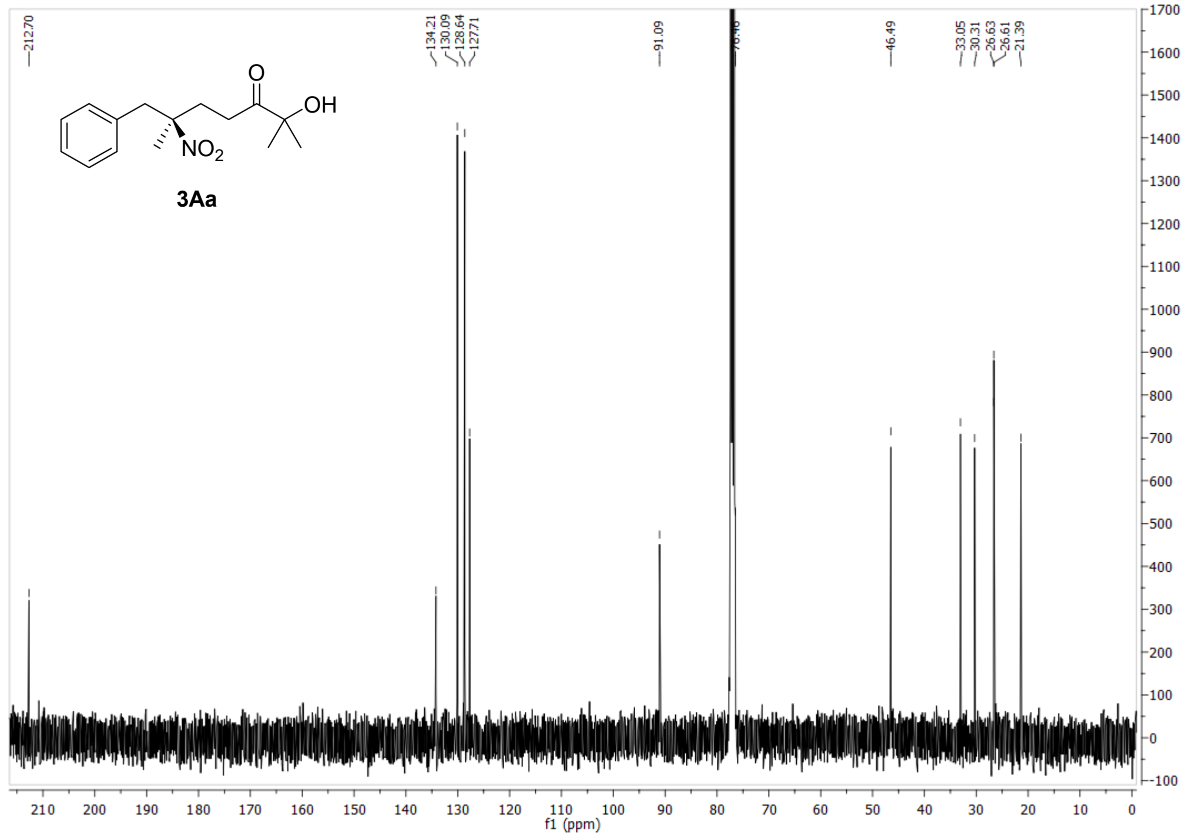

^{1}H NMR (400 MHz, $CDCl_3$) of compound **3Ab**:

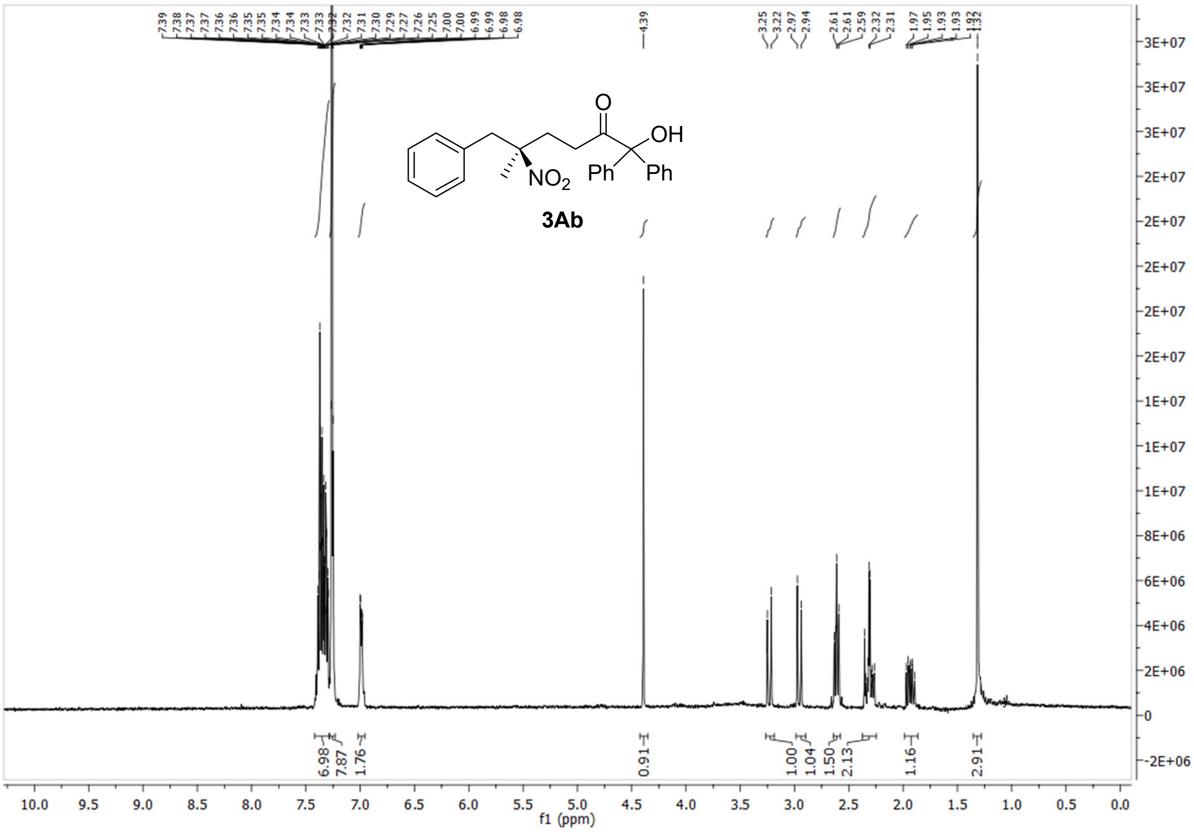

^{13}C NMR (100 MHz, $CDCl_3$) of compound **3Ab**:

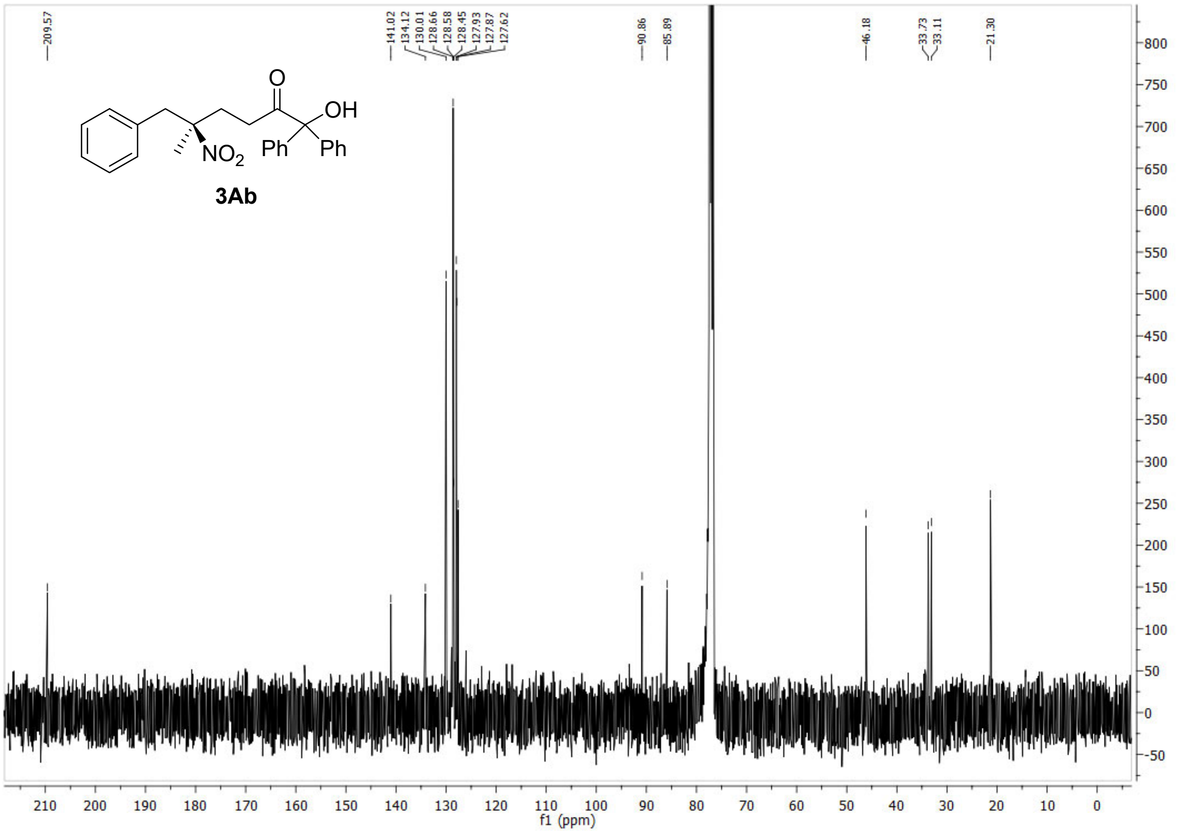

^{1}H NMR (400 MHz, $CDCl_3$) of compound **3Ac**:

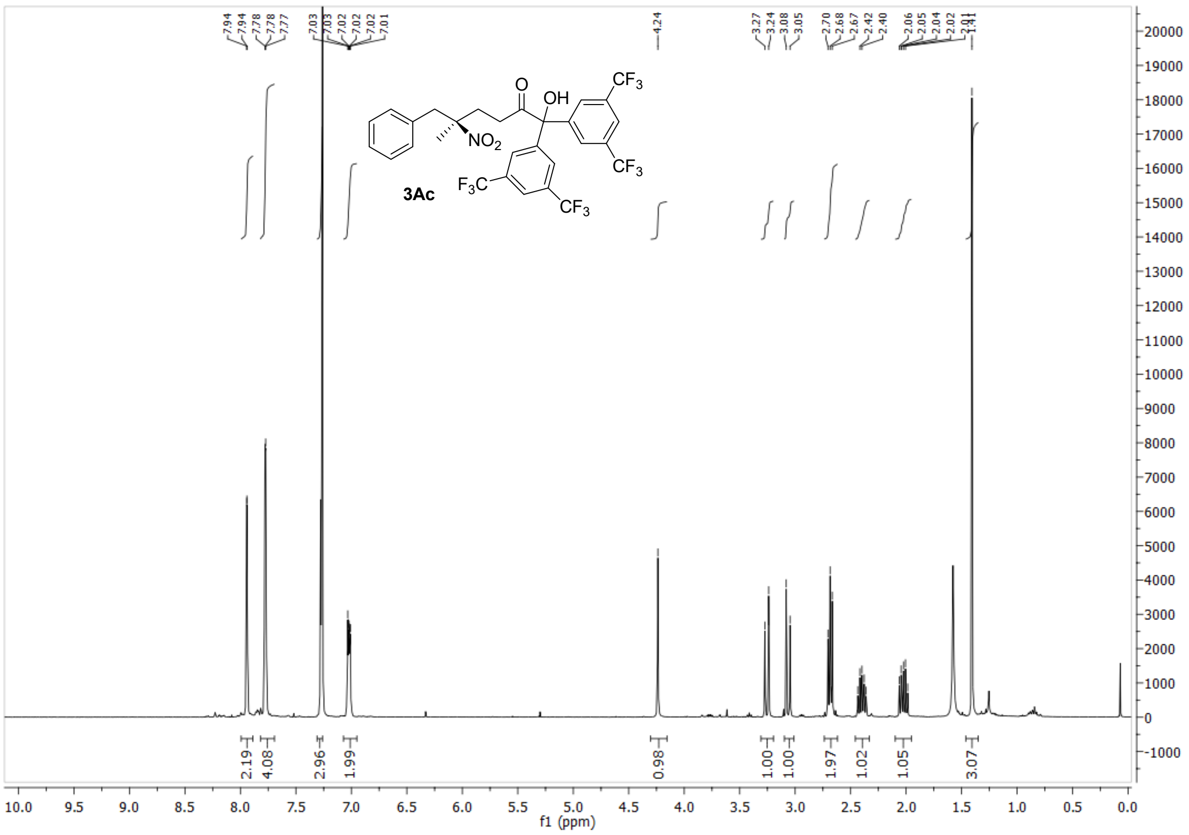

^{13}C NMR (100 MHz, $CDCl_3$) of compound **3Ac**:

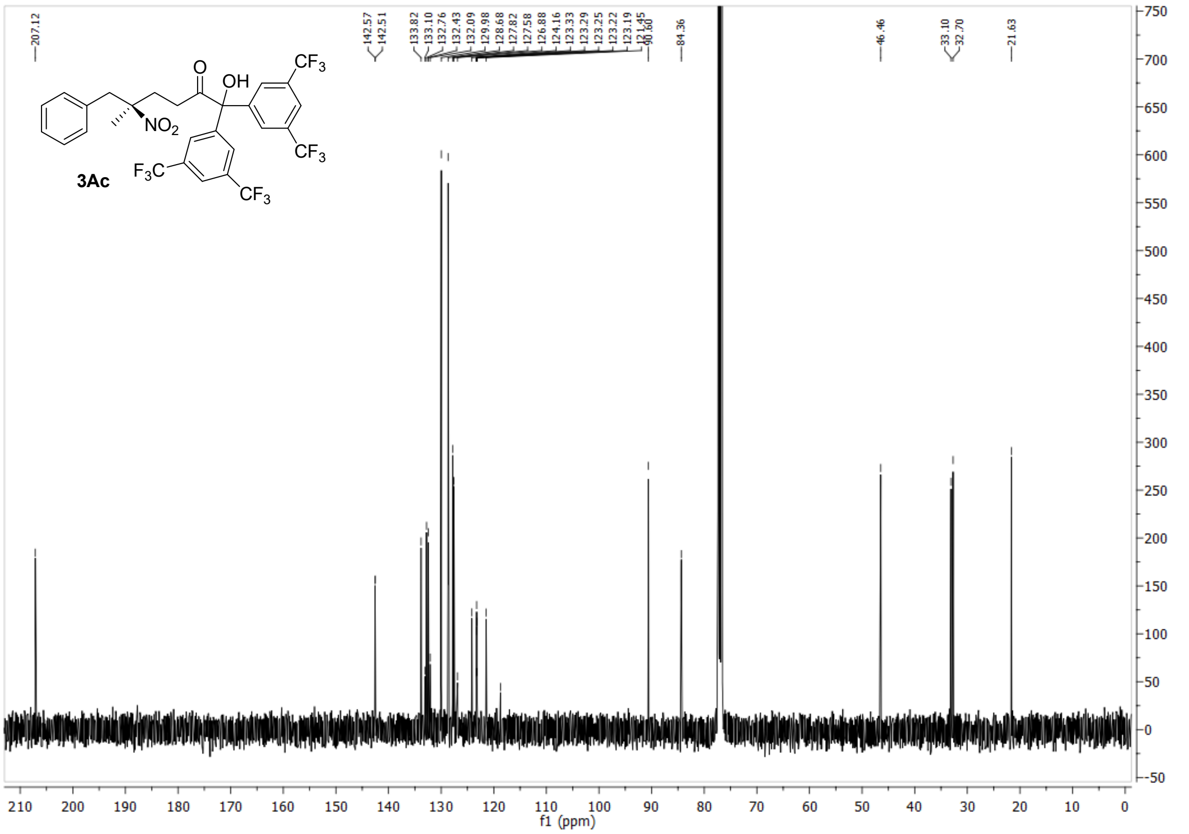

^{1}H NMR (400 MHz, $CDCl_3$) of compound **3Ad**:

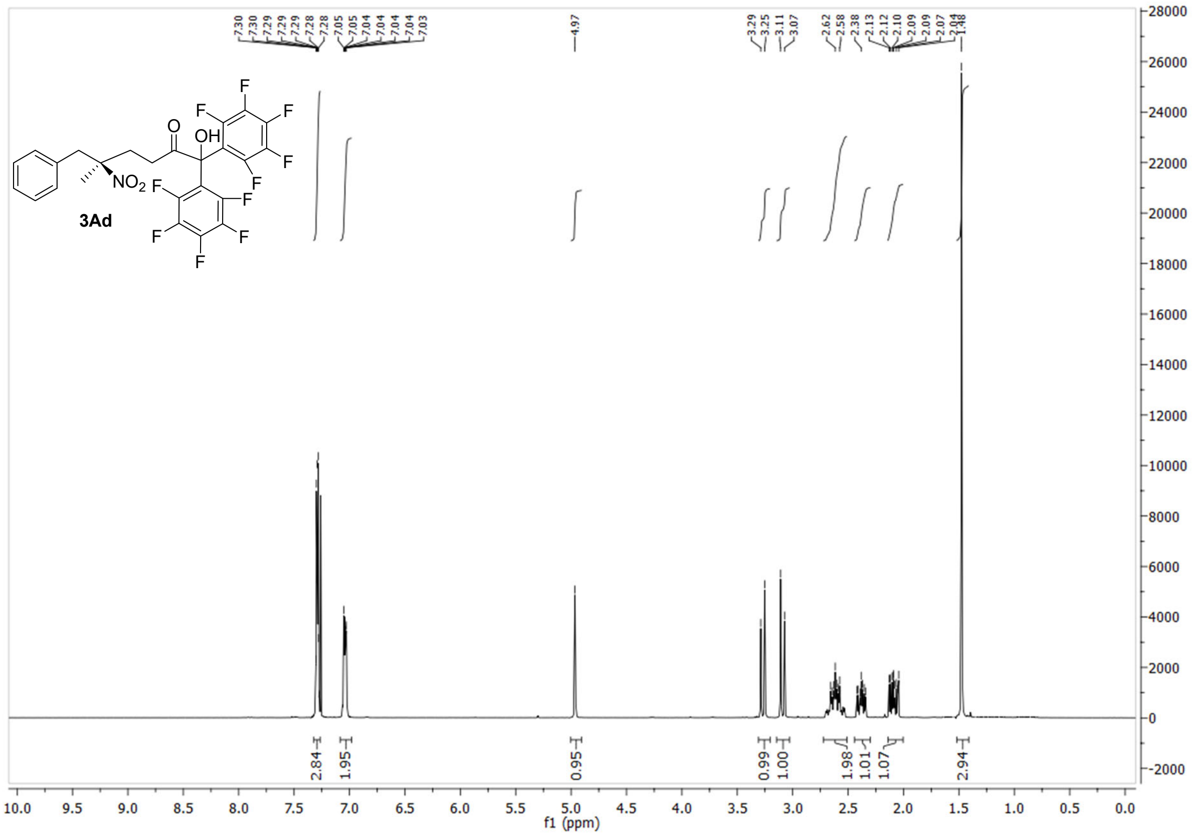

^{13}C NMR (100 MHz, $CDCl_3$) of compound **3Ad**:

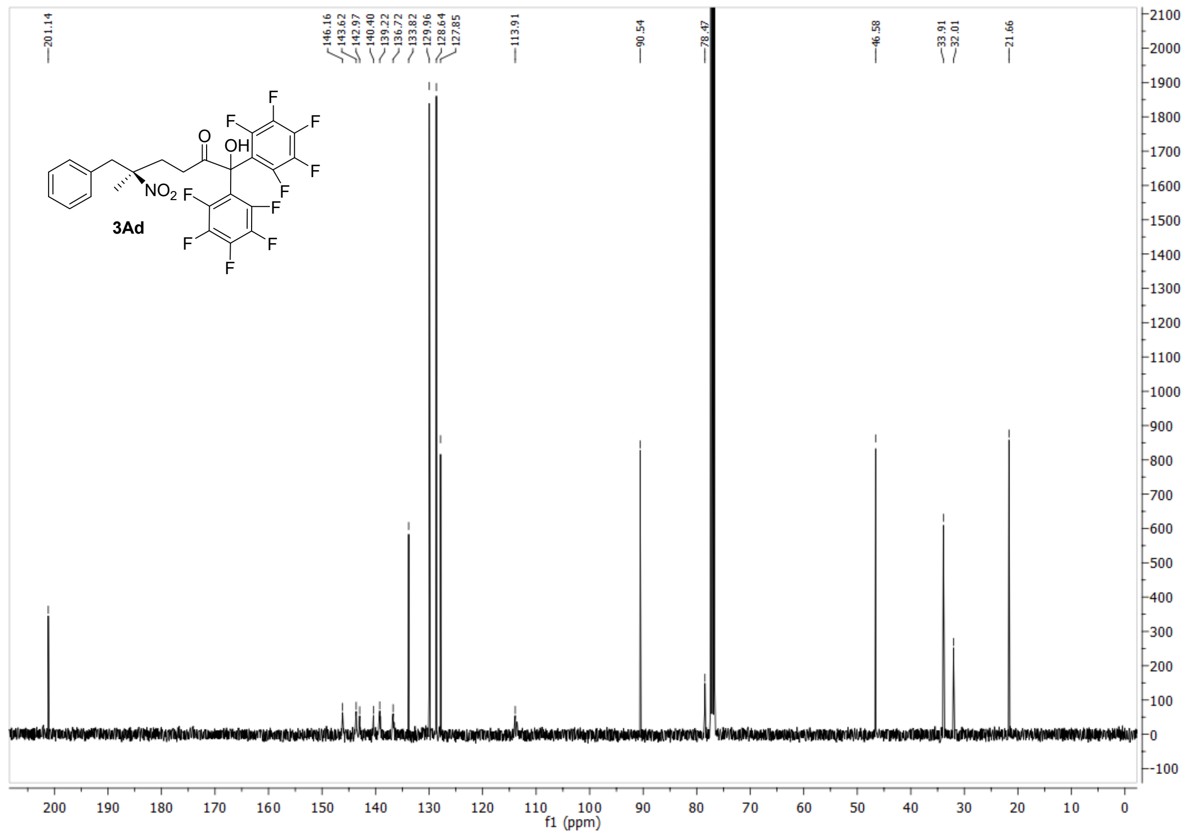

^{1}H NMR (400 MHz, $CDCl_3$) of compound **3Ae**:

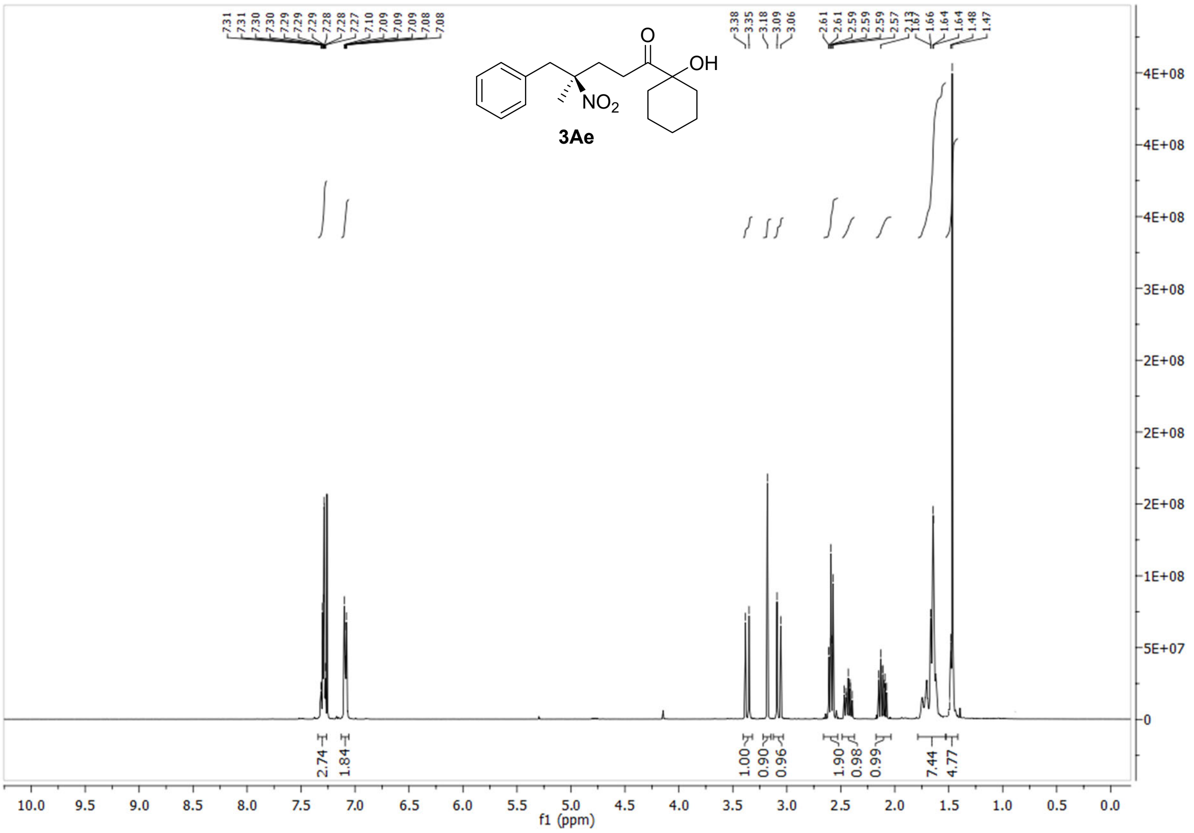

^{13}C NMR (100 MHz, $CDCl_3$) of compound **3Ae**:

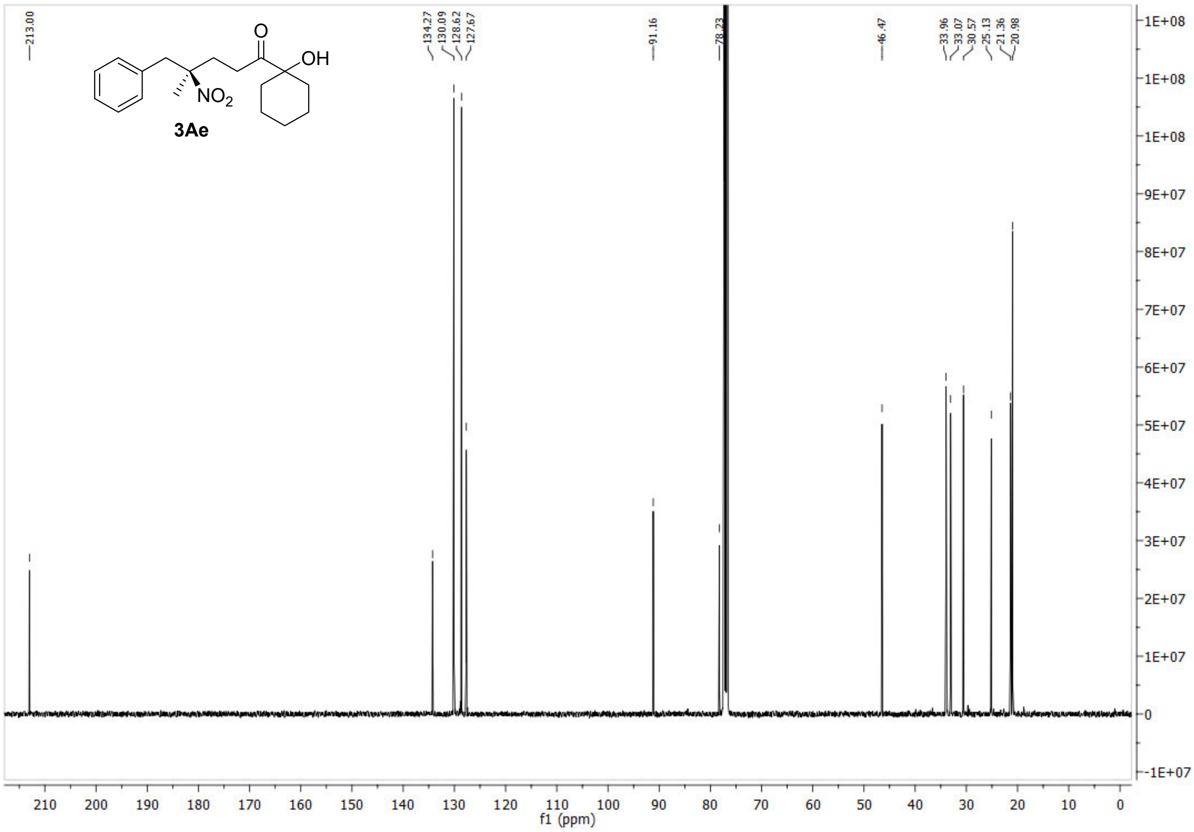

^{1}H NMR (400 MHz, $CDCl_3$) of compound **3Af**:

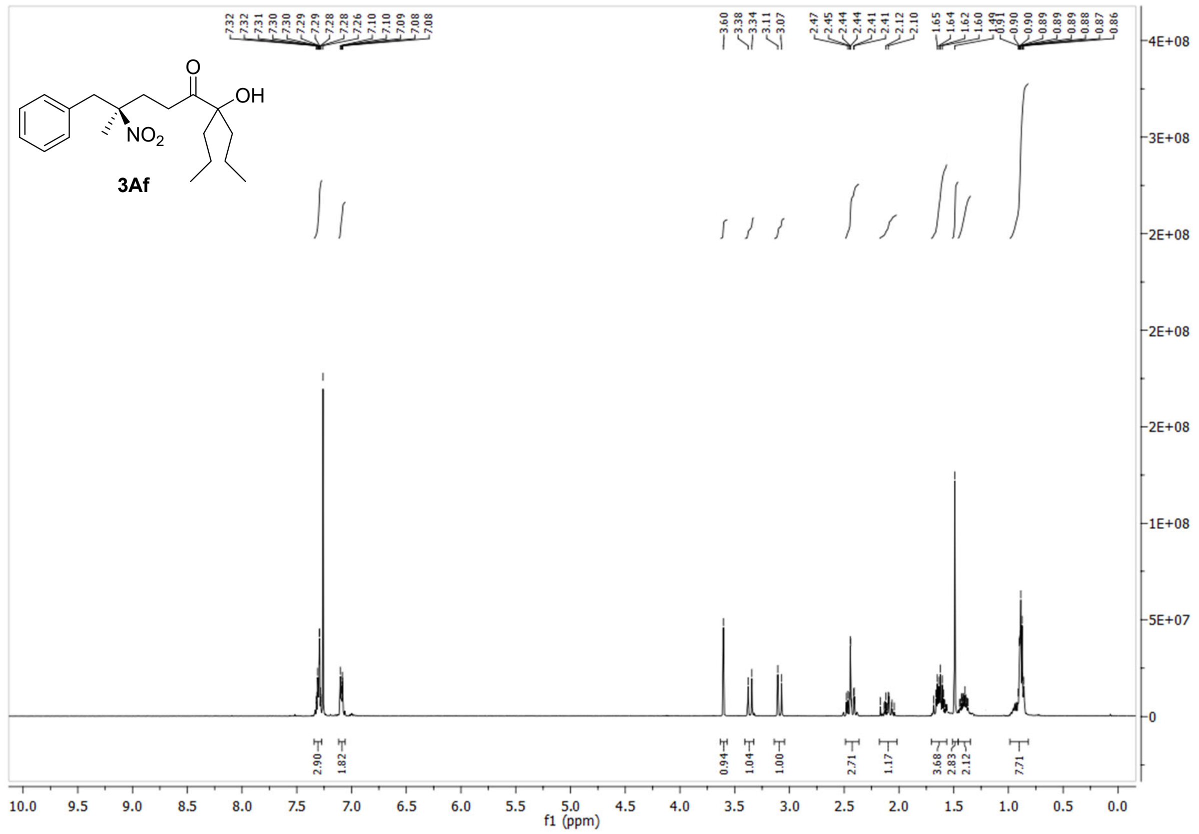

^{13}C NMR (100 MHz, $CDCl_3$) of compound **3Af**:

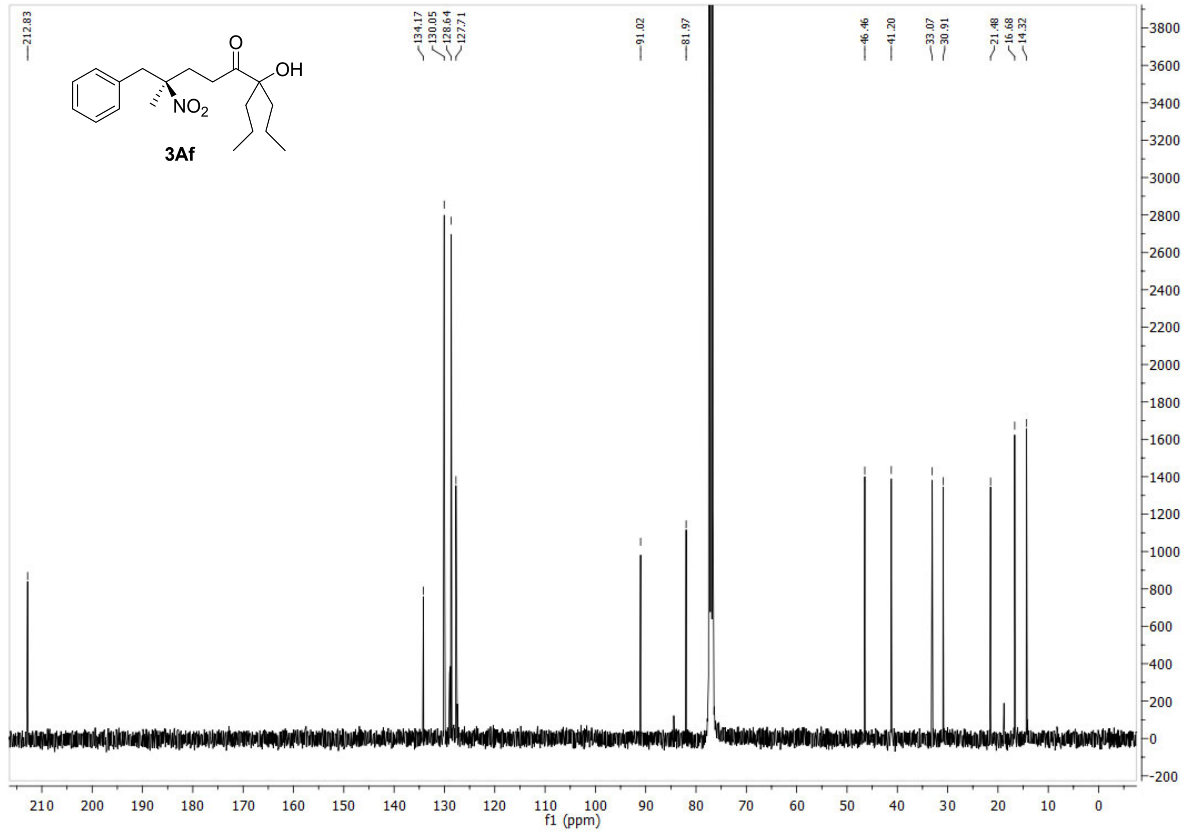

^{1}H NMR (400 MHz, $CDCl_3$) of compound **3Ag**:

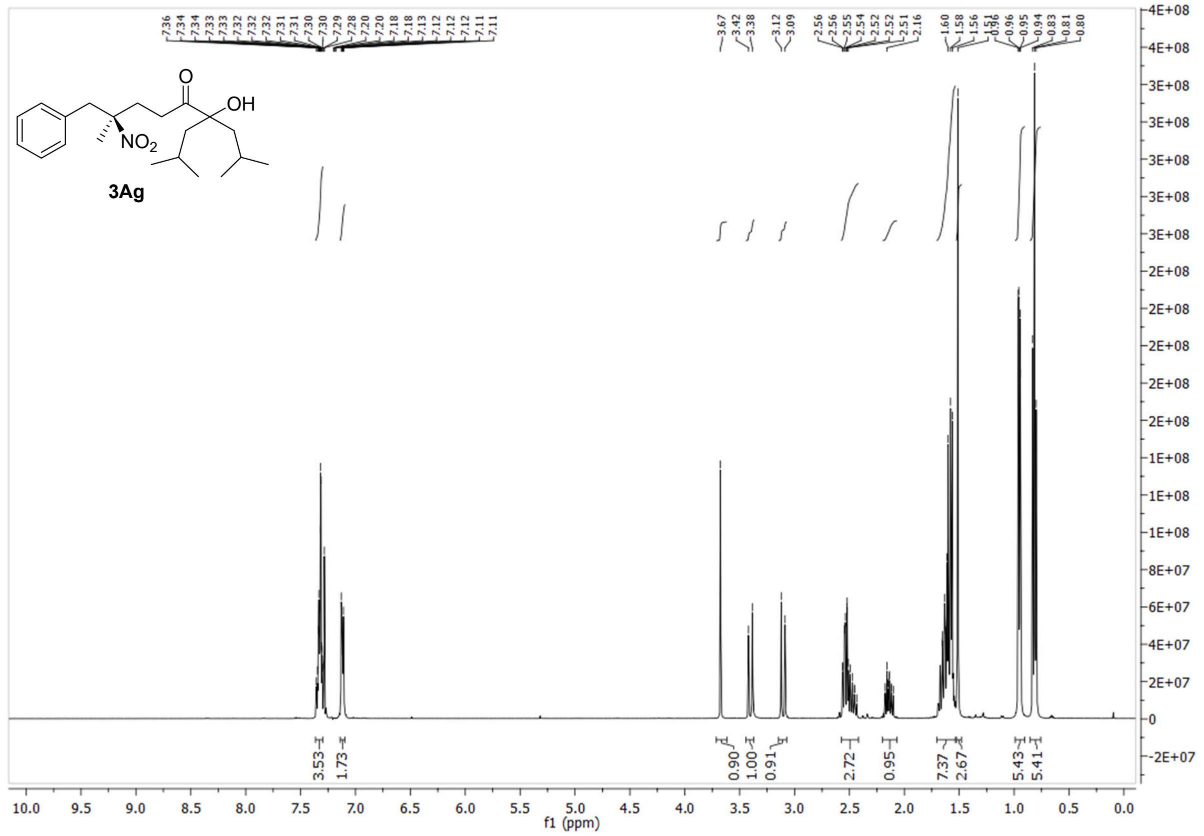

^{13}C NMR (100 MHz, $CDCl_3$) of compound **3Ag**:

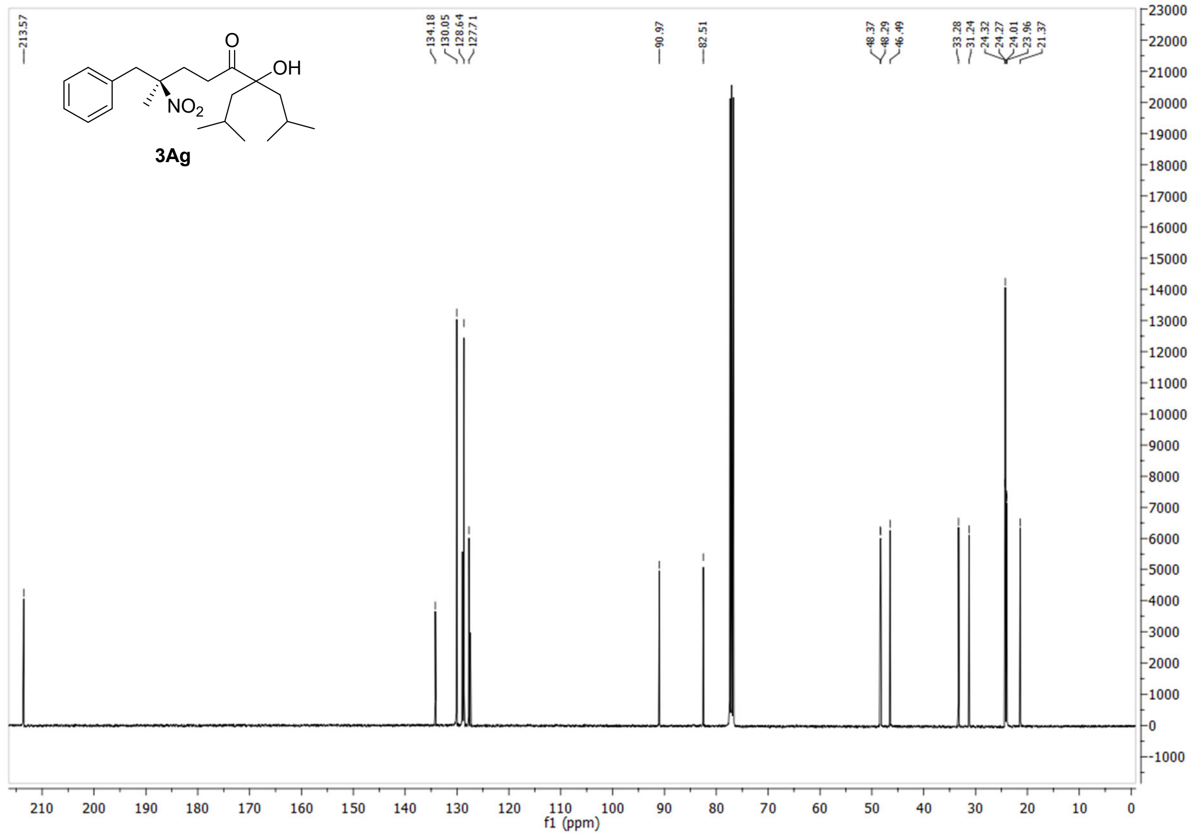

^{1}H NMR (300 MHz, $CDCl_3$) of compound **3Ah**:

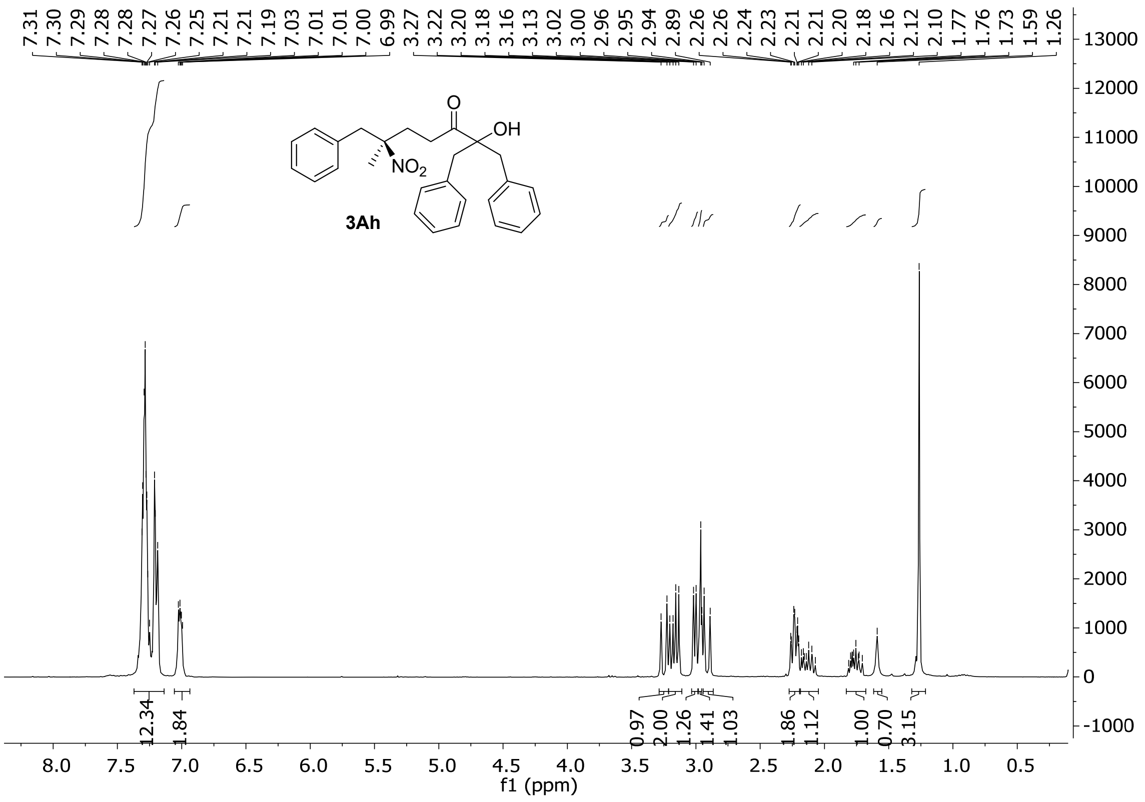

^{13}C NMR (75 MHz, $CDCl_3$) of compound **3Ah**:

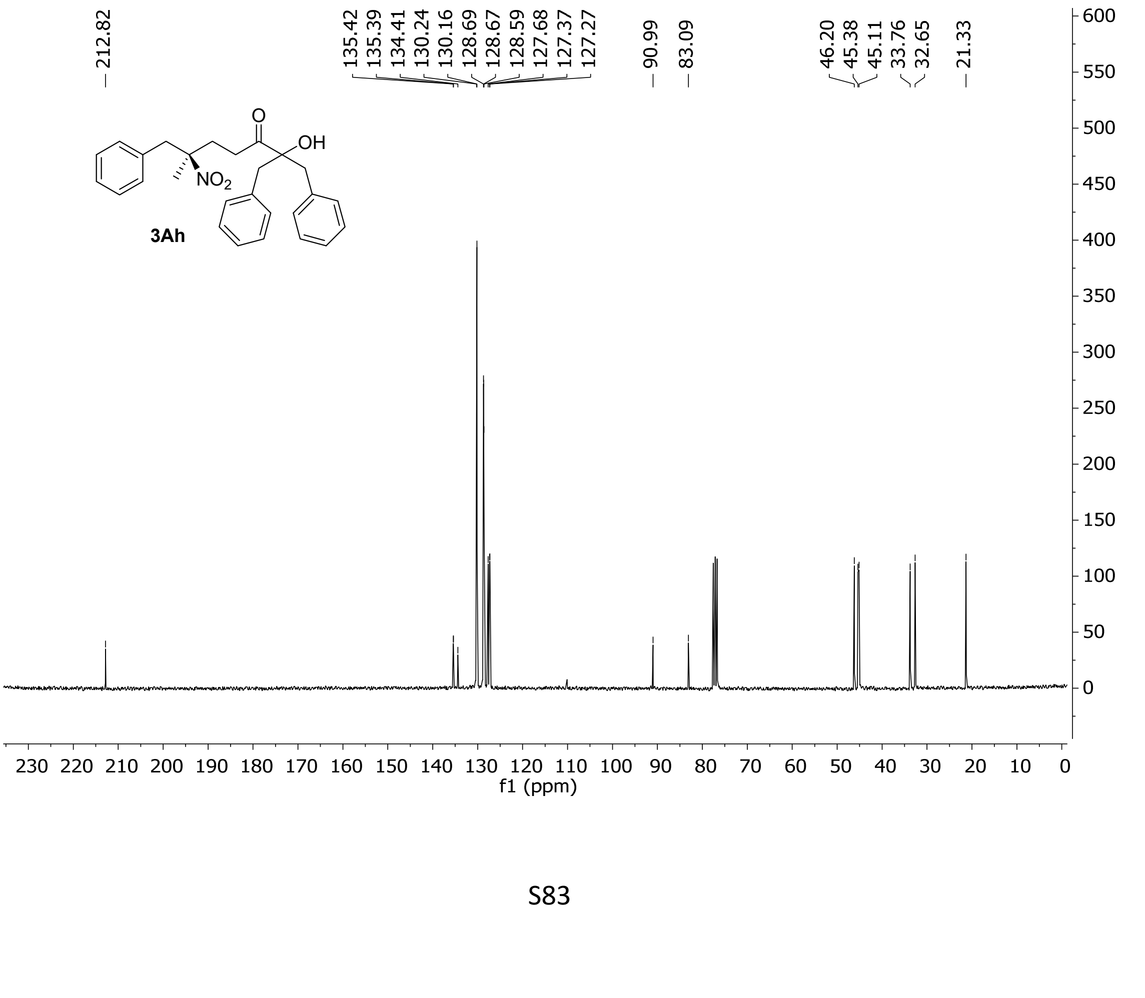

^{1}H NMR (400 MHz, $CDCl_3$) of compound **3Ai**:

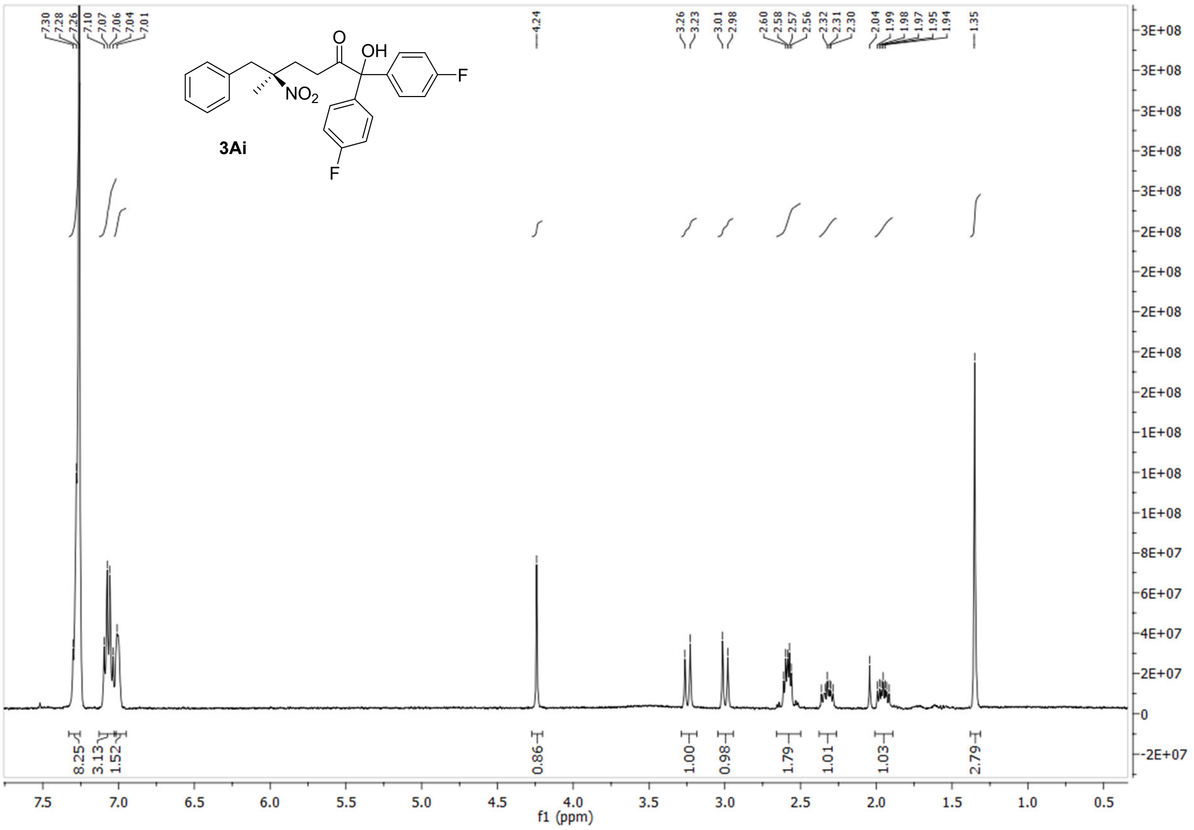

^{13}C NMR (100 MHz, $CDCl_3$) of compound **3Ai**:

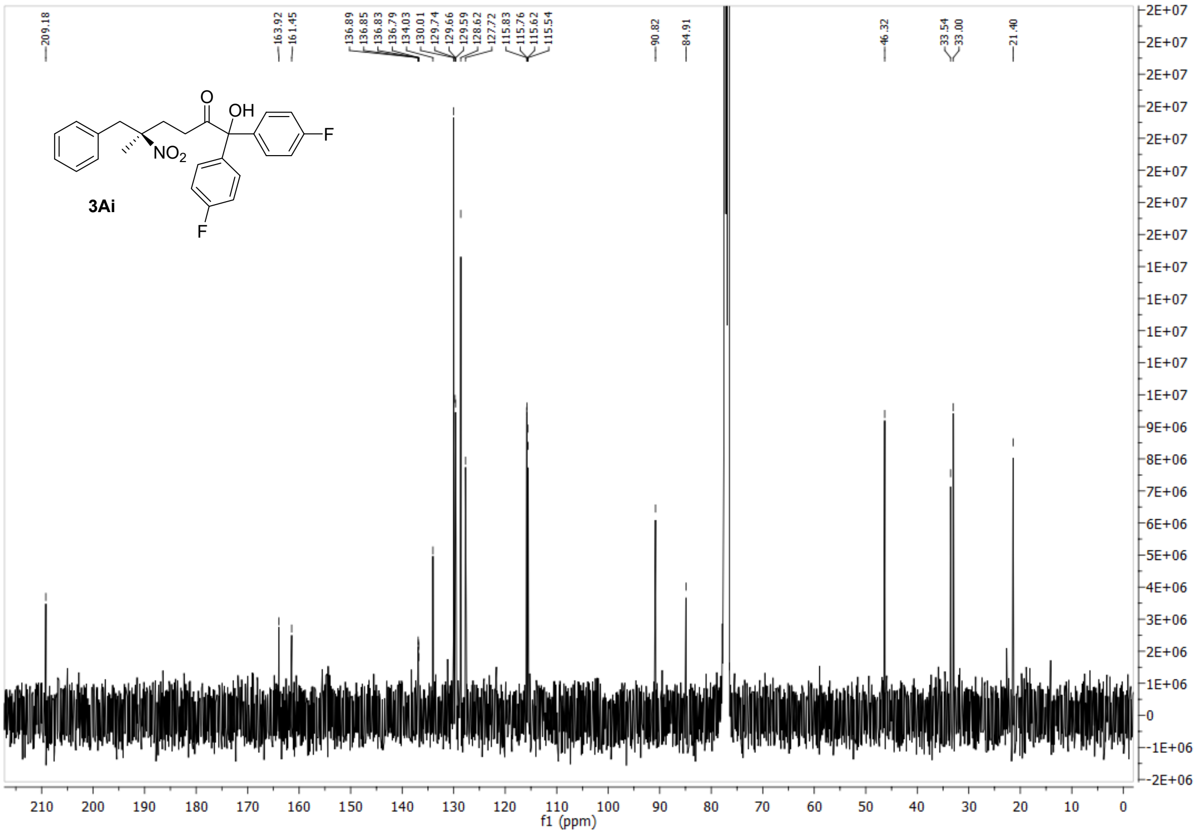

^{1}H NMR (400 MHz, $CDCl_3$) of compound **3Aj**:

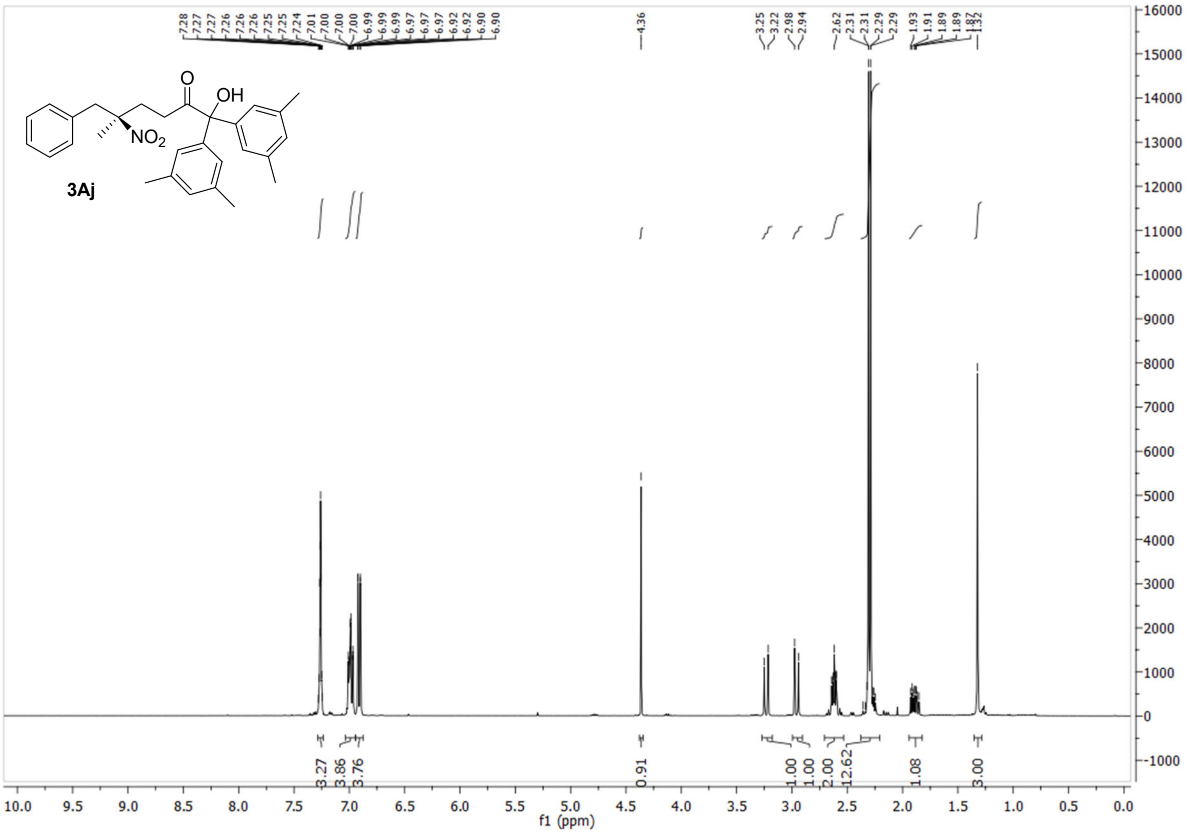

^{13}C NMR (100 MHz, $CDCl_3$) of compound **3Aj**:

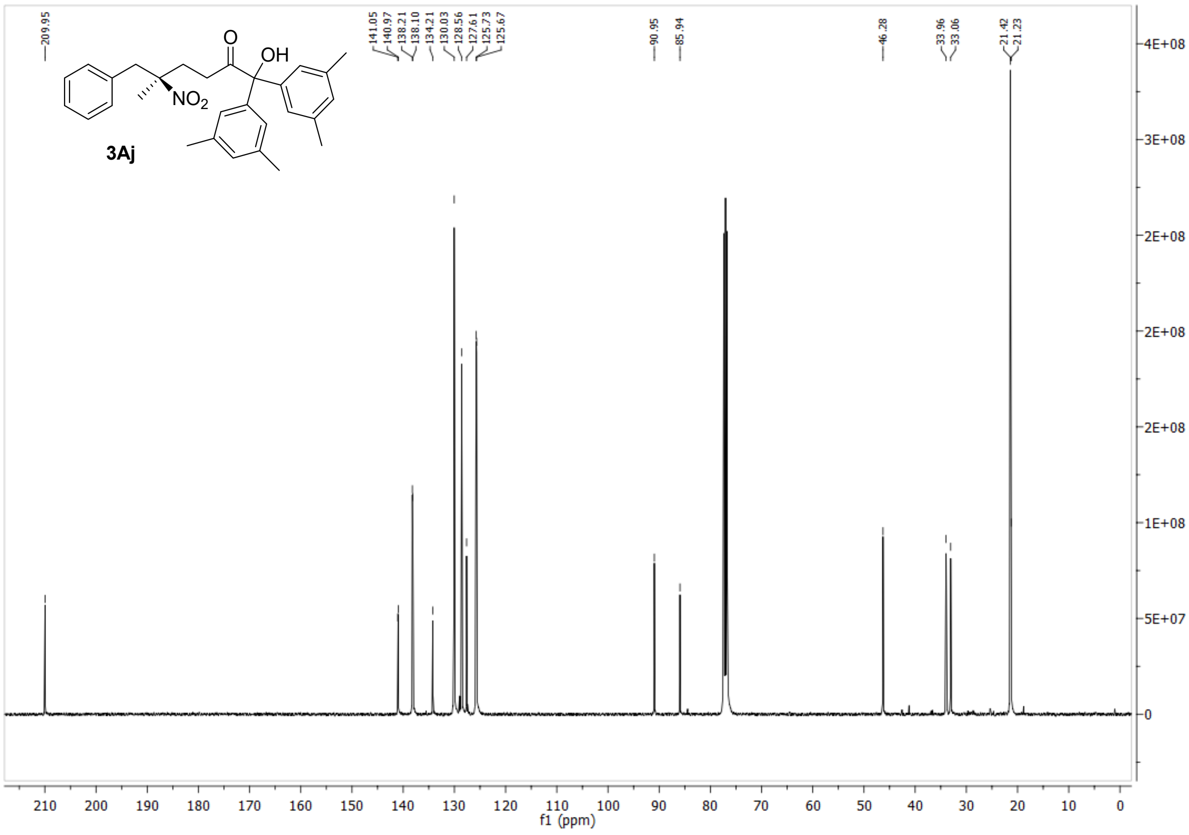

^{1}H NMR (400 MHz, $CDCl_3$) of compound **3Ak**:

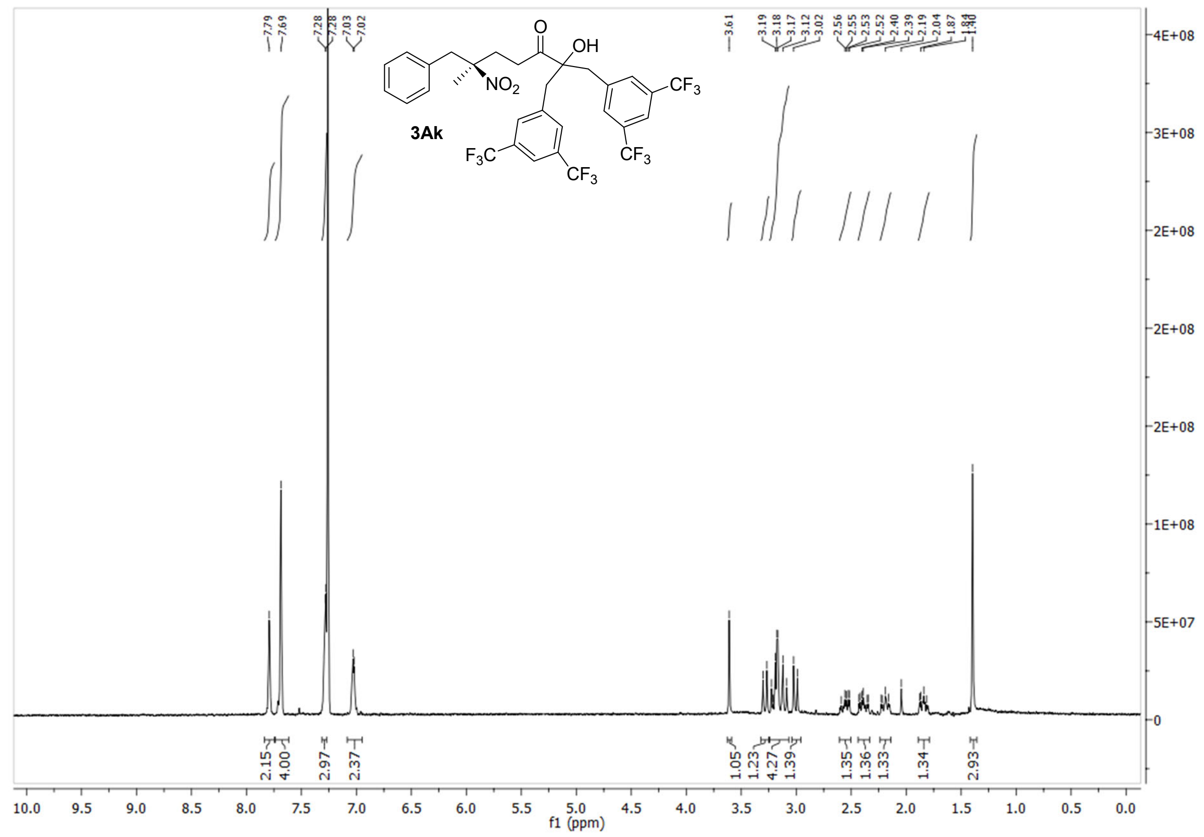

^{13}C NMR (100 MHz, $CDCl_3$) of compound **3Ak**:

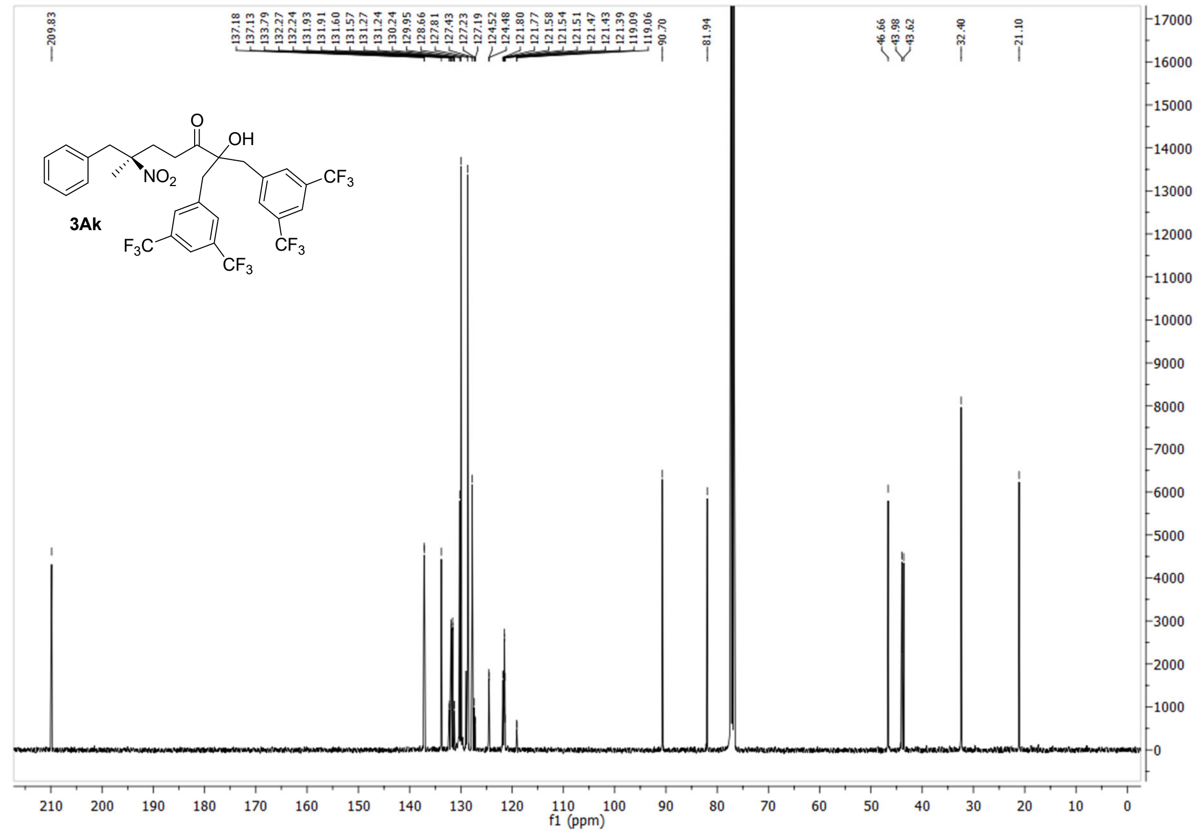

^{1}H NMR (400 MHz, $CDCl_3$) of compound **3Al**:

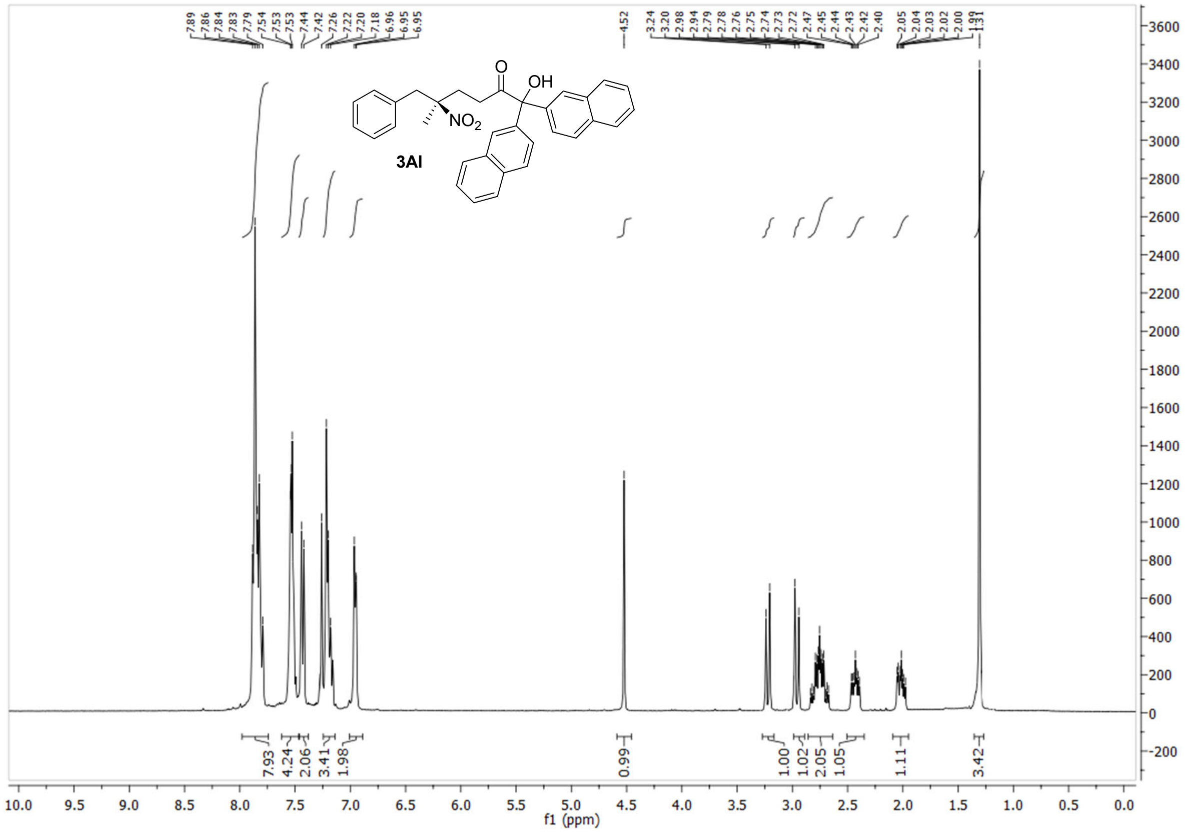

^{13}C NMR (100 MHz, $CDCl_3$) of compound **3Al**:

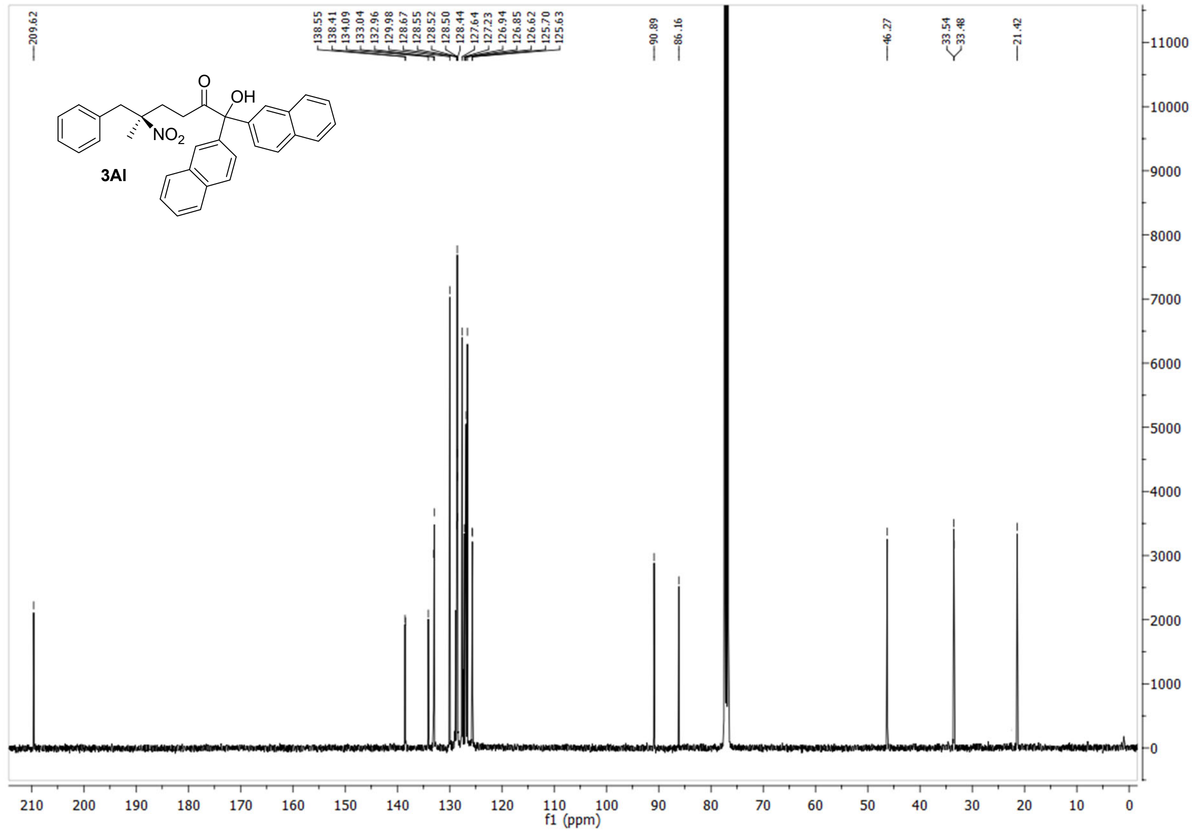

^{1}H NMR (400 MHz, $CDCl_3$) of compound **3Am**:

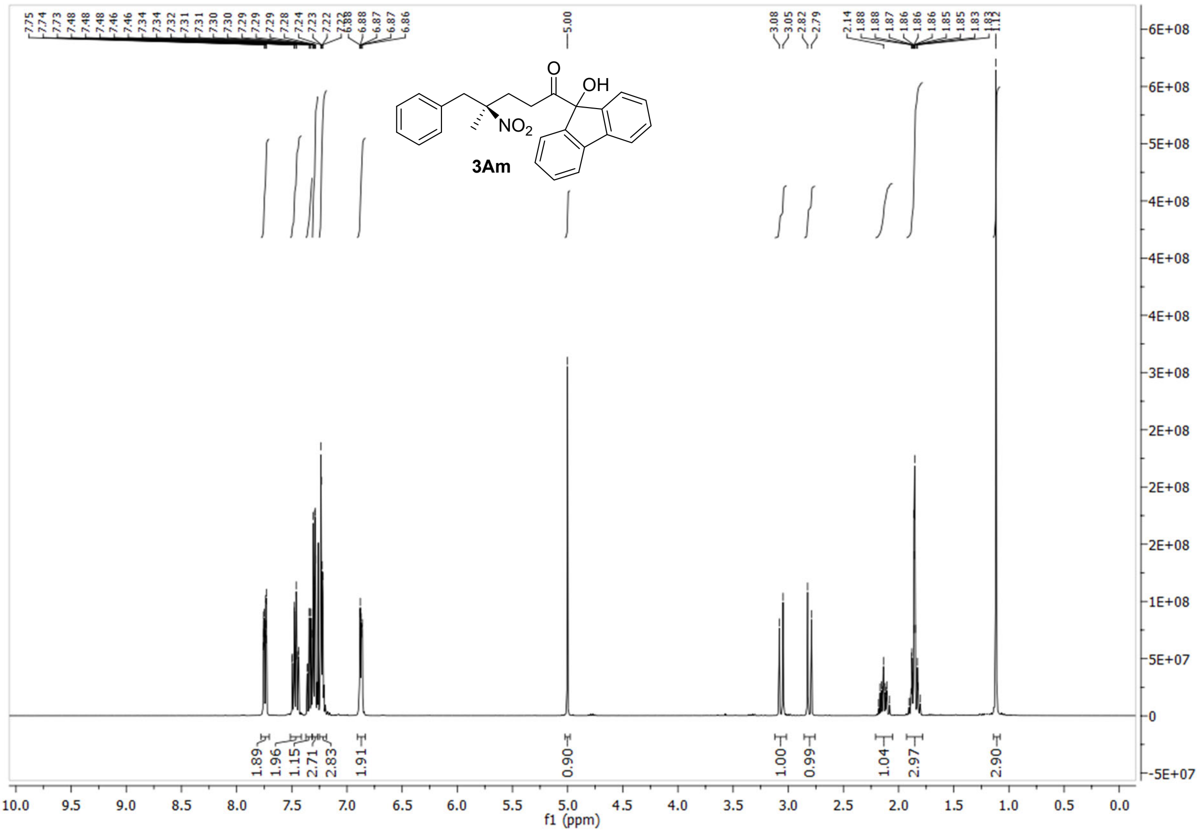

^{13}C NMR (100 MHz, $CDCl_3$) of compound **3Am**:

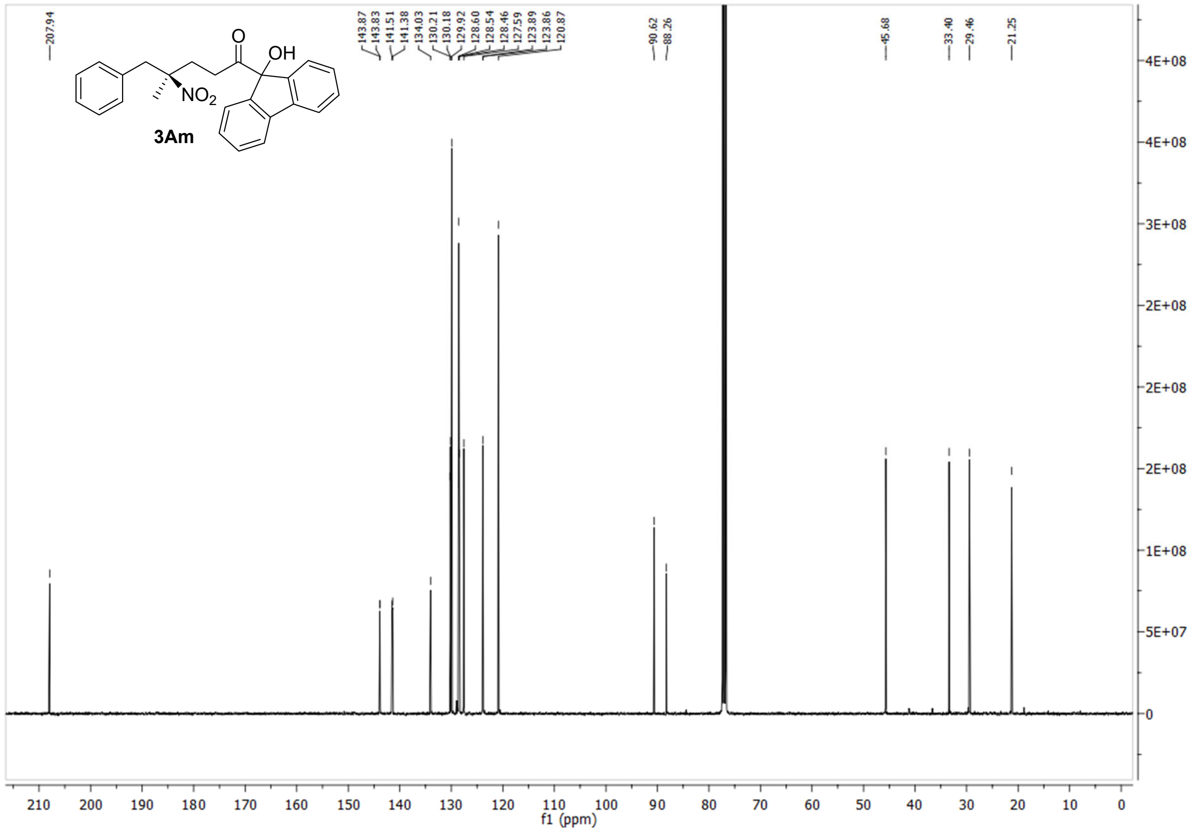

^{1}H NMR (400 MHz, $CDCl_3$) of compound **3Bc**:

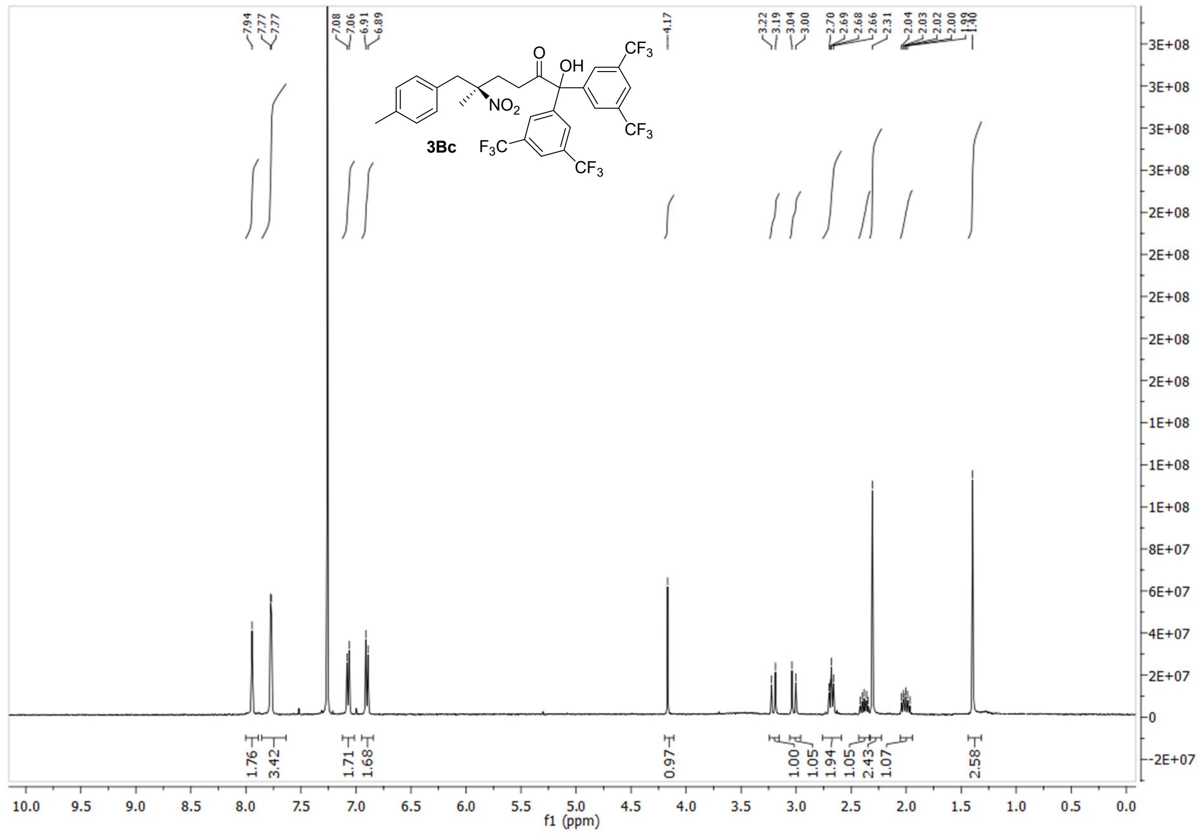

^{13}C NMR (100 MHz, $CDCl_3$) of compound **3Bc**:

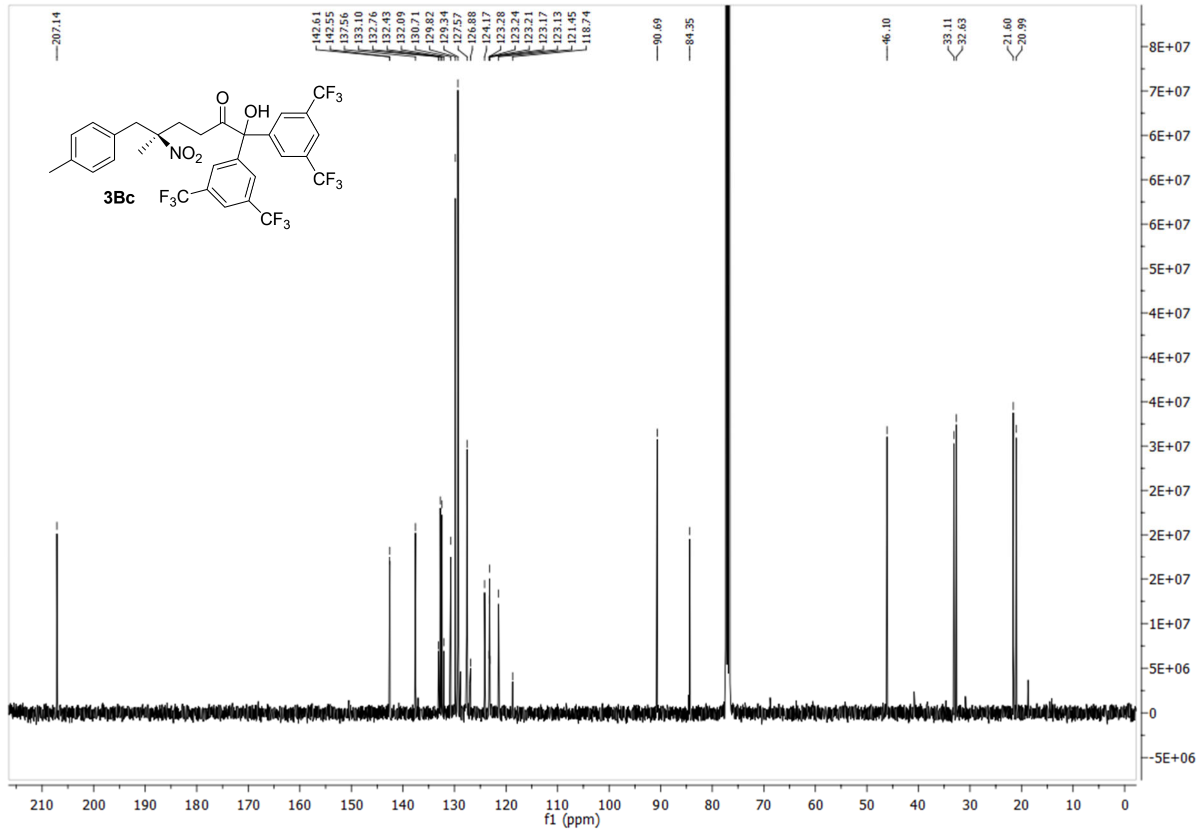

^{1}H NMR (400 MHz, $CDCl_3$) of compound **3Cc**:

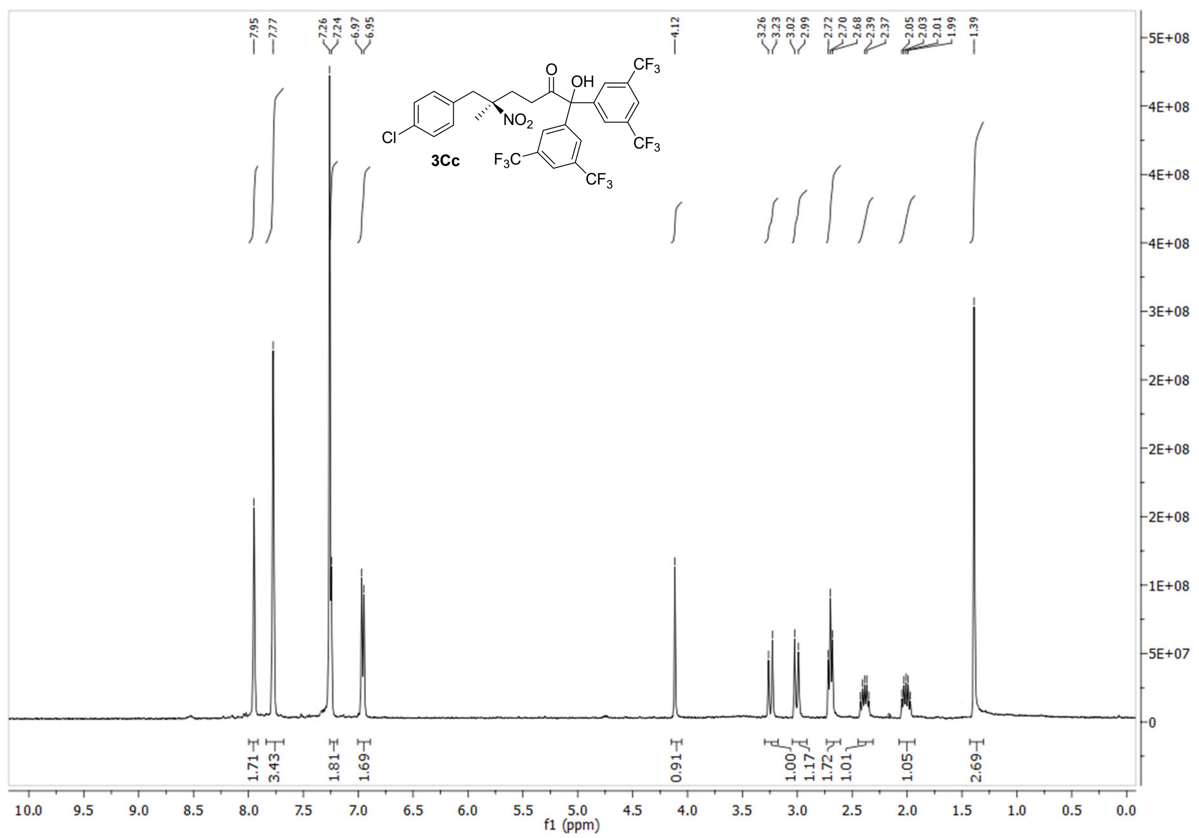

^{13}C NMR (100 MHz, $CDCl_3$) of compound **3Cc**:

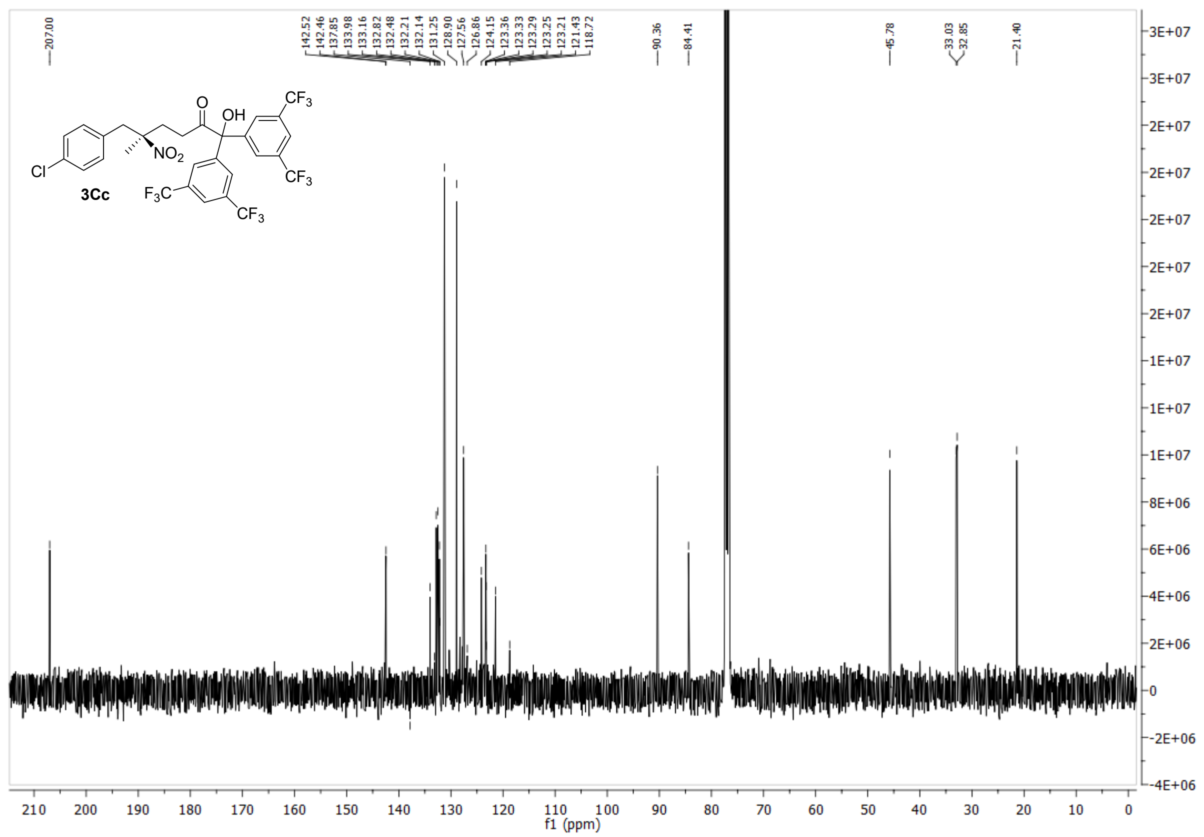

^{1}H NMR (400 MHz, $CDCl_3$) of compound **3Dc**:

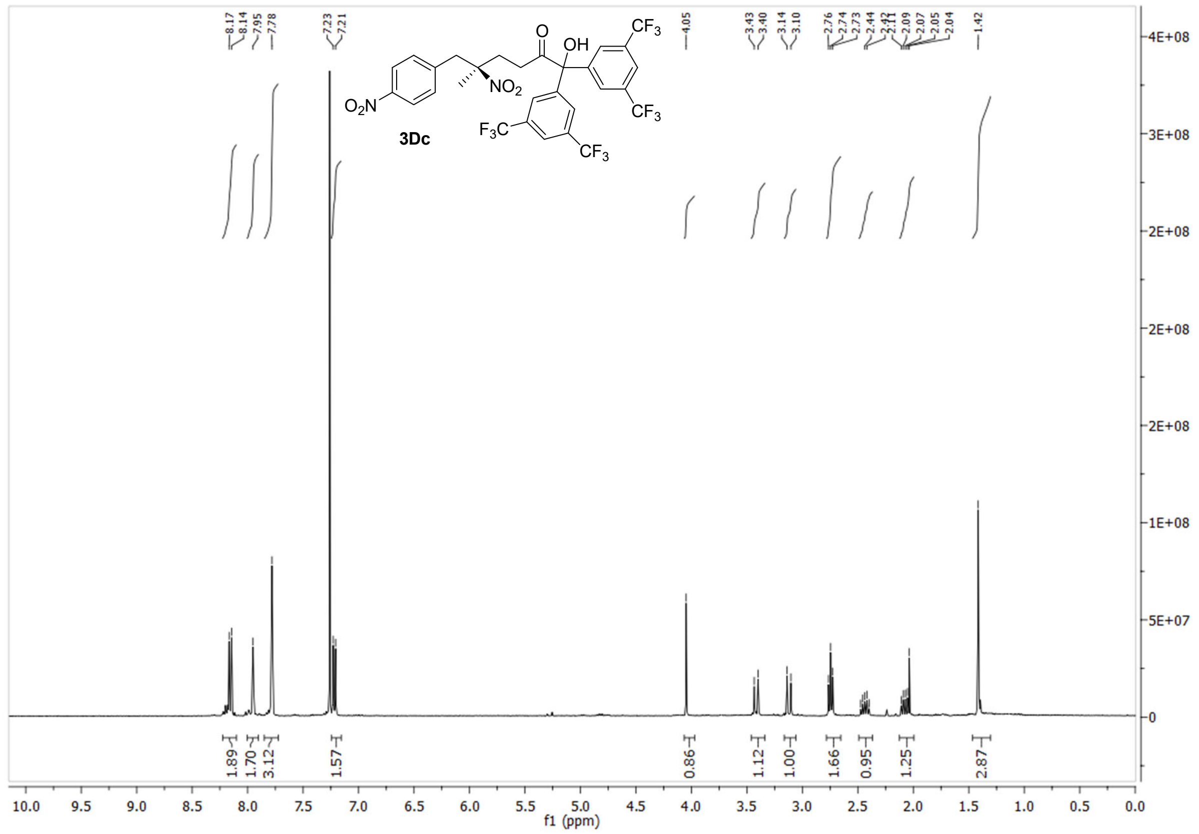

^{13}C NMR (100 MHz, $CDCl_3$) of compound **3Dc**:

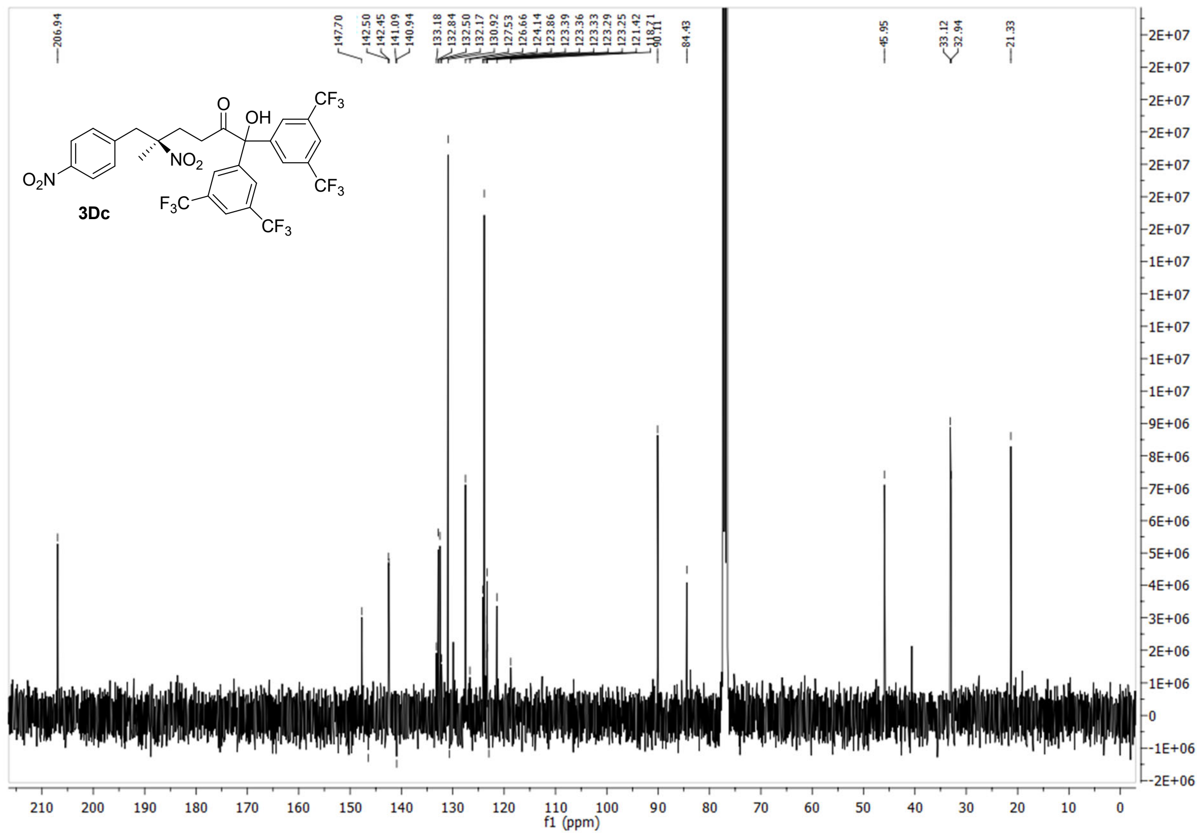

^{1}H NMR (400 MHz, $CDCl_3$) of compound **3Ea**:

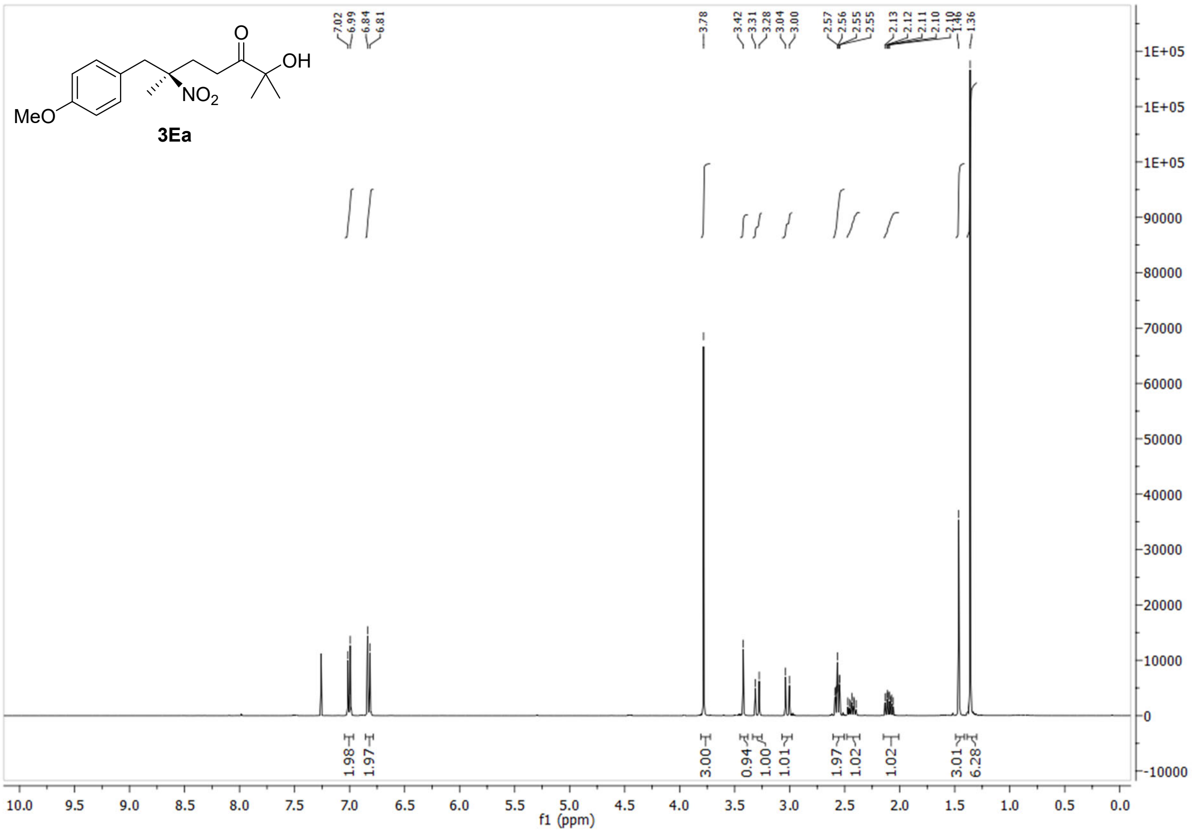

^{13}C NMR (100 MHz, $CDCl_3$) of compound **3Ea**:

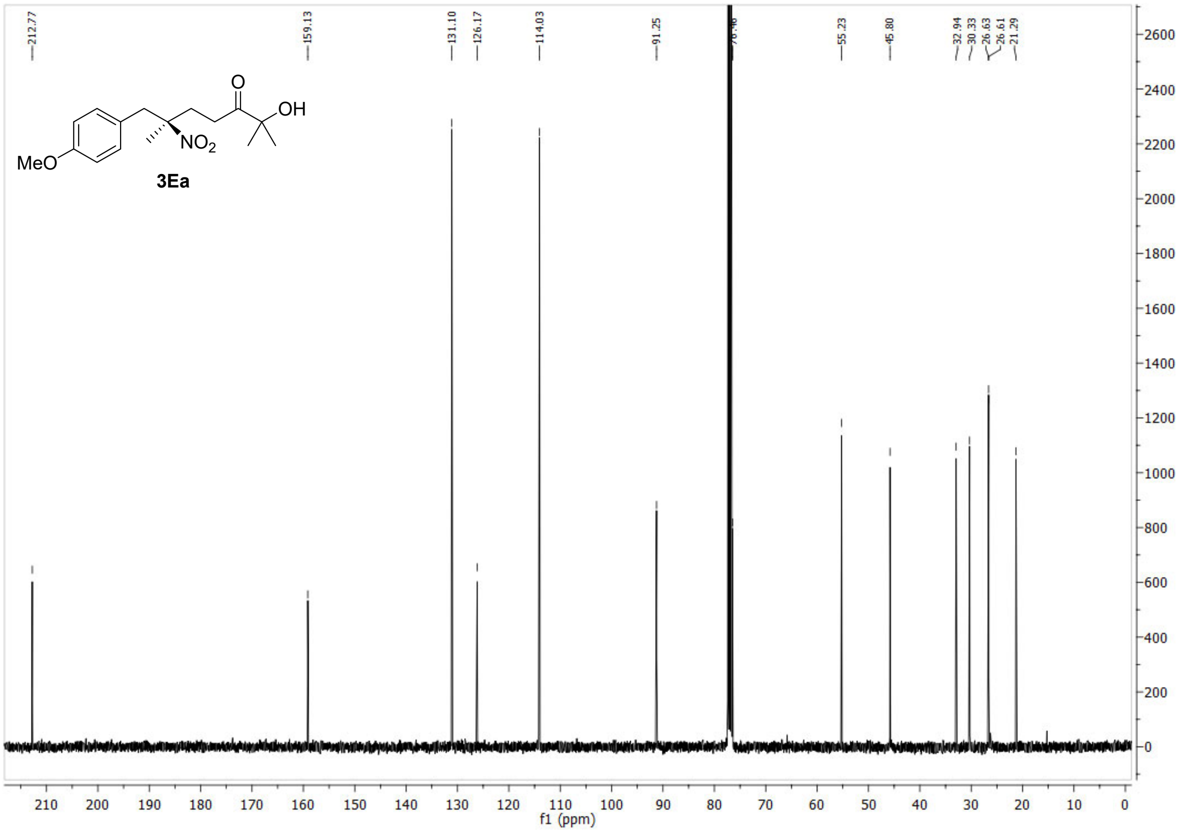

^{1}H NMR (400 MHz, $CDCl_3$) of compound **3Ec**:

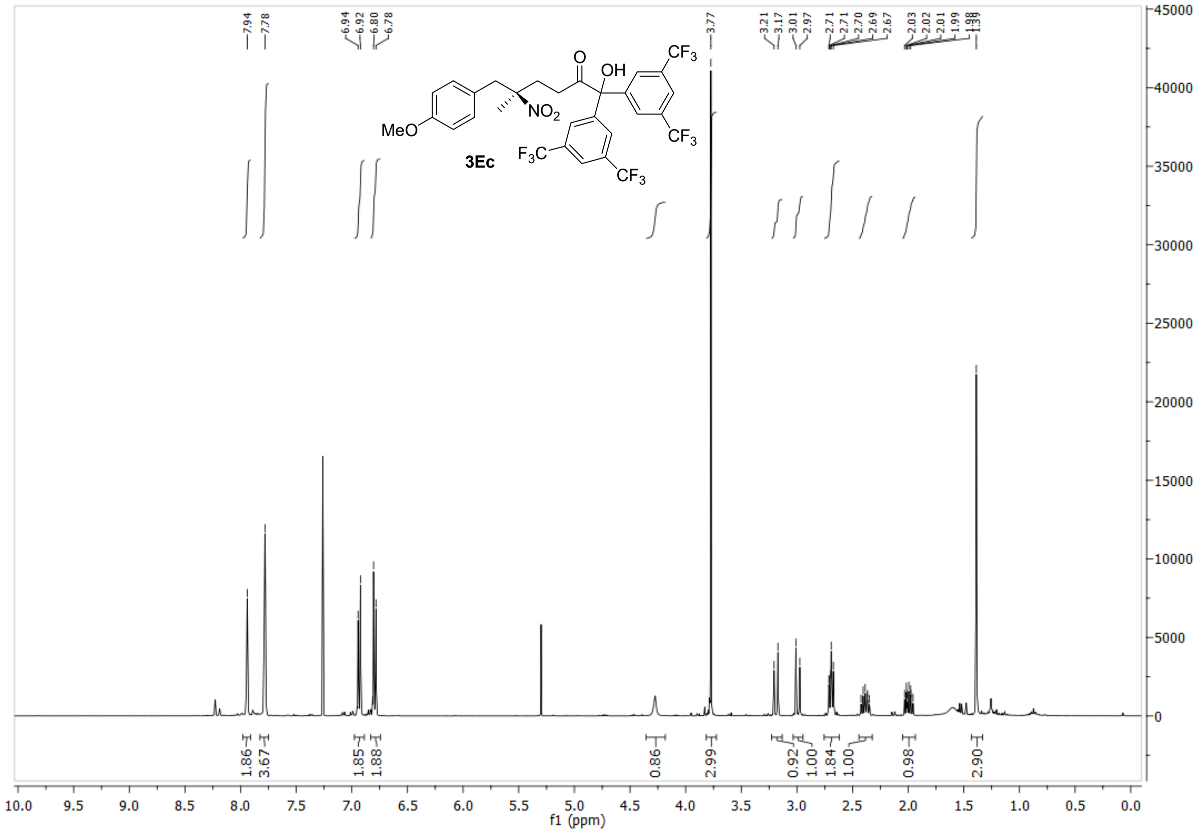

^{13}C NMR (100 MHz, $CDCl_3$) of compound **3Ec**:

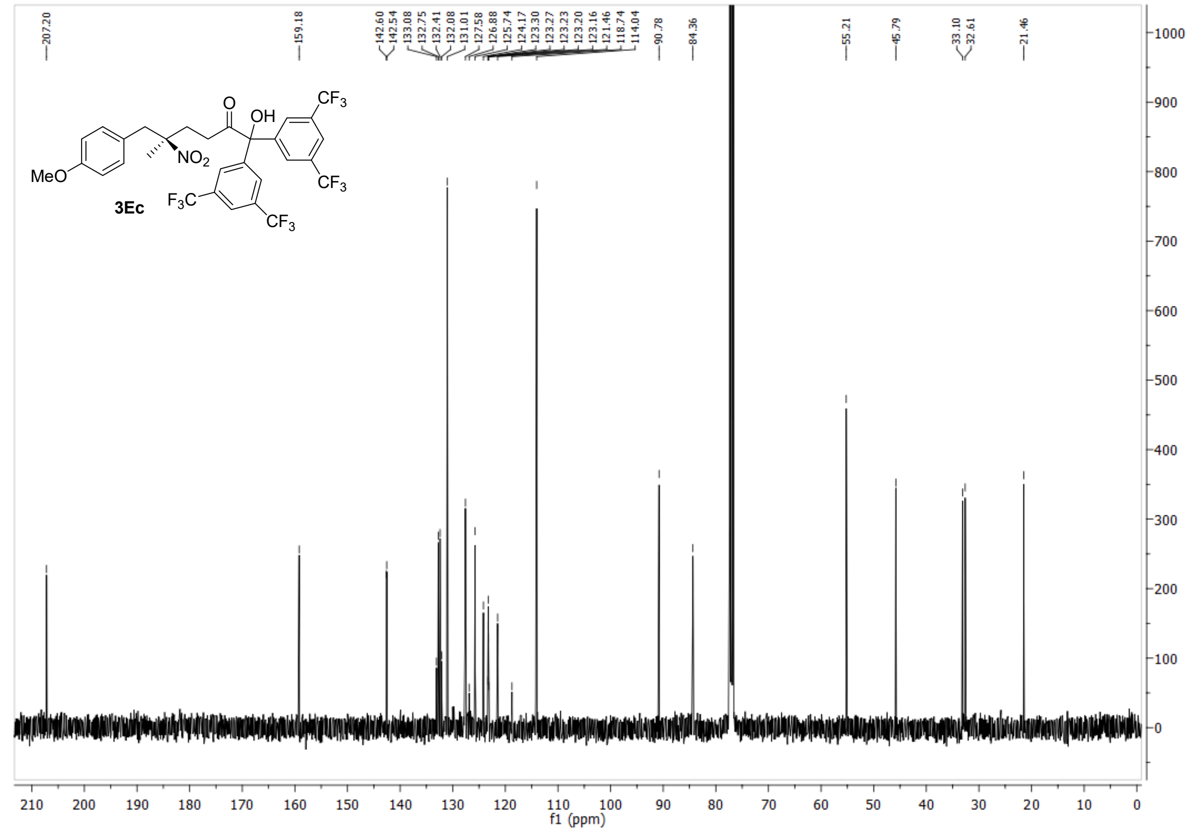

^{1}H NMR (400 MHz, $CDCl_3$) of compound **3Fa**:

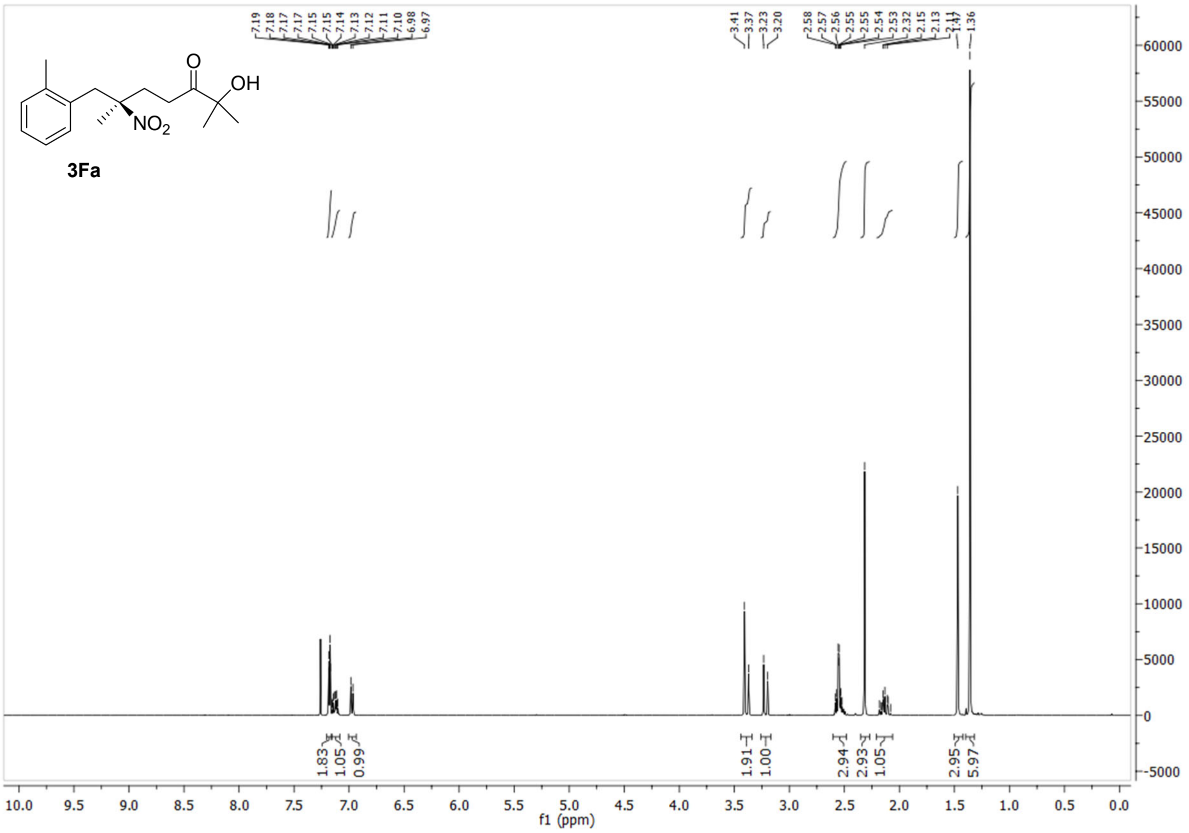

^{13}C NMR (100 MHz, $CDCl_3$) of compound **3Fa**:

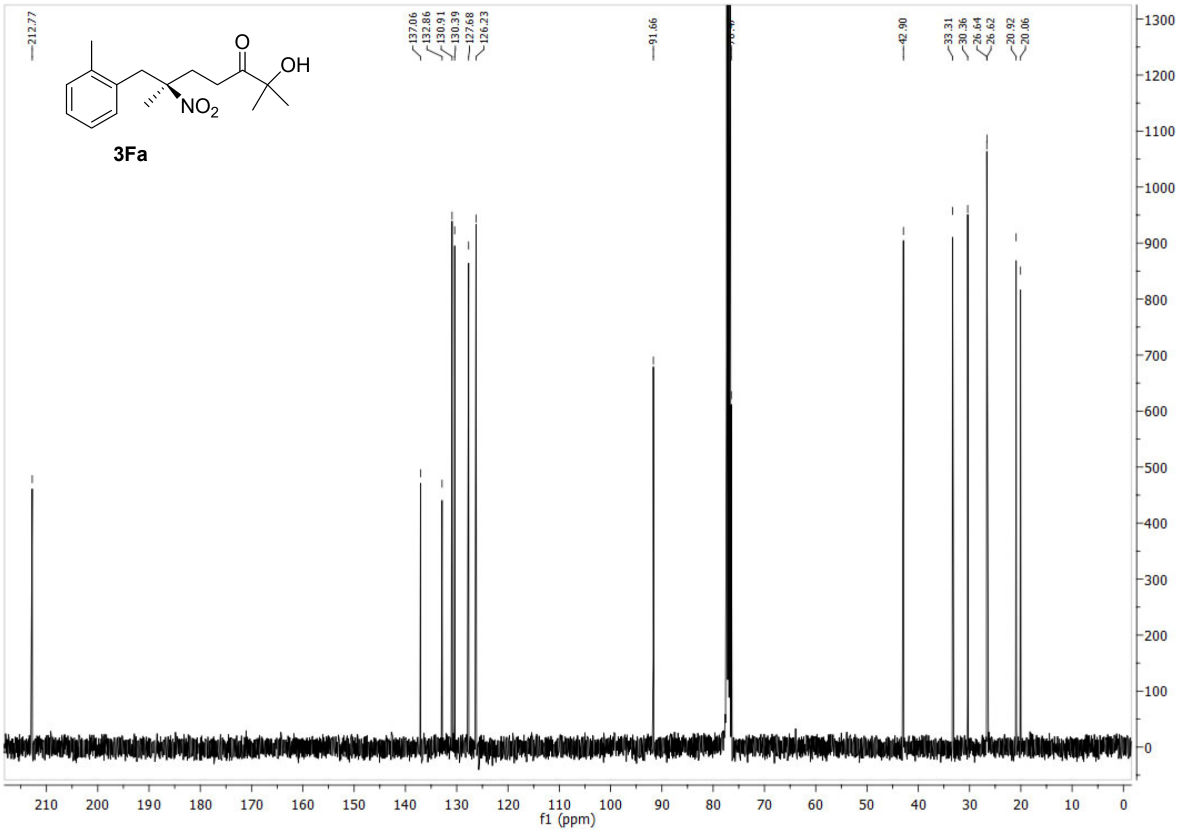

^{1}H NMR (400 MHz, $CDCl_3$) of compound **3Fc**:

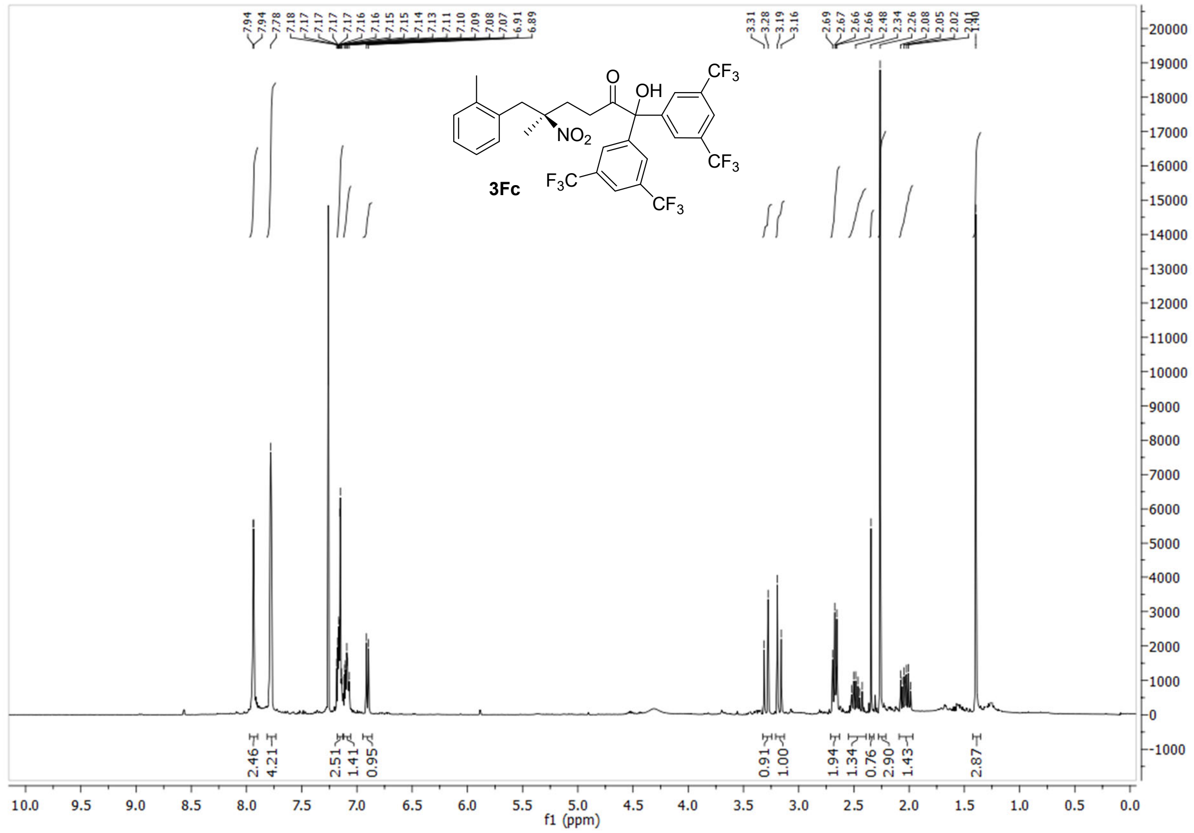

^{13}C NMR (100 MHz, $CDCl_3$) of compound **3Fc**:

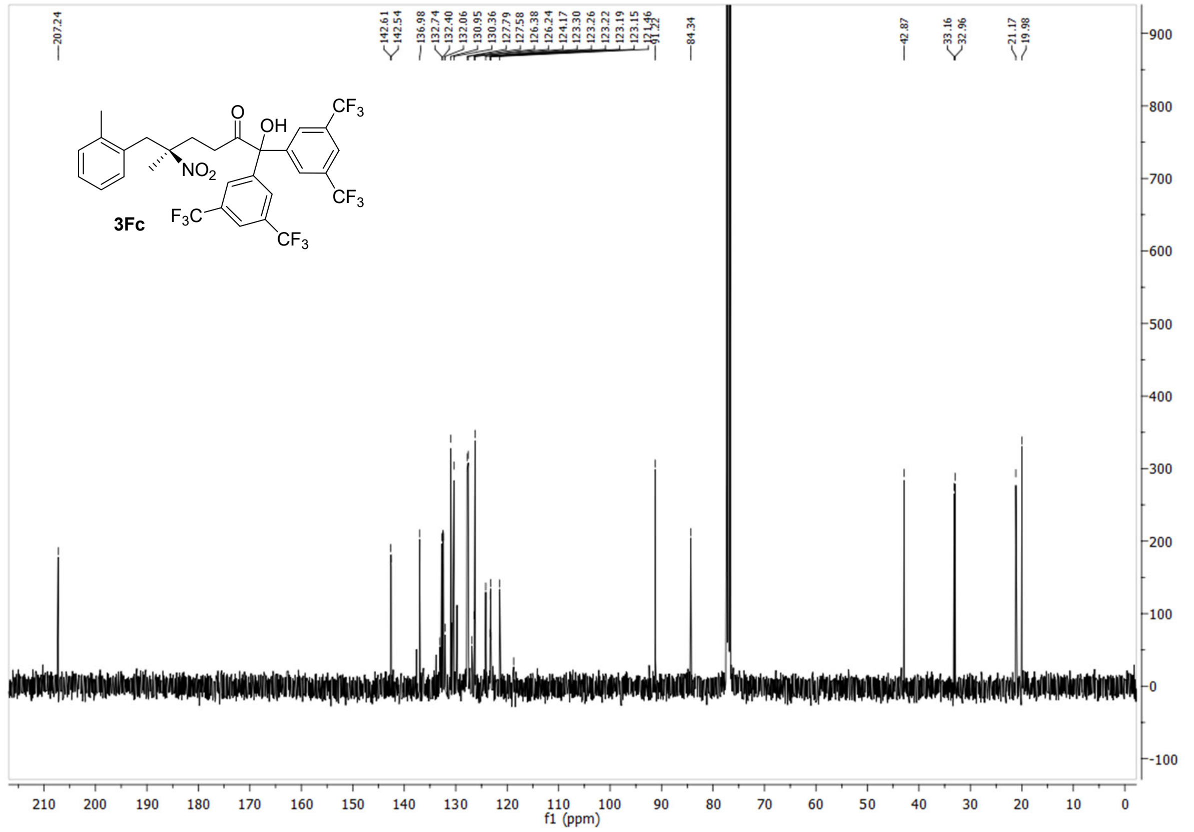

^{1}H NMR (400 MHz, $CDCl_3$) of compound **3Gc**:

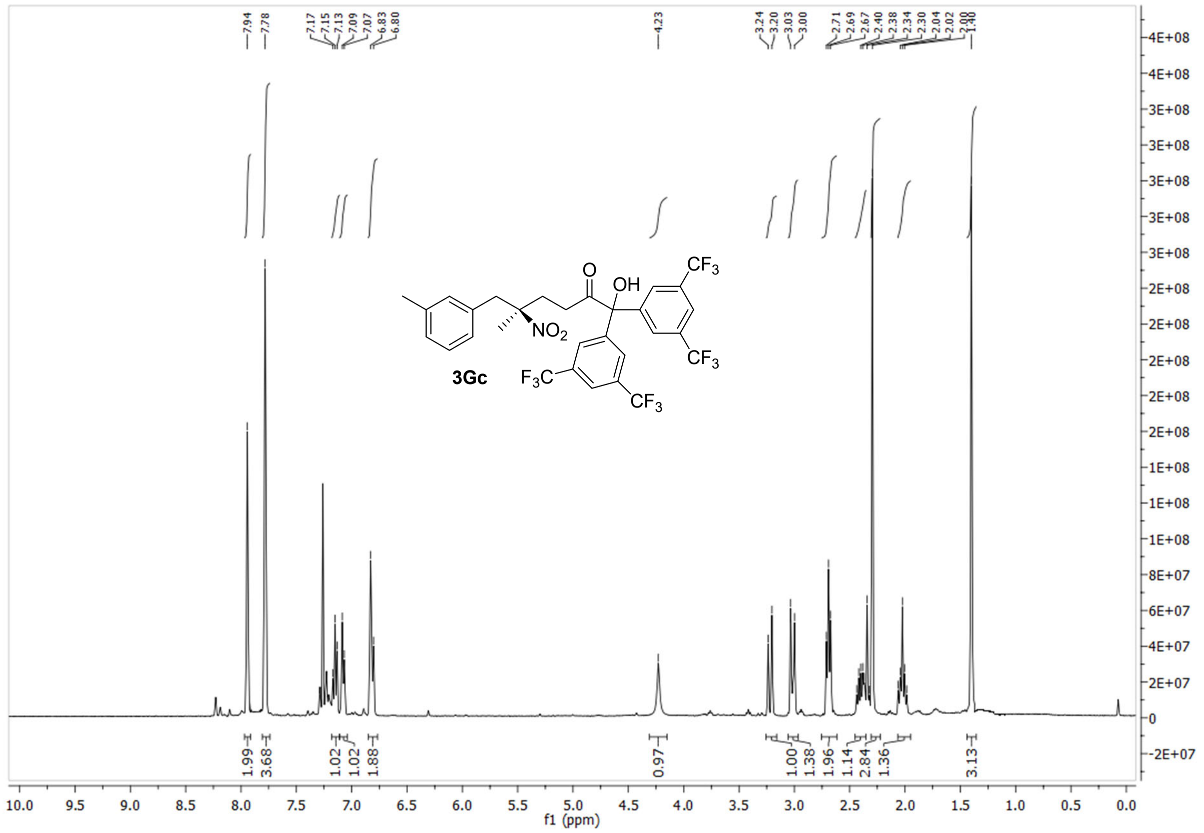

^{13}C NMR (100 MHz, $CDCl_3$) of compound **3Gc**:

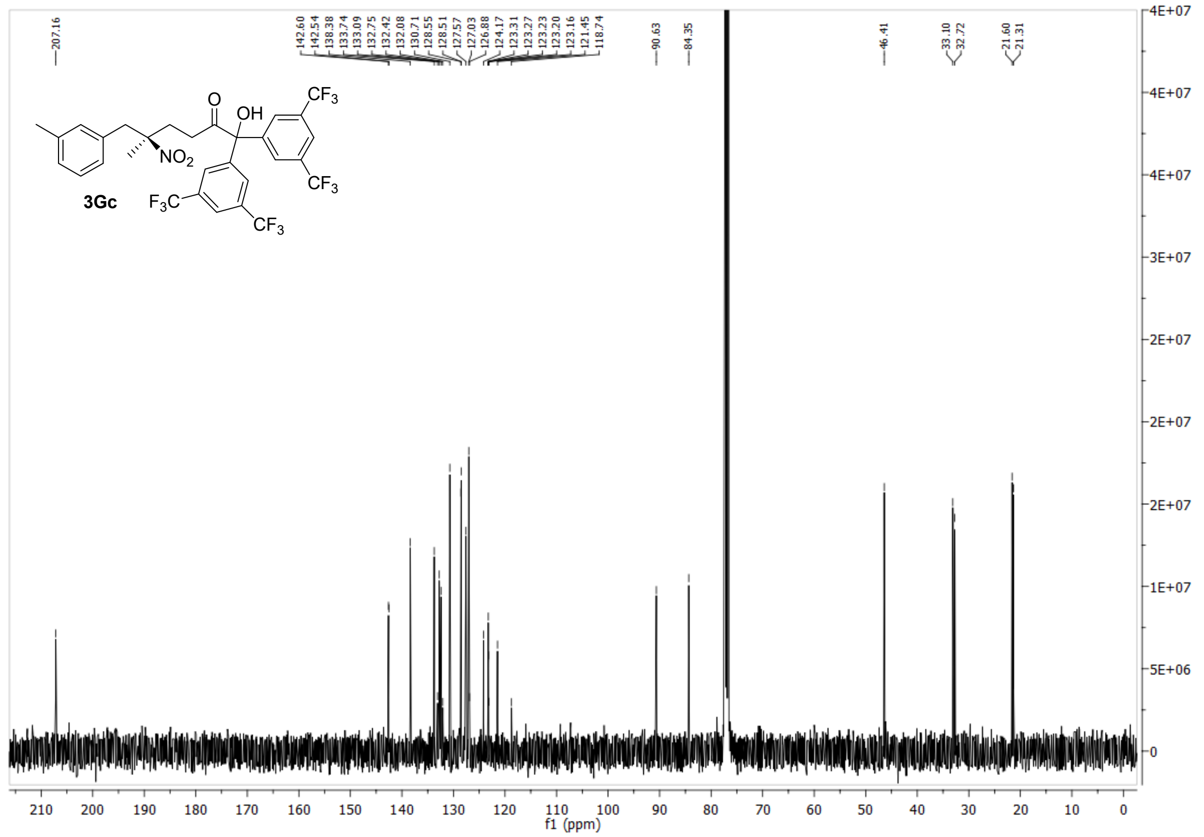

^{1}H NMR (400 MHz, $CDCl_3$) of compound **3Hc**:

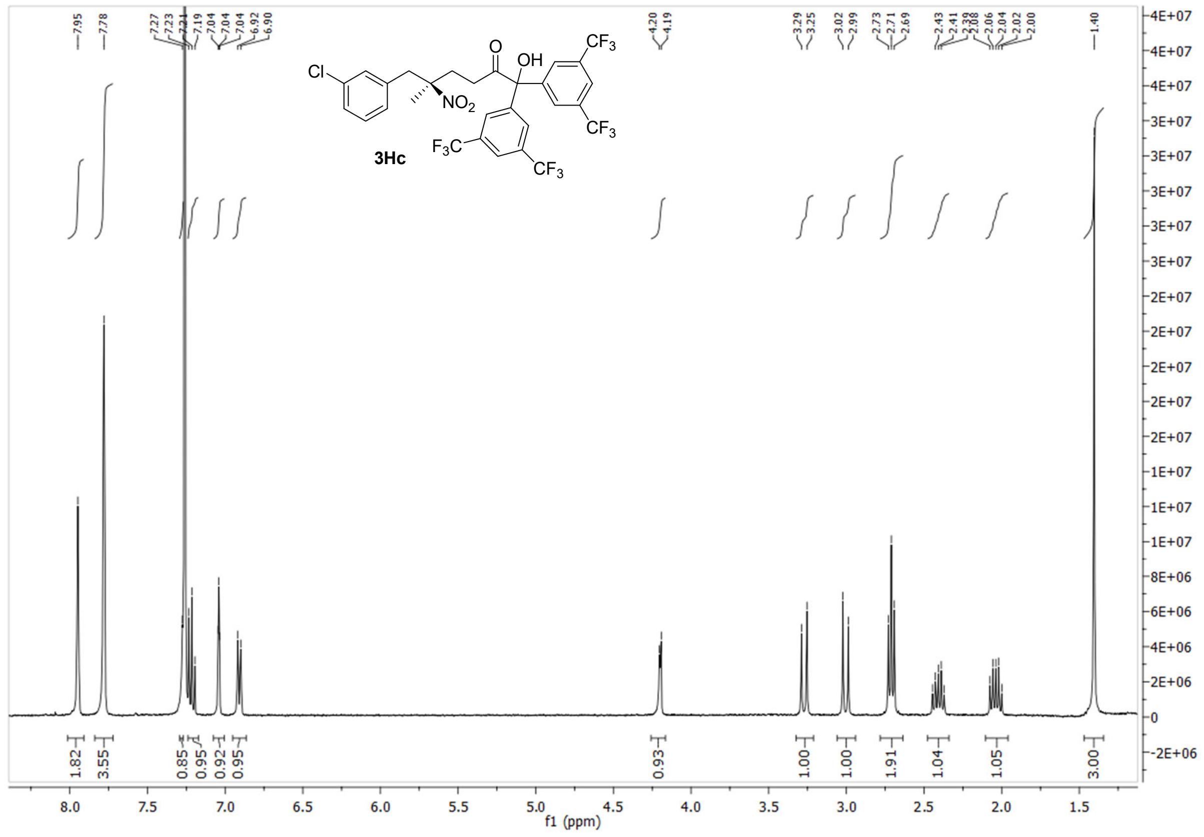

^{13}C NMR (100 MHz, $CDCl_3$) of compound **3Hc**:

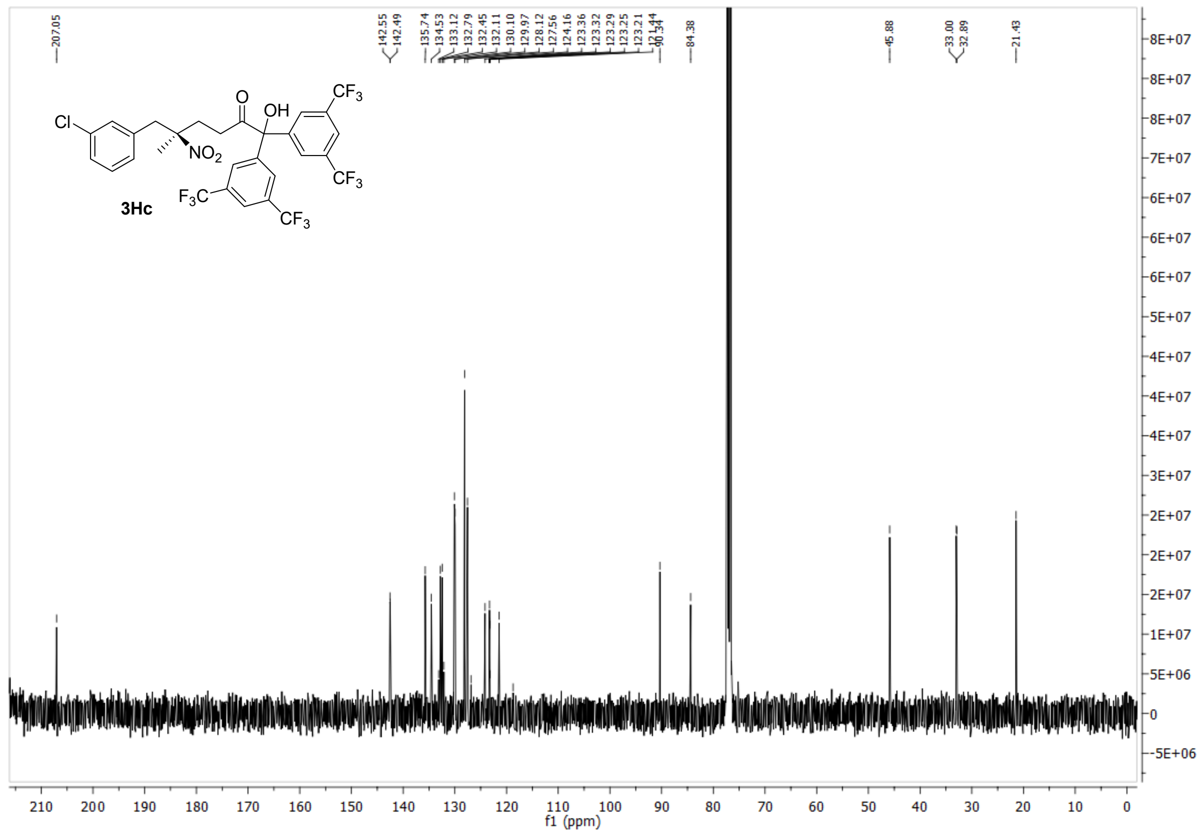

^1H NMR (400 MHz, $CDCl_3$) of compound **3lc**:

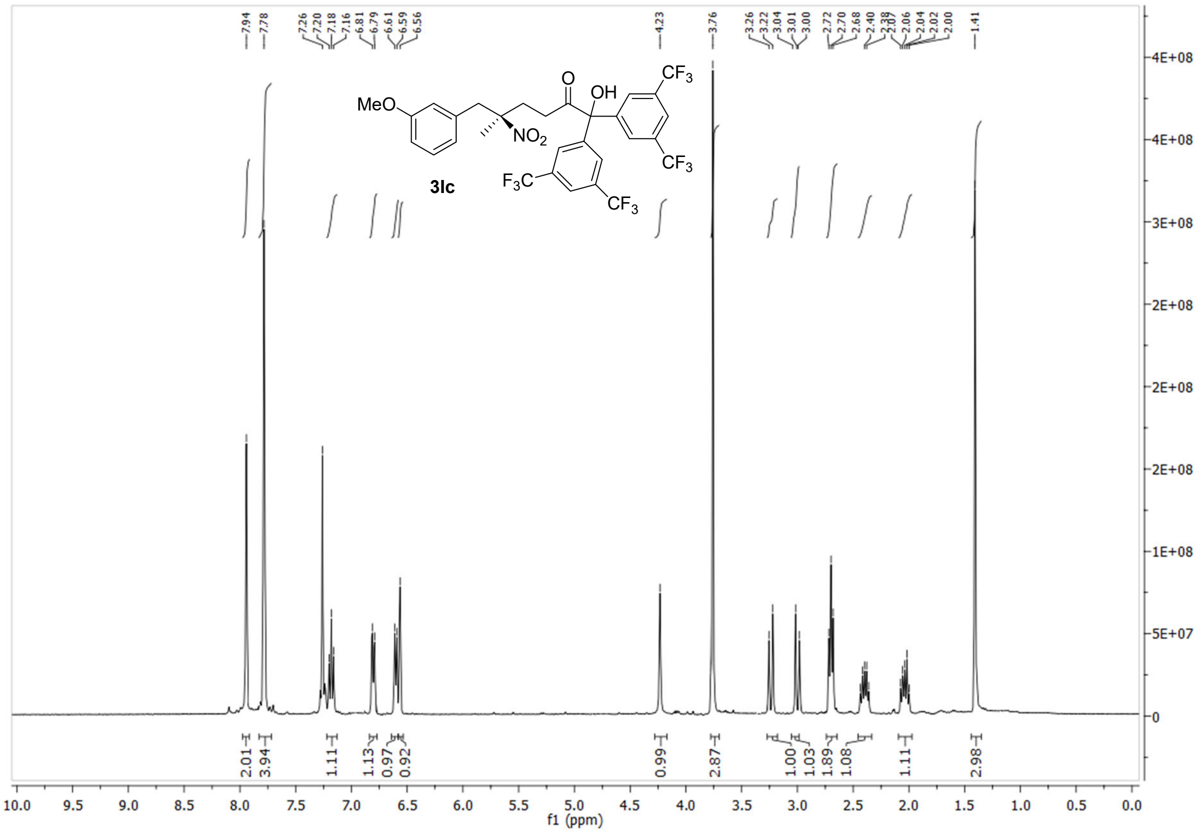

^{13}C NMR (100 MHz, $CDCl_3$) of compound **3lc**:

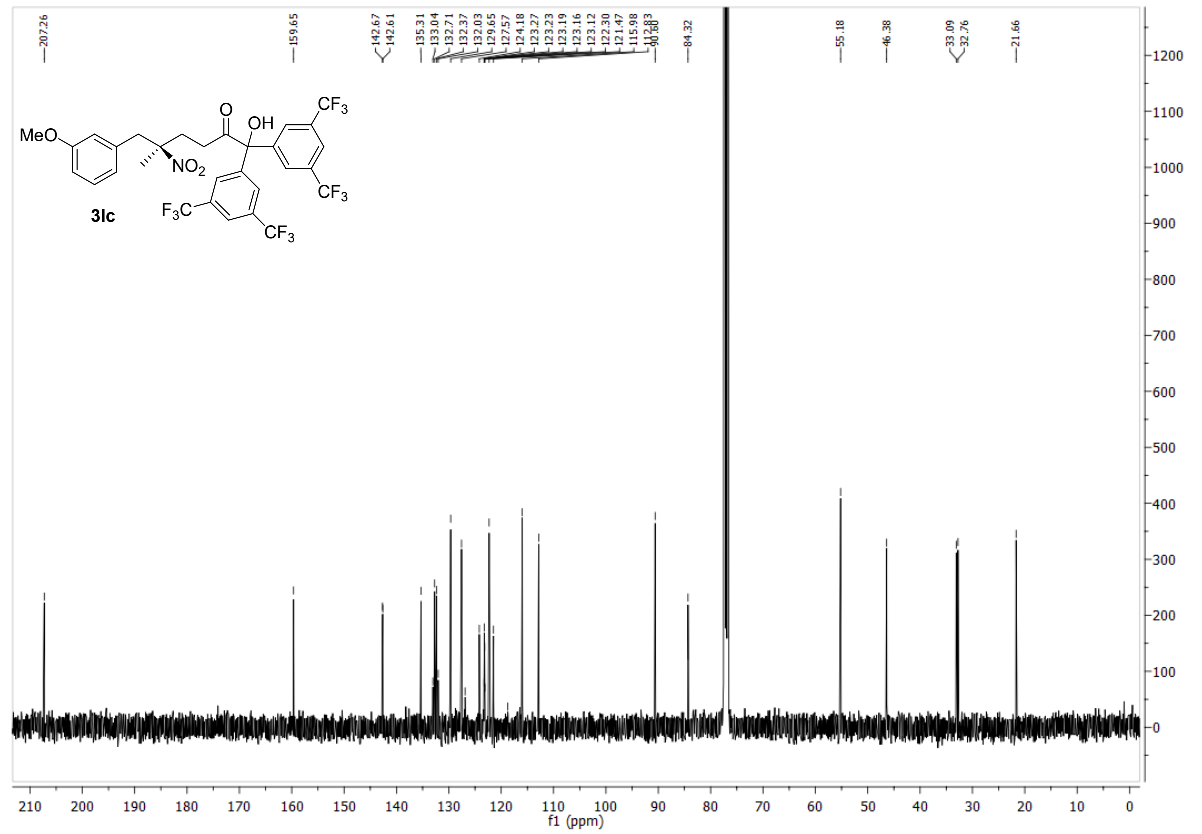

^{1}H NMR (400 MHz, $CDCl_3$) of compound **3Ja**:

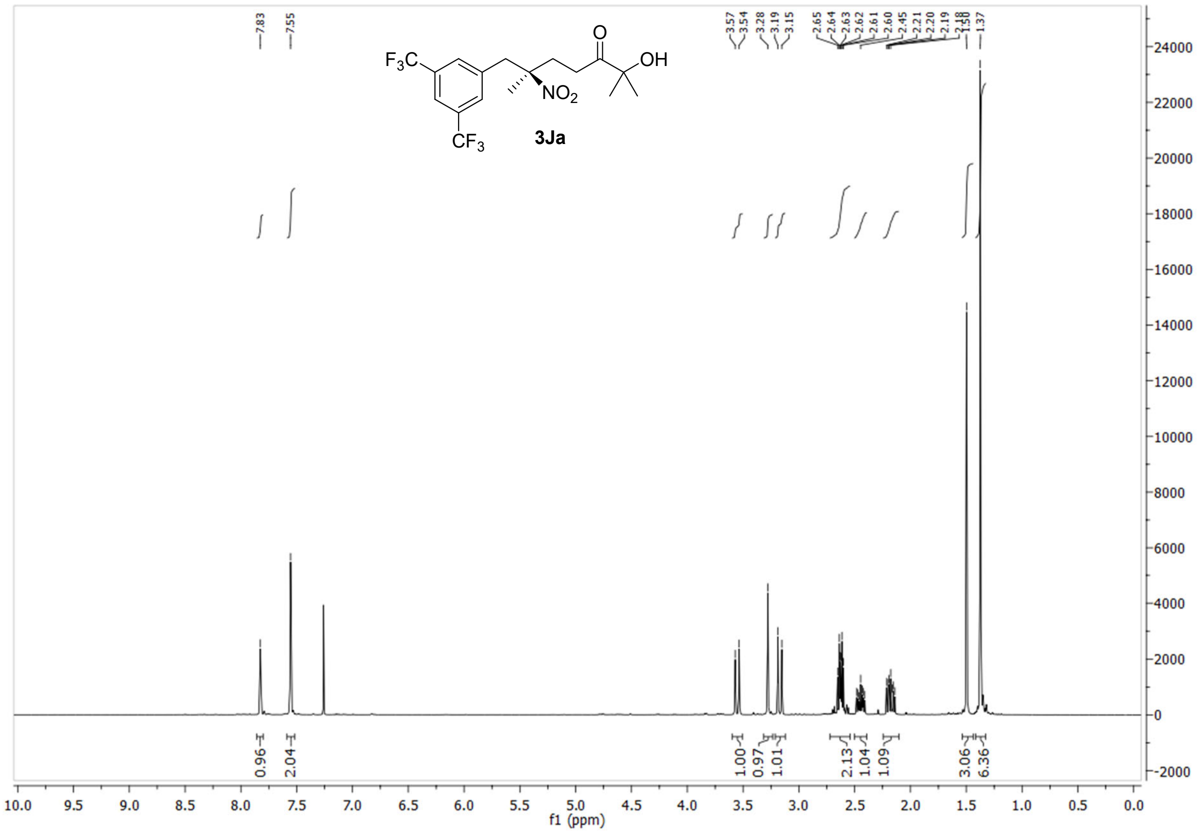

^{13}C NMR (100 MHz, $CDCl_3$) of compound **3Ja**:

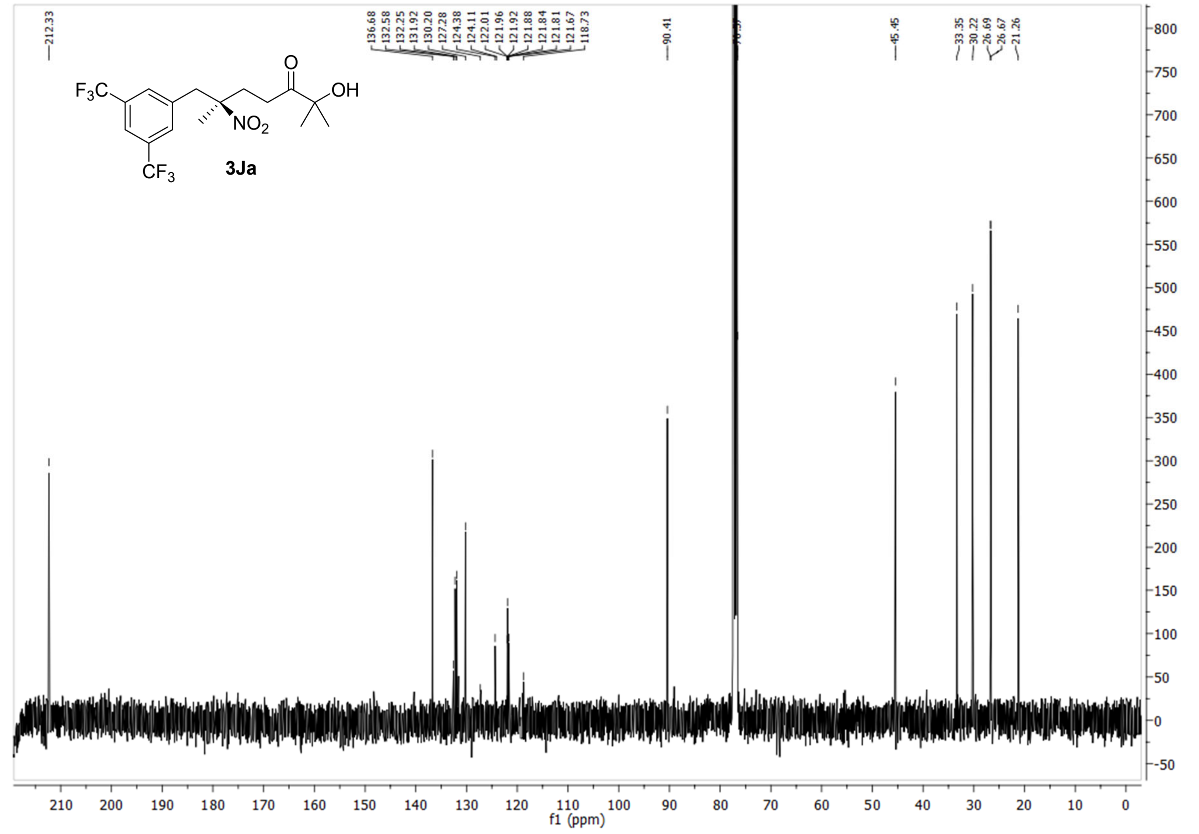

^{1}H NMR (400 MHz, $CDCl_3$) of compound **3Jc**:

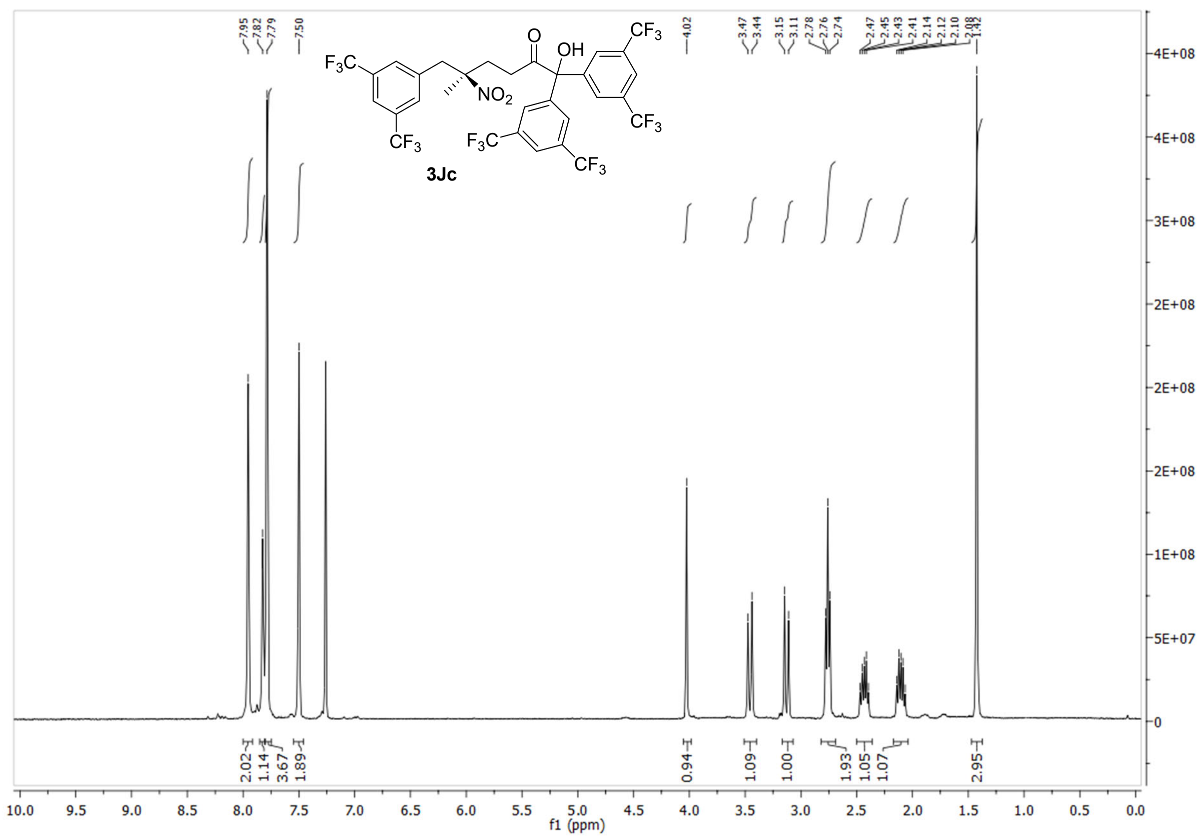

^{13}C NMR (100 MHz, $CDCl_3$) of compound **3Jc**:

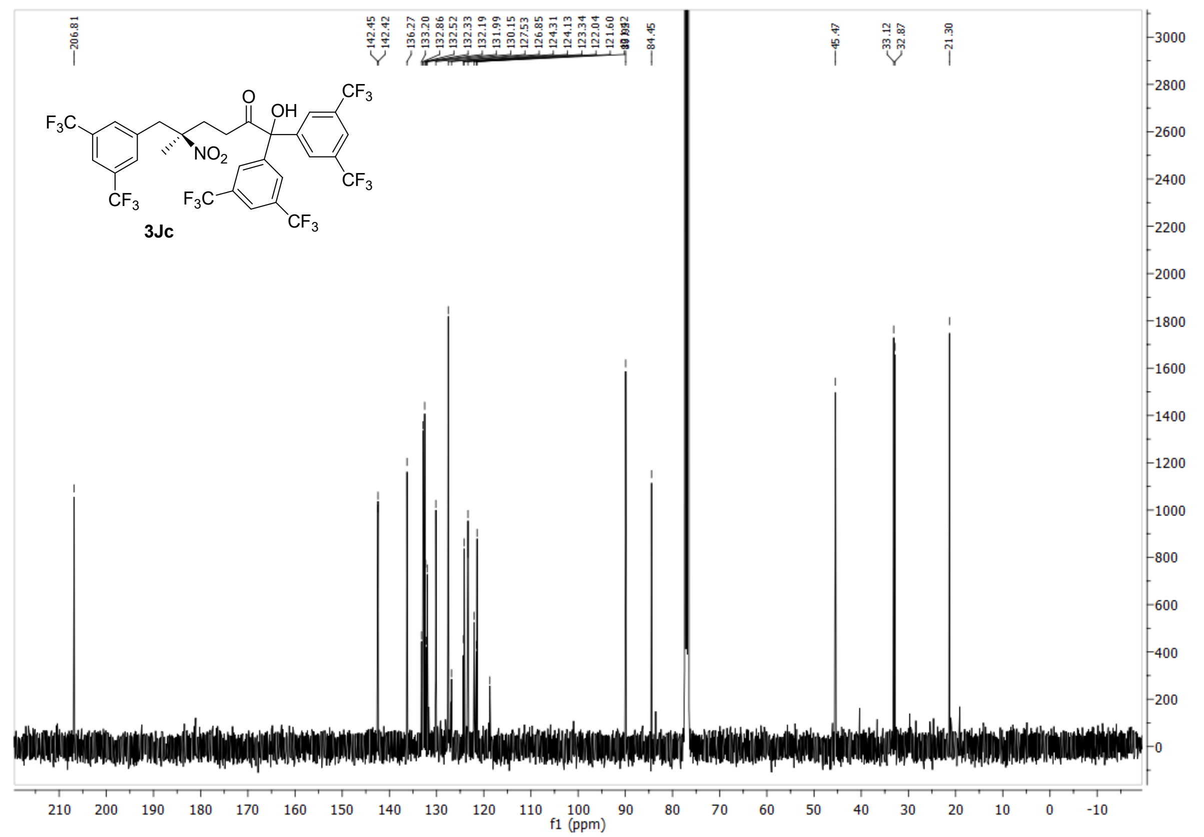

^{1}H NMR (400 MHz, $CDCl_3$) of compound **3Kc**:

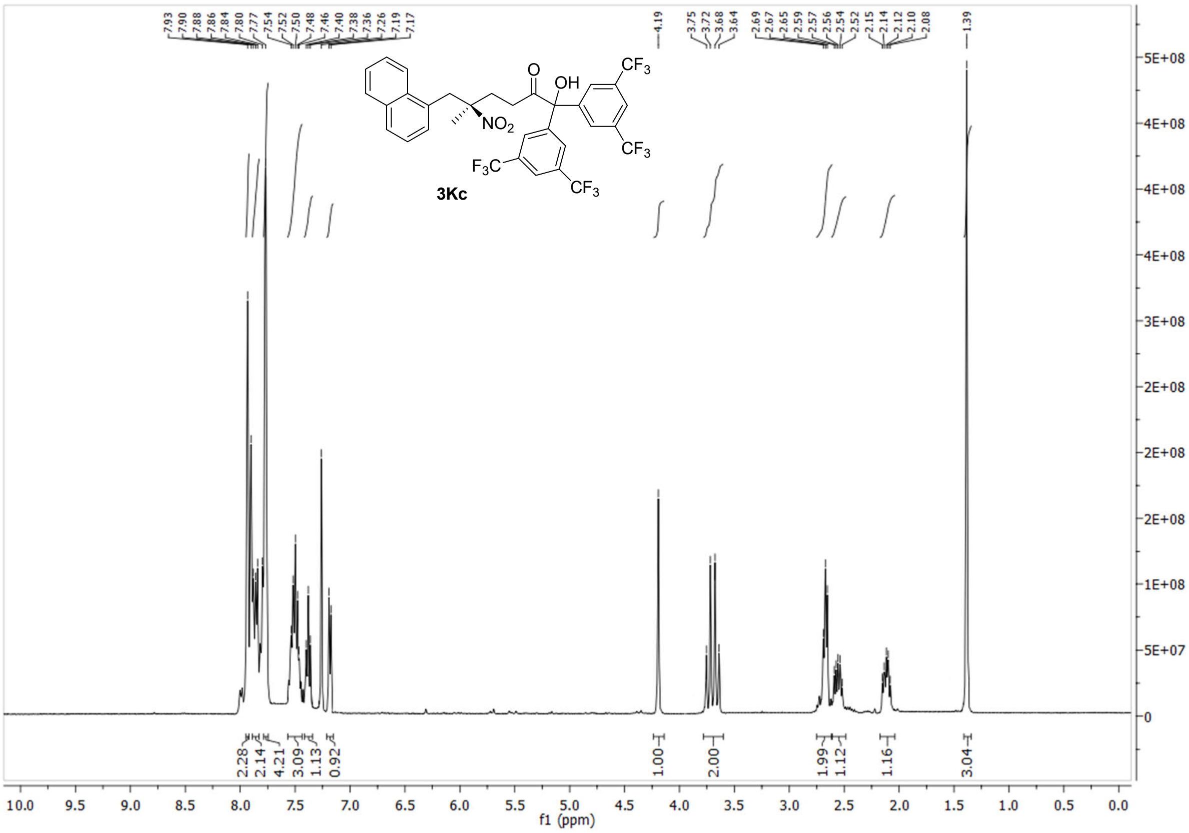

^{13}C NMR (100 MHz, $CDCl_3$) of compound **3Kc**:

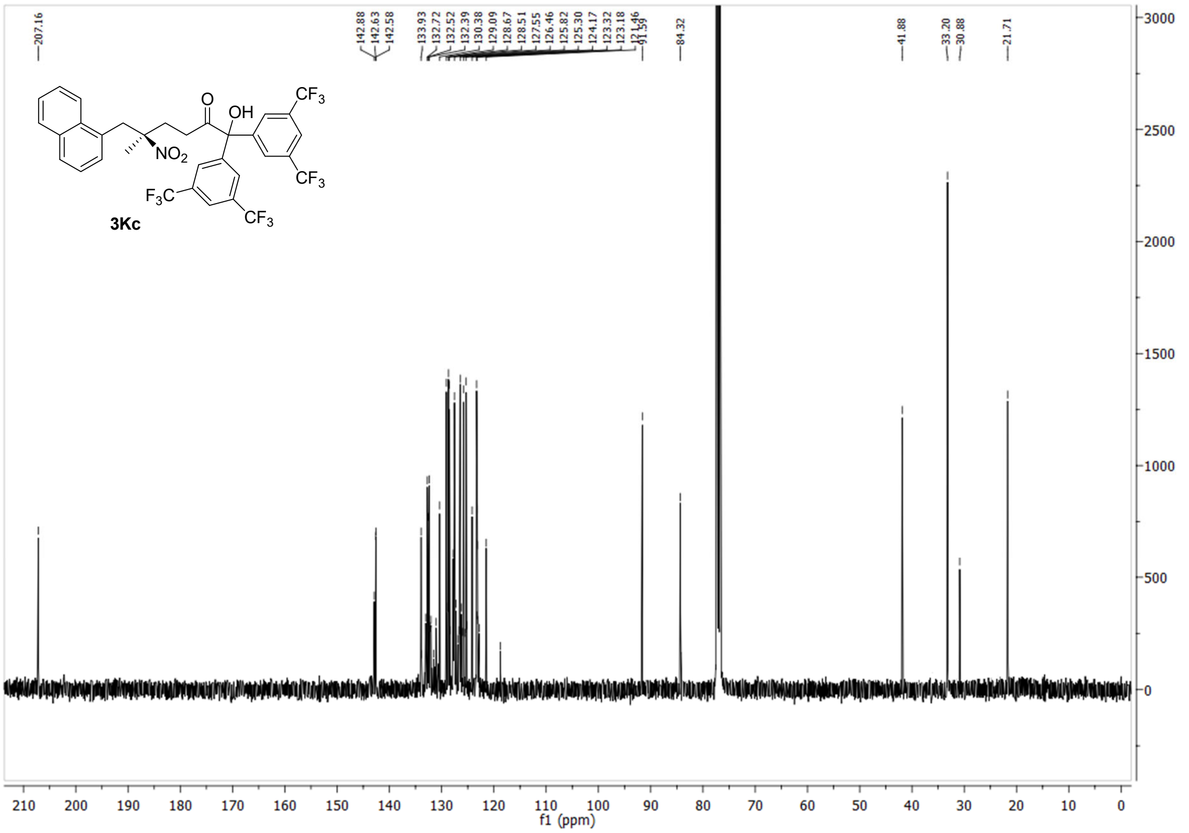

^{1}H NMR (400 MHz, $CDCl_3$) of compound **3Lc**:

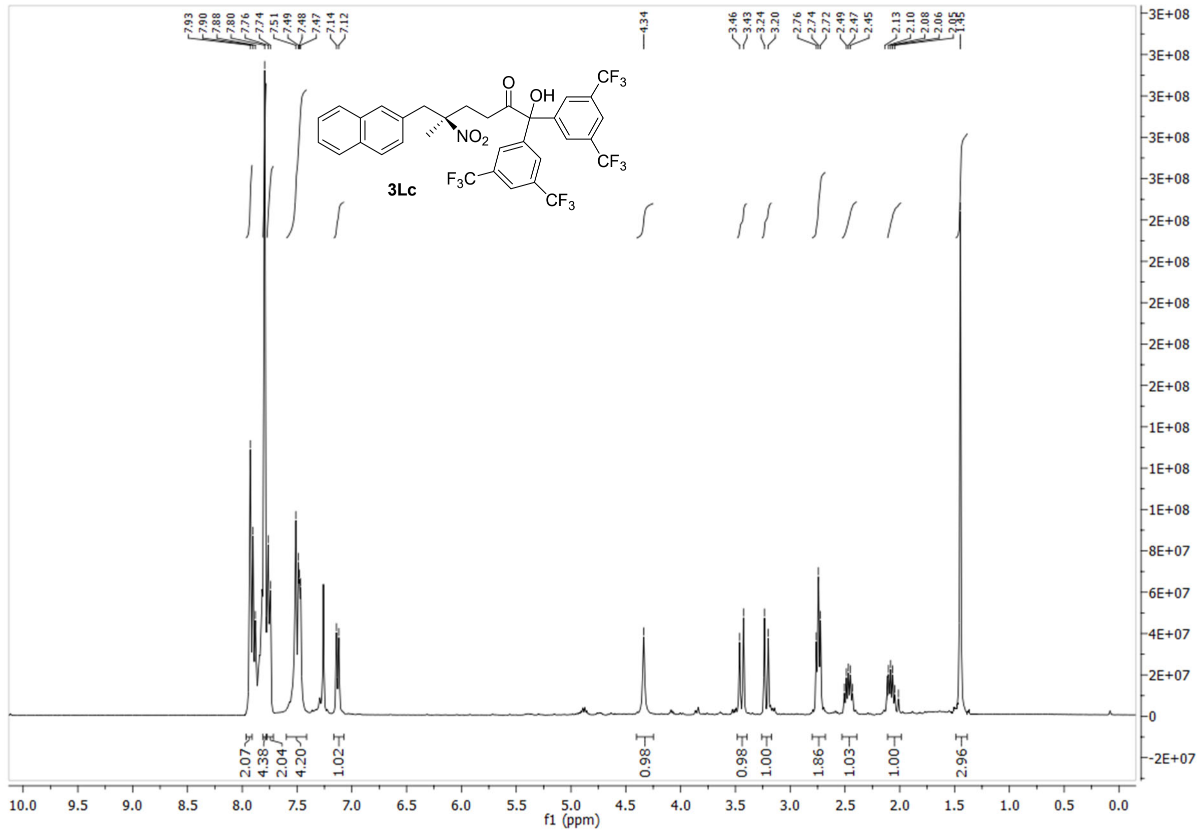

^{13}C NMR (100 MHz, $CDCl_3$) of compound **3Lc**:

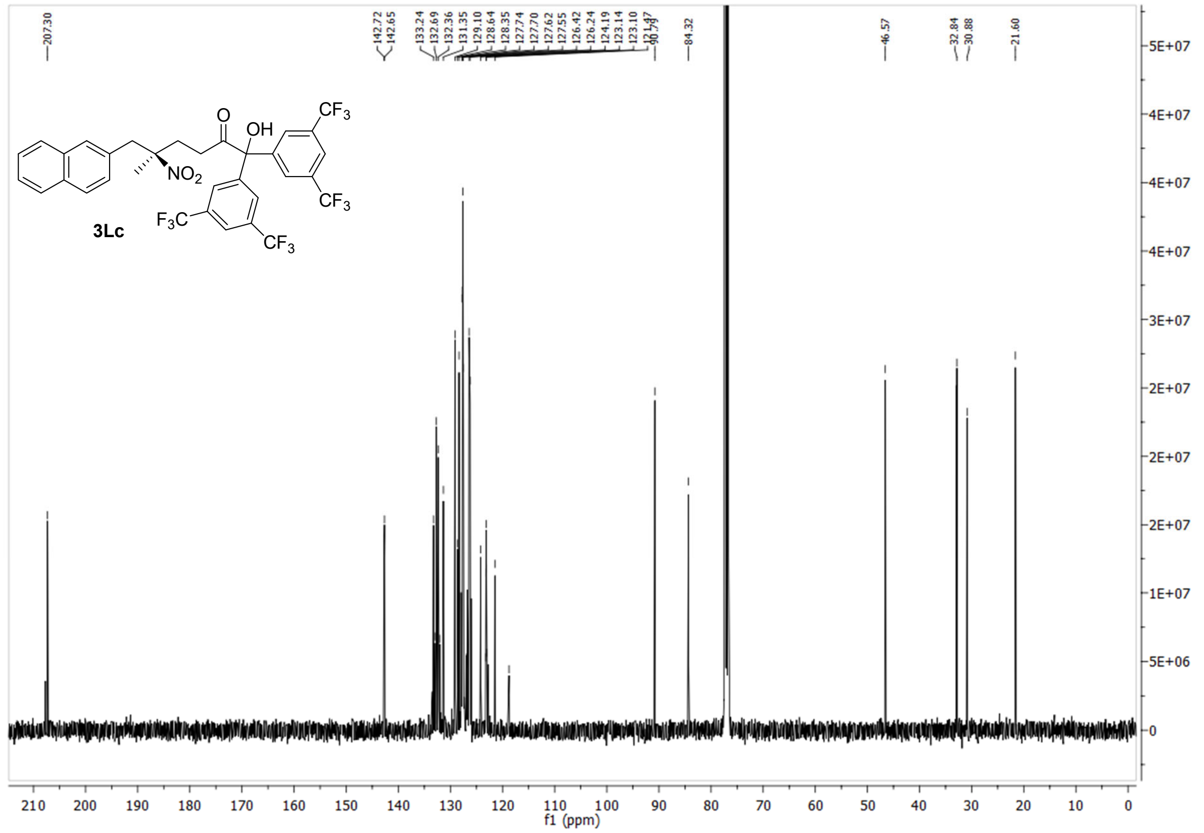

^{1}H NMR (400 MHz, $CDCl_3$) of compound **3Mc**:

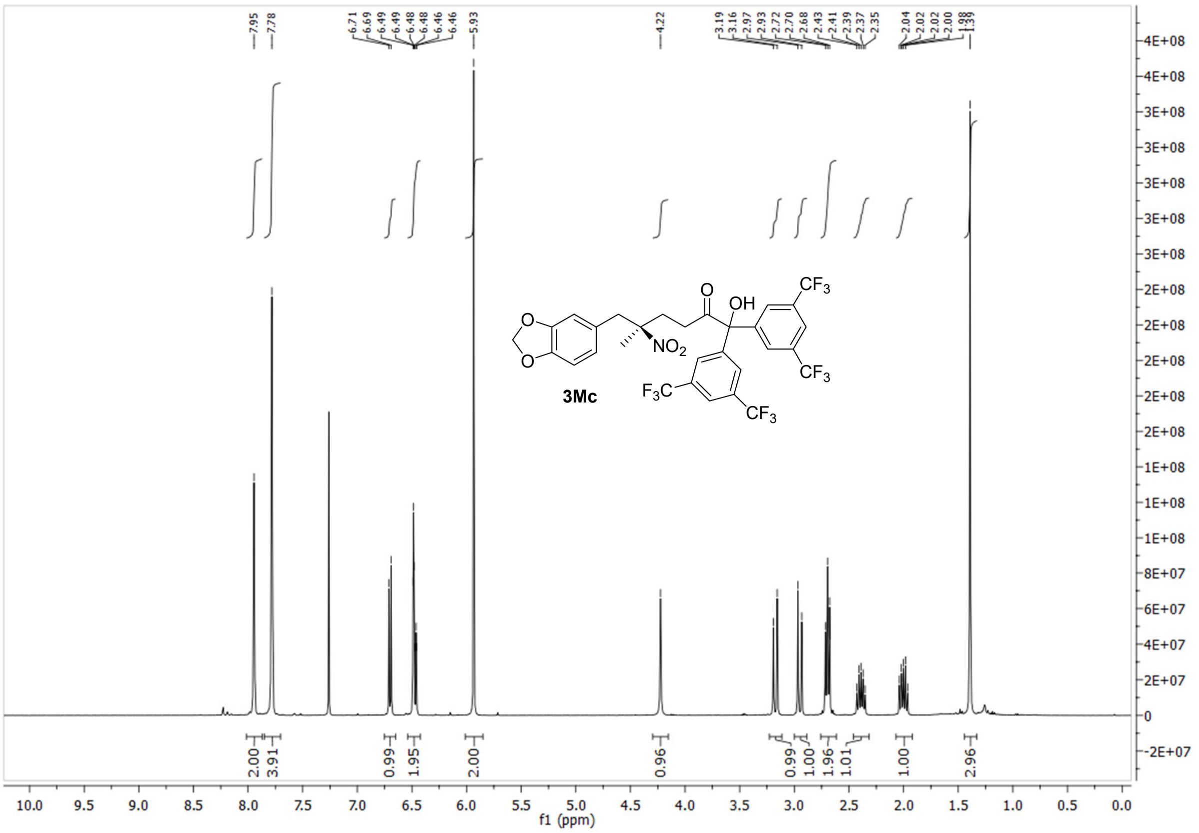

^{13}C NMR (100 MHz, $CDCl_3$) of compound **3Mc**:

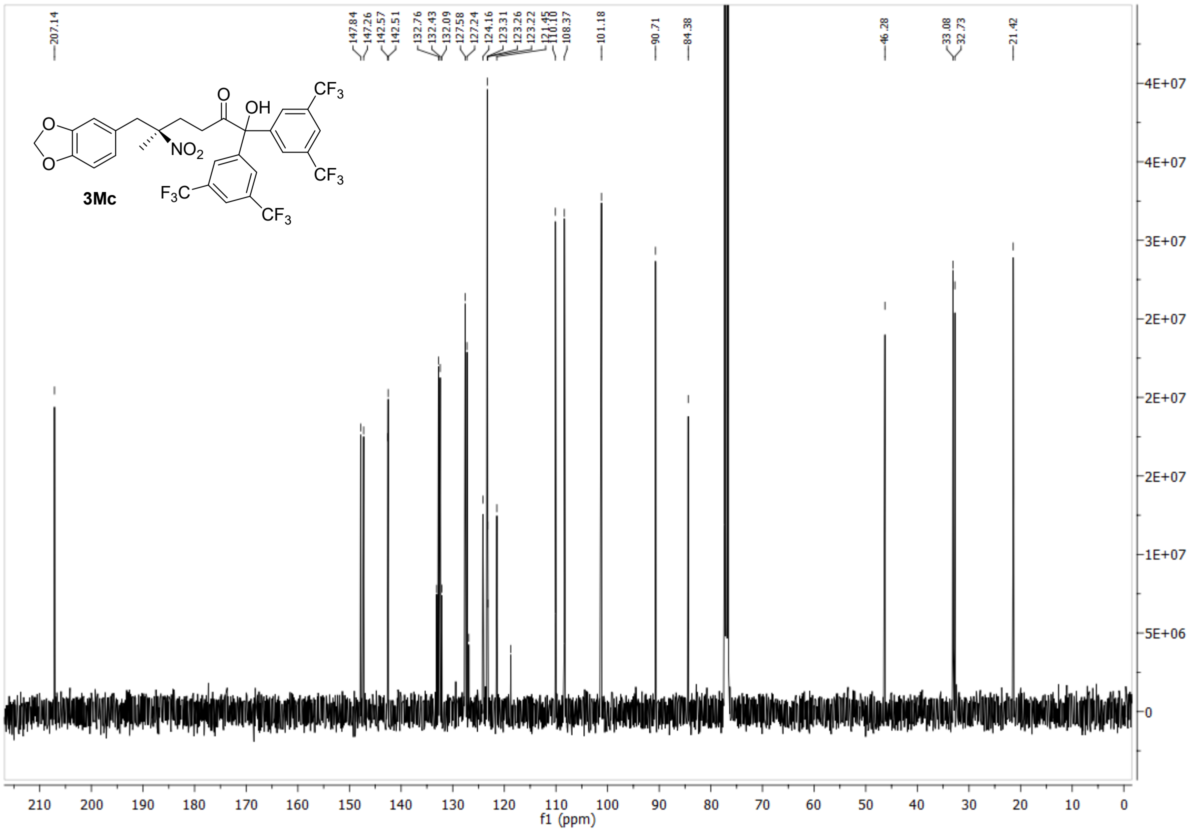

^{1}H NMR (400 MHz, $CDCl_3$) of compound **3Na**:

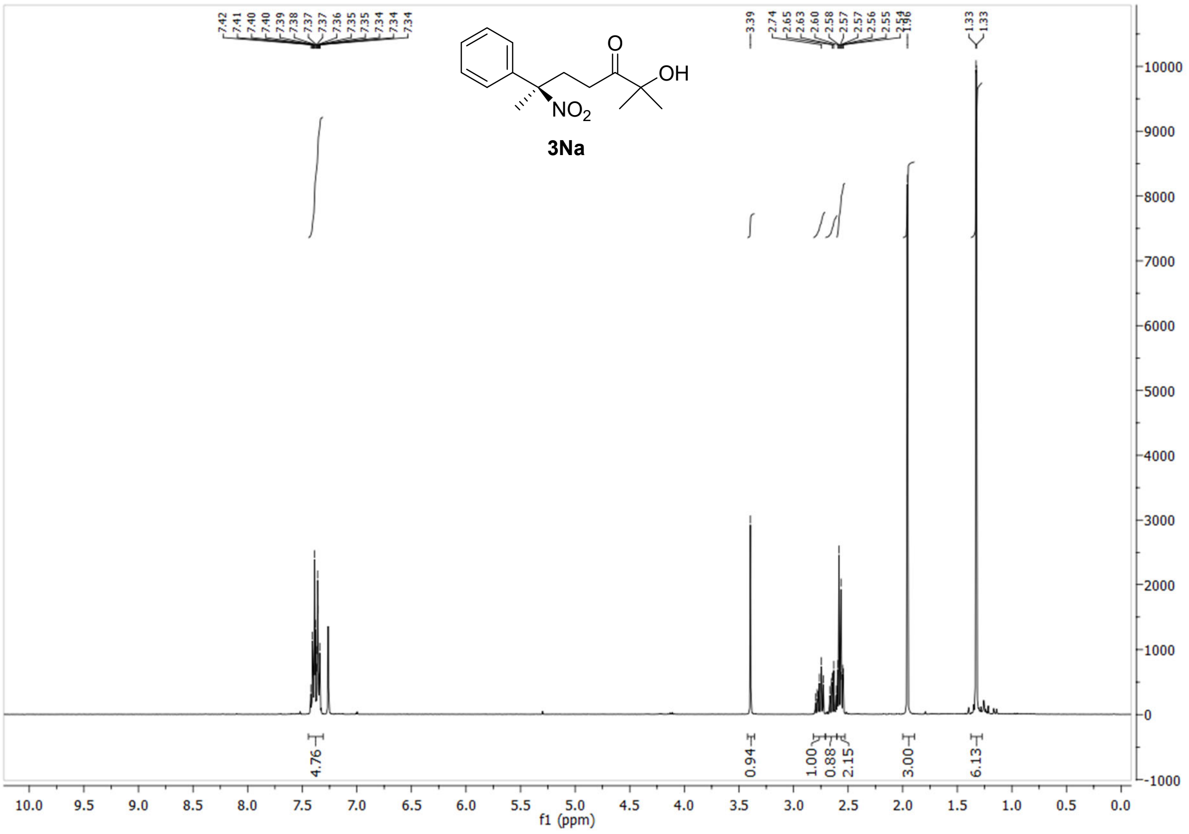

^{13}C NMR (100 MHz, $CDCl_3$) of compound **3Na**:

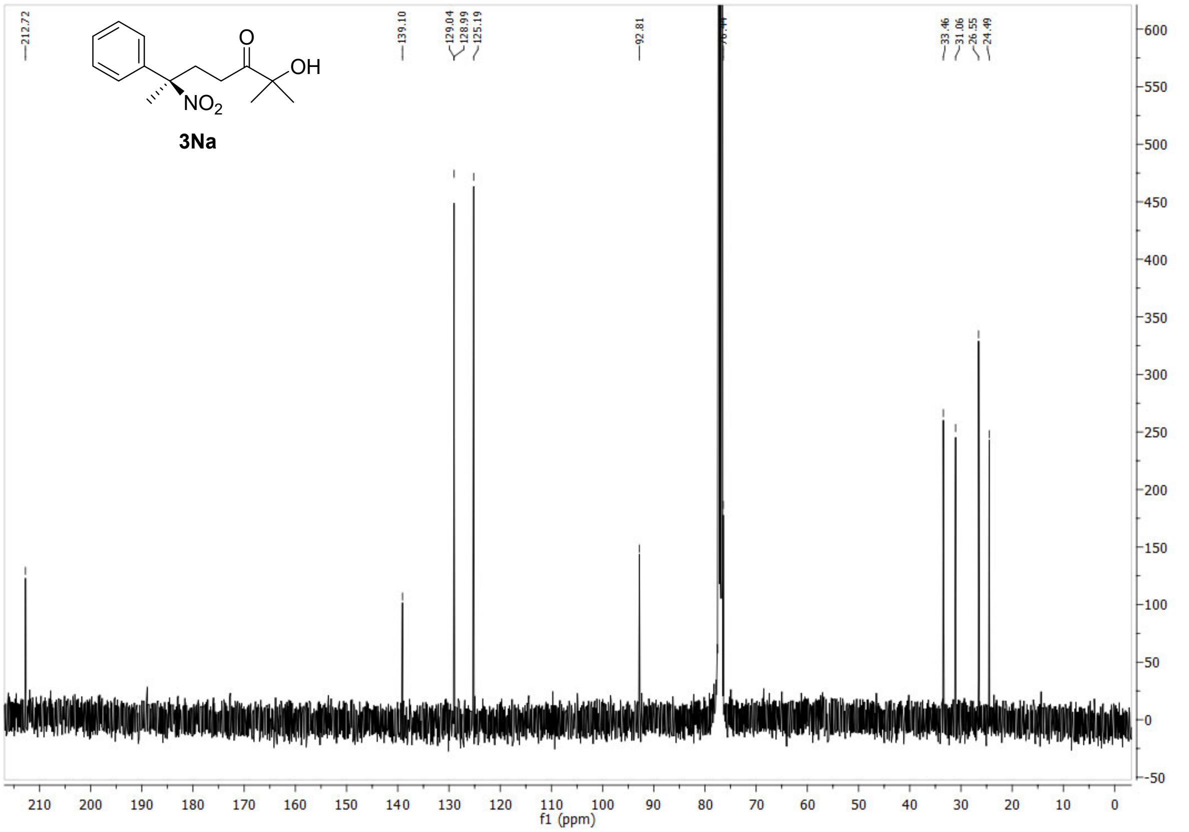

^{1}H NMR (400 MHz, $CDCl_3$) of compound **3Nc**:

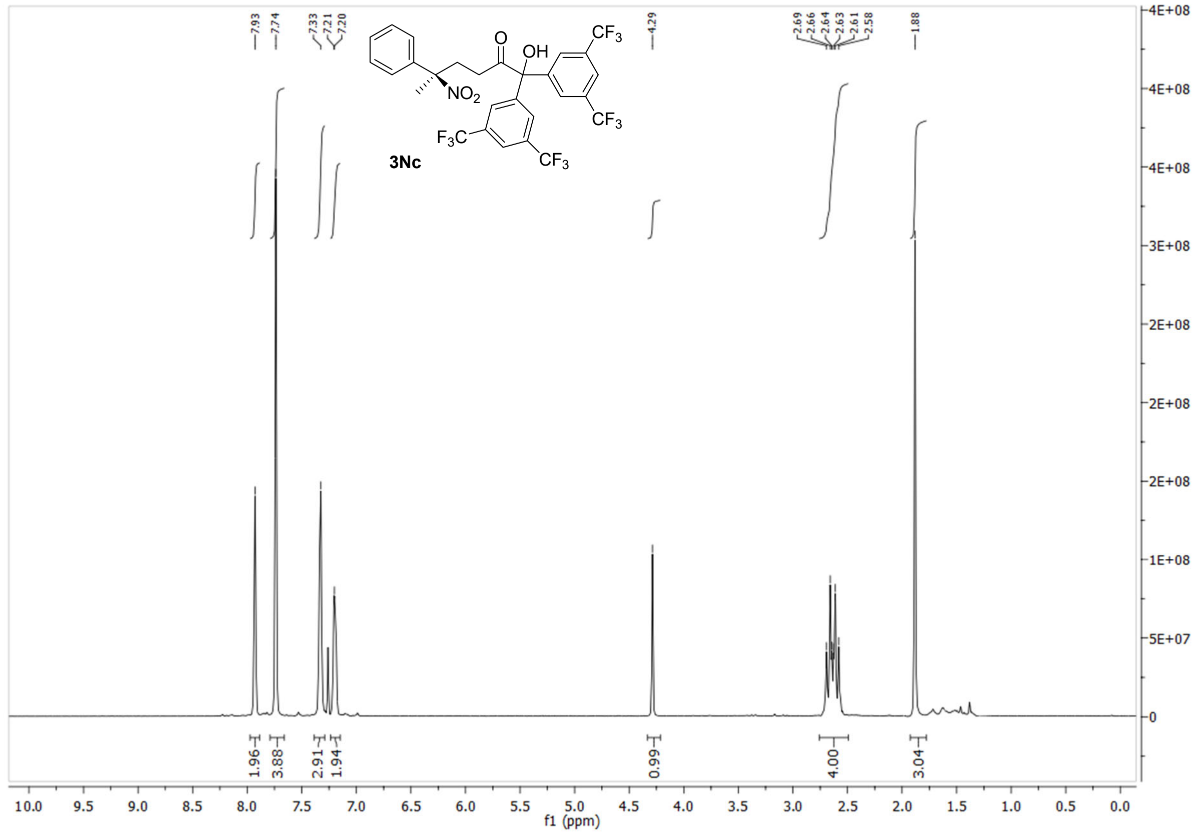

^{13}C NMR (100 MHz, $CDCl_3$) of compound **3Nc**:

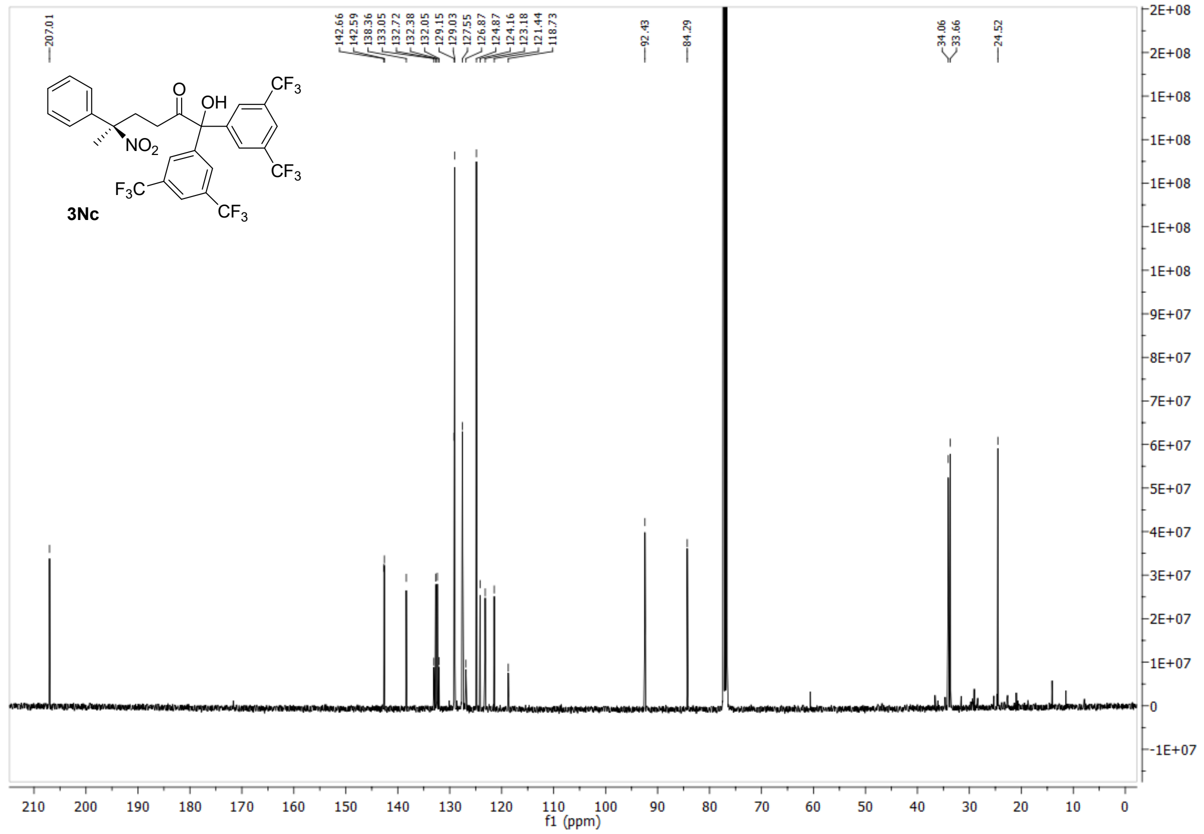

^{1}H NMR (400 MHz, $CDCl_3$) of compound **3Oa**:

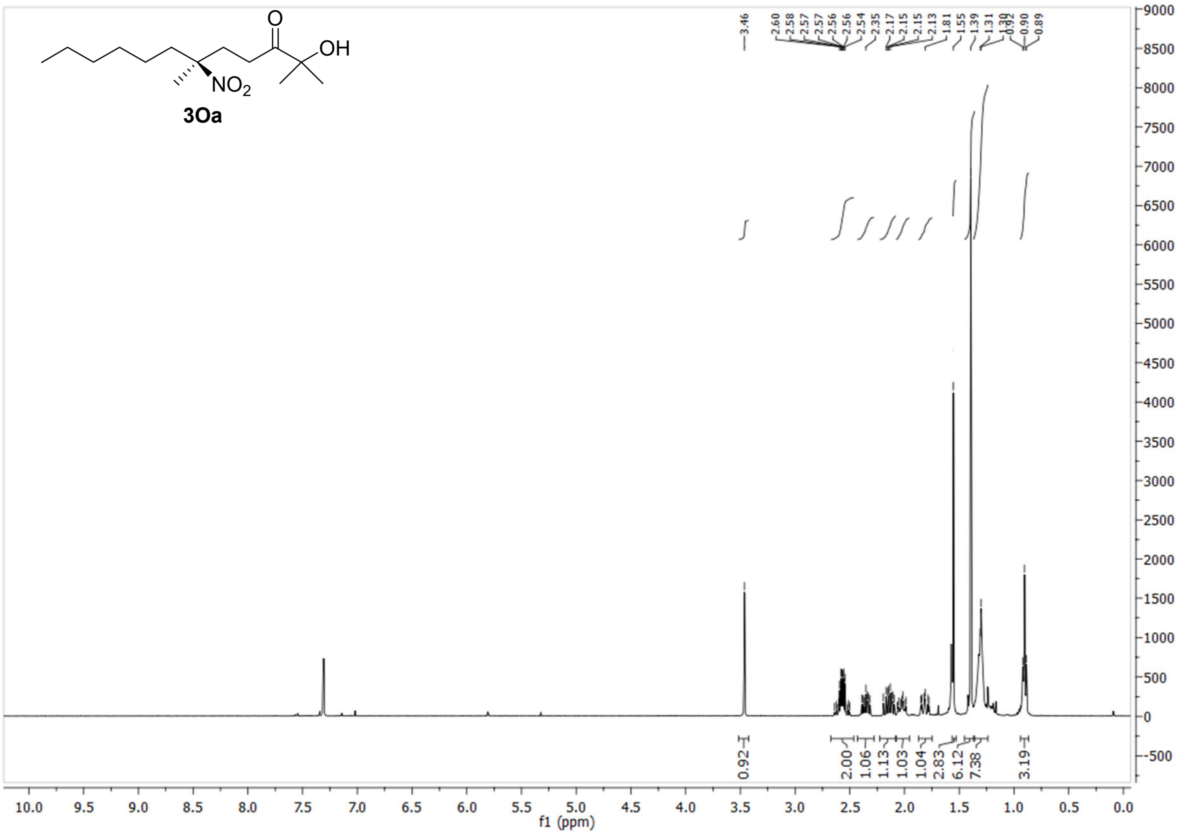

^{13}C NMR (100 MHz, $CDCl_3$) of compound **3Oa**:

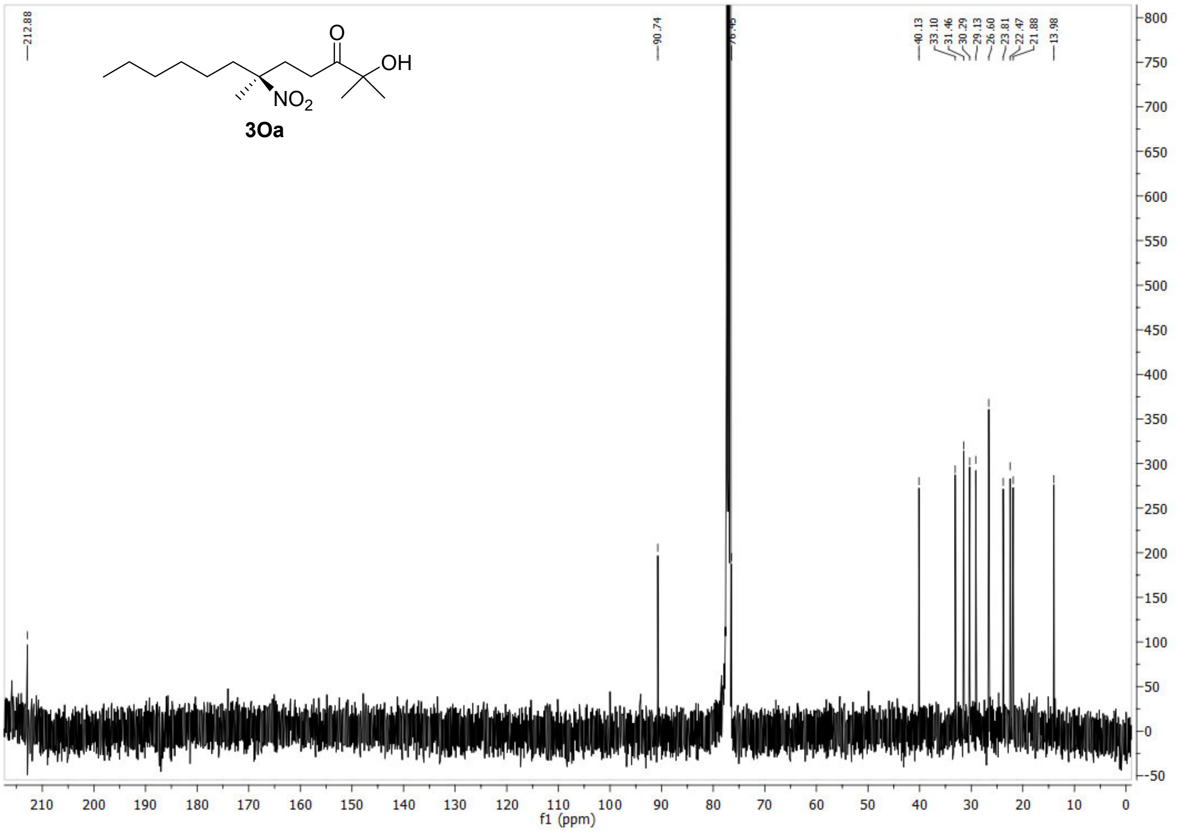

^{1}H NMR (400 MHz, $CDCl_3$) of compound **3Oc**:

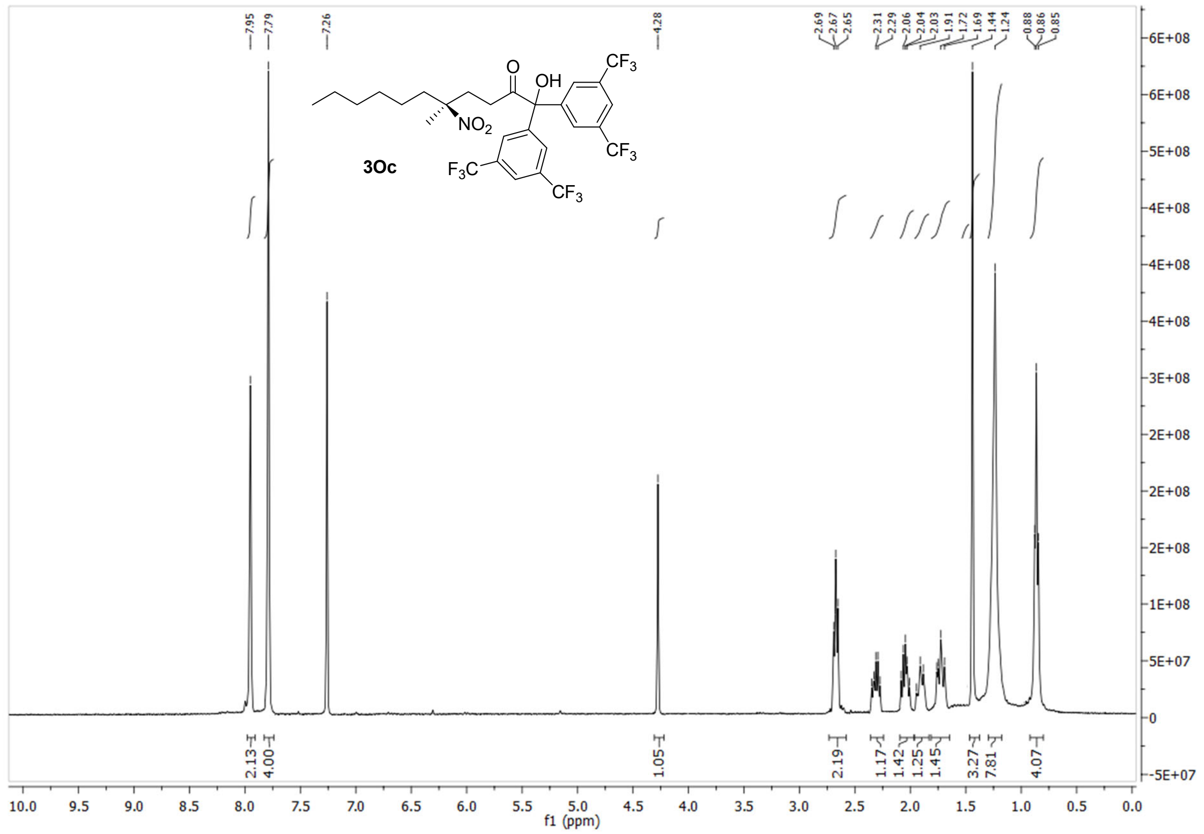

^{13}C NMR (100 MHz, $CDCl_3$) of compound **3Oc**:

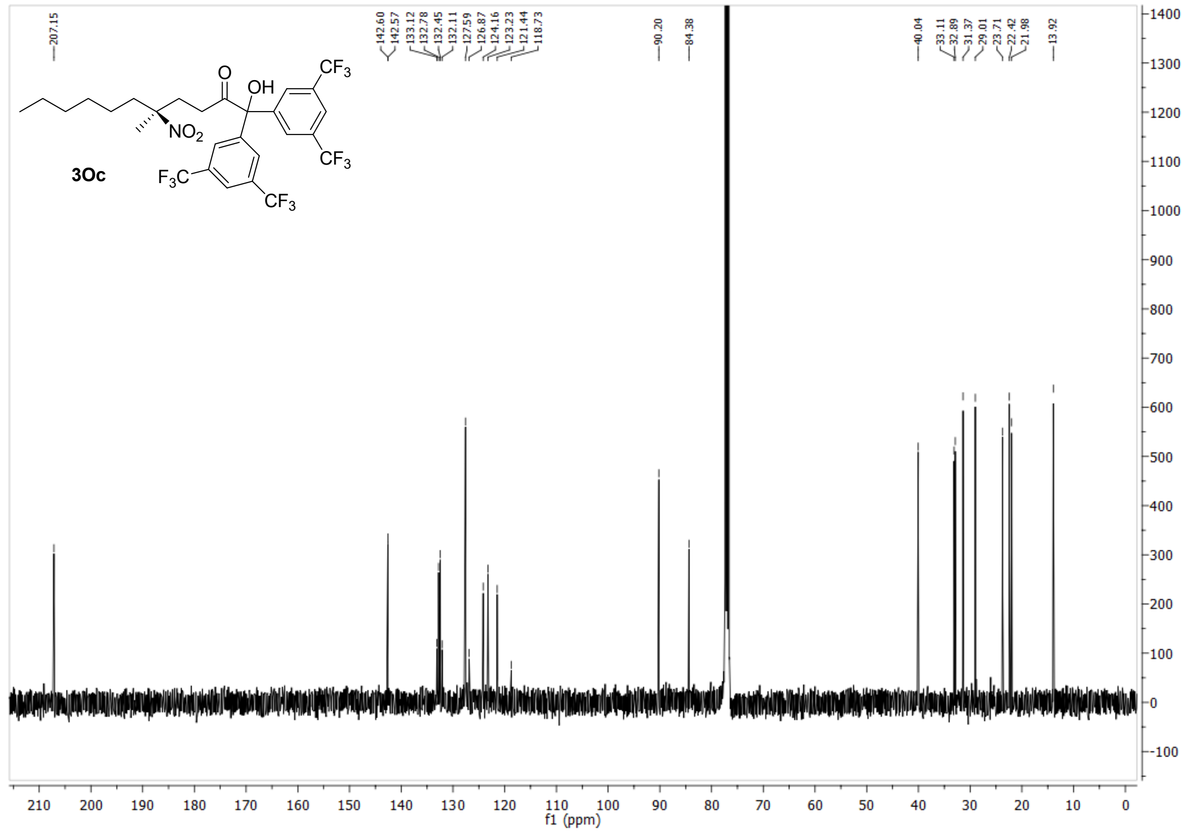

^{1}H NMR (400 MHz, $CDCl_3$) of compound **4A**:

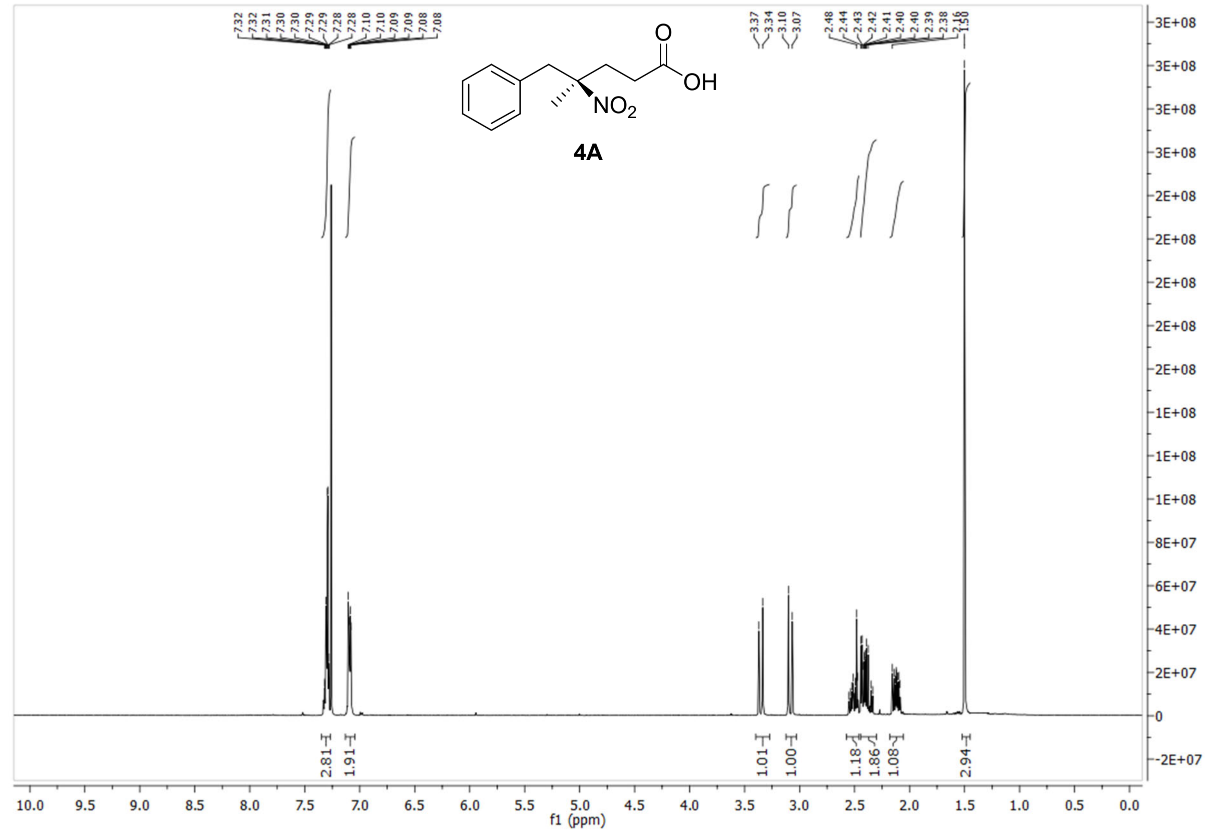

^{13}C NMR (100 MHz, $CDCl_3$) of compound **4A**:

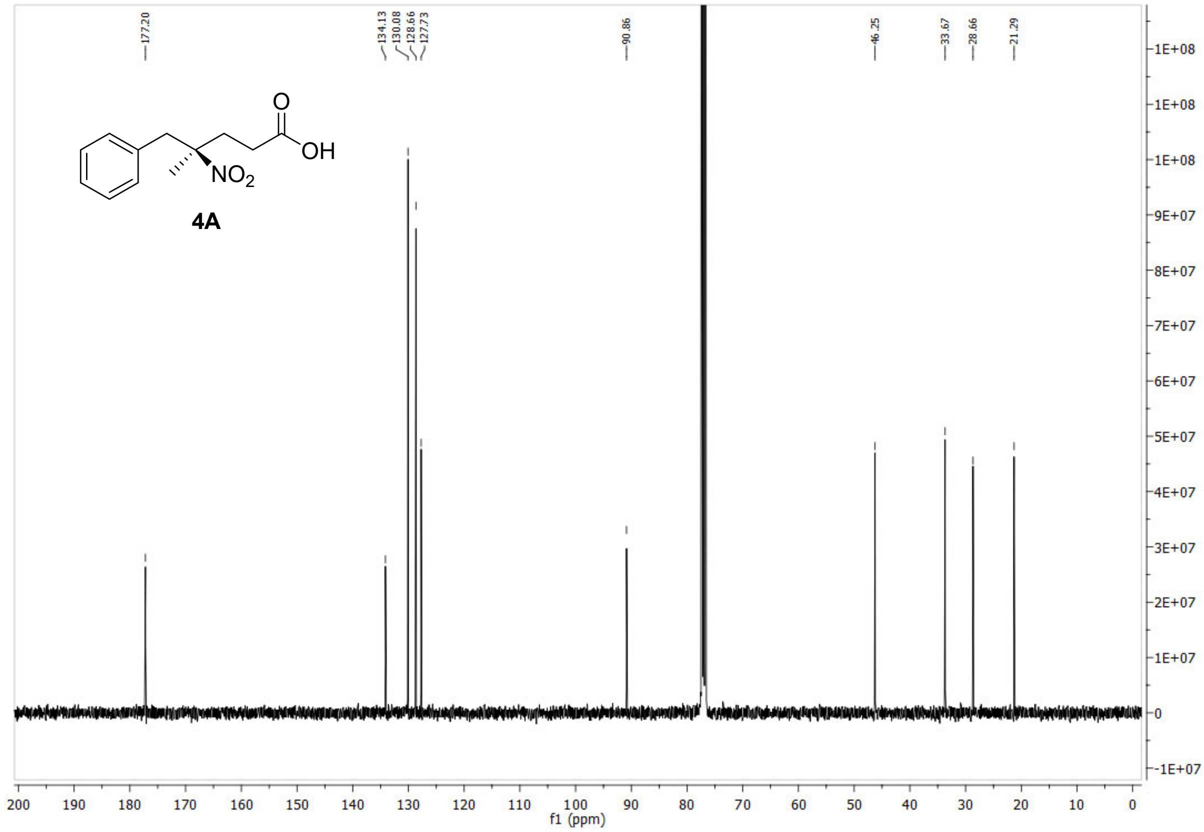

^{1}H NMR (400 MHz, $CDCl_3$) of compound **4C**:

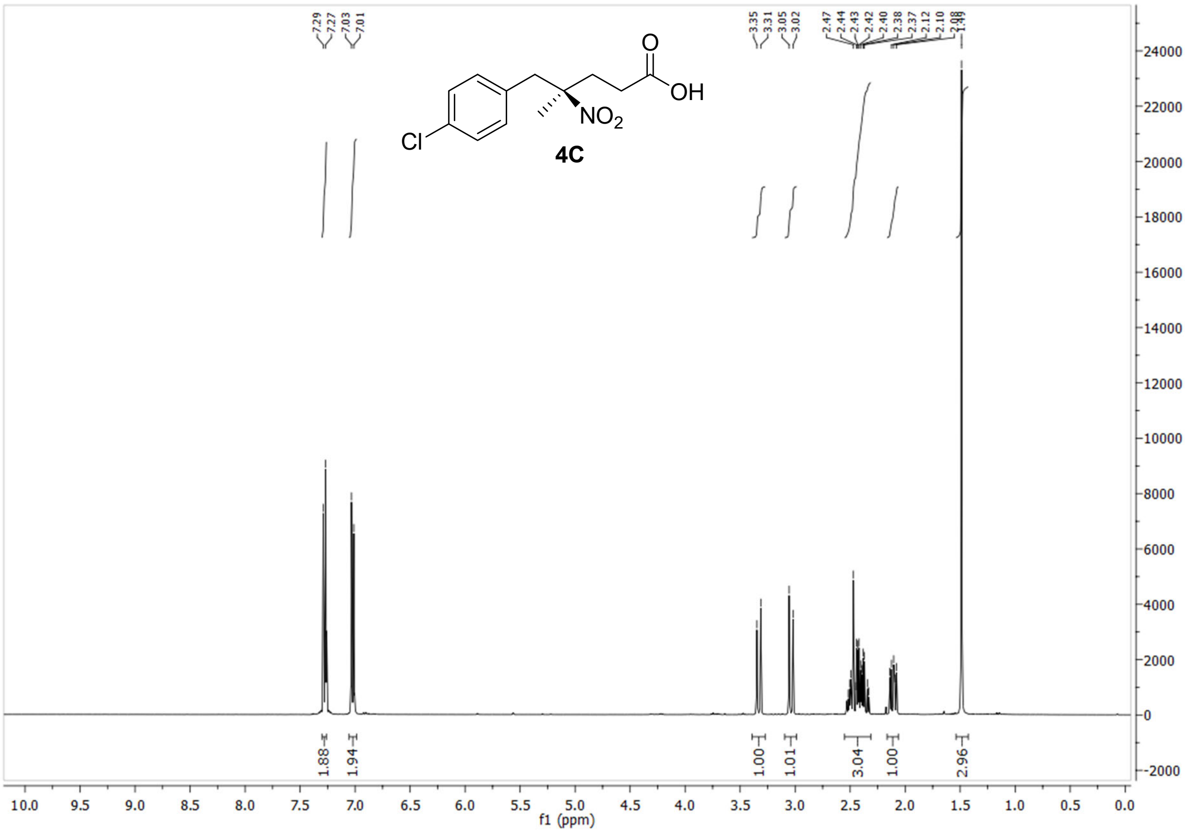

^{13}C NMR (100 MHz, $CDCl_3$) of compound **4C**:

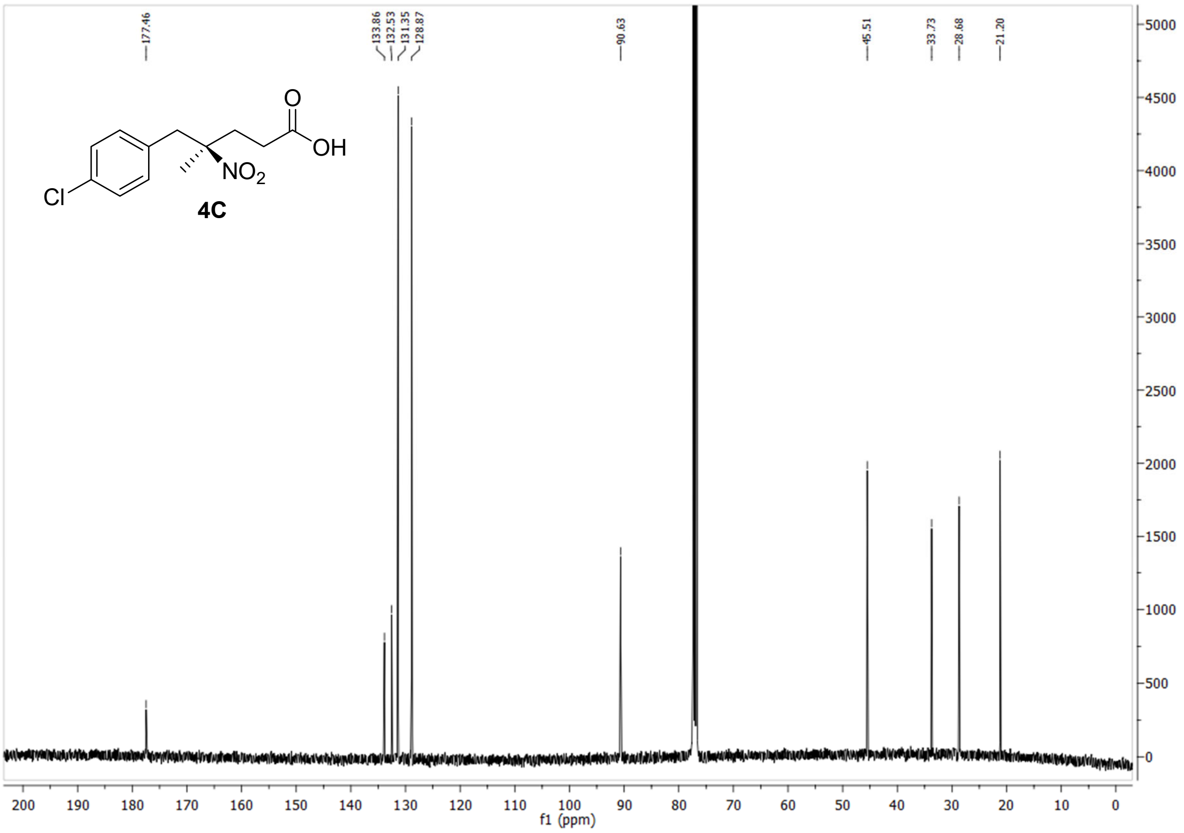

^{1}H NMR (400 MHz, $CDCl_3$) of compound **4G**:

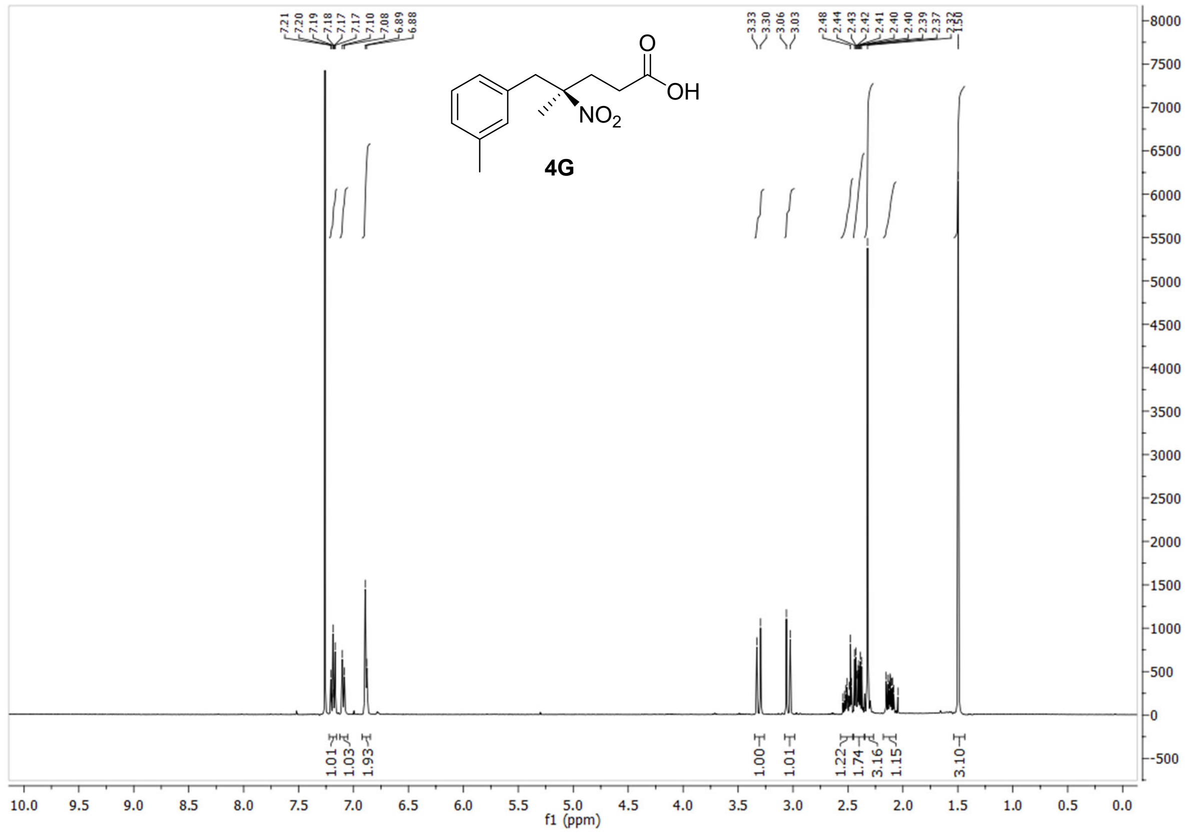

^{13}C NMR (100 MHz, $CDCl_3$) of compound **4G**:

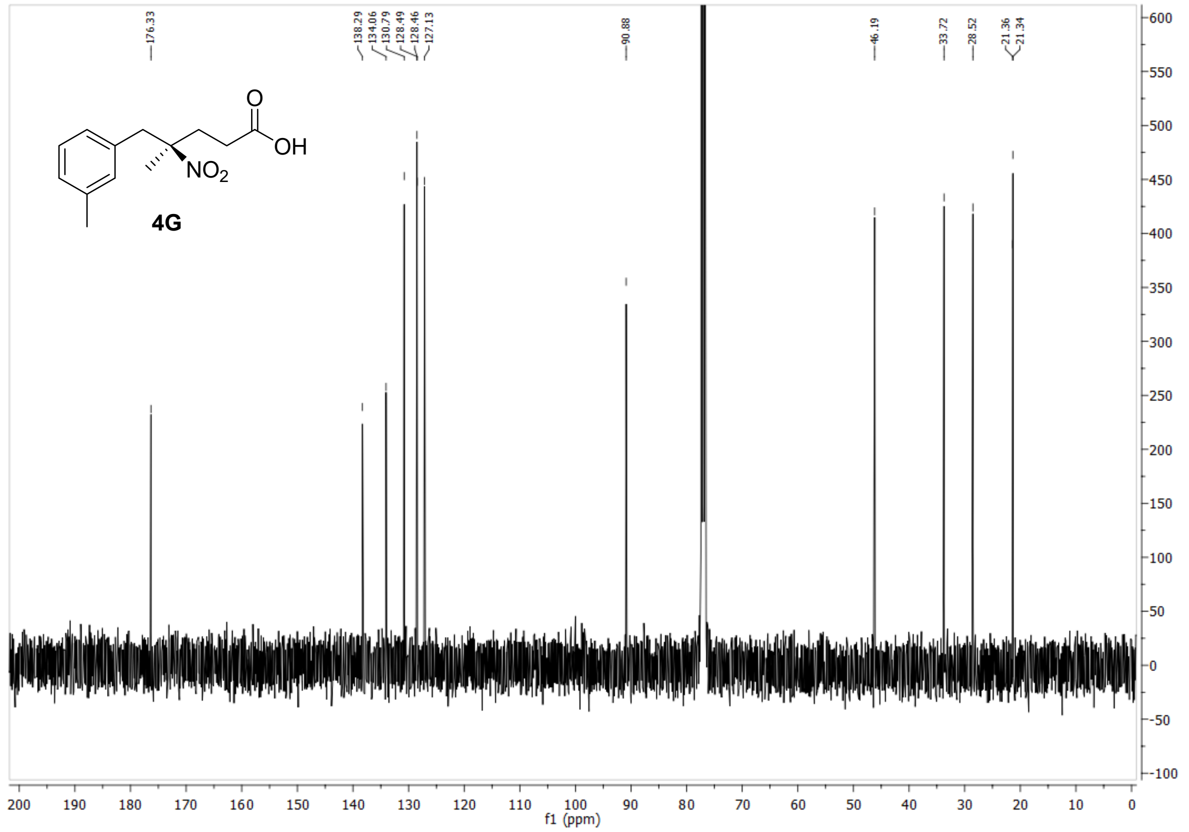

^{1}H NMR (400 MHz, $CDCl_3$) of compound **4L**:

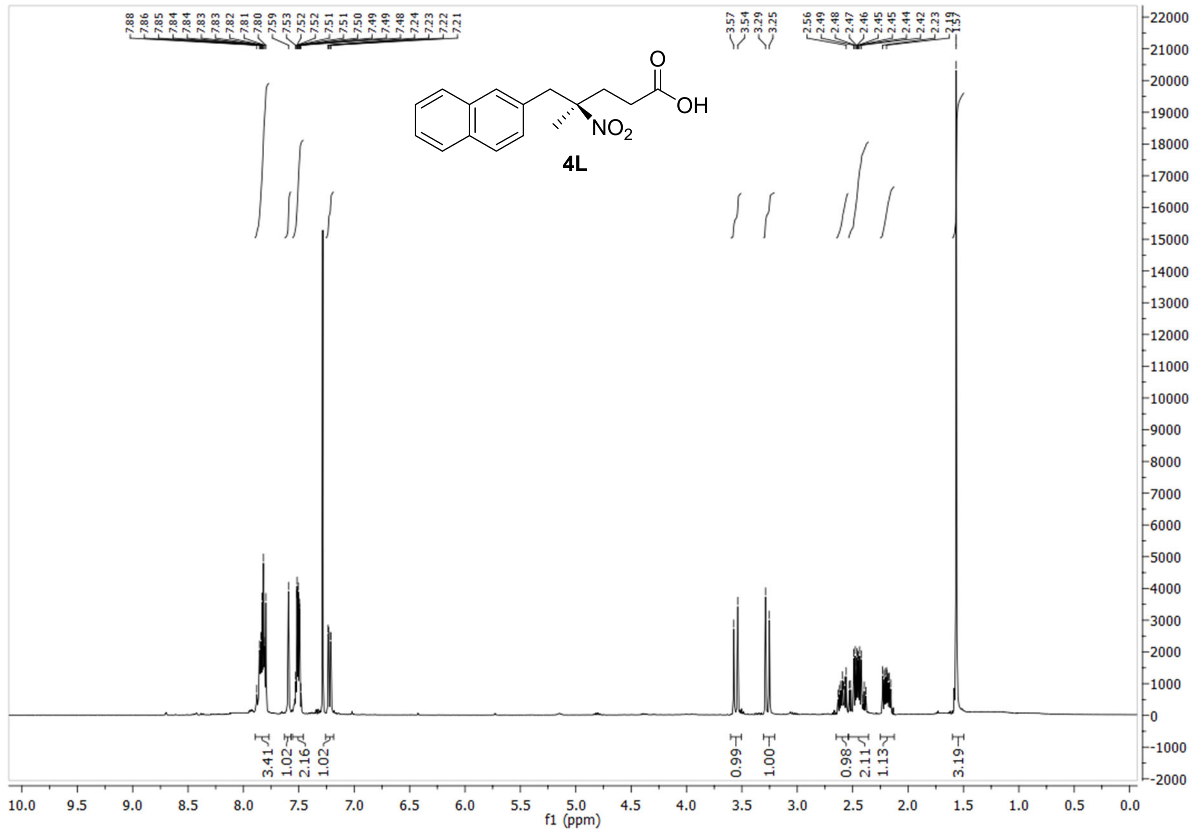

^{13}C NMR (100 MHz, $CDCl_3$) of compound **4L**:

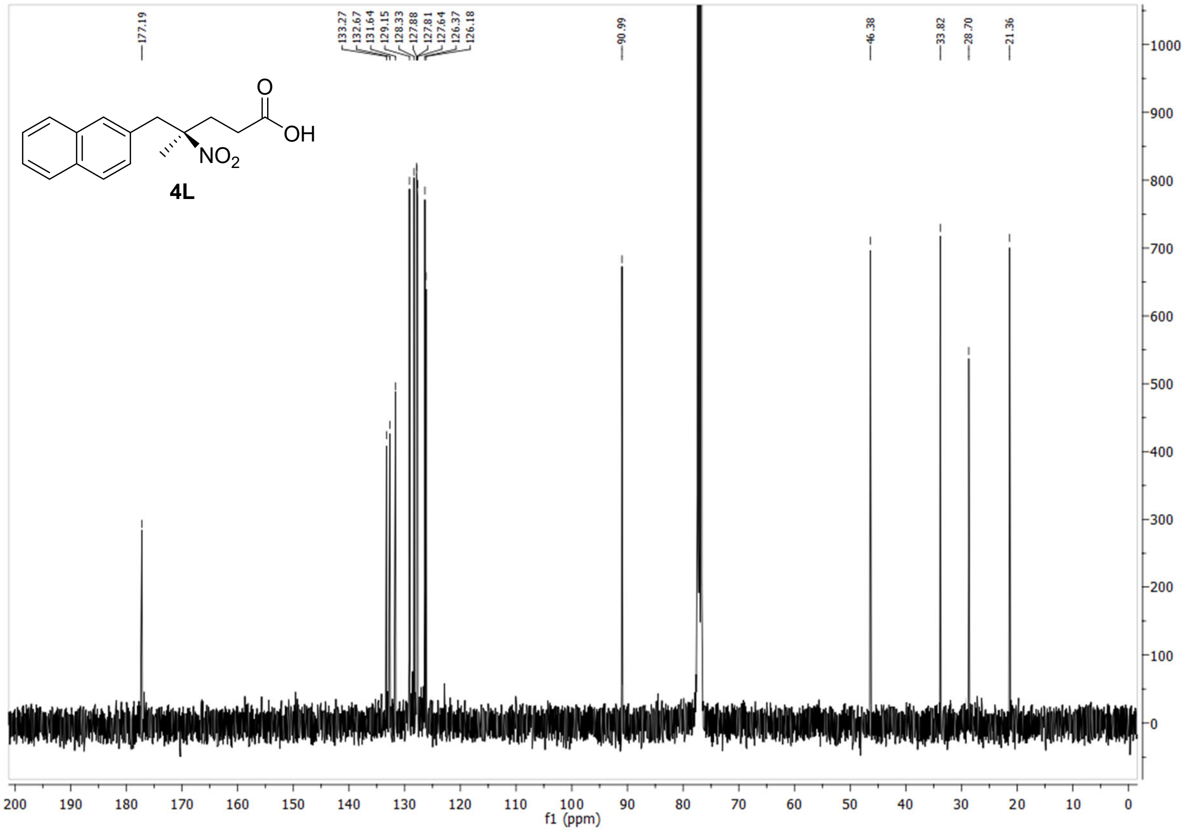

^1H NMR (400 MHz, $CDCl_3$) of compound **5**:

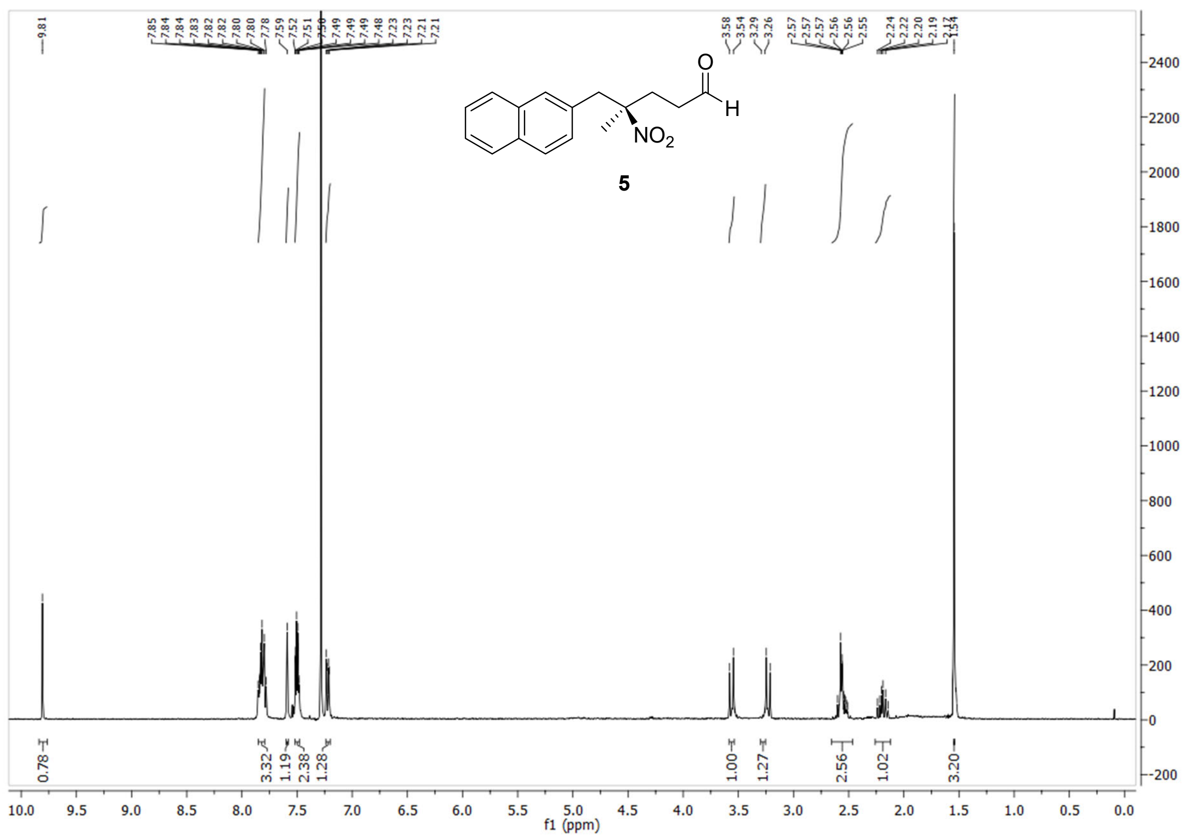

^{13}C NMR (100 MHz, $CDCl_3$) of compound **5**:

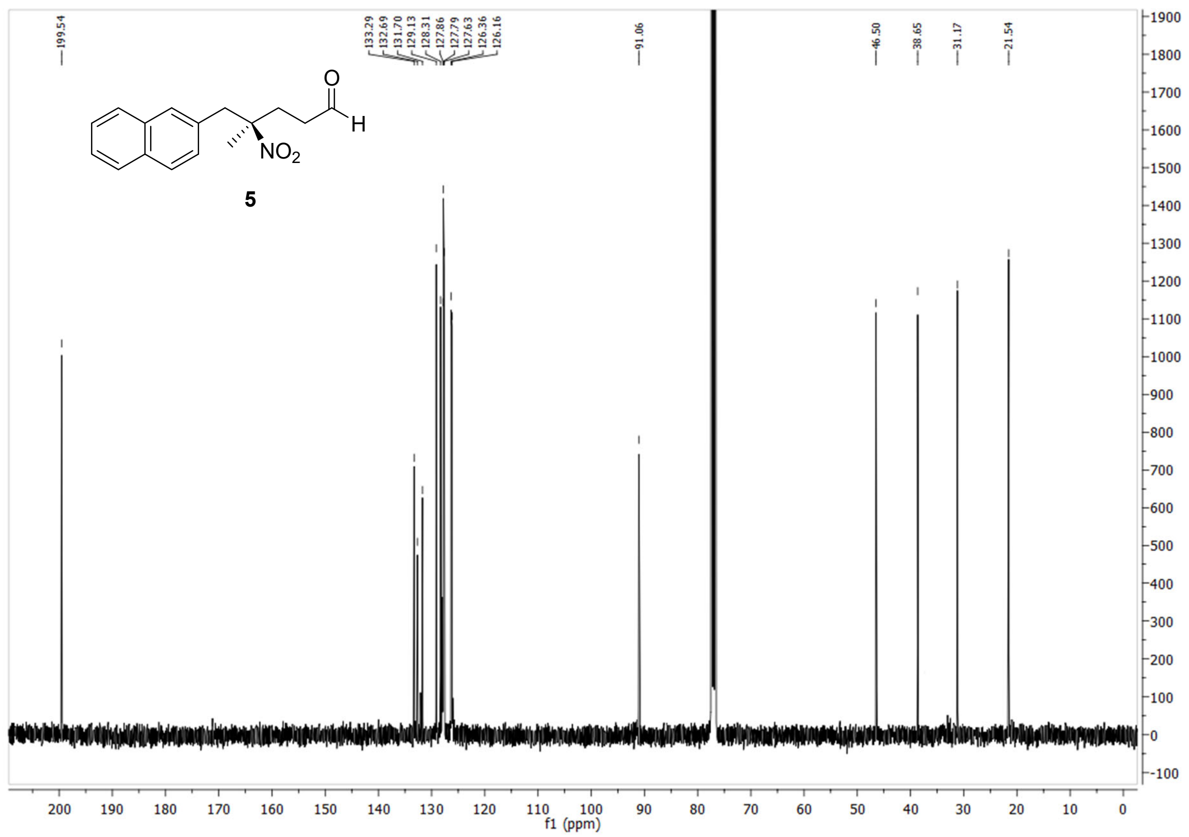

^{1}H NMR (400 MHz, $CDCl_3$) of compound **6A**:

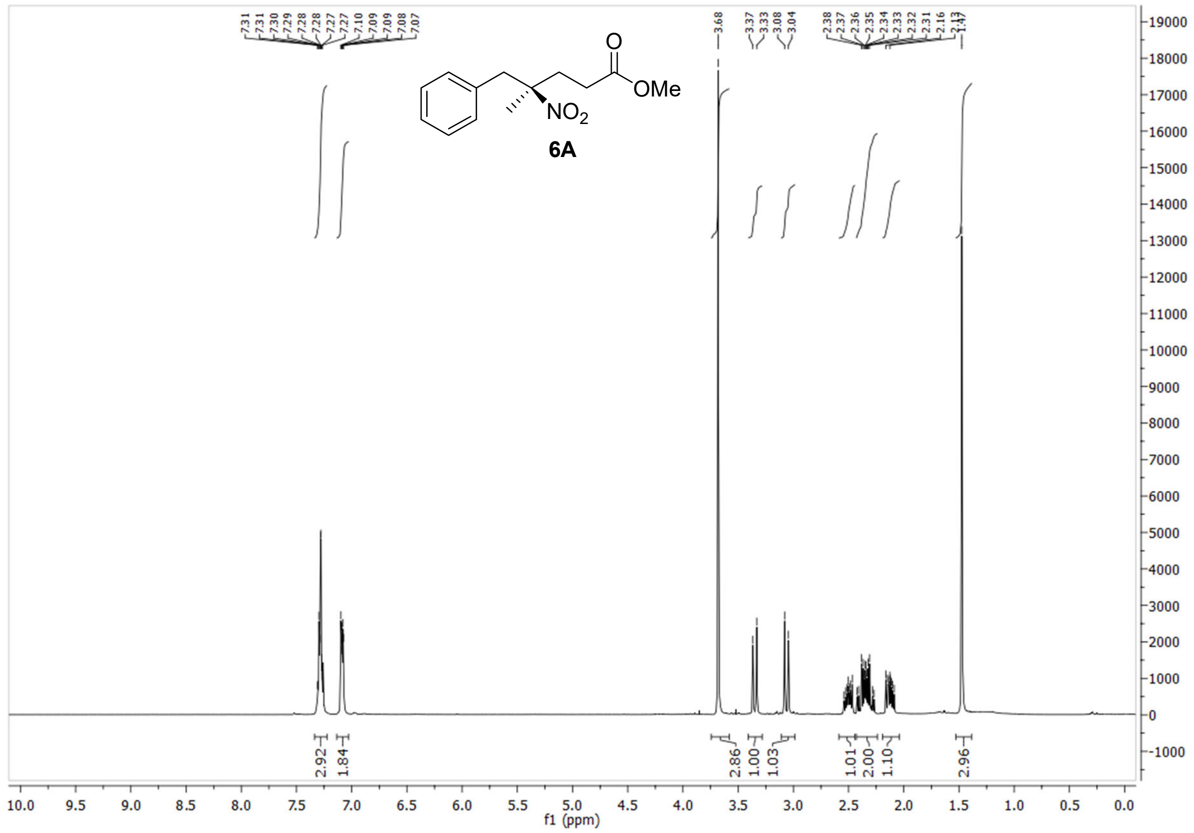

^{13}C NMR (100 MHz, $CDCl_3$) of compound **6A**:

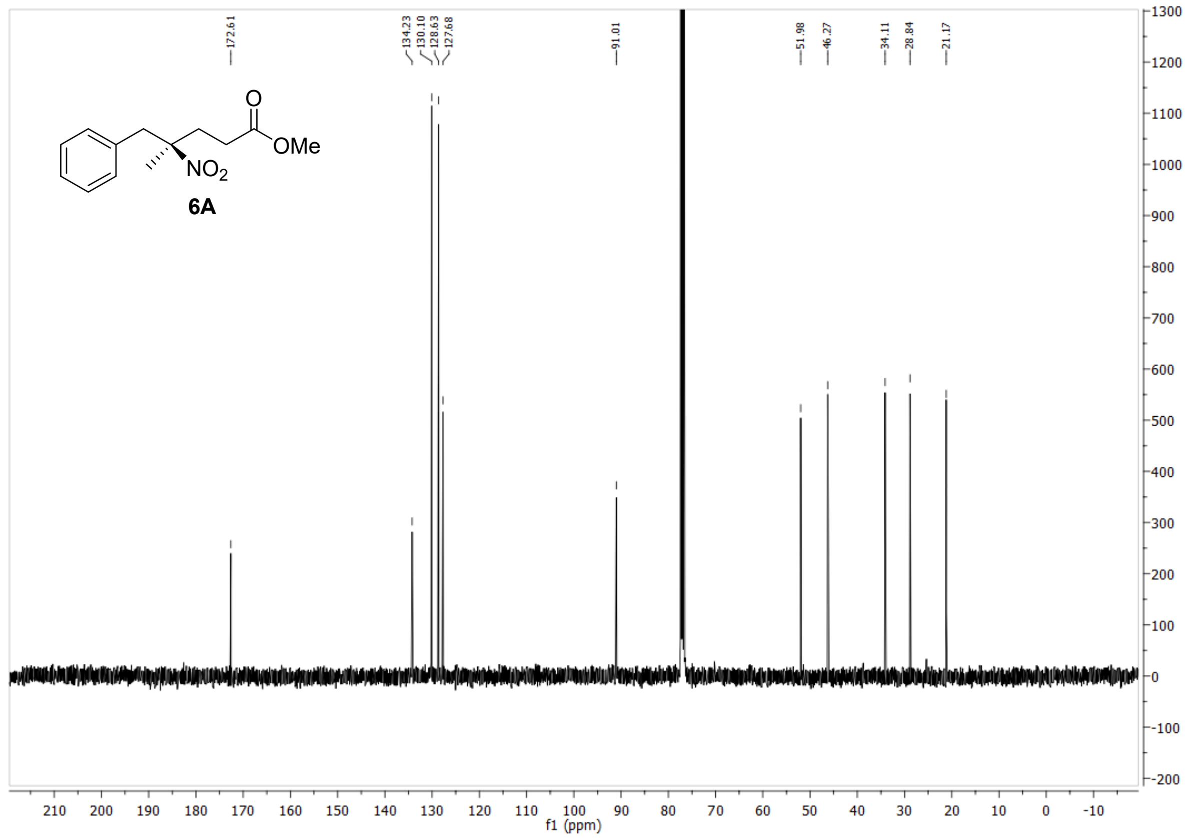

^1H NMR (400 MHz, $CDCl_3$) of compound **6C**:

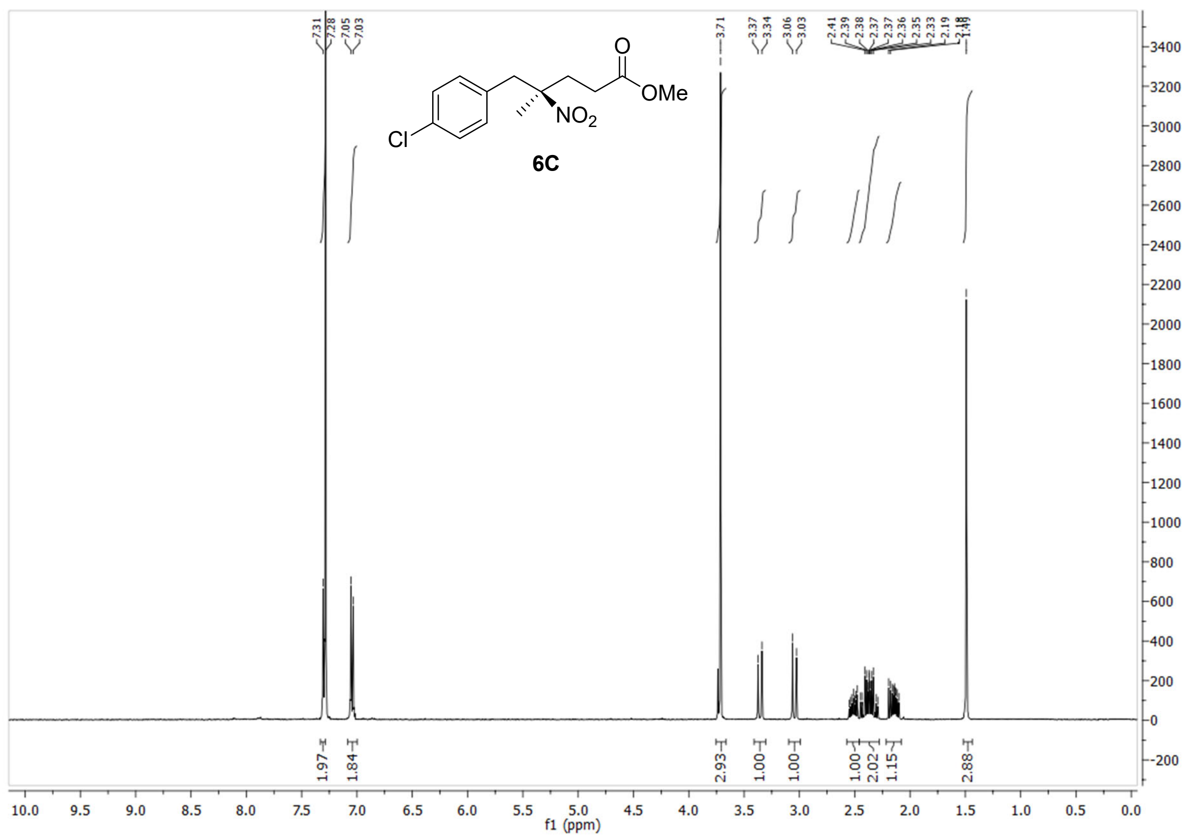

^{13}C NMR (100 MHz, $CDCl_3$) of compound **6C**:

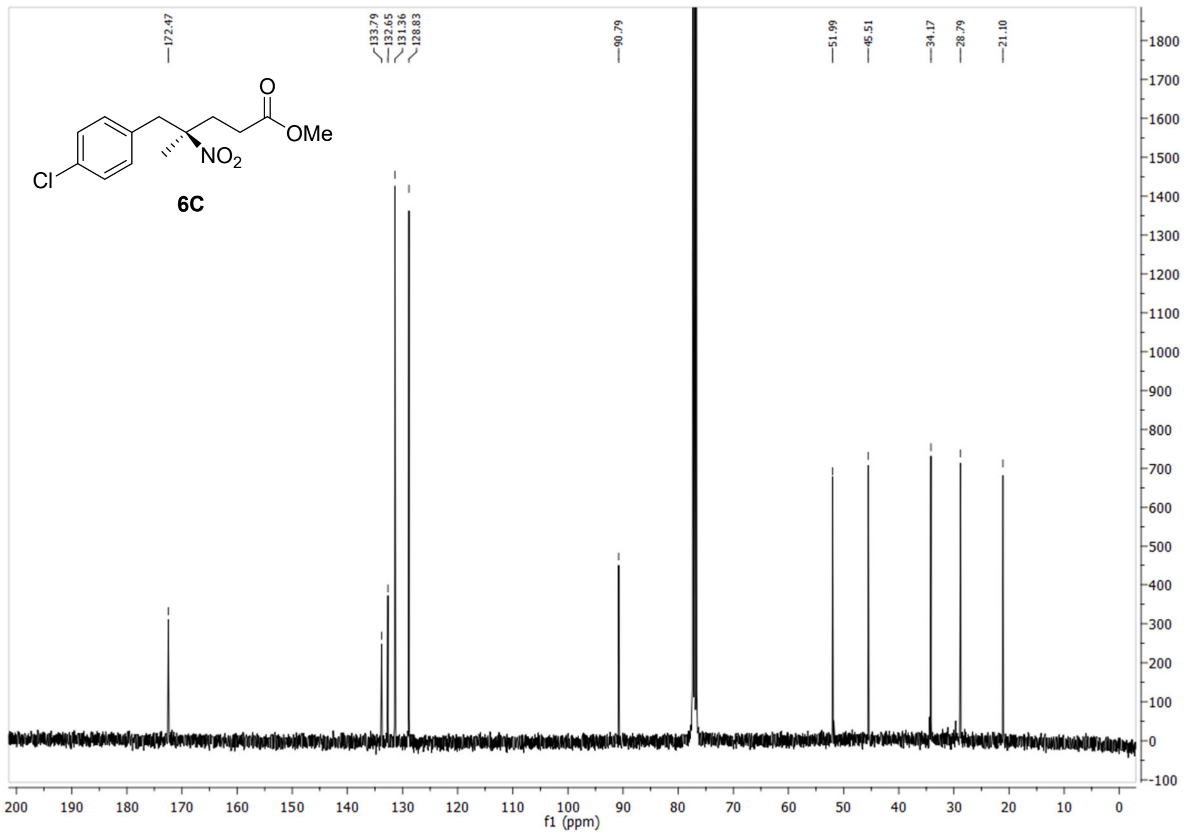

^{1}H NMR (400 MHz, $CDCl_3$) of compound **6G**:

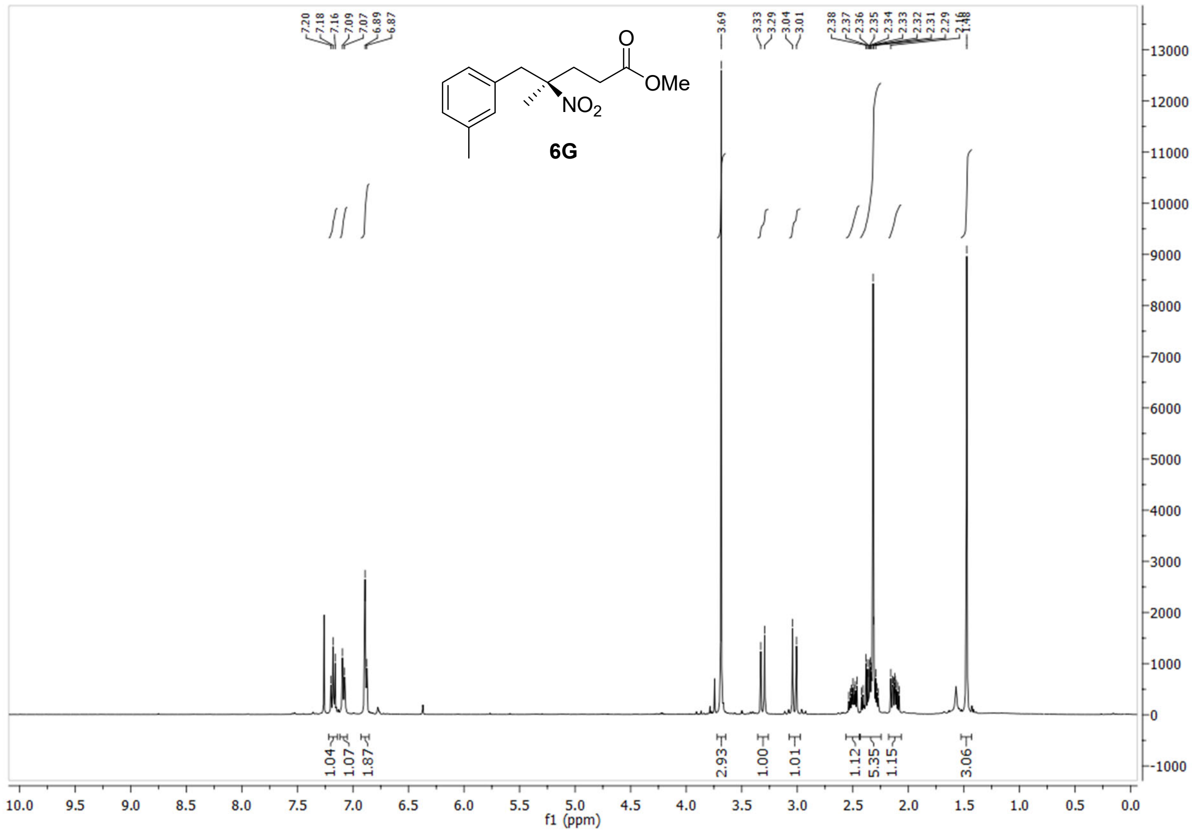

^{13}C NMR (100 MHz, $CDCl_3$) of compound **6G**:

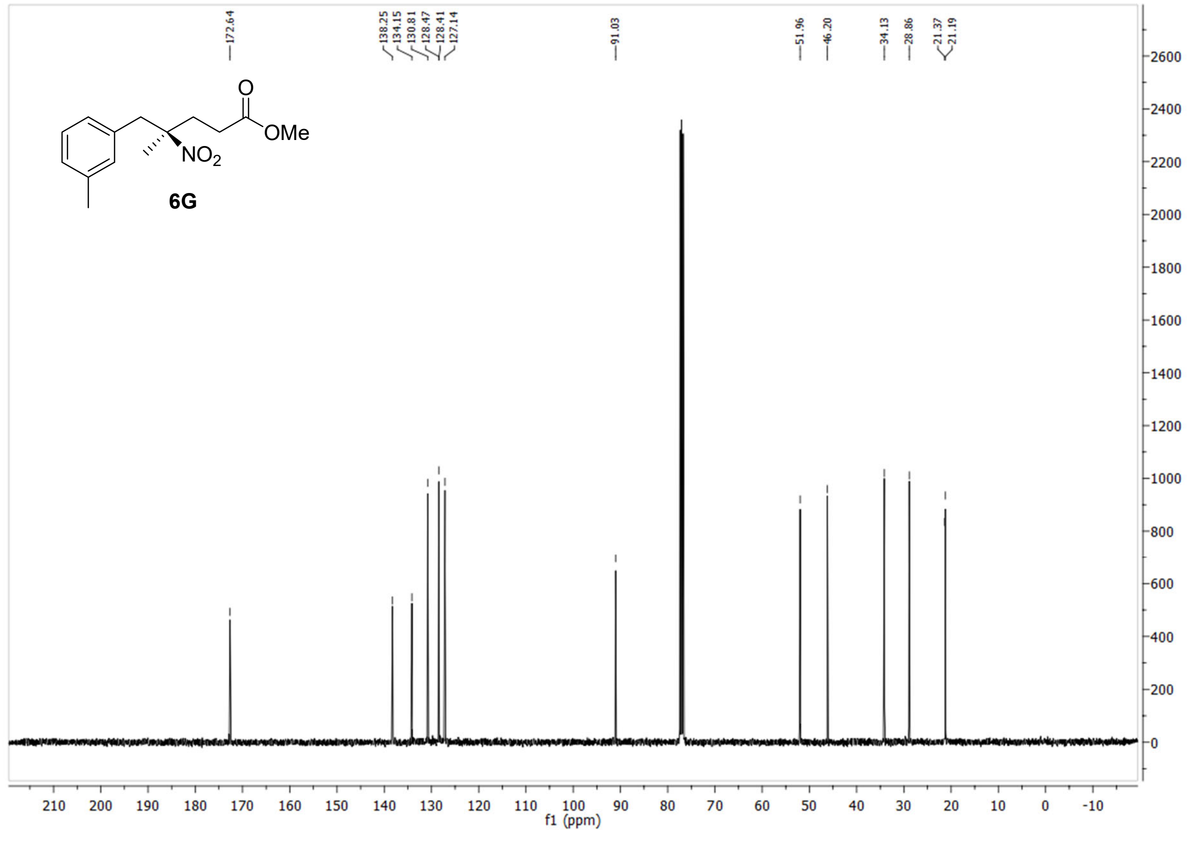

^{1}H NMR (400 MHz, $CDCl_3$) of compound **7C**:

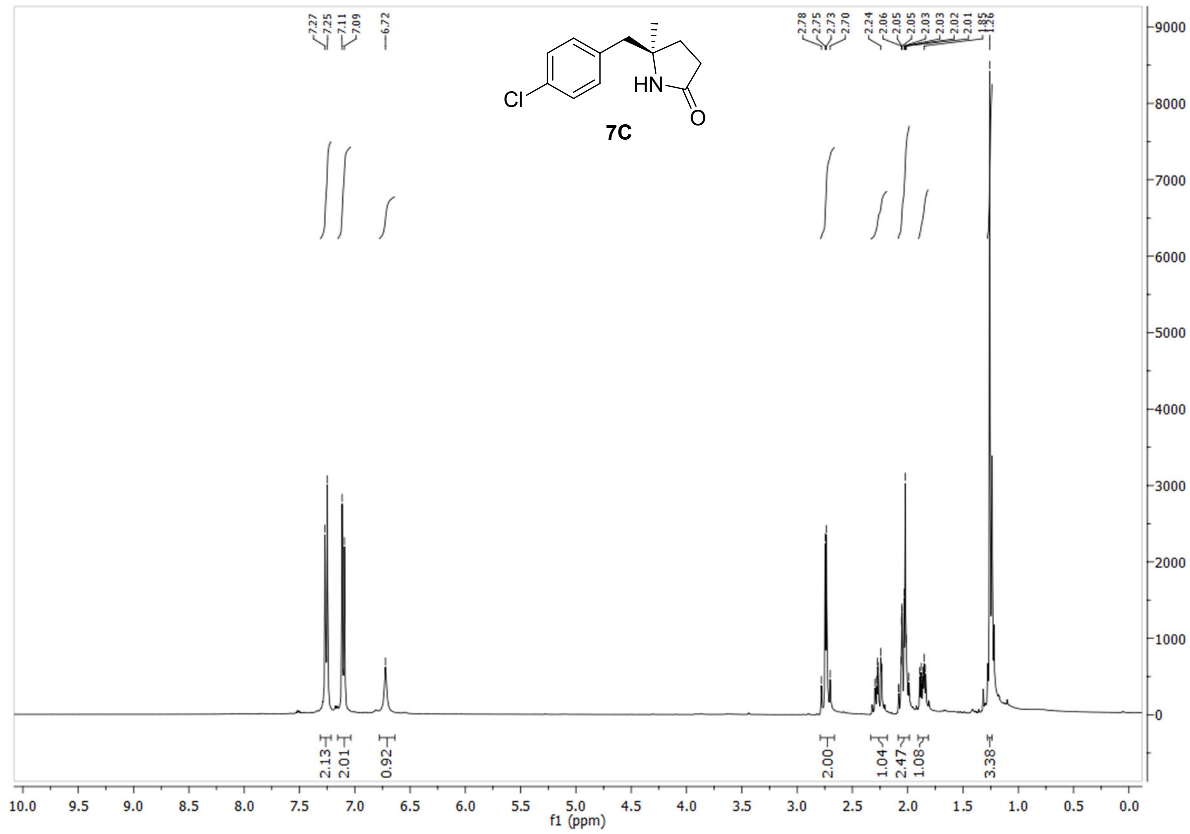

^{13}C NMR (100 MHz, $CDCl_3$) of compound **7C**:

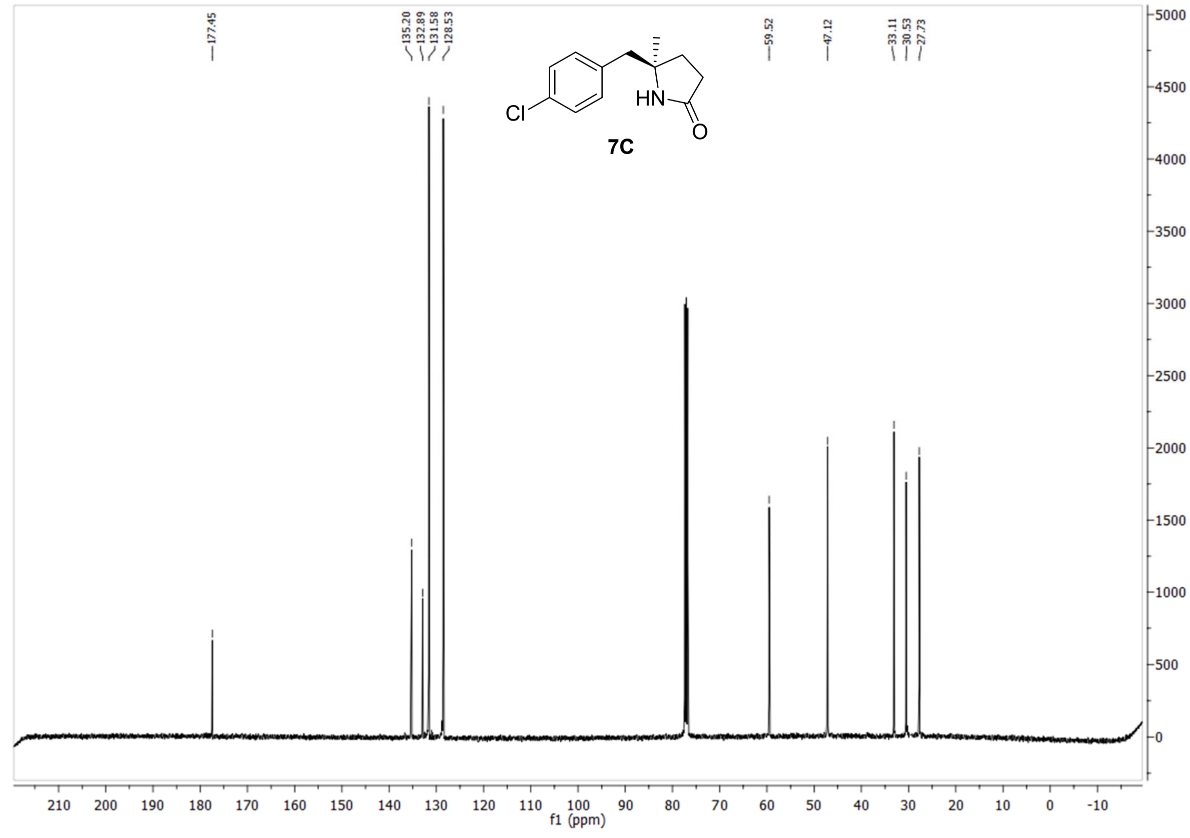

^{1}H NMR (400 MHz, $CDCl_3$) of compound **7G**:

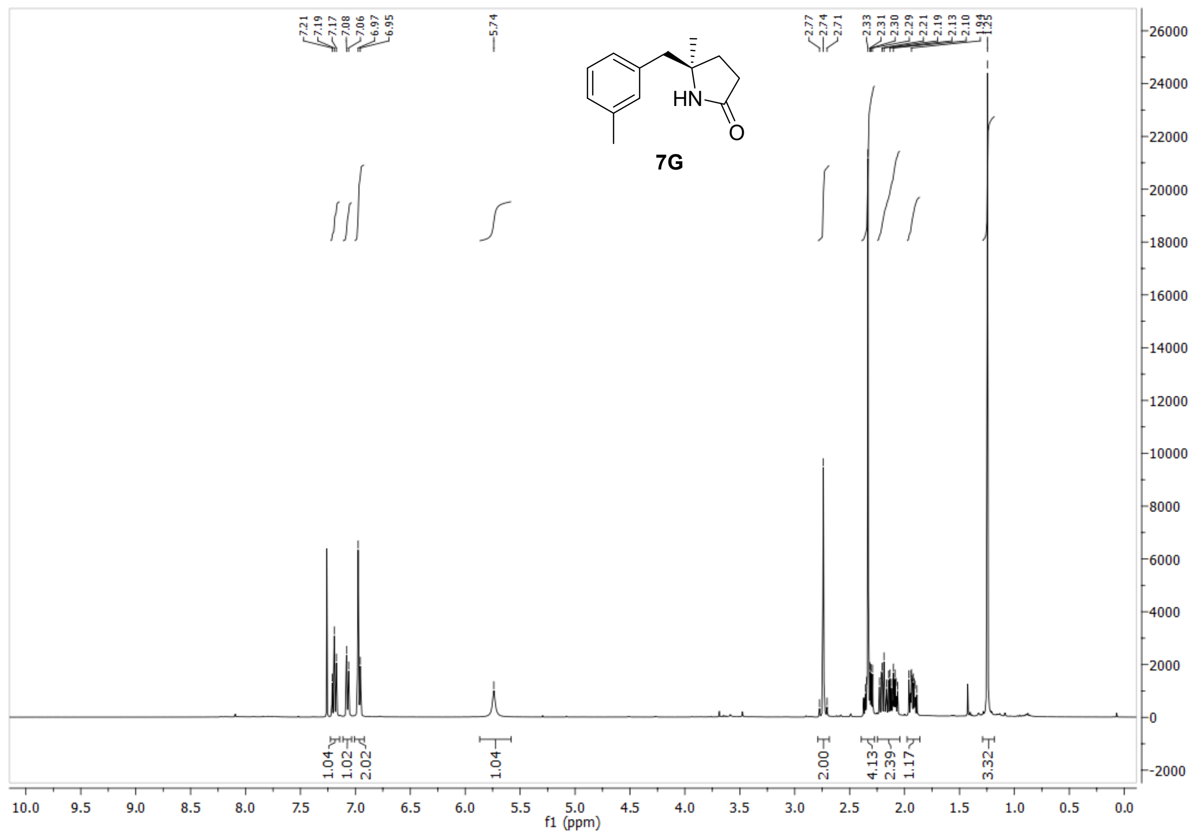

^{13}C NMR (100 MHz, $CDCl_3$) of compound **7G**:

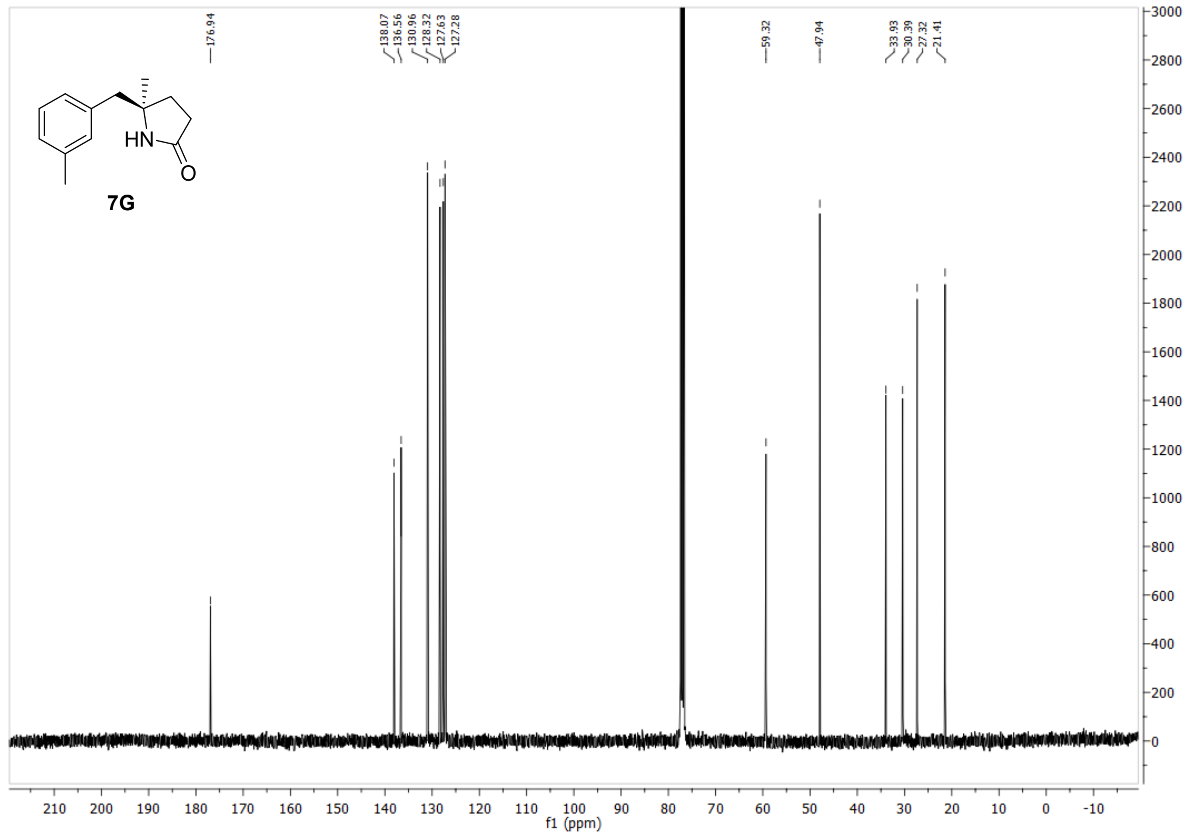

^{1}H NMR (400 MHz, $CDCl_3$) of compound **8**:

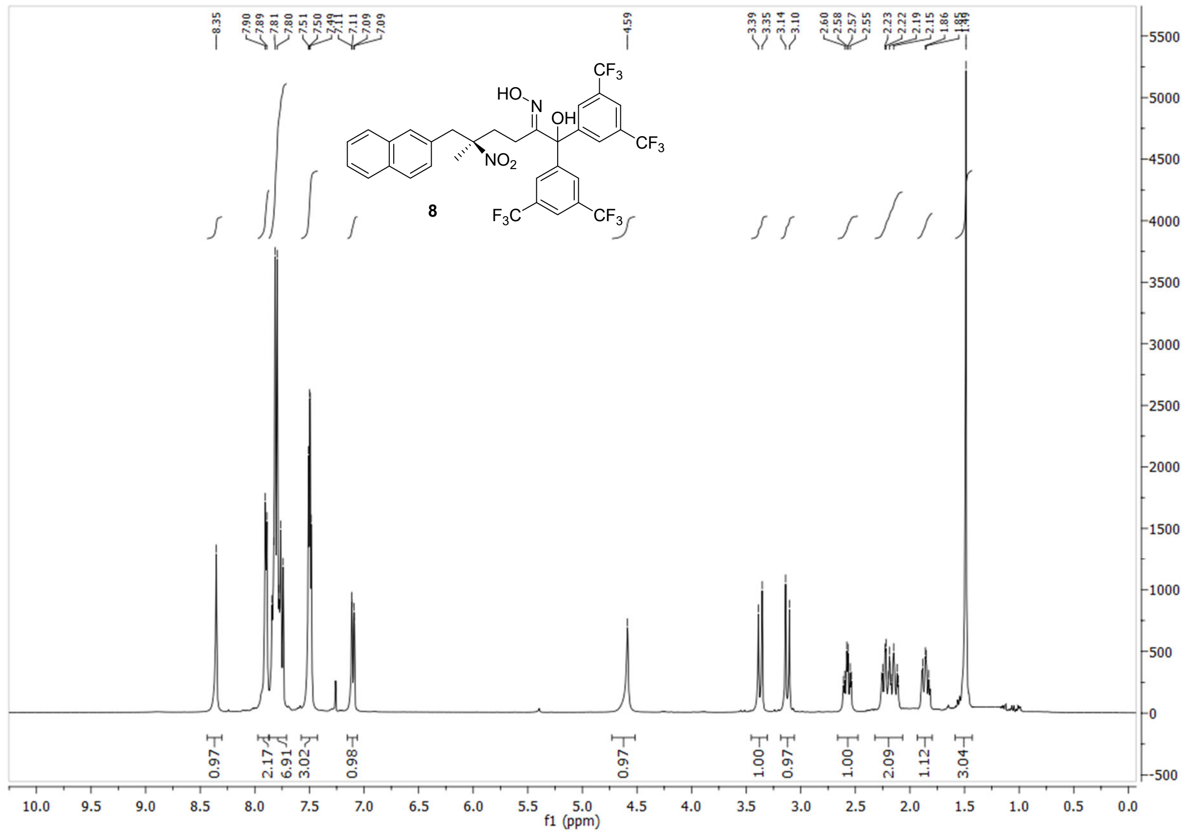

^{13}C NMR (100 MHz, $CDCl_3$) of compound **8**:

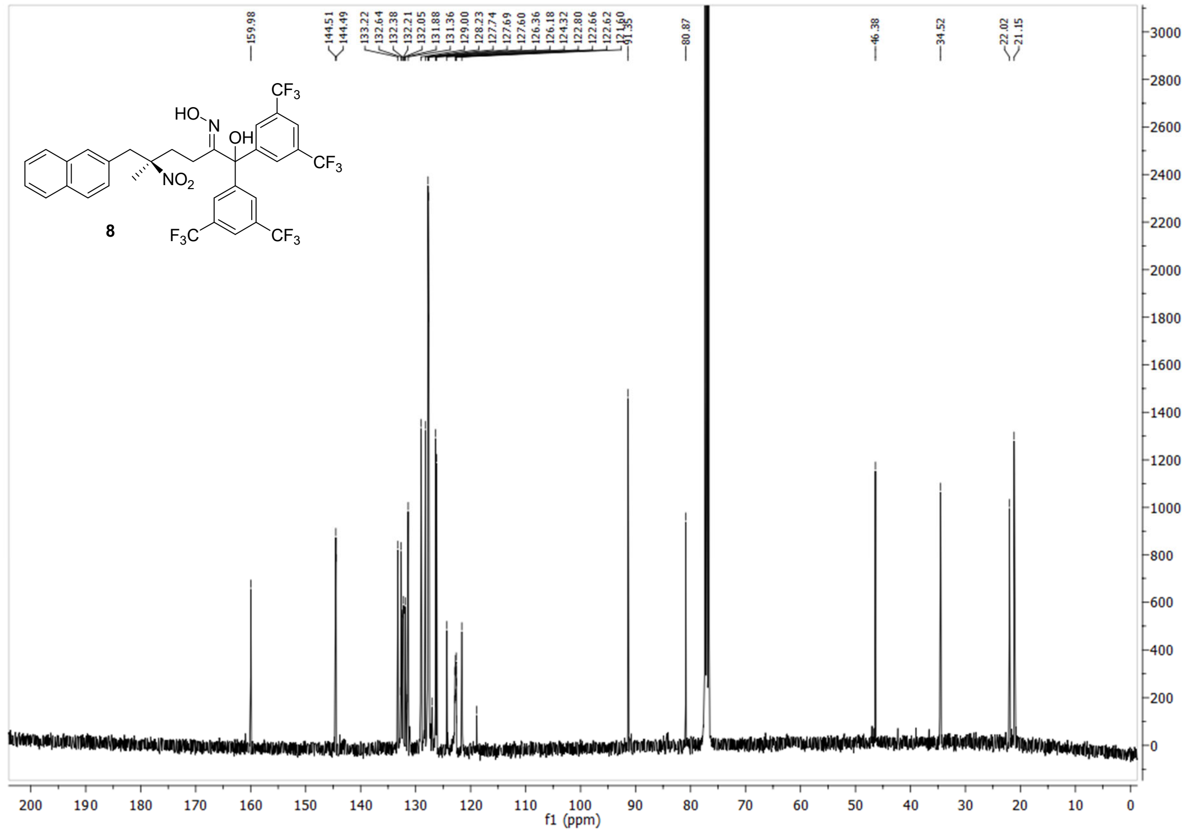

NMR (400 MHz, $CDCl_3$) of compound **9**:

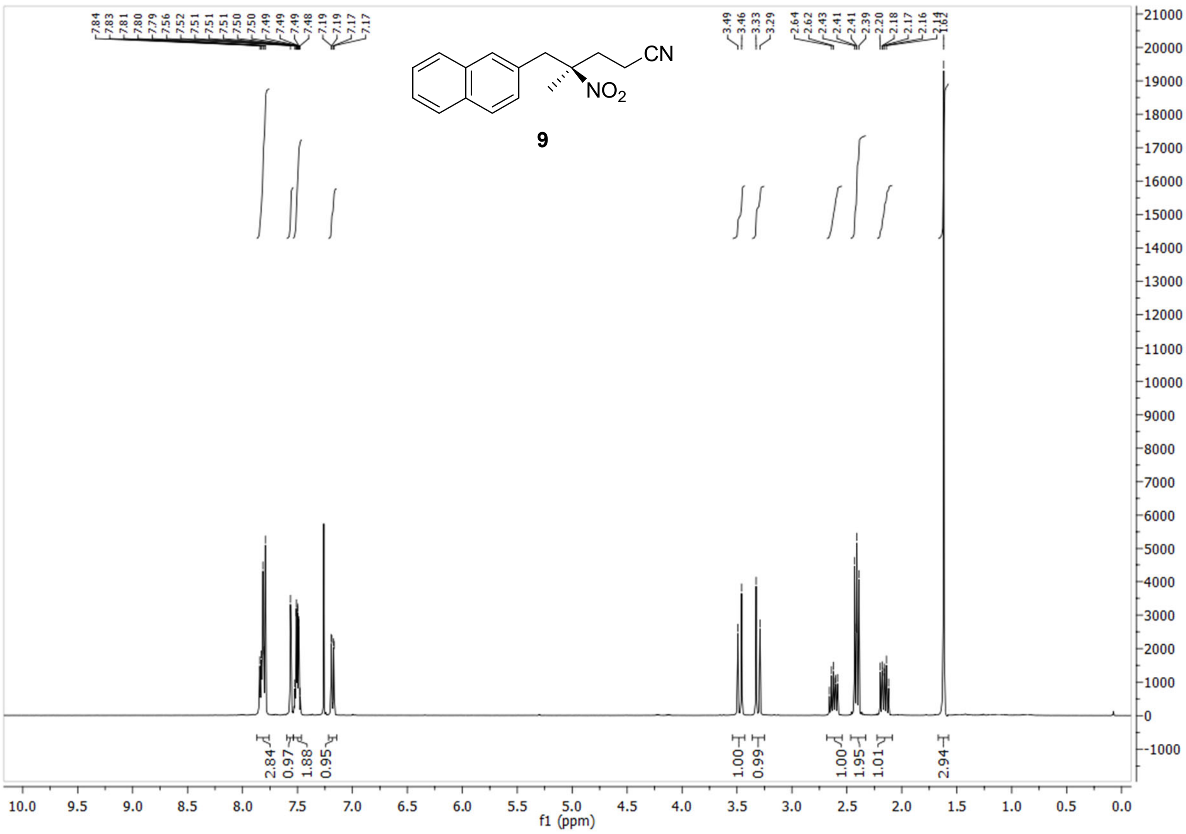

^{13}C NMR (100 MHz, $CDCl_3$) of compound **9**:

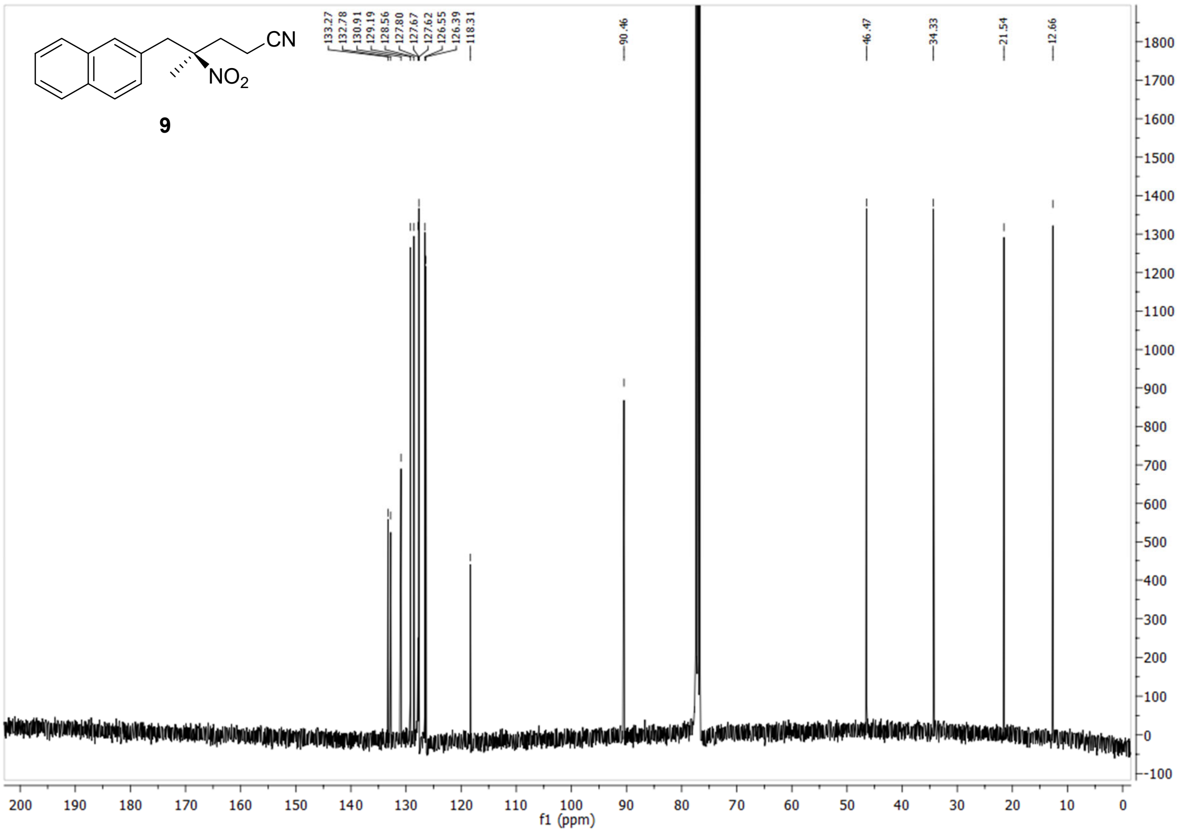

7. HPLC chromatograms for *e.r.* determinations

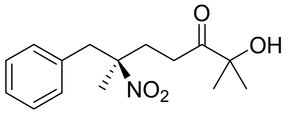

3Aa

The enantiomeric excess was determined by HPLC analysis (Daicel Chiralpak IA, Hexane/^{i}PrOH = 95/5, flow rate = 0.5 mL/min, λ = 202 nm).

(±) 3Aa

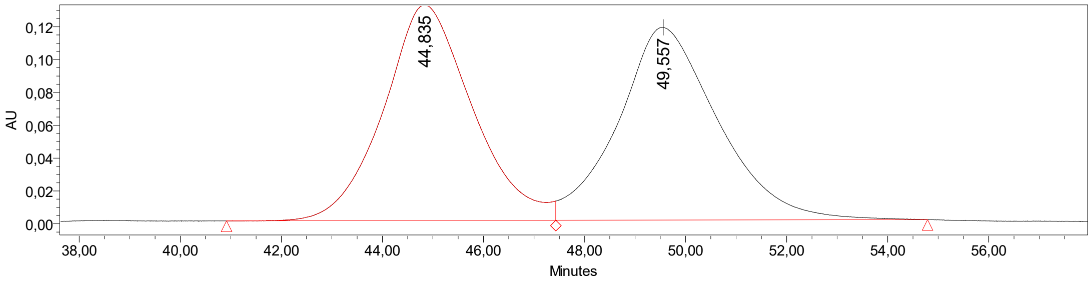

	Retention Time	% Area
1	44,835	49,94
2	49,557	50,06

3Aa

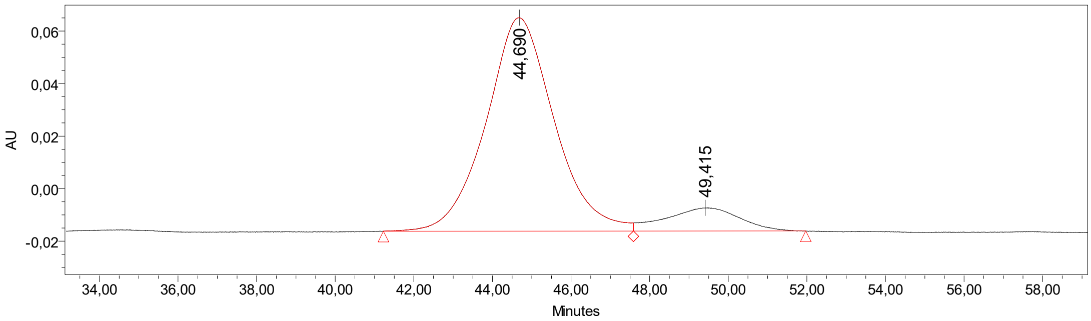

	Retention Time	% Area
1	44,690	89,75
2	49,415	10,25

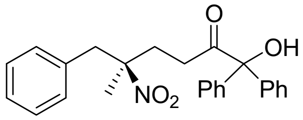

3Ab

The enantiomeric ratio was determined by HPLC analysis (Daicel Chiralpak IA, Hexane/^{i}PrOH = 90/10, flow rate = 0.5 mL/min, λ = 202 nm).

(±) 3Ab

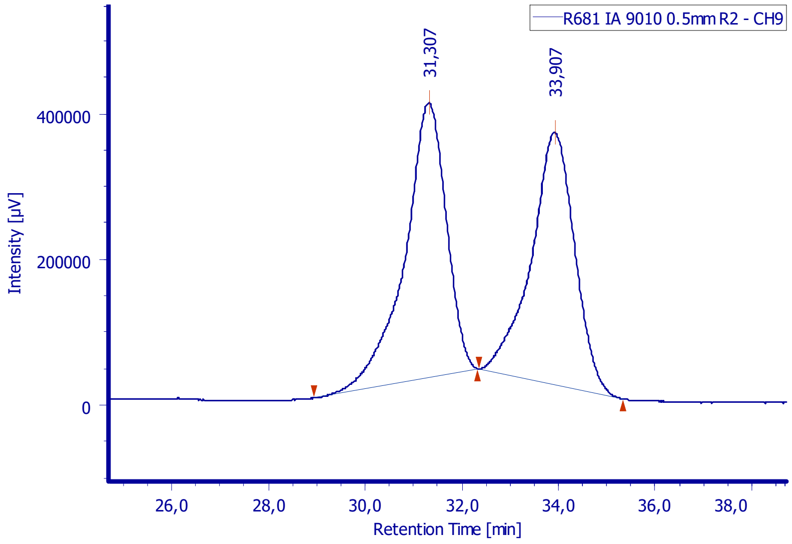

tR	Area	Area%
31,307	22401244	50,887
33,907	21620418	49,113

3Ab

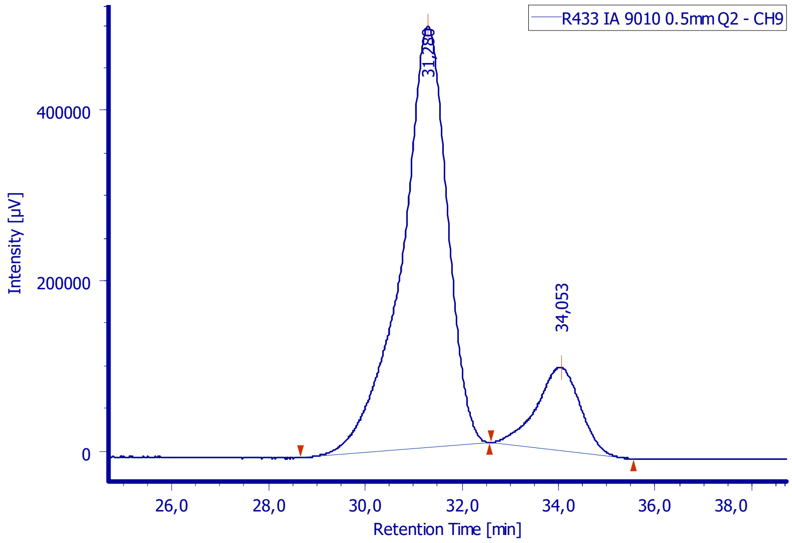

tR	Area	Area%
31,280	32354024	84,151
34,053	6093648	15,849

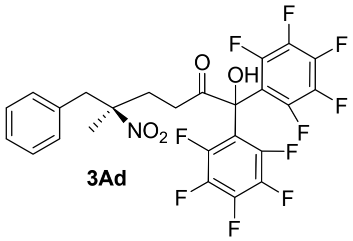

3Ad

The enantiomeric ratio was determined by HPLC analysis (Phenomenex Lux 3μm Cellulose 1, Hexane/^{i}PrOH = 95/5, flow rate = 1 mL/min, λ = 202 nm).

(±) 3Ad

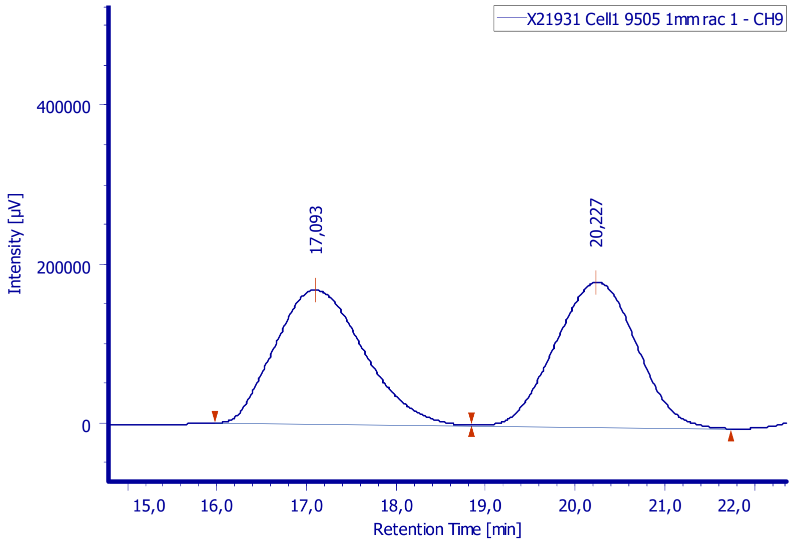

tR	Area	Area%
17.093	11610662	50.228
20.227	11505090	49.772

3Ad (reaction carried out at r.t.)

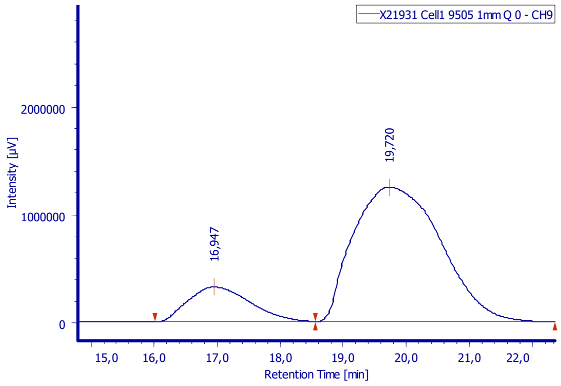

tR	Area	Area%
16.947	22076889	15.635
19.720	119127606	84.365

3Ad (reaction carried out at 0 °C)

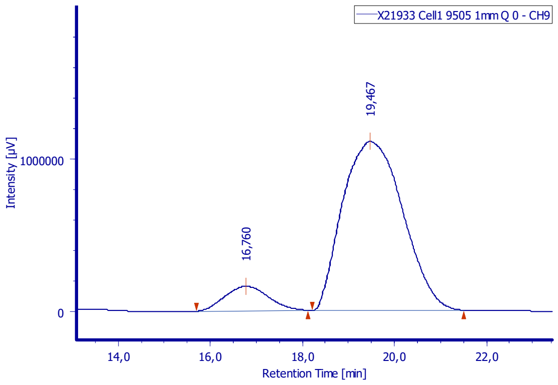

tR	Area	Area%
16,760	10446198	9,440
19,467	100213288	90,560

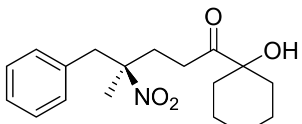

3Ae

The enantiomeric ratio was determined by HPLC analysis (Phenomenex Lux 3μm Cellulose 1, Hexane/^{i}PrOH = 98/2, flow rate = 1 mL/min, λ = 202 nm).

(±) 3Ae

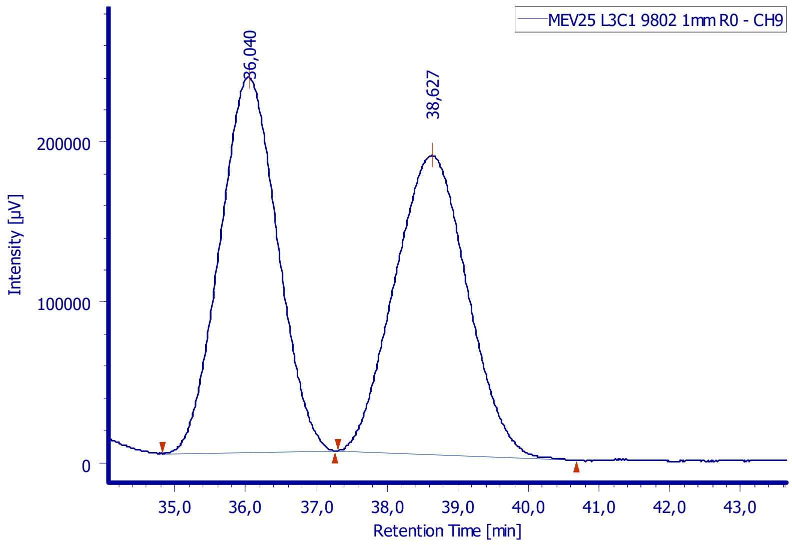

tR	Area	Area%
36.040	13216097	49.848
38.627	13296790	50.152

3Ae

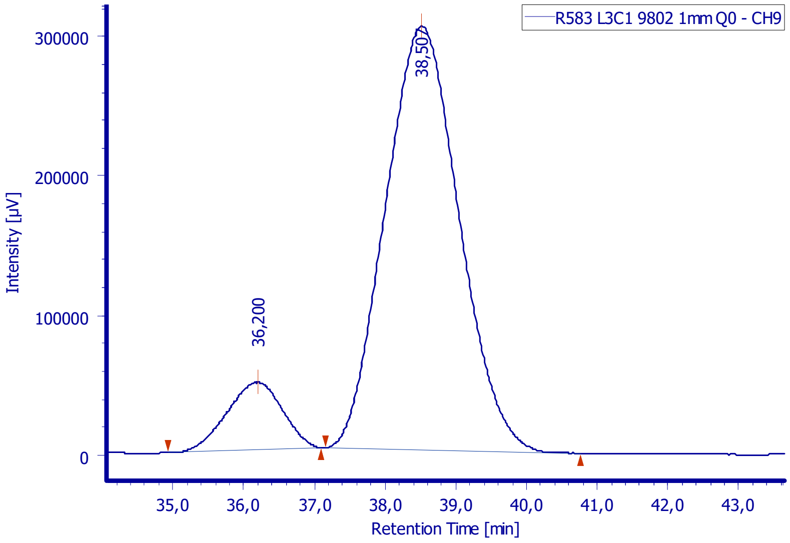

tR	Area	Area%
36.200	2633321	10.703
38.507	21970052	89.297

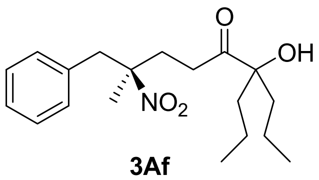

3Af

The enantiomeric ratio was determined by HPLC analysis (Daicel Chiralpak IA, Hexane/^{i}PrOH = 99/1, flow rate = 1 mL/min, λ = 202 nm).

(±) 3Af

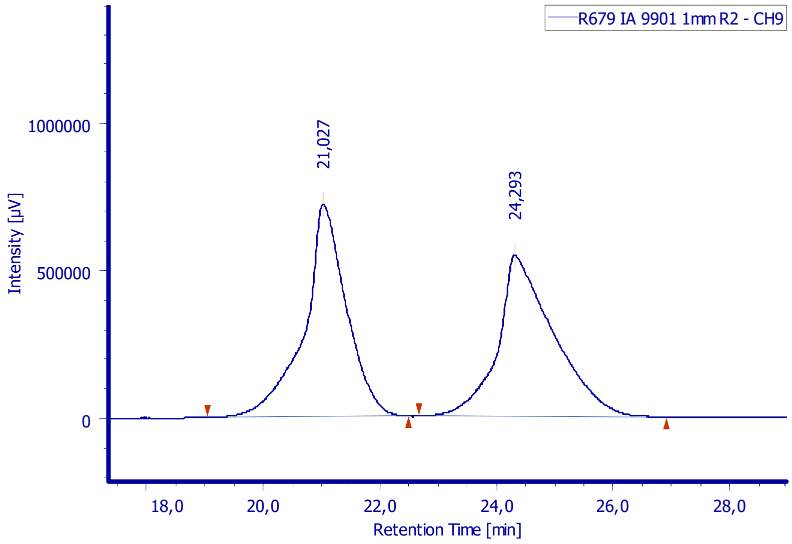

tR	Area	Area%
21,027	36140640	49,958
24,293	36201048	50,042

3Af

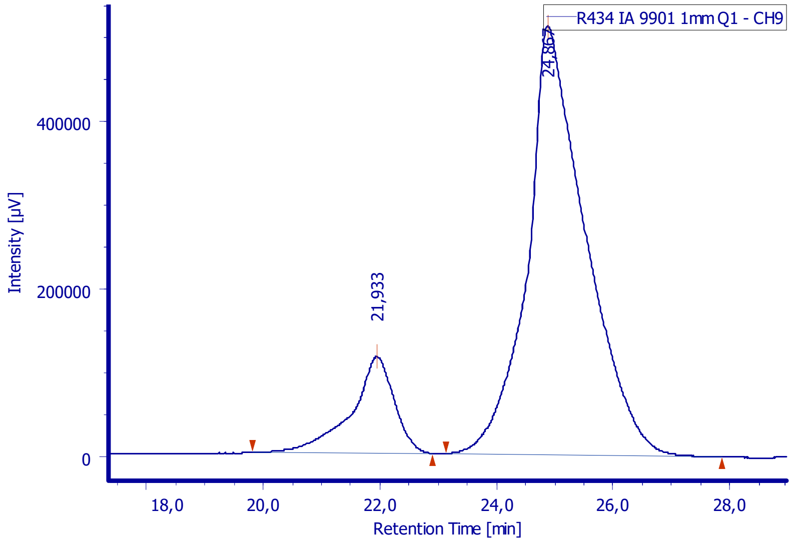

tR	Area	Area%
21,933	5902667	14,364
24,867	35189757	85,636

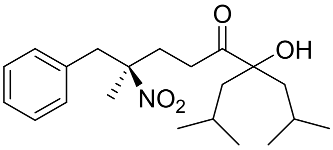

3Ag

The enantiomeric ratio was determined by HPLC analysis (Daicel Chiralpak IA, Hexane/^{i}PrOH = 99/1, flow rate = 1 mL/min, λ = 202 nm).

(±) 3Ag

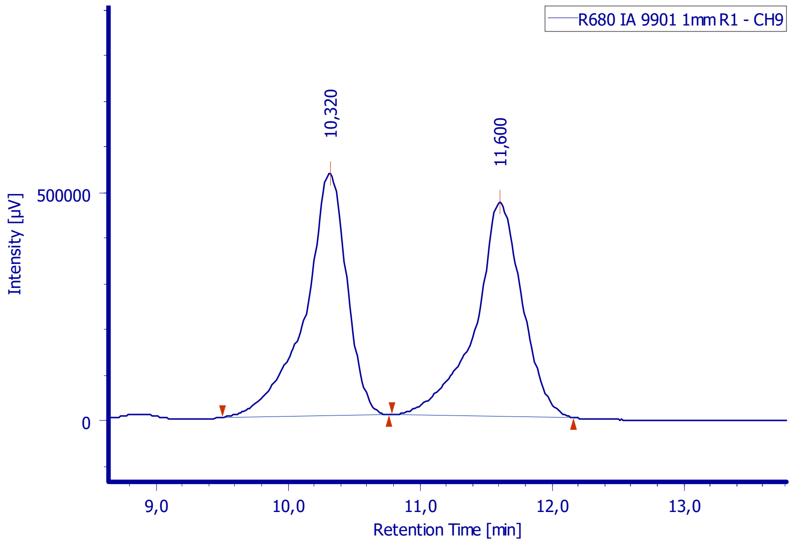

tR	Area	Area%
10,320	11612607	50,047
11,600	11590929	49,953

3Ag

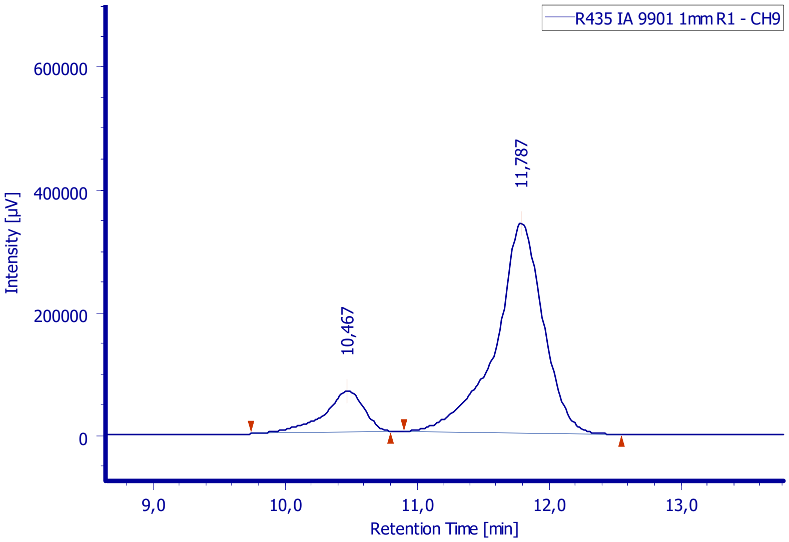

tR	Area	Area%
10,467	1415212	14,245
11,787	8519320	85,755

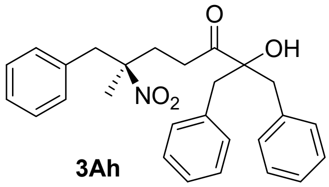

3Ah

The enantiomeric ratio was determined by HPLC analysis (Phenomenex Lux 3µm Cellulose 2, Hexane/^{i}PrOH = 95/5, flow rate = 1 mL/min, λ = 202 nm).

(±) 3Ah

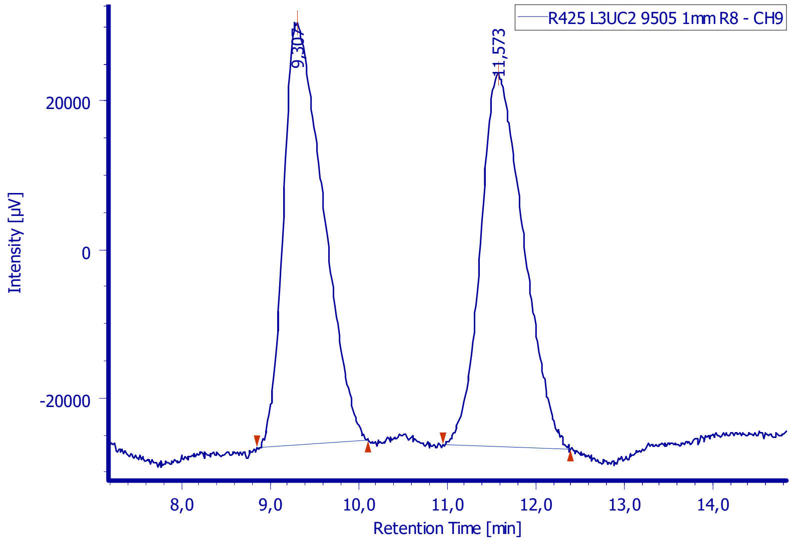

tR	Area	Area%
9.307	1757414	50.872
11.573	1697194	49.128

3Ah

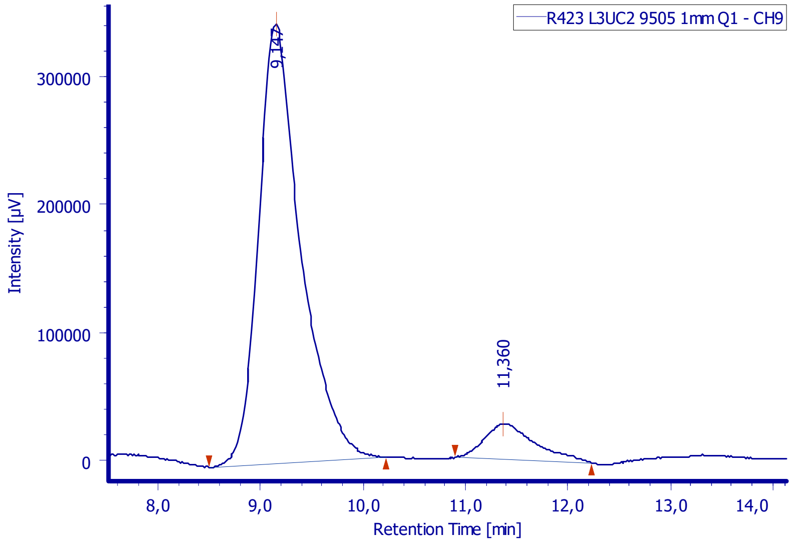

tR	Area	Area%
9.147	9832244	91.158
11.360	953712	8.842

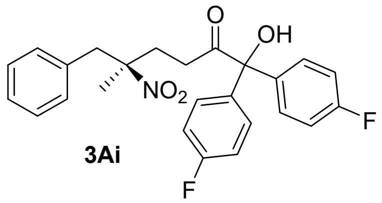

3Ai

The enantiomeric ratio was determined by HPLC analysis (Phenomenex Lux 3µm Cellulose 2, Hexane/^{i}PrOH = 98/2, flow rate = 1 mL/min, λ = 202 nm).

(±) 3Ai

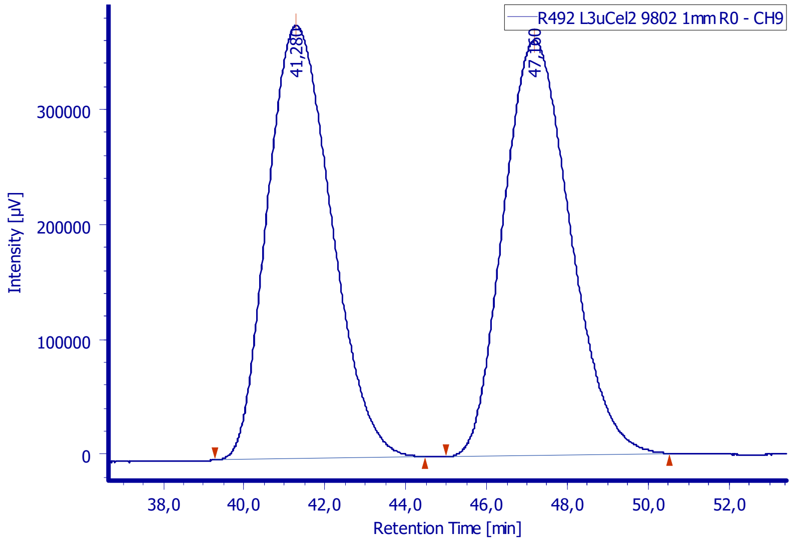

tR	Area	Area%
41.280	41287189	50.010
47.160	41270025	49.990

3Ai

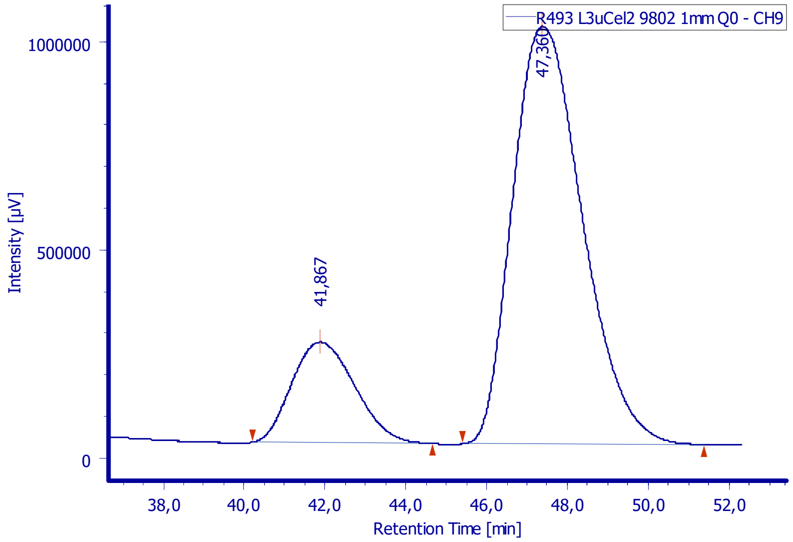

tR	Area	Area%
41.867	26125246	17.852
47.360	120218765	82.148

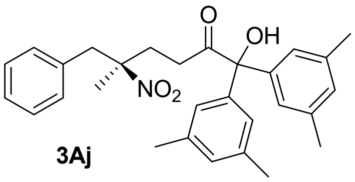

3Aj

The enantiomeric ratio was determined by HPLC analysis (Daicel Chiralpak IC-3, Hexane/^{i}PrOH = 98/2, flow rate = 1 mL/min, λ = 202 nm).

(±) 3Aj

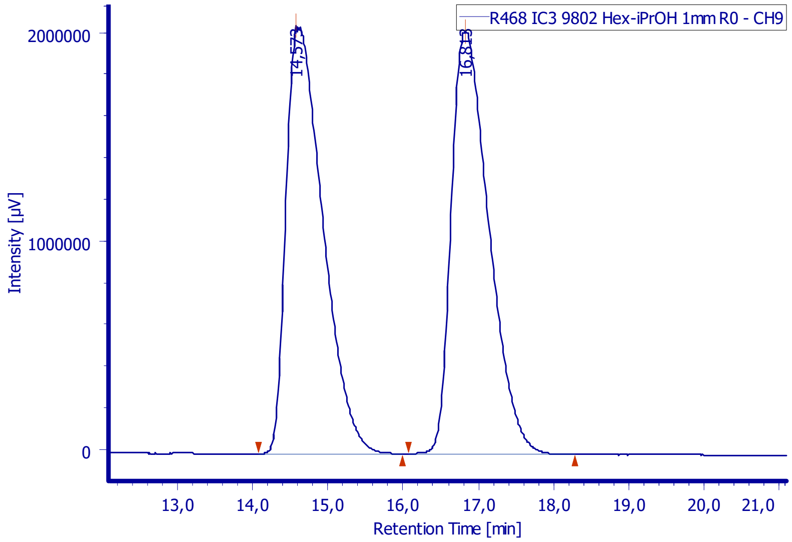

tR	Area	Area%
14.573	69298233	49.753
16.813	69986005	50.247

3Aj

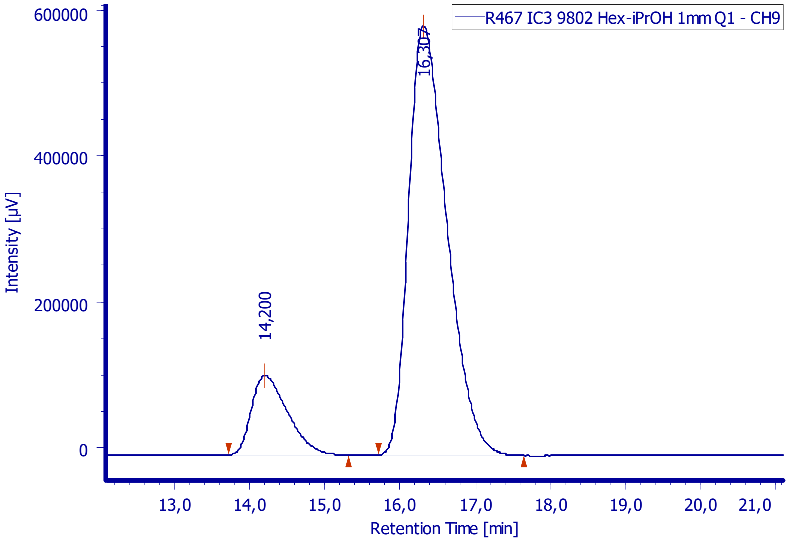

tR	Area	Area%
14.200	3627404	14.856
16.307	20789927	85.144

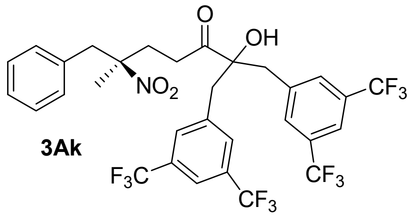

3Ak

The enantiomeric ratio was determined by HPLC analysis (Phenomenex Lux 3μm Cellulose 1, Hexane/EtOH = 99/1, flow rate = 1 mL/min, 0 ˚C, λ = 202 nm).

(±) 3Ak

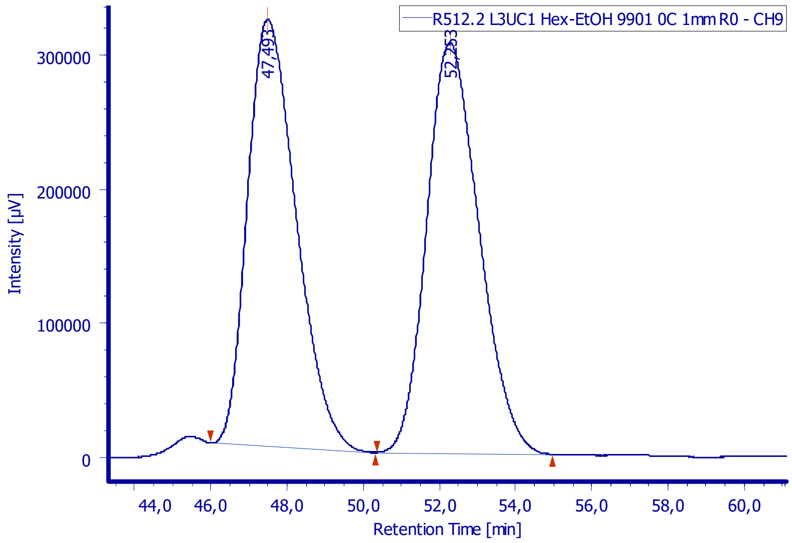

tR	Area	Area%
47.493	28120223	49.169
52.253	29070213	50.831

3Ak

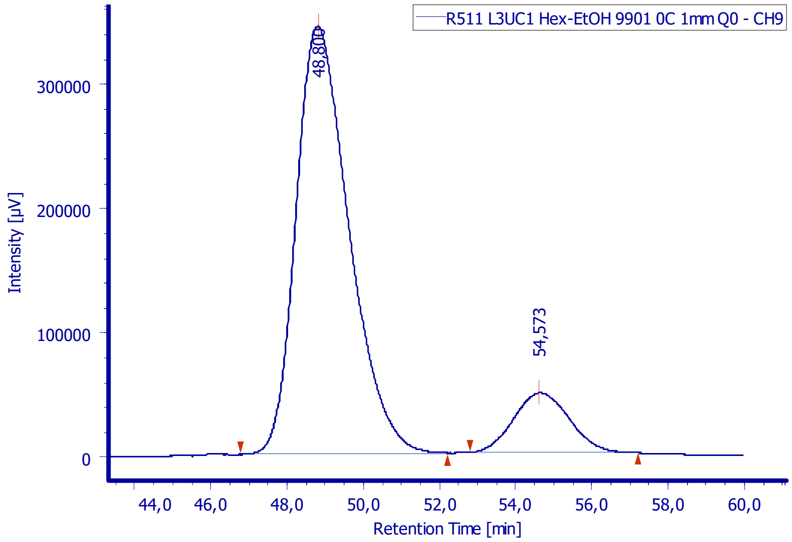

tR	Area	Area%
48.800	33069033	87.193
54.573	4857228	12.807

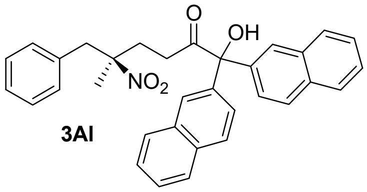

3Al

The enantiomeric ratio was determined by HPLC analysis (Daicel Chiralpak OD-H, Hexane/^{i}PrOH = 80/20, flow rate = 0.5 mL/min, λ = 202 nm).

(±) 3Al

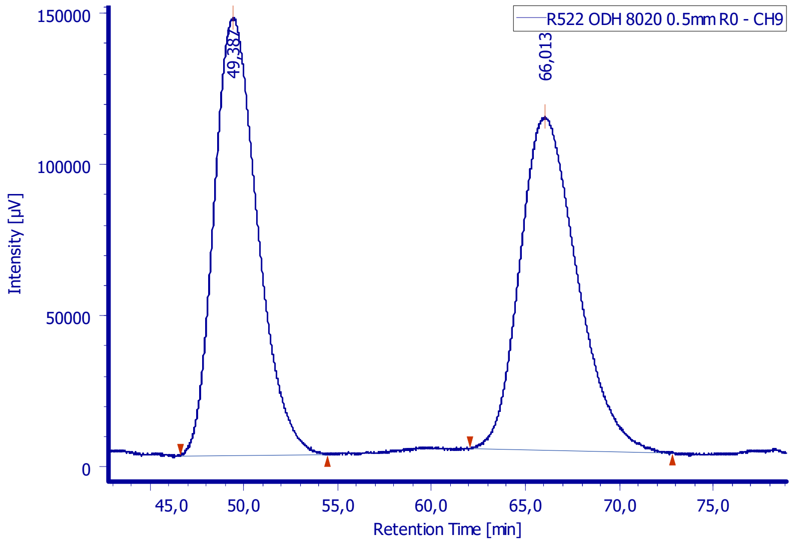

tR	Area	Area%
49.387	23133905	49.829
66.013	23293061	50.171

3Al

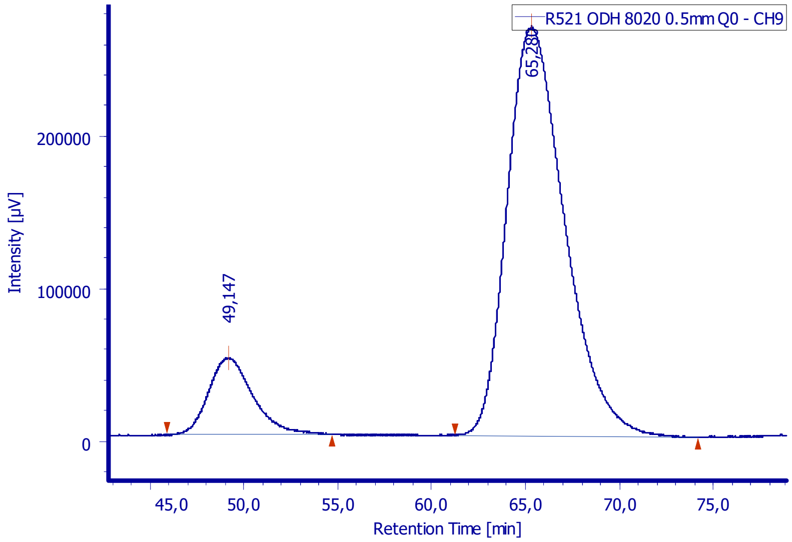

tR	Area	Area%
49.147	8276445	12.669
65.280	57054334	87.331

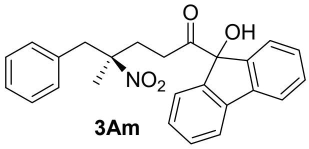

3Am

The enantiomeric ratio was determined by HPLC analysis (Daicel Chiralpak IA, Hexane/^{i}PrOH = 98/2, flow rate = 1 mL/min, λ = 202 nm).

(±) 3Am

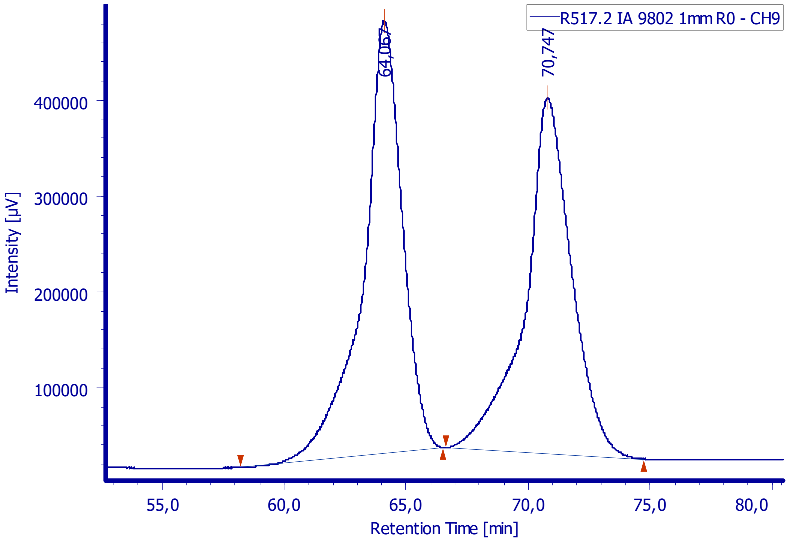

tR	Area	Area%
64.067	50242115	50.612
70.747	49027033	49.388

3Am

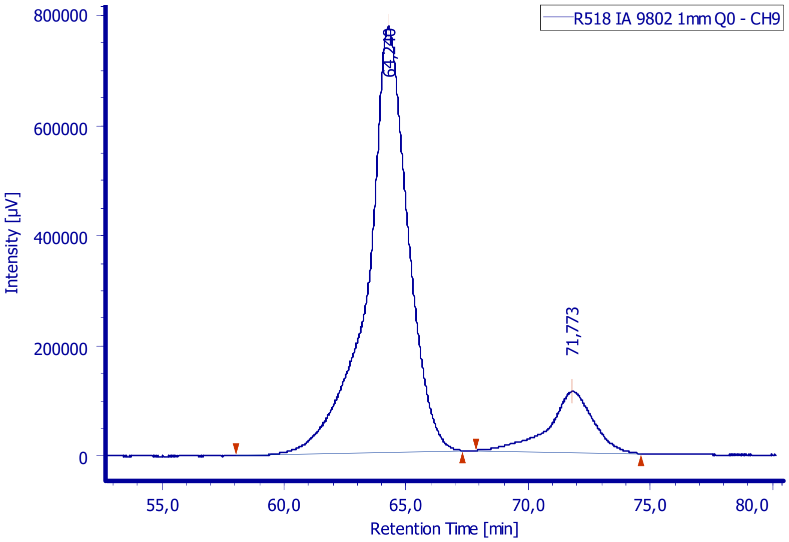

tR	Area	Area%
64.240	88896251	86.597
71.773	13759163	13.403

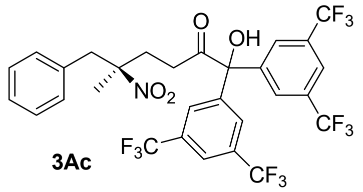

3Ac

The enantiomeric ratio was determined by HPLC analysis (Phenomenex Lux 3µm Cellulose 1, Hexane/EtOH = 98/2, flow rate = 1 mL/min, 0 ˚C, λ = 202 nm).

(±) 3Ac

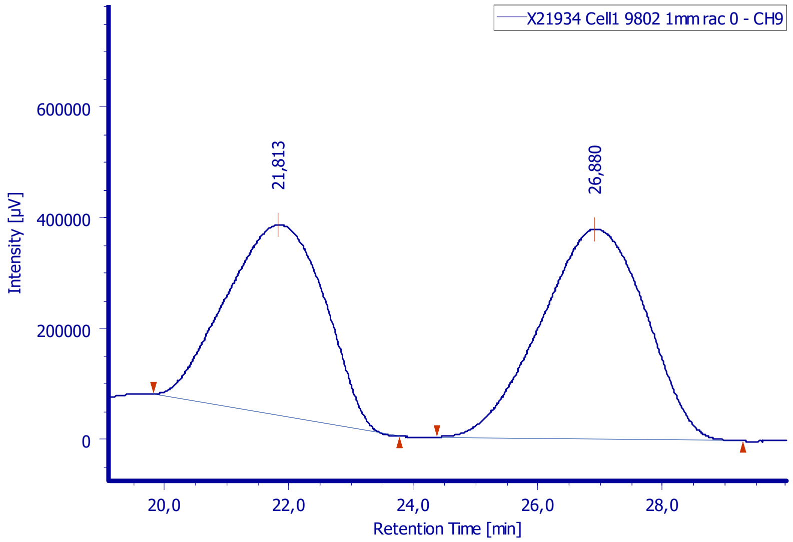

tR	Area	Area%
21.813	37746803	46.069
26.880	44188628	53.931

3Ac

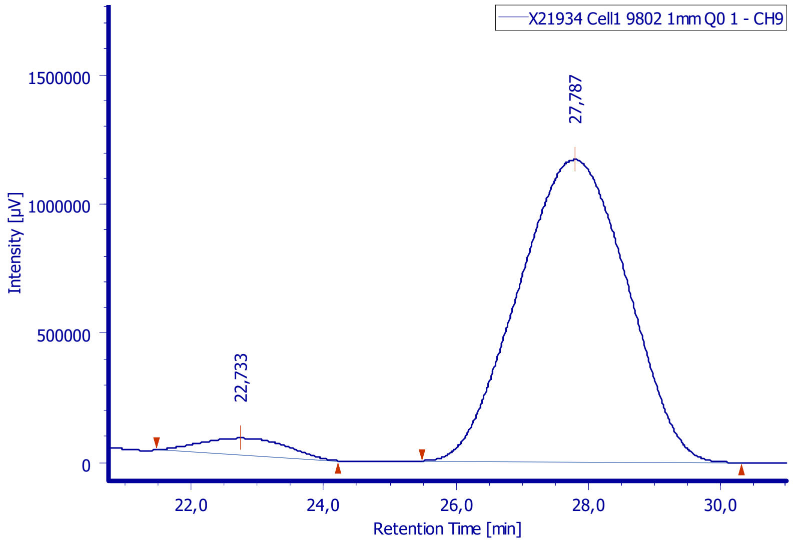

tR	Area	Area%
22.733	5904537	4.269
27.787	132418710	95.731

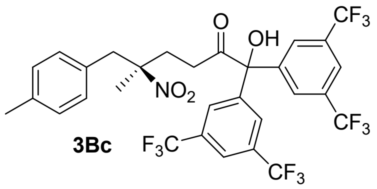

3Bc

The enantiomeric ratio was determined by HPLC analysis (Phenomenex Lux 3μm Cellulose 1, Hexane/^{i}PrOH = 95/5, flow rate = 1 mL/min, λ = 202 nm).

(±) 3Bc

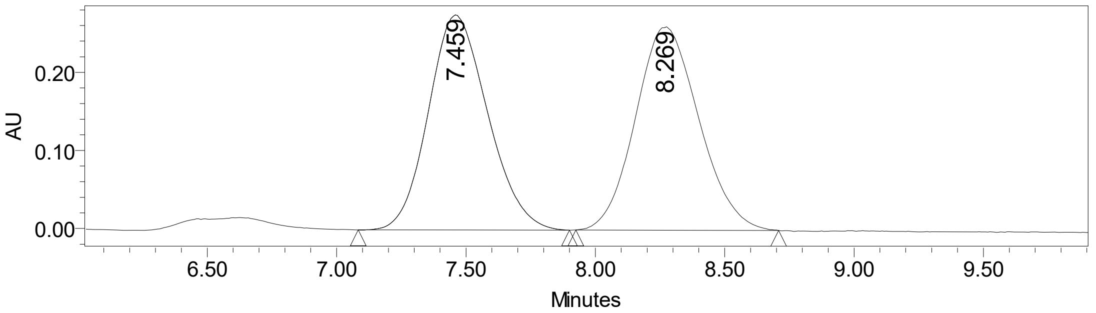

Retention Time	Area	% Area
7.459	4391021	49.68
8.269	4447660	50.32

3Bc

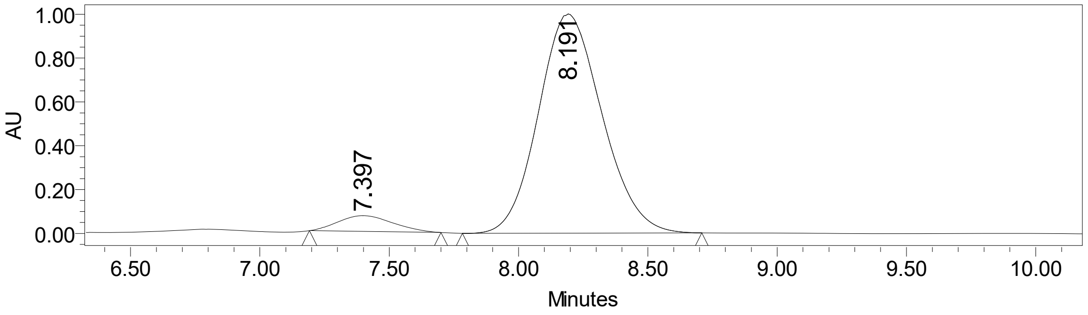

Retention Time	Area	% Area
7.397	1049923	5.79
8.191	17095826	94.21

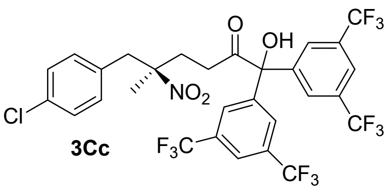

3Cc

The enantiomeric ratio was determined by HPLC analysis (Daicel Chiralpak IB, Hexane/^{i}PrOH = 99:1, flow rate = 1 mL/min, λ = 202 nm).

(±) 3Cc

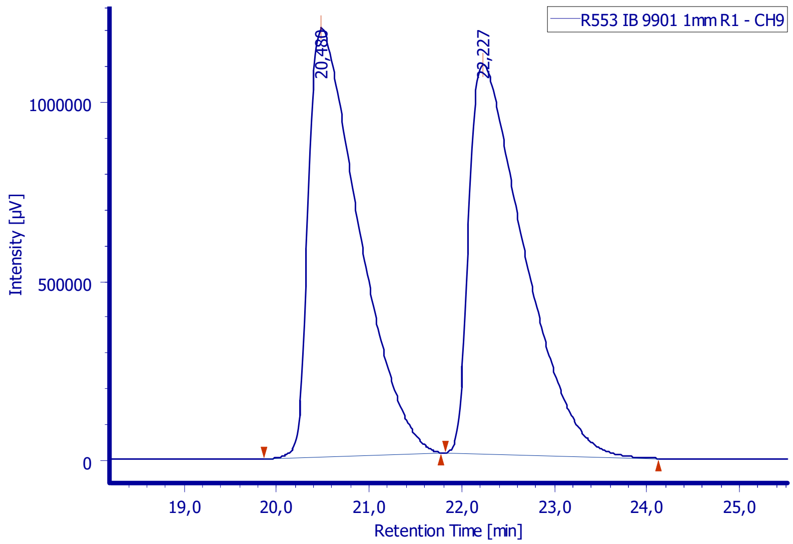

tR	Area	Area%
20,480	45067926	50,170
22,227	44763137	49,830

3Cc

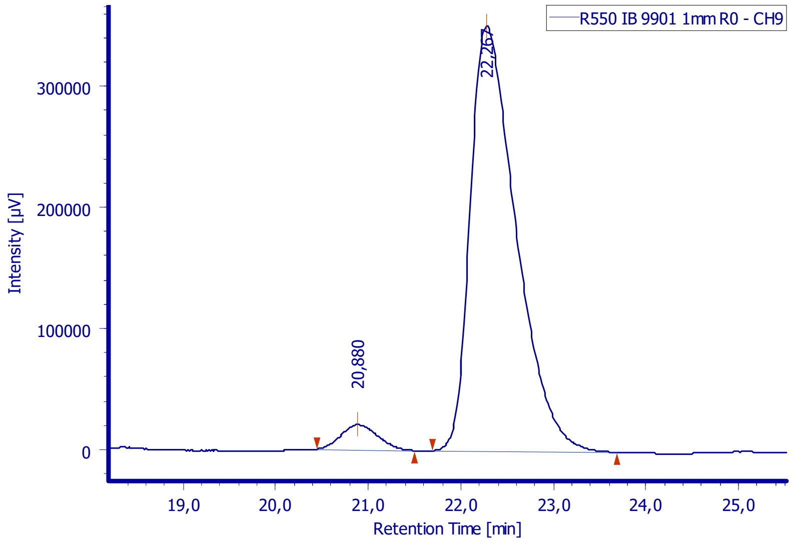

tR	Area	Area%
20,880	539150	4,290
22,267	12028467	95,710

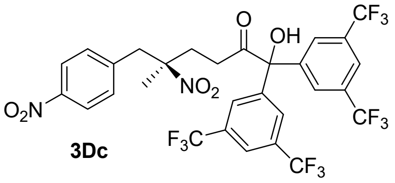

3Dc

The enantiomeric ratio was determined by HPLC analysis (Phenomenex Lux 3µm Cellulose 1, Hexane/[i]PrOH = 95/5, flow rate = 1 mL/min, λ = 202 nm).

(±) 3Dc

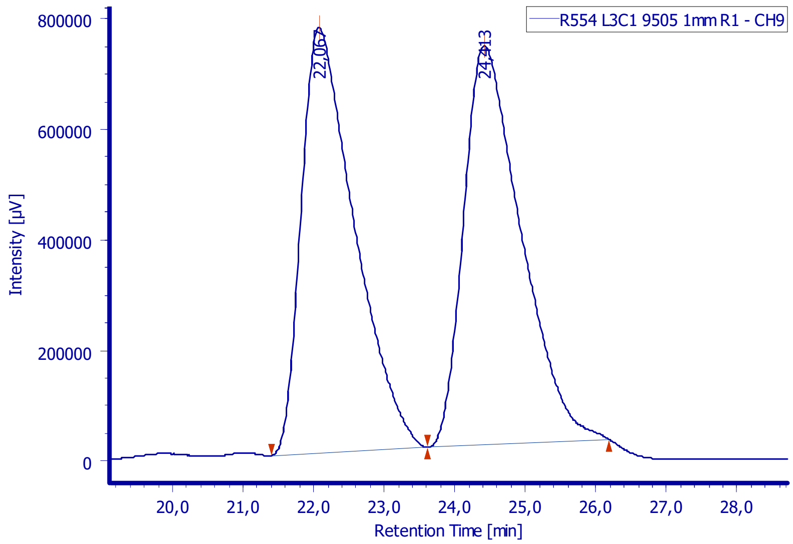

tR	Area	Area%
22,067	40147444	49,724
24,413	40593208	50,276

3Dc

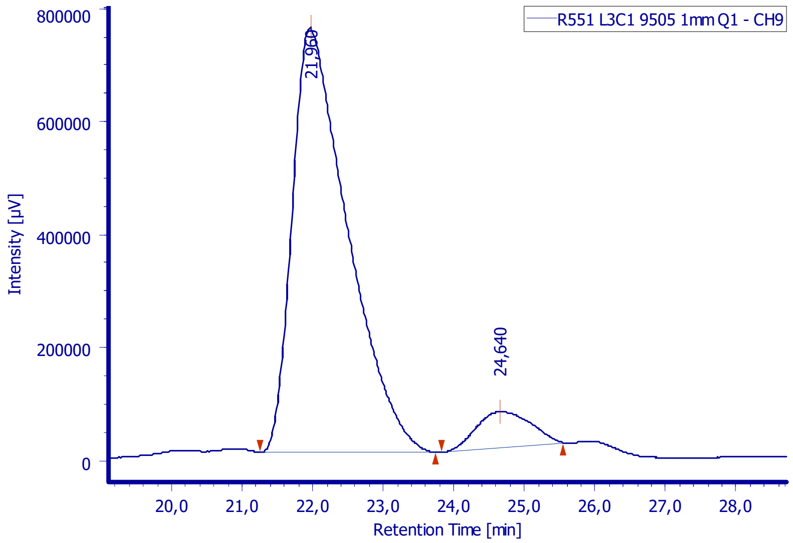

tR	Area	Area%
21,960	40841584	92,318
24,640	3398648	7,682

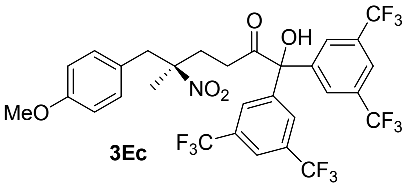

3Ec

The enantiomeric ratio was determined by HPLC analysis (Daicel Chiralpak IB, Hexane/^{i}PrOH = 98/2, flow rate = 1 mL/min, λ = 202 nm).

(±) 3Ec

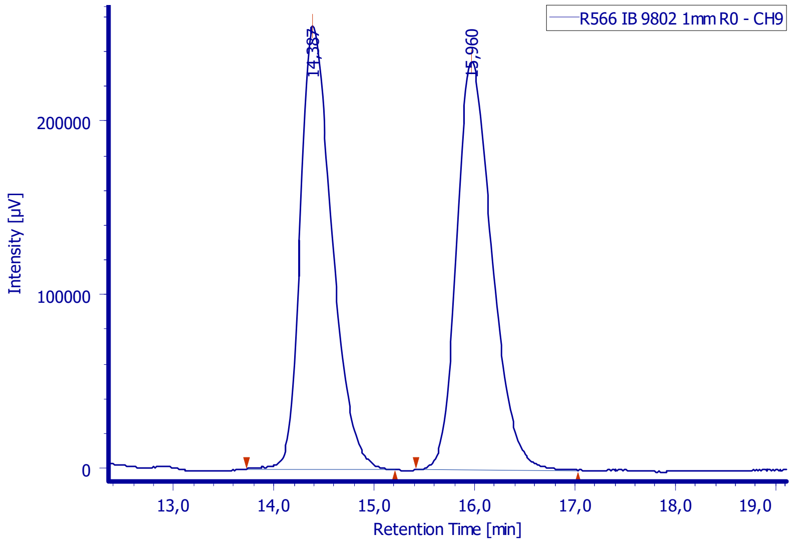

tR	Area	Area%
14,387	5555898	49,124
15,960	5753980	50,876

3Ec

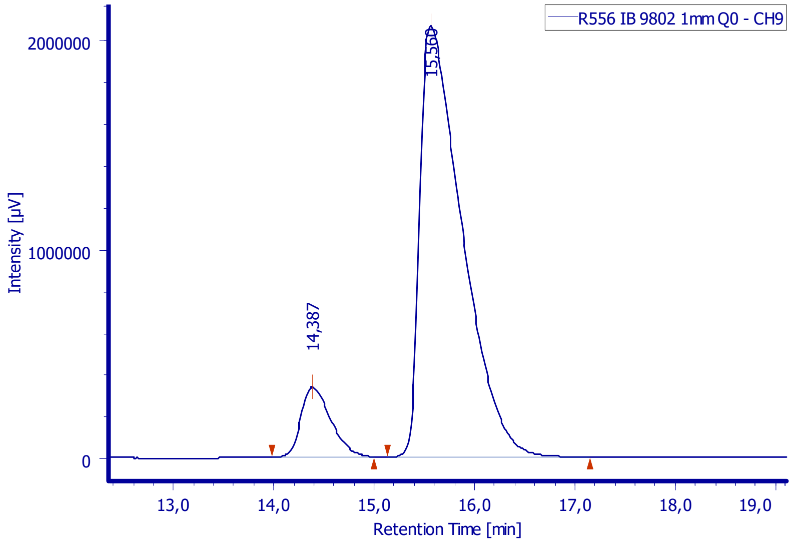

tR	Area	Area%
14,387	6865656	10,246
15,560	60139241	89,754

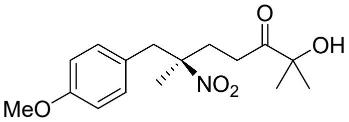

3Ea

The enantiomeric ratio was determined by HPLC analysis (Daicel Chiralpak IA, Hexane/^{i}PrOH = 95/5, flow rate = 0.5 mL/min, λ = 202 nm).

(±) 3Ea

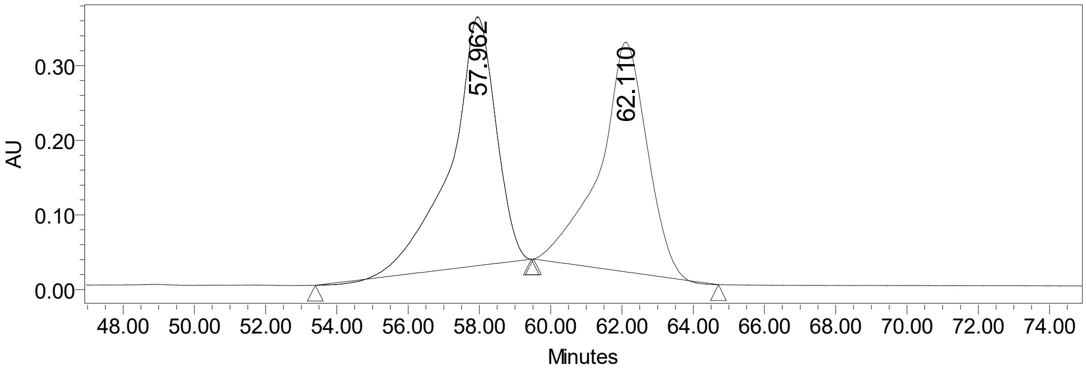

Retention Time	Area	% Area
57.962	29826311	50.86
62.110	28821394	49.14

3Ea

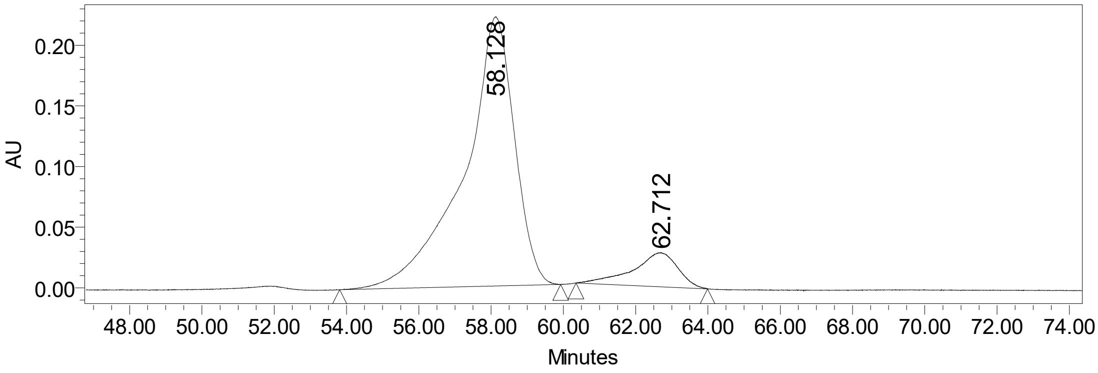

Retention Time	Area	% Area
58.128	21529133	89.86
62.712	2429597	10.14

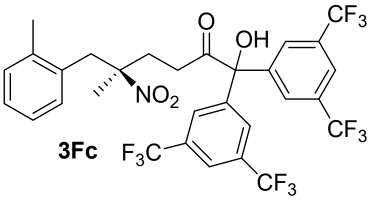

3Fc

The enantiomeric ratio was determined by HPLC analysis (Daicel Chiralpak IB, Hexane/^{i}PrOH = 98/2, flow rate = 1 mL/min, λ = 202 nm).

(±) 3Fc

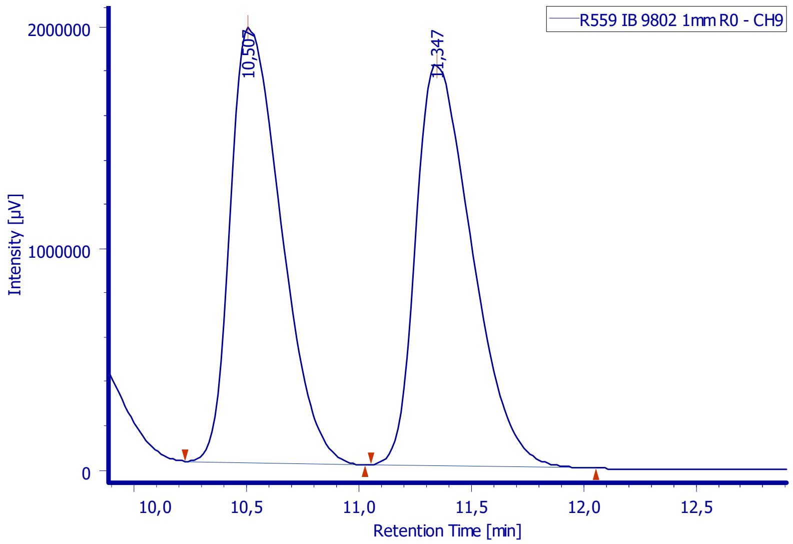

tR	Area	Area%
10,507	30853140	49,457
11,347	31530340	50,543

3Fc

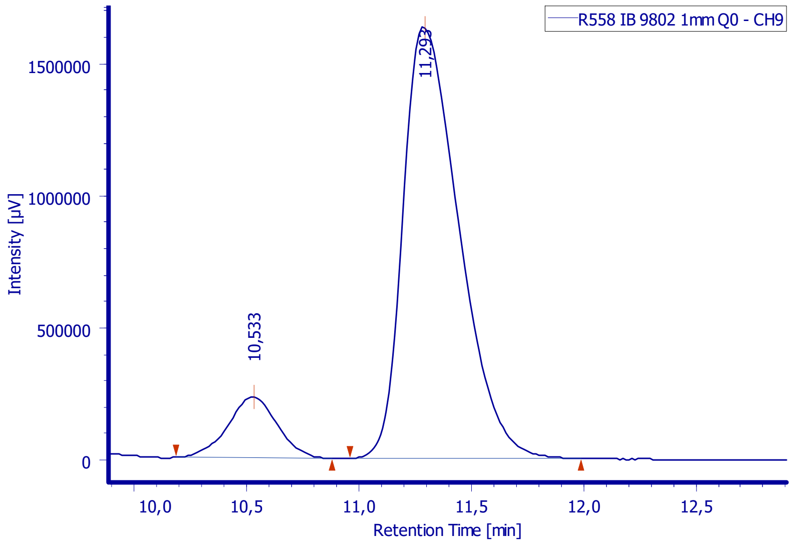

tR	Area	Area%
10,533	3534325	11,133
11,293	28212266	88,867

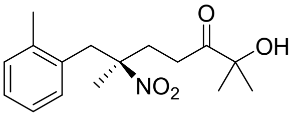

3Fa

The enantiomeric ratio was determined by HPLC analysis (Daicel Chiralpak IA, Hexane/^{i}PrOH = 95/5, flow rate = 1 mL/min, λ = 202 nm).

(±) 3Fa

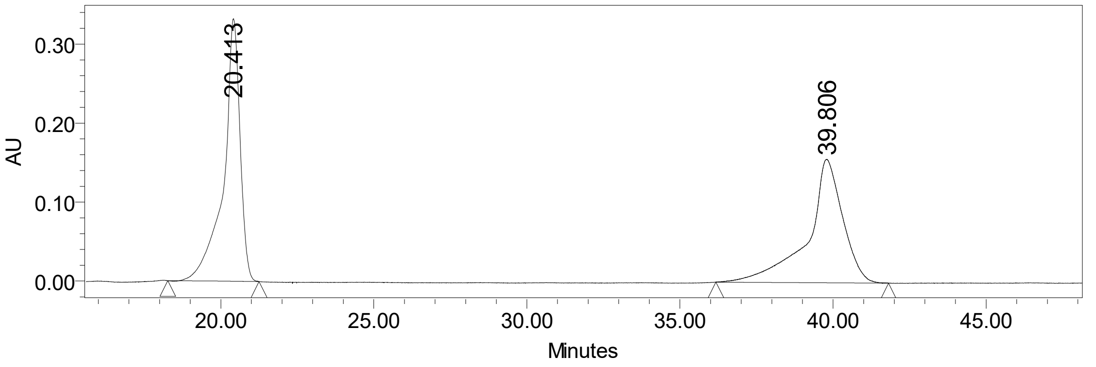

Retention Time	Area	% Area
20.413	12422054	49.90
39.806	12473065	50.10

3Fa

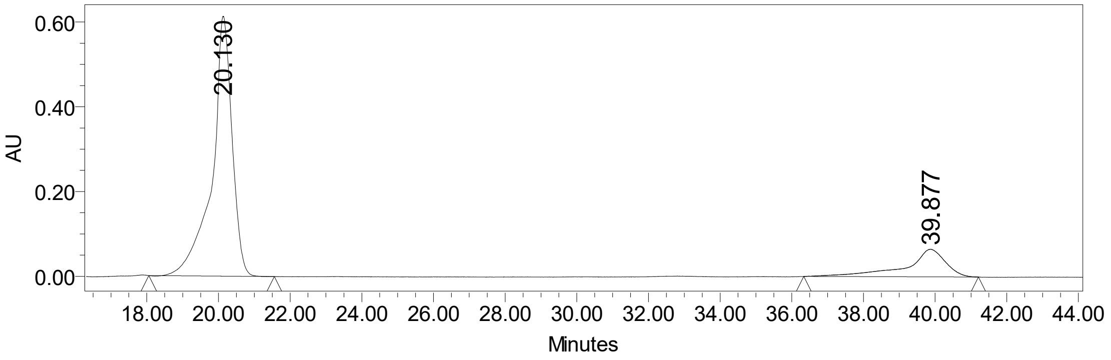

Retention Time	Area	% Area
20.130	23680158	82.68
39.877	4960636	17.32

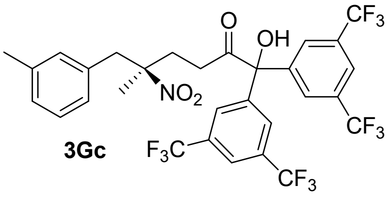

3Gc

The enantiomeric ratio was determined by HPLC analysis (Daicel Chiralpak IB, Hexane/^{i}PrOH = 98/2, flow rate = 1 mL/min, λ = 202 nm).

(±) 3Gc

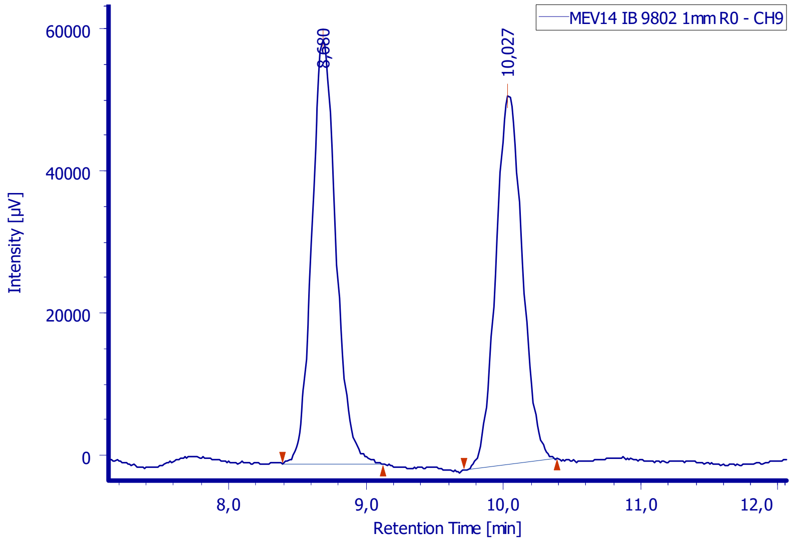

tR	Area	Area%
8,680	727942	50,943
10,027	700989	49,057

3Gc

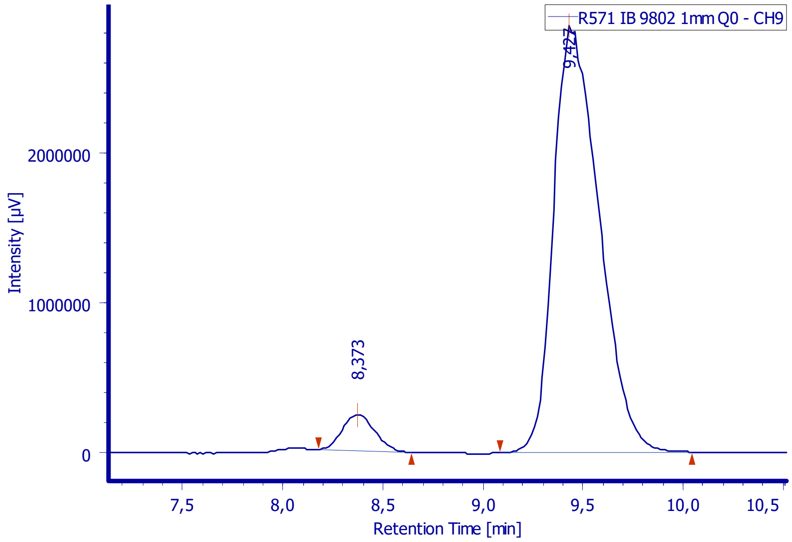

tR	Area	Area%
8,373	2738639	5,751
9,427	44883854	94,249

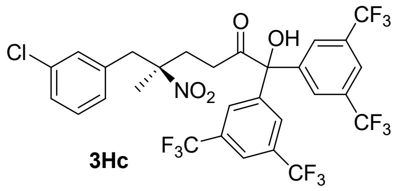

3Hc

The enantiomeric ratio was determined by HPLC analysis (Phenomenex Lux 3µm Cellulose 1, Hexane/^{i}PrOH = 98/2, flow rate = 1 mL/min, λ = 202 nm).

(±) 3Hc

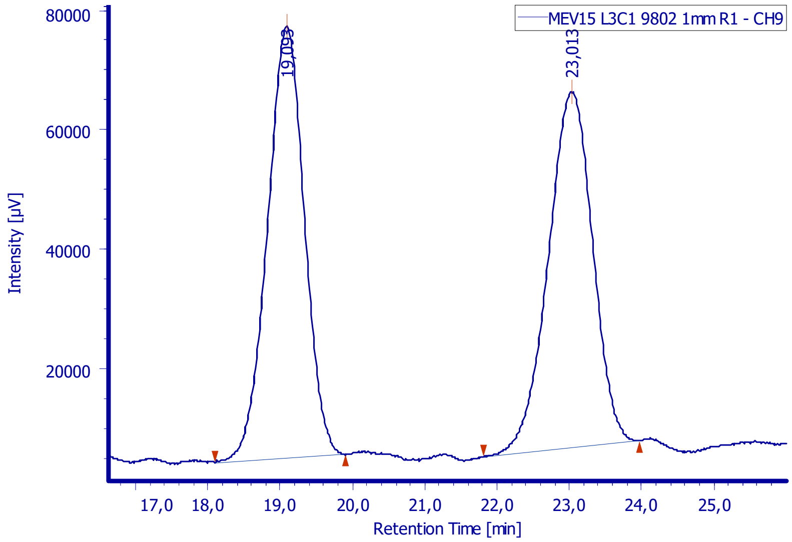

tR	Area	Area%
19,093	2555227	50,221
23,013	2532776	49,779

3Hc

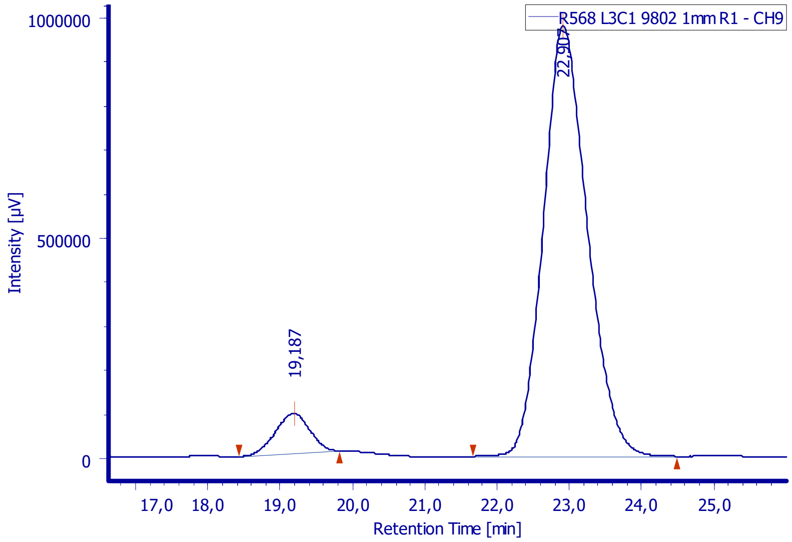

tR	Area	Area%
19,187	3025012	6,857
22,907	41088329	93,143

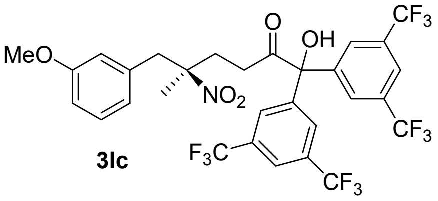

3lc

The enantiomeric ratio was determined by HPLC analysis (Daicel Chiralpak IB, Hexane/^{i}PrOH = 98/2, flow rate = 1 mL/min, λ = 202 nm).

(±) 3lc

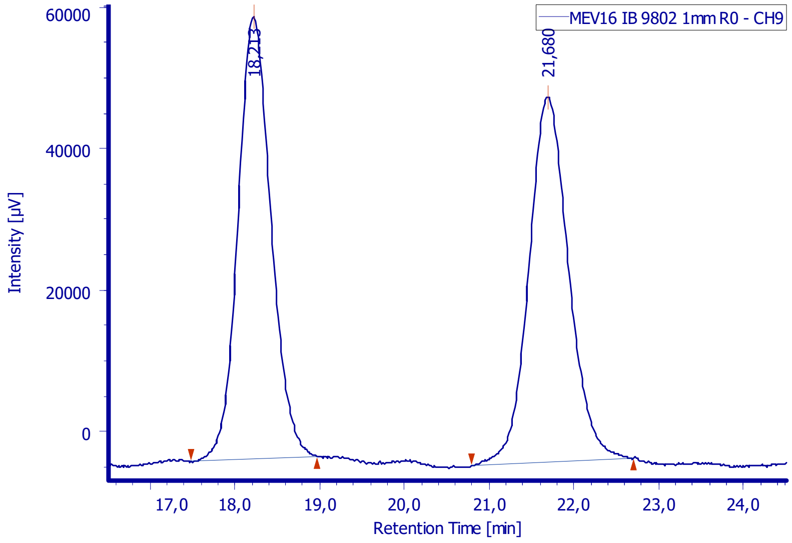

tR	Area	Area%
18,213	1686489	49,952
21,680	1689713	50,048

3lc

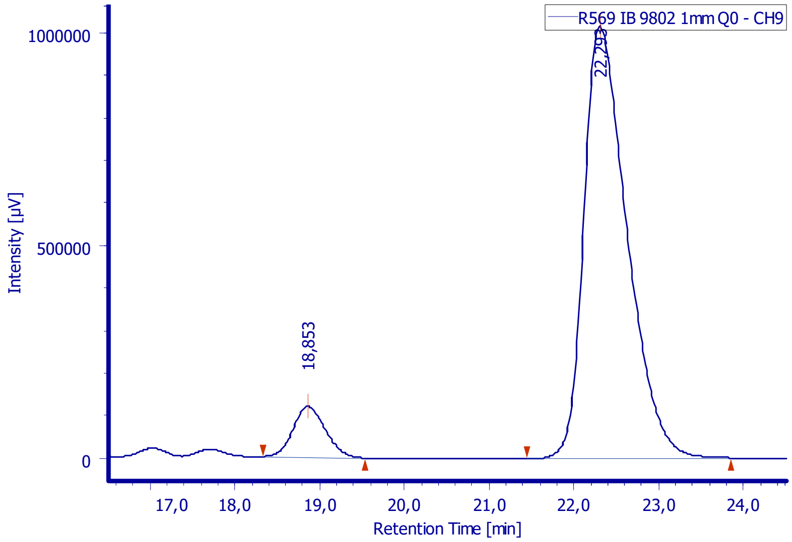

tR	Area	Area%
18,853	3133294	8,058
22,293	35748796	91,942

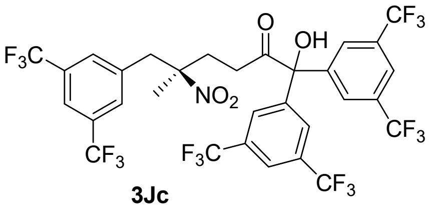

3Jc

The enantiomeric ratio was determined by HPLC analysis (Phenomenex Lux 3µm Cellulose 1, Hexane/^{i}PrOH = 98/2, flow rate = 1 mL/min, λ = 202 nm).

(±) 3Jc

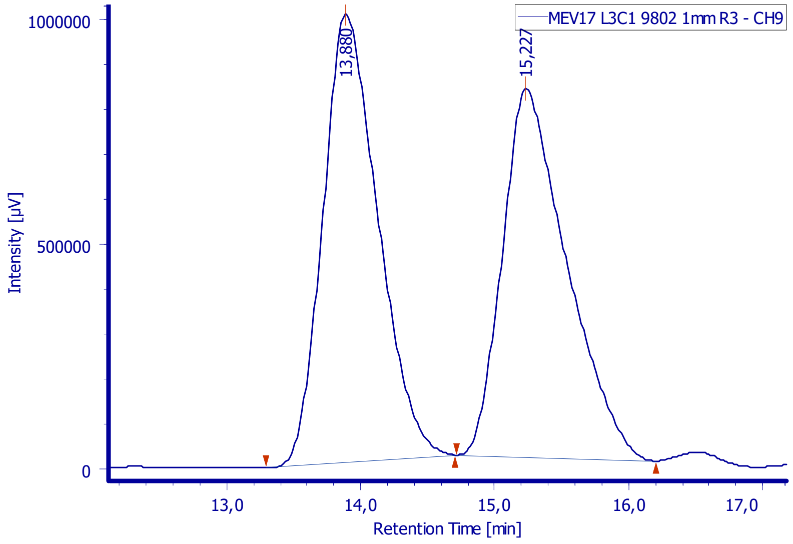

tR	Area	Area%
13,880	28397978	50,993
15,227	27292495	49,007

3Jc

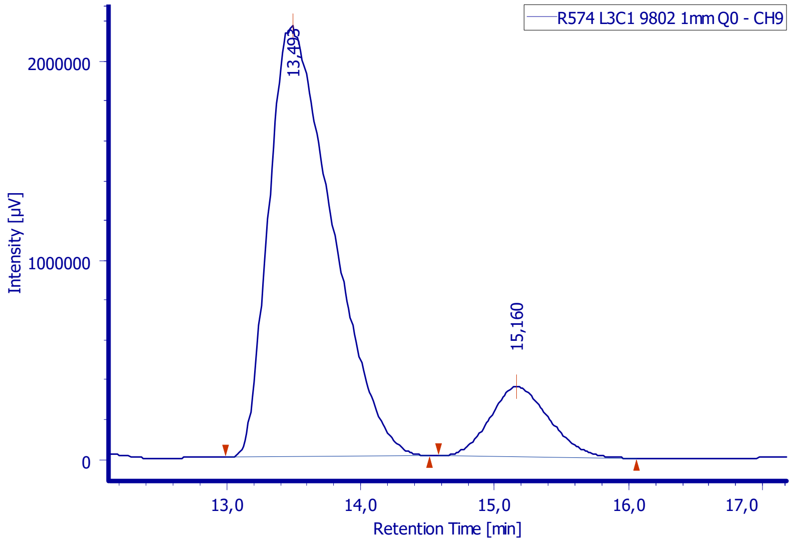

tR	Area	Area%
13,493	70607425	86,685
15,160	10845384	13,315

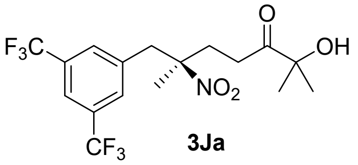

3Ja

The enantiomeric ratio was determined by HPLC analysis (Phenomenex Lux 3μm Cellulose 2, Hexane/^{i}PrOH = 95/5, flow rate=1 mL/min, λ = 202 nm).

(±) 3Ja

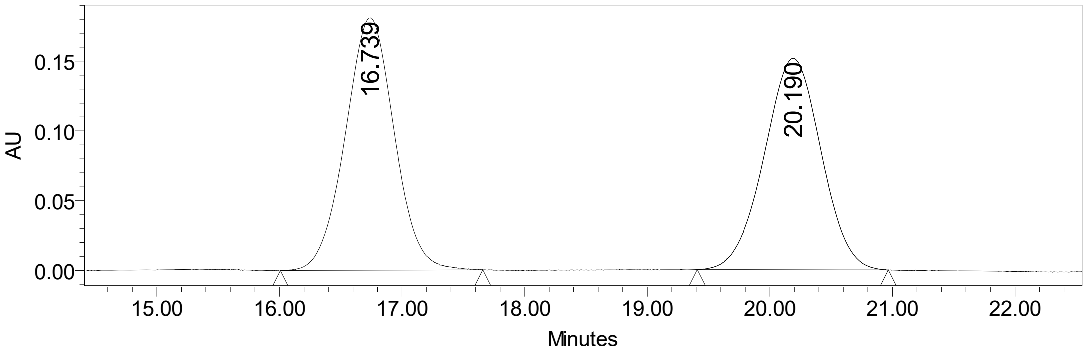

Retention Time	Area	% Area
16.739	5022489	50.67
20.190	4889554	49.33

3Ja

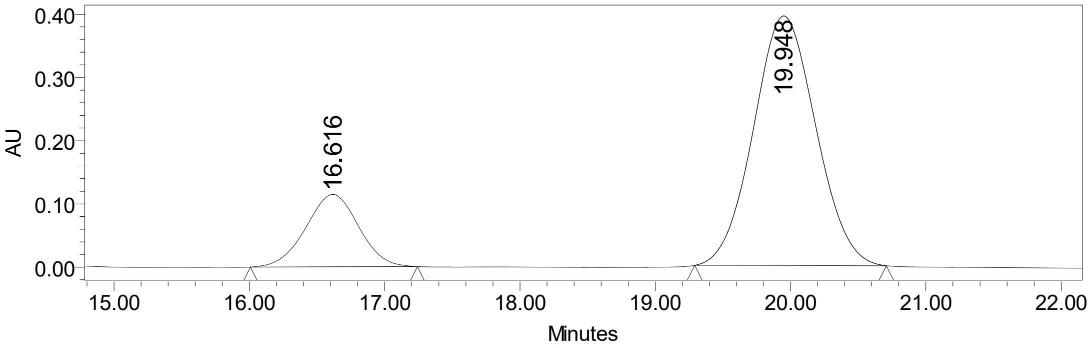

Retention Time	Area	% Area
16.616	3095059	19.68
19.948	12635006	80.32

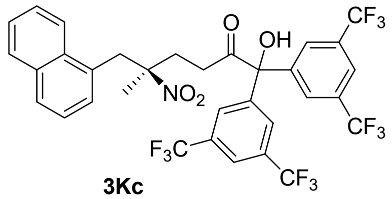

3Kc

The enantiomeric ratio was determined by HPLC analysis (Phenomenex Lux 3µm Cellulose 1, Hexane/^{i}PrOH = 95/5, flow rate = 1 mL/min, λ = 202 nm).

(±) 3Kc

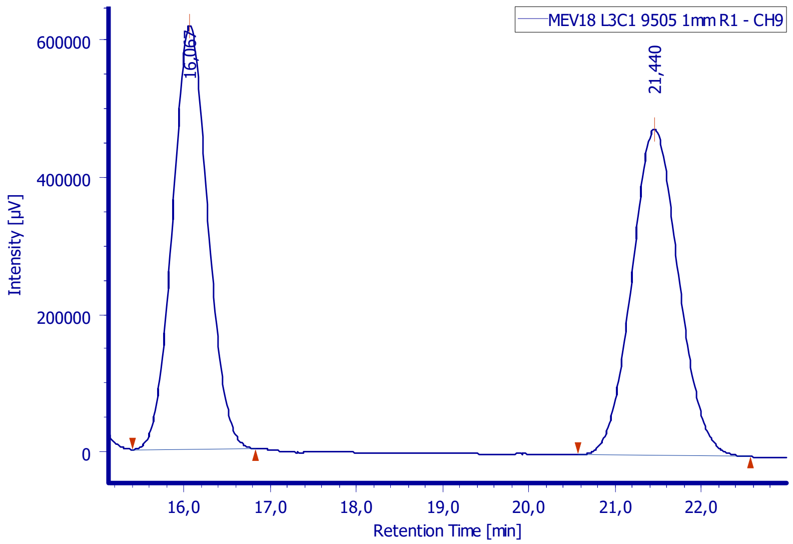

tR	Area	Area%
16,067	17428339	49,308
21,440	17917266	50,692

3Kc

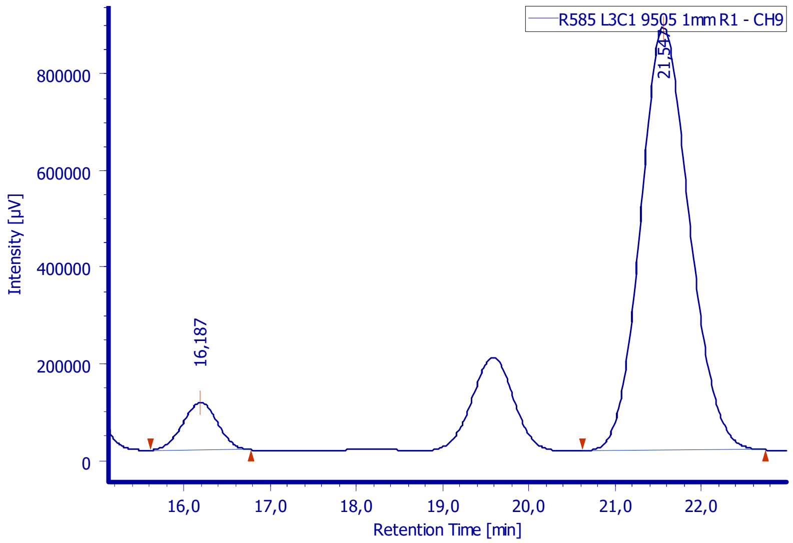

tR	Area	Area%
16,187	2714194	7,330
21,547	34314383	92,670

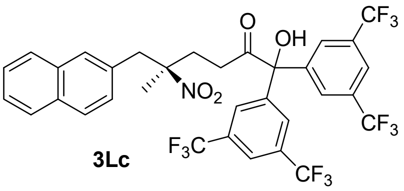

3Lc

The enantiomeric ratio was determined by HPLC analysis (Phenomenex Lux 3µm Cellulose 1, Hexane/^{i}PrOH = 98/2, flow rate = 1 mL/min, λ = 202 nm).

(±) 3Lc

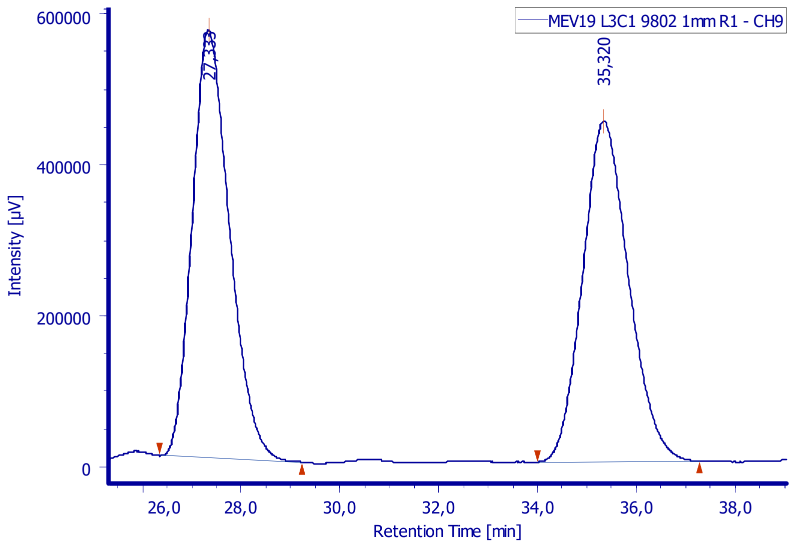

tR	Area	Area%
27,333	29701240	50,511
35,320	29100150	49,489

3Lc

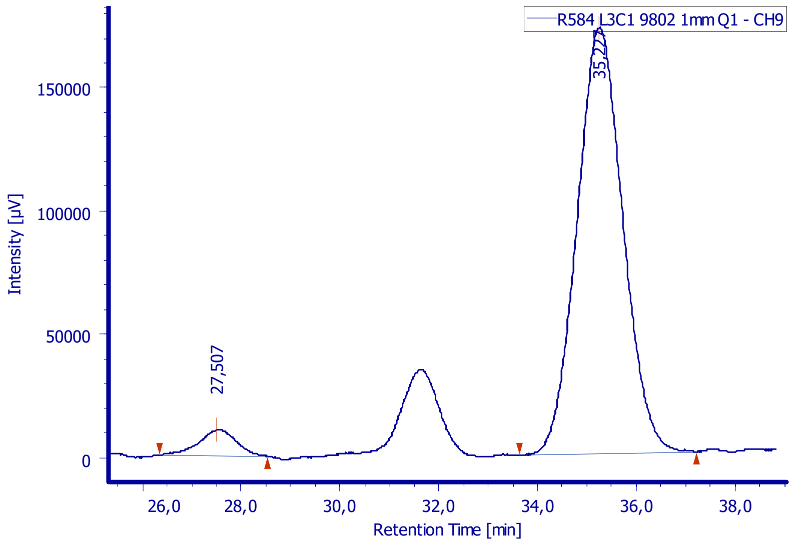

tR	Area	Area%
27,507	549389	4,731
35,227	11063124	95,269

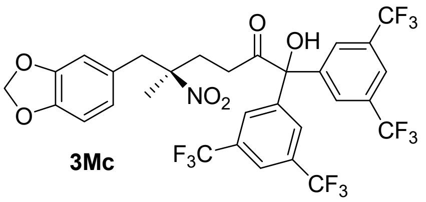

The enantiomeric ratio was determined by HPLC analysis (Phenomenex Lux 3µm Cellulose 1, Hexane/^{i}PrOH = 98/2, flow rate = 1 mL/min, λ = 202 nm).

(±) 3Mc

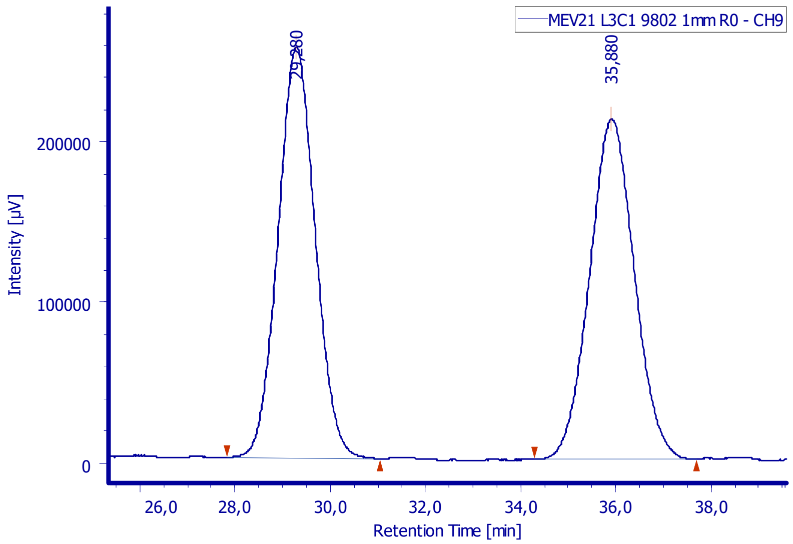

tR	Area	Area%
29,280	13873676	50,163
35,880	13783401	49,837

3Mc

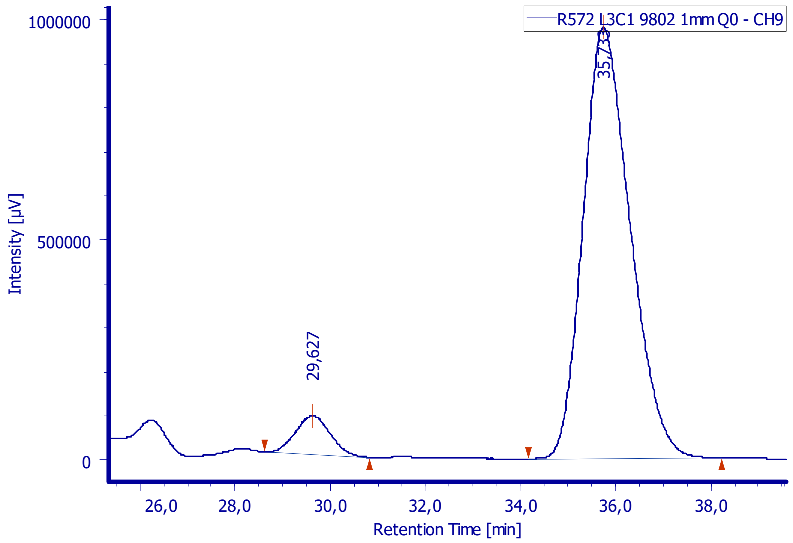

tR	Area	Area%
29,627	4387753	6,151
35,733	66941461	93,849

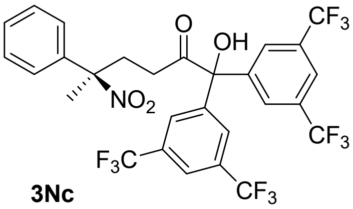

3Nc

The enantiomeric ratio was determined by HPLC analysis (Phenomenex Lux 3µm Cellulose 1, Hexane/^{i}PrOH = 98/2, flow rate = 1 mL/min, λ = 202 nm).

(±) 3Nc

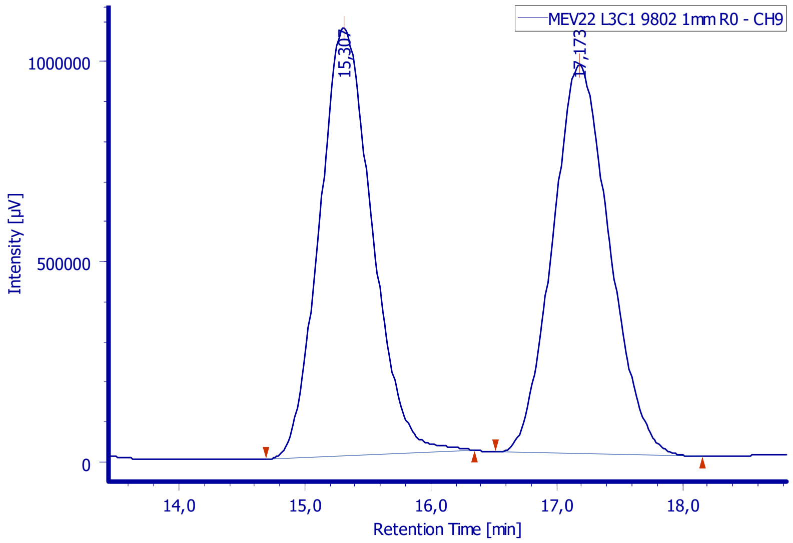

tR	Area	Area%
15,307	31051565	50,387
17,173	30575055	49,613

3Nc

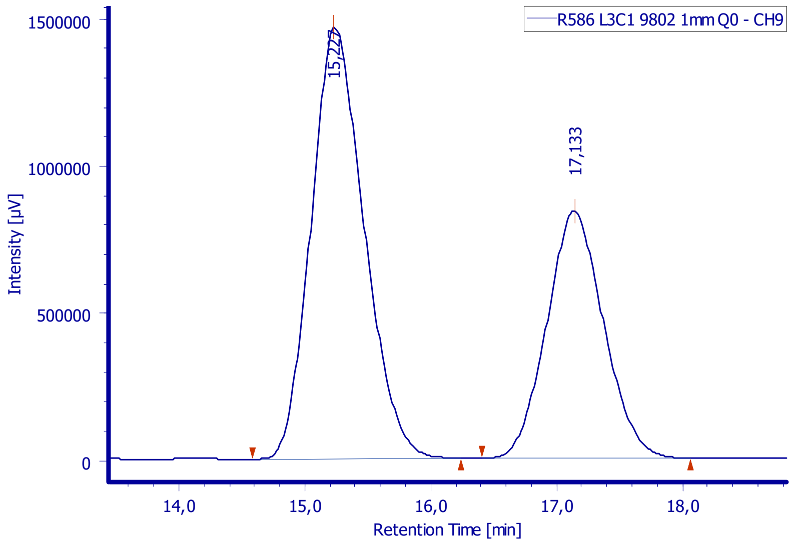

tR	Area	Area%
15,227	42738833	61,926
17,133	26277278	38,074

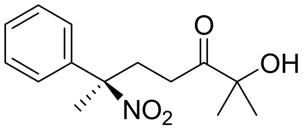

3Na

The enantiomeric ratio was determined by HPLC analysis (Daicel Chiralpak IA, Hexane/^{i}PrOH = 90/10, flow rate = 1 mL/min, λ = 202 nm).

(±) 3Na

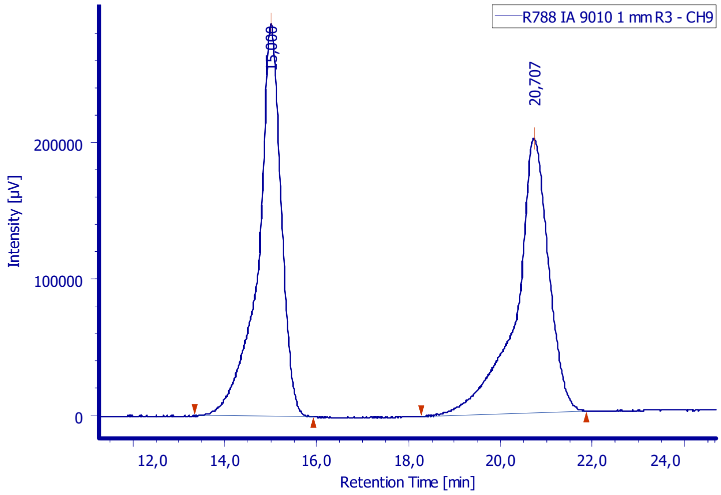

tR	Area	Area%
15,000	10026245	50,106
20,707	9983757	49,894

3Na

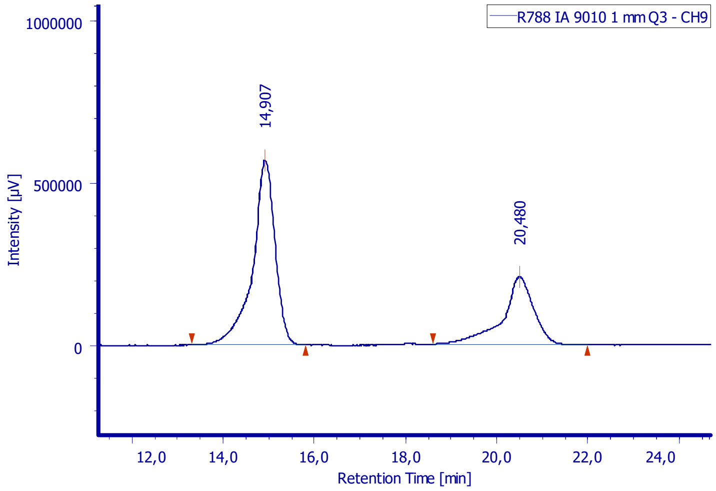

tR	Area	Area%
14,907	19285784	66,133
20,480	9876473	33,867

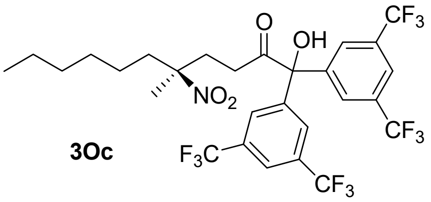

3Oc

The enantiomeric ratio was determined by HPLC analysis (Phenomenex Lux 3µm Cellulose 1, Hexane/iPrOH = 98/2, flow rate = 1 mL/min, λ = 202 nm).

(±) 3Oc

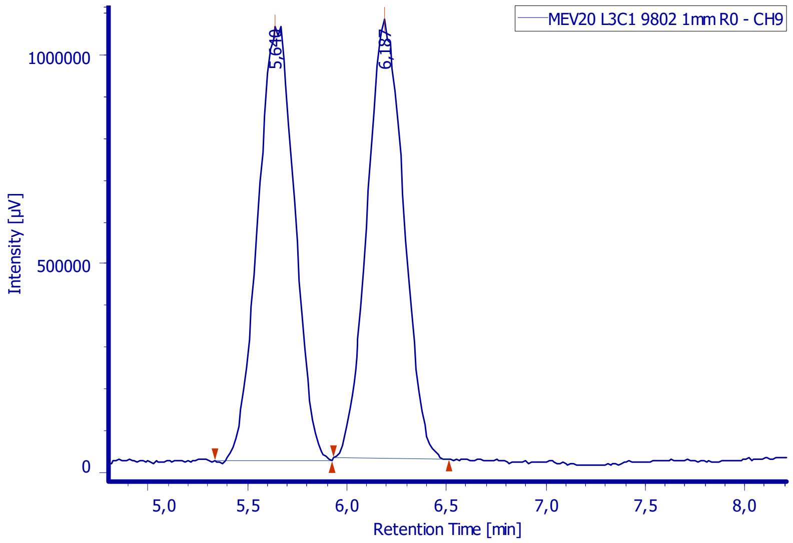

tR	Area	Area%
5,640	13413769	49,906
6,187	13464170	50,094

3Oc

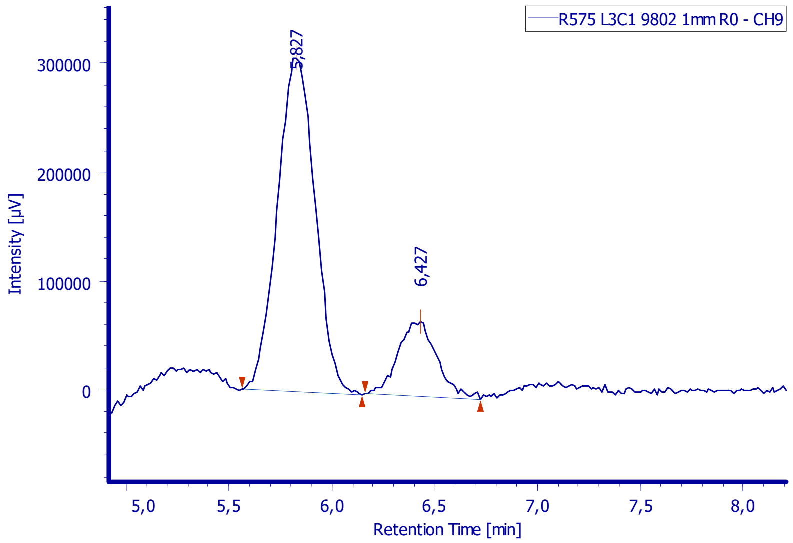

tR	Area	Area%
5,827	3906302	81,307
6,427	898055	18,693

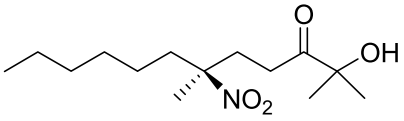

3Oa

The enantiomeric ratio was determined by HPLC analysis (Phenomenex Lux 3μm Cellulose 1, Hexane/^{i}PrOH = 98/2, flow rate = 1 mL/min, λ = 202 nm).

(±) 3Oa

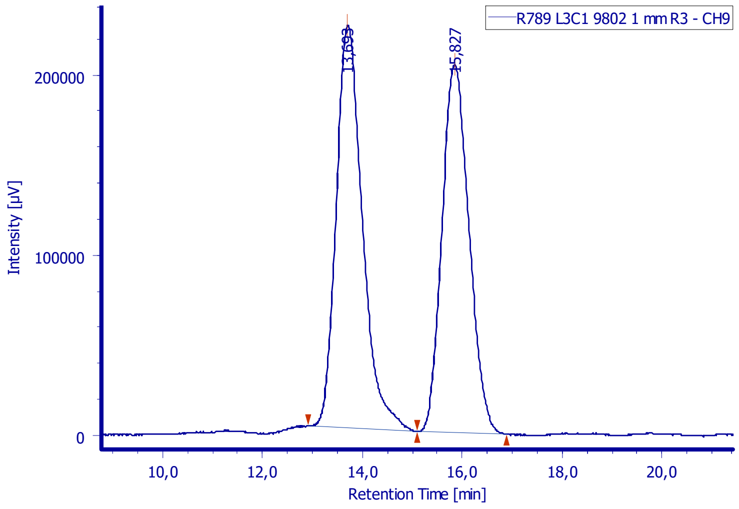

tR	Area	Area%
13,693	8151833	51,615
15,827	7641734	48,385

3Oa

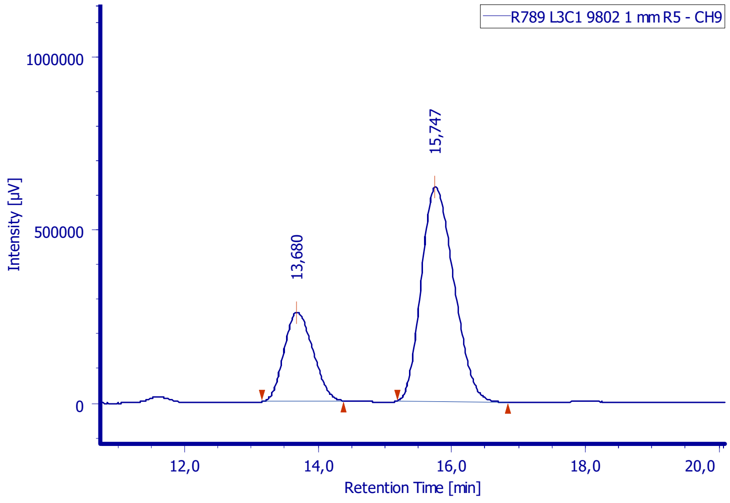

tR	Area	Area%
13,680	7687018	26,748
15,747	21051534	73,252

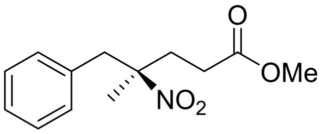

6A

The enantiomeric ratio was determined by HPLC analysis (Phenomenex Lux 3µm Cellulose 1, Hexane/iPrOH = 95/5, flow rate = 1 mL/min, λ = 202 nm).

(±) 6A

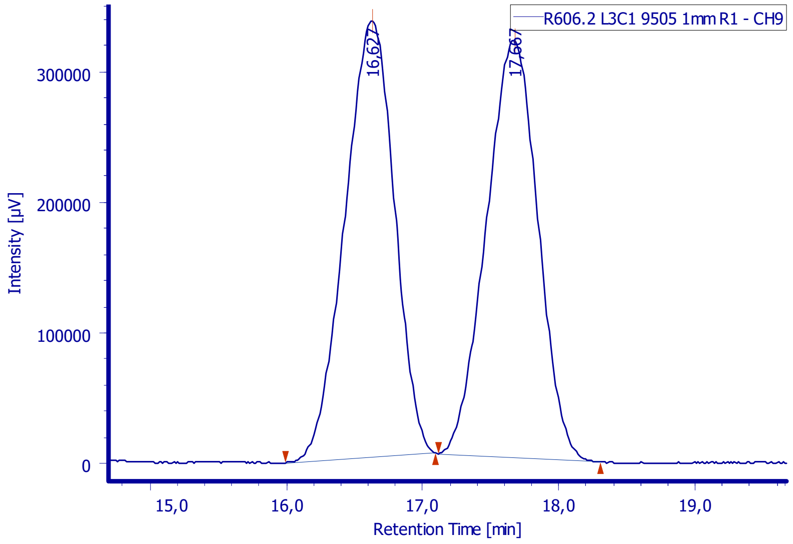

tR	Area	Area%
16,627	8407347	50,046
17,667	8391733	49,954

6A

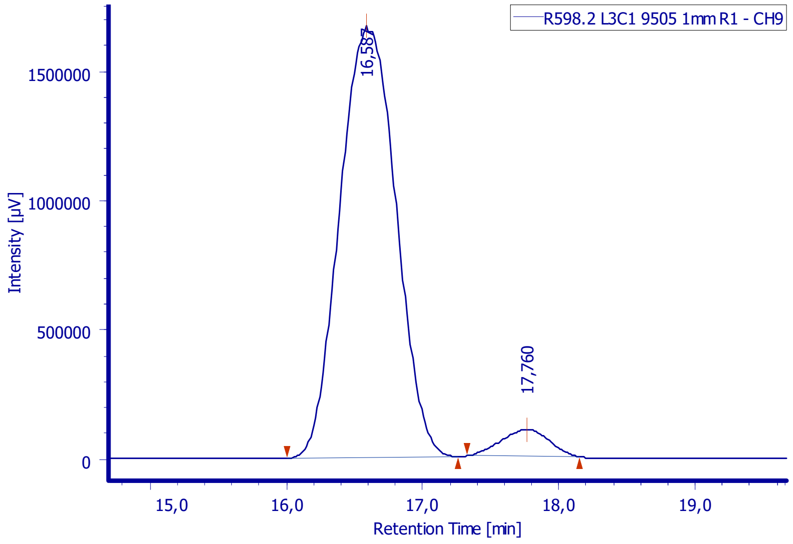

tR	Area	Area%
16,587	47931848	95,773
17,760	2115501	4,227

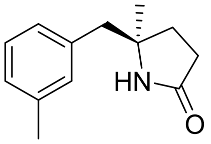

7G

The enantiomeric ratio was determined by HPLC analysis (Daicel Chiralpak IA, Hexane/^{i}PrOH = 95/5, flow rate = 1 mL/min, λ = 202 nm).

(±) 7G

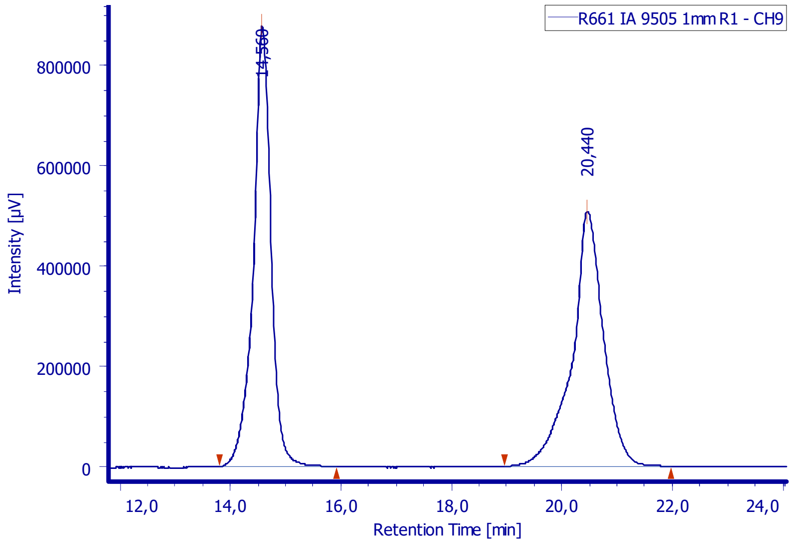

tR	Area	Area%
14,560	19590248	49,485
20,440	19998259	50,515

7G

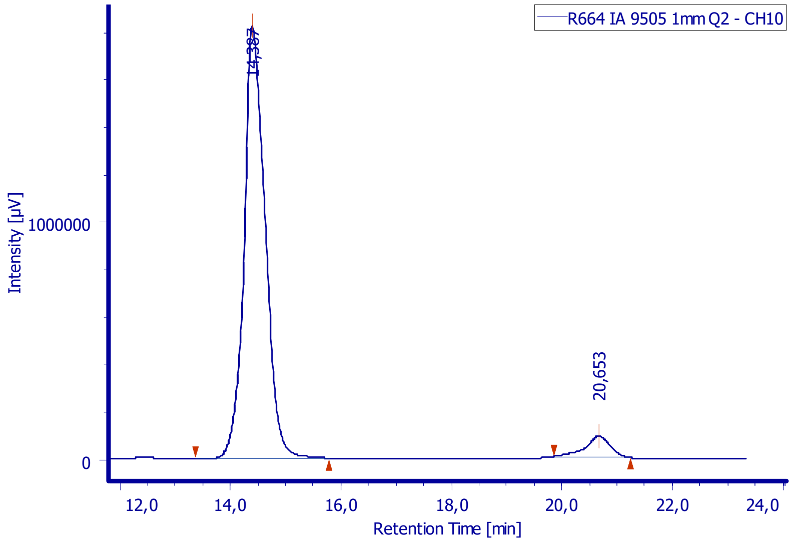

tR	Area	Area%
14,387	5623293	94,402
20,653	333459	5,598

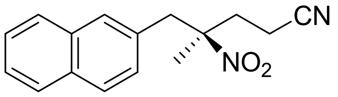

9

The enantiomeric ratio was determined by HPLC analysis (Daicel Chiralpak OD-H, Hexane/^{i}PrOH = 90/10, flow rate = 1 mL/min, λ = 202 nm).

(±) 9

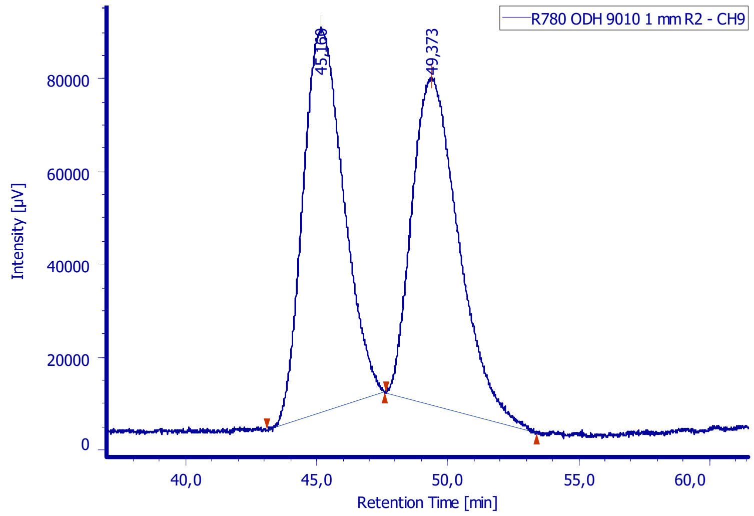

tR	Area	Area%
45,160	8903418	50,389
49,373	8765895	49,611

9

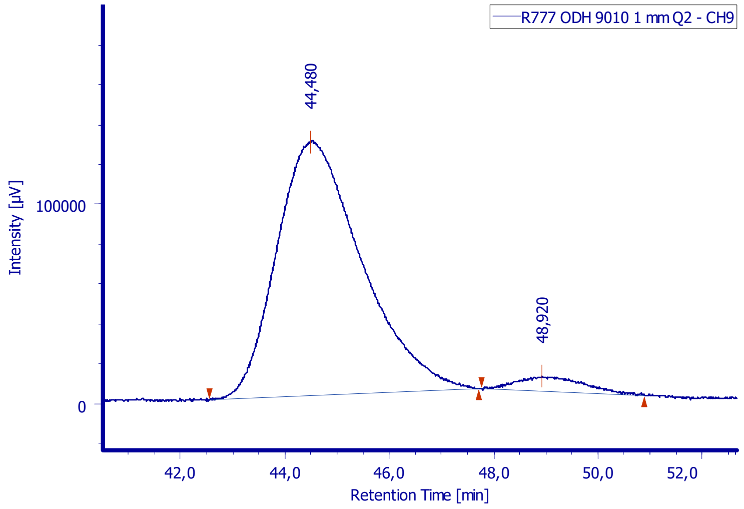

tR	Area	Area%
44,480	14280930	95,459
48,920	679346	4,541

8. ORTEP diagram of compound 9

X-Ray Analysis

View of the molecular structure of **9** with 50% probability displacement ellipsoids.

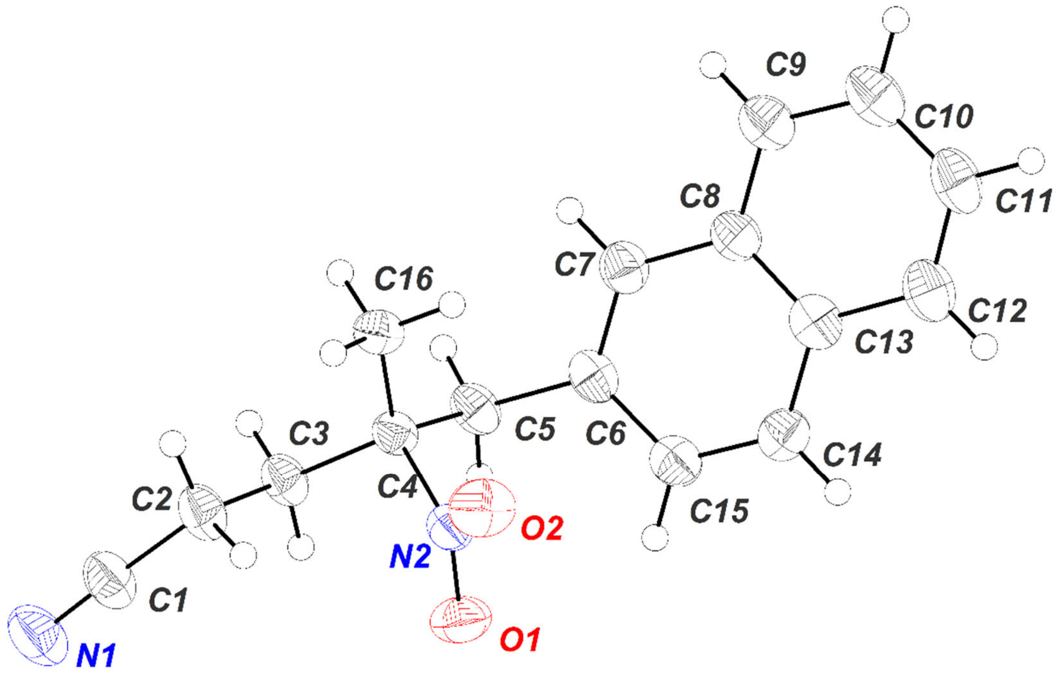

Single crystals of **9** were obtained by slowly evaporating a chloroform and hexane solution of **9** in a vial at ambient temperature. A suitable crystal was selected and mounted on a SuperNova, Dual, Cu at home/near, HyPix diffractometer. The crystal was kept at 170.00(10) K during data collection. Using Olex2, the structure was solved with the ShelXT structure solution program using Intrinsic Phasing and refined with the ShelXL refinement package using Least Squares minimisation. The X-ray data have been deposited at the Cambridge Crystallographic Data Centre (CCDC 2300164).

Table. Crystal data and structure refinement for 9.

Empirical formula	$C_{16}H_{16}N_2O_2$
Formula weight	268.31
Temperature/K	170.00(10)
Crystal system	monoclinic
Space group	$P2_1$
a/Å	8.1712(2)
b/Å	6.2090(2)
c/Å	13.9372(3)
α/°	90.0
β/°	102.737(2)
γ/°	90.0

Volume/Å^3	689.70(3)
Z	2
ρ_{calc}g/cm^3	1.292
μ/mm^{-1}	0.697
F(000)	284.0
Crystal size/mm^3	0.236 × 0.073 × 0.04
Radiation	CuKα (λ = 1.54184)
2Θ range for data collection/°	6.502 to 137.96
Index ranges	-9 ≤ h ≤ 9, -6 ≤ k ≤ 7, -16 ≤ l ≤ 16
Reflections collected	12327
Independent reflections	2356 [R_{int} = 0.0507, R_{sigma} = 0.0333]
Data/restraints/parameters	2356/1/182
Goodness-of-fit on F^2	1.127
Final R indexes [I>=2σ (I)]	R_1 = 0.0399, wR_2 = 0.1121
Final R indexes [all data]	R_1 = 0.0432, wR_2 = 0.1144
Largest diff. peak/hole / e Å^{-3}	0.22/-0.15
Flack parameter	0.1(3)
